# A UMLS-based spell checker for natural language processing in vaccine safety

**DOI:** 10.1186/1472-6947-7-3

**Published:** 2007-02-12

**Authors:** Herman D Tolentino, Michael D Matters, Wikke Walop, Barbara Law, Wesley Tong, Fang Liu, Paul Fontelo, Katrin Kohl, Daniel C Payne

**Affiliations:** 1 Bacterial Vaccine-Preventable Diseases Branch, Epidemiology and Surveillance Division, National Immunization Program, Centers for Disease Control and Prevention, Atlanta GA, 30333, USA; 2 Public Health Informatics Fellowship Program, Office of Workforce and Career Development, Centers for Disease Control and Prevention, Atlanta GA, 30333, USA; 3 Division for Heart Disease and Stroke Prevention, National Center for Chronic Disease Prevention and Health Promotion, Centers for Disease Control and Prevention, Atlanta GA, 30341, USA; 4 Immunization & Respiratory Infections Division, Centre for Infectious Disease Prevention & Control, Public Health Agency of Canada, Ottawa, Ontario K1A 0K9, Canada; 5 Honours Biology and Pharmacology Programme, McMaster University, Hamilton, Ontario L8S 4L8, Canada; 6 Office of High Performance Computing and Communications, National Library of Medicine, National Institutes of Health, Bethesda MD, 20894, USA; 7 Immunization Safety Office, Office of the Chief Science Officer, Centers for Disease Control and Prevention, Atlanta GA, 30333, USA

## Abstract

**Background:**

The Institute of Medicine has identified patient safety as a key goal for health care in the United States. Detecting vaccine adverse events is an important public health activity that contributes to patient safety. Reports about adverse events following immunization (AEFI) from surveillance systems contain free-text components that can be analyzed using natural language processing. To extract Unified Medical Language System (UMLS) concepts from free text and classify AEFI reports based on concepts they contain, we first needed to clean the text by expanding abbreviations and shortcuts and correcting spelling errors. Our objective in this paper was to create a UMLS-based spelling error correction tool as a first step in the natural language processing (NLP) pipeline for AEFI reports.

**Methods:**

We developed spell checking algorithms using open source tools. We used de-identified AEFI surveillance reports to create free-text data sets for analysis. After expansion of abbreviated clinical terms and shortcuts, we performed spelling correction in four steps: (1) error detection, (2) word list generation, (3) word list disambiguation and (4) error correction. We then measured the performance of the resulting spell checker by comparing it to manual correction.

**Results:**

We used 12,056 words to train the spell checker and tested its performance on 8,131 words. During testing, sensitivity, specificity, and positive predictive value (PPV) for the spell checker were 74% (95% CI: 74–75), 100% (95% CI: 100–100), and 47% (95% CI: 46%–48%), respectively.

**Conclusion:**

We created a prototype spell checker that can be used to process AEFI reports. We used the UMLS Specialist Lexicon as the primary source of dictionary terms and the WordNet lexicon as a secondary source. We used the UMLS as a domain-specific source of dictionary terms to compare potentially misspelled words in the corpus. The prototype sensitivity was comparable to currently available tools, but the specificity was much superior. The slow processing speed may be improved by trimming it down to the most useful component algorithms. Other investigators may find the methods we developed useful for cleaning text using lexicons specific to their area of interest.

## Background

The Institute of Medicine (IOM) in the United States identified patient safety as a key goal in the delivery of health care. In a 2004 report, "Patient Safety: Achieving a New Standard for Care," the IOM highlighted the importance of pursuing an applied research agenda on patient safety, focused on enhancing knowledge, developing tools, and disseminating results to maximize the impact of patient safety systems [1]. The capacity for using computer technology has grown rapidly in the domain of reporting adverse events following immunizations (AEFI), particularly with its increasing use in pharmacovigilance surveillance systems.

Bates et al. noted that manual chart review is an effective method for identifying different types of adverse events in the research setting but this approach is too costly for routine use. Nevertheless, they emphasized the role of analyzing the free-text components of electronic patient records to increase the chance of capturing these adverse events [2]. Currently, AEFI reports, such as those submitted to the U.S. Vaccine Adverse Event Reporting System (VAERS) [3], contain free-text components that need to be processed manually by human encoders. The clinical information contained in free text can be subsequently translated to controlled vocabulary codes for adverse events, such as the Coding Symbols for Thesaurus of Adverse Event Reaction Terms (COSTART), the Medical Dictionary for Regulatory Activities (MedDRA) or the World Health Organization Adverse Reaction Terminology (WHO-ART). However, four unique challenges arise from linguistic variation found in the free-text components of AEFI reports: (1) synonyms and paraphrases can refer to a single symptom; (2) medical concepts are recorded by providers using abbreviations and acronyms aligned to a particular care setting; (3) the same health care or clinical concept can be described using combinations of different parts of speech; and (4) words are often mistyped which can cause unpredictable errors [4]. In this paper we address challenges 2 and 4 to replace abbreviations and acronyms and correct misspelled words.

Ruch et al. state that the correction of spelling in medical records is a critical issue. They found that the rate of misspelling in medical records is 10% higher than the rate for other texts, such as those in newspapers [5]. In analyzing 124,993 unique tokens from 238,898 documents of a 590 MB corpus from the Oregon Health Sciences University electronic medical record, Hersh et al. discovered that around 7 % are misspelled words [6]. Fisk et al. estimated that the spelling error rate for a 2.8 million-document data warehouse from the Veterans Administration Medical Center in Connecticut was around 4% [7]. No figures for vaccine safety reports are known to have been published.

Outside of the medical domain, spelling correction as a problem area in text recognition and editing is not new. The advent and widespread use of optical character recognition devices in the 1990s have encouraged increased research activity to enable seamless conversion of large amounts of paper-based information into spell-corrected digital format. The rapid advances in computer hardware and software also mainstreamed the use of spell checkers in software development as well [8,9]. Kukich carried out an extensive and authoritative review of articles from this domain from the 1960s to the 1990s and we refer to the pertinent details of spell-correction methods described in that review throughout this paper. In describing automatic word correction research, Kukich enumerated three problem areas: (1) non-word error detection; (2) isolated-word error correction; and (3) context-dependent word correction [8].

In the medical domain, spelling correction has been applied to chief complaint data to expand acronyms, abbreviations and truncations and to correct spelling errors. Boyce et al. argue that standardization of data should improve the classification of chief complaint data as a first step in a syndromic classification pipeline [10]. Dara and Chapman, however, found only marginal improvement in the sensitivity of their Bayesian statistical classifier after pre-processing which includes spelling correction, expansion of abbreviation and removal of words that do not have clinical meaning [11].

As we worked with AEFI reports, we encountered two types of orthographic variation: (1) intended variations, which clinicians intentionally created to make documentation activities efficient; and, (2) non-intended variations, which resulted from spelling errors. The first type of variation can be addressed with regular expressions, or tiny pieces of text processing code usually written in Practical Extraction and Report Language or PERL [12]. These regular expressions are essentially concise, pattern-matching, rule-based algorithms used to expand common abbreviations. For example "Dx" is expanded to "diagnosis," "P/C" to "phone call," or "N/V" to "nausea and vomiting." The second type of variation can be addressed with the use of domain-specific lexicons and word lists.

**Figure 1:**
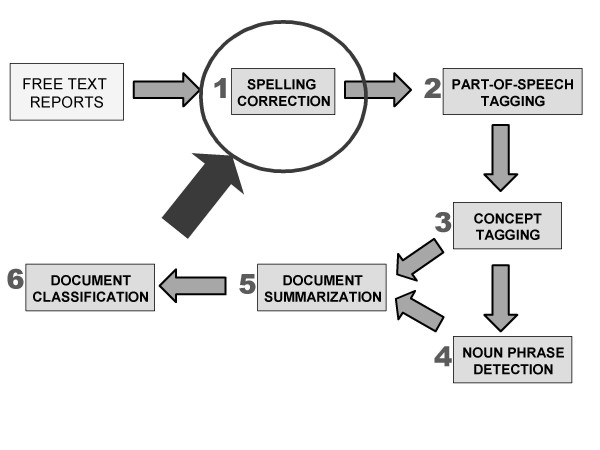
**Framework for concept extraction using the UMLS**. The natural language processing pipeline for this project is made up of the steps in this diagram. For this paper, we are focusing on step 1, spelling correction.

A collaborative workgroup of the Vaccine Analytic Unit in the Centers for Disease Control and Prevention (CDC) National Immunization Program (NIP), the Public Health Agency of Canada (PHAC), the Brighton Collaboration (BC) and the National Library of Medicine (NLM) is currently working on tagging free-text components of AEFI reports (Figure 1) with concepts found in the Unified Medical Language System (UMLS) [13]. The BC is an international voluntary collaboration to facilitate the development, evaluation and dissemination of high quality information about the safety of human vaccines [14]. The NLM develops, maintains and distributes UMLS.

The main goal of this paper was to describe the development and evaluation of spelling error correction algorithms as a first step in the natural language processing pipeline for classifying AEFI reports into UMLS concept clusters.

## Methods

### Data source

To begin developing these algorithms we constructed a corpus of vaccine safety reports. The body of this corpus was derived from paper records provided by the BC partners from the PHAC. For the period January 1, 2000 and May 15, 2005, a list of AEFI reports was obtained meeting the following two criteria: that they were not from either Quebec or Ontario (mostly electronically reported with little or no text), and did not pertain to the influenza vaccine (with little free text). From this list, reports were selected starting with the first report and every third thereafter, and a return to the second report and subsequent third thereafter until there were 100 reports. The selected reports were de-identified by our BC partners prior to processing and analysis. We extracted the free-text sections from these reports and entered them twice using a custom interface [see Additional file 2]. This same interface compared the two entries and visually provided cues about discrepancies between them [using source code in Additional file 15]. An algorithm within this interface also automated the assignment of documents to either the training (60%) set or the test (40%) set during raw data entry. We used the first data set for development and testing of required algorithms and the second for testing spelling checker performance.

### Algorithm development

The tools we used to develop the algorithms are publicly available. For rapid code prototyping, we used PHP (a recursive acronym for PHP Hypertext Pre-processor), a cross-platform, web scripting language that also facilitated the creation of the presentation layer of the prototype application [see Additional file 4] [15]. For database storage we used MySQL, an open source database that can be easily installed on any operating system (Windows, MacOS, Linux, and UNIX) [16]. We included algorithms that track and collect statistics on processing times and algorithm contribution to word list generation and word selection.

### Dictionary construction

According to Strohmaier et al., the perfect dictionary for post-correction satisfies three principles. First, the dictionary contains each word in the corpus. Second, the dictionary contains only words from the corpus. And third, for each input word, the dictionary stores the frequency of each word in the corpus [9]. For this project, we used the licensed 2005AA version of the UMLS from NLM and WordNet [see Additional file 16], a semantic lexicon from the Princeton Cognitive Sciences Laboratory [17] to construct our custom dictionary. We assumed that since this dictionary contains both domain-specific and generic words, it would provide adequate lexical coverage of vaccine safety reports. The UMLS has three knowledge sources: (1) the Semantic Network (SN), a high-level categorization of medical concepts [see Additional file 14]; (2) the Metathesaurus (MT), a knowledge base of concepts aggregated from source vocabularies that include AEFI-related controlled vocabularies like the International Classification of Diseases version 9 Clinical Modification (ICD-9-CM), the Coding Symbols and Thesaurus for Adverse Reaction Terminology (COSTART), the Medical Dictionary for Drug Regulatory Activities (MedDRA) and the World Health Organization Adverse Reaction Terminology (WHO-ART); and, (3) the Specialist Lexicon (SL), a set of tools and tables that serve as a specific resource for NLP [see Additional files 17 and 18]. We used two of the knowledge sources in this study: the MT and the SL. The UMLS SL contains a table for inflections (LRAGR), and another one for abbreviations (LRABR). These were extracted and combined with the WordNet tables to create a custom dictionary that has specific columns to enable error detection and correction, and that approximates Strohmaier's three principles of dictionary construction. Table 1 provides descriptions of table columns for this custom dictionary. The *word_id *column is the numeric, auto-generated, unique identifier for a word entry. The *word_str *column stores the word itself. The *word_ngram *column stores n-grams of words, which are overlapping n-character representations of a word. In this case we use two characters or bigrams. An example of what the *word_ngram *column contains can be found in Table 1. We obtained the source code of this n-gram algorithm from an open source program created by Saari [18].

**Table 1:** Column descriptions of the custom dictionary

**Column**	**Description**
word_id	Unique identifier
word_str	Dictionary word
word_ngram	Bigrams of the dictionary word. Example: "pediatrician" would have the following bigrams: *pe, ed, di, ia, at, tr, ri, ic, ci, ia, an*
word_metaphone	The metaphone value of the dictionary word. Example: pediatrician would have the metaphone *PTTRXN*
word_header	The first 4 characters of the word. Example: "pediatrician" would have the header *pedi*
word_anterior	The 4 characters after the first character of the dictionary word
word_posterior	The 4 characters before the last character of the dictionary word
word_fragment	If the dictionary word is longer than 10 characters the first 10 characters of the dictionary word

We used the *word_metaphone *column to sort the words according to how they sound in English. Kukich described a similar step in her review using the SOUNDEX function [8]. A metaphone, or a phonetic summary, for a word is generated by reducing it to a few key consonants. To create the metaphone of a word, we passed it through a PHP function called *metaphone *[15]. An example of a metaphone can be found in Table 1. We also included word fragments as columns: (1) the first 4 characters, called the *word_header *column; (2) characters 2–5, or the *word_anterior *column, to simulate a first character deletion; (3) the 4 characters before the last one, or the *word_posterior*, to simulate a last character deletion; and (4) the *word_fragment *column which is used to store the first 10 characters of the word if it is longer than 10 characters. We created a smaller dictionary with the same table structure as above to contain the words found in the training data set. Kukich described this step as dictionary partitioning [8]. We did this to speed up queries for spelling error detection by using a smaller number of records. This smaller dictionary has an additional frequency column to indicate how frequently the word has been used in the corpus as described by Strohmaier et al. [9].

### Cleaning free text with regular expressions

**Figure 2:**
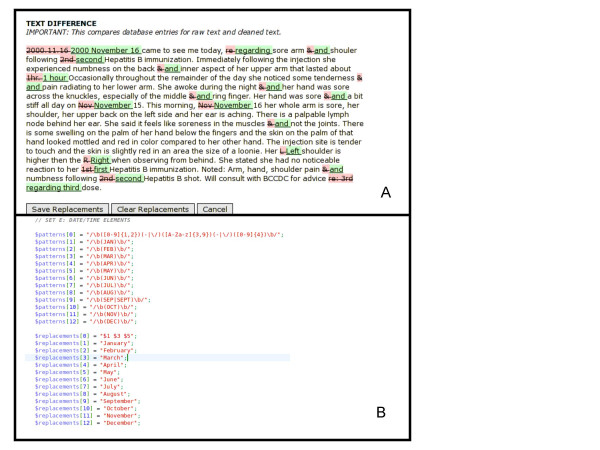
**Screenshots from the spell checker showing sample regular expression code and effect on free text**. This figure combines two screen shots. Box A shows the interface that displays changes to free text done by regular expression. Box B shows examples of regular expression code that changes abbreviated month names to their full form. Note how several instances of the abbreviated month "Nov" are detected and converted to the full form "November".

The use of PERL regular expressions is a well-described standard method for processing and transforming text in a wide range of applications that require character level text manipulation and pattern matching, from web search engines to bioinformatics applications [19]. We developed more than 200 regular expressions to transform abbreviations, contractions, and medical shortcuts that doctors and nurses use in the clinical setting. Figure 2 shows an example of what regular expressions do in our spell checker.

### Spelling checker steps

**Figure 3:**
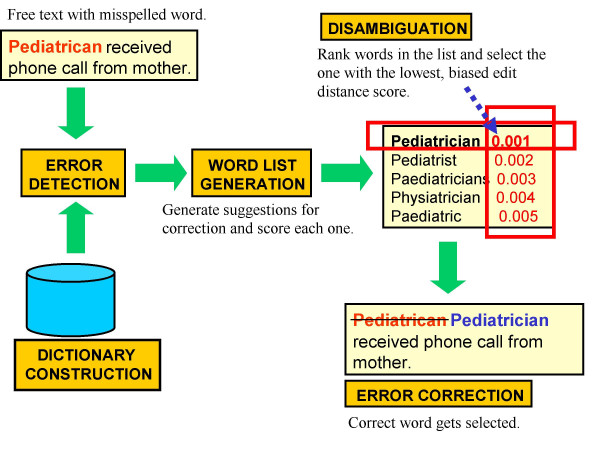
Stages of spelling correction.

In her review article, Kukich described three main steps that characterize isolated-word error correction methods used by various authors: error detection, candidate word generation and word ranking [8]. In our paper, the essential steps we carried out in spelling correction after cleaning with regular expressions were (1) error detection, (2) word list generation, (3) word list disambiguation and (4) error correction (Figure 3). Steps 3 and 4 in our methods correspond to Kukich's Step 3.

### Step one – error detection

We determined whether or not a word in the AEFI report was misspelled by comparing it with entries, first, using the smaller custom dictionary and then, the bigger dictionary if the word was not found in the smaller one.

### Step two – word list generation

From preliminary testing of a concept extraction tool, we found that UMLS concepts come from nouns, noun phrases, verbs, adjectives, and adverbs [20]. It was therefore logical for us to focus on these parts of speech that would have the highest yield for concepts. We used *MedPost*, a part-of-speech (POS) tagger developed by the U.S. National Institutes of Health (NIH) to provide the tags that we needed to narrow down our search for possible sources of UMLS concepts [see Additional file 9]. The MedPost POS tagger was trained on the Medline corpus and is described elsewhere [21]. The POS tags also helped us carry out part-of-speech disambiguation in the next step below. We extracted the word list from the custom dictionary using these word-list generation algorithms: metaphone, header, N-gram, transposition, deletion, insertion and substitution. The **metaphone **algorithm was used to search for similar sounding words. The **header **algorithm looked for words with the same first 4 characters. The **N-gram **algorithm searched for words containing the next 4 characters after the first one, and those containing 4 characters before the last one (essentially mid-string searches). The **transposition **algorithm searched for words where any 2 characters are switched. The **deletion **algorithm searched for word matches by sequentially inserting a wildcard character in the misspelled word to simulate a character deletion. The **insertion **algorithm searched for word matches by sequentially deleting a character in the misspelled word to simulate a character insertion. The **substitution **algorithm searched for word matches by simulating character substitution. The last four algorithms are based on Damerau's findings that 80% of all misspelled words contained a single instance of one of the four error types (transposition, deletion, insertion and substitution), also known as a class of single-error misspellings [22].

### Step three – word list disambiguation

The objective of this step was to rank the candidate words by determining which word from the word list in step 2 had the lowest Levenshtein score, which is the number of edits needed to transform a misspelled word to any of its possible corrections [23]. This candidate word with the lowest Levenshtein score then served as the correction word. A built-in PHP function, called *levenshtein*, calculated the number of possible corrections (the Levenshtein or edit-distance score) by simulating the four types of errors (insertion, deletion, transposition and substitution). During edit-distance scoring, two or more words might still have the same Levenshtein score, a "deadlock" or a "tie" situation for the spell checker. We applied several word sense disambiguation algorithms to enable the spell checker to refine its ranking by "smoothing" the Levenshtein score. We used the following smoothing algorithms: (1) the **UMLS concept algorithm**, if the candidate word maps to a UMLS Concept Unique Identifier (CUI), regardless of semantic type; (2) the **metaphone algorithm**, assuming words that sound the same have the greater propensity to be misspelled; (3) the **homonym algorithm**, if the misspelled word had the same first letter and metaphone as the candidate word; (4) the **N-gram algorithm**, if the misspelled word contains similar character subsets as the candidate word, i.e., "wretch" and "retch"; (5) the **length algorithm**, assuming the clinician did not intend to misspell with a longer term; (6) the **POS algorithm**, if the candidate word has a similar part-of-speech tag as the misspelled word; and (7) the **history algorithm**, if the candidate word already existed with a probability distribution in our NLP database. The spell checker applied all six smoothing algorithms to the entire candidate word list to help discriminate between similar scores. With the exception of the N-gram algorithm, the rest of the smoothing algorithms are context-dependent. This smoothing step produced a final set of ranked words from which the lowest Levenshtein score was used to select the correction.

### Step four – error correction

After disambiguating and selecting the most probable correct word using the ranking score described above, we replaced the misspelled word with the correct one.

### Performance measurements

These measurements entailed a comparison of how the automated correction with the spell checker performed compared to "gold standard" manual correction. We calculated the sensitivity of the spell checker by comparing the number of words that both the spell checker and the human observers correctly identified. We calculated specificity as the proportion of words identified by the human observers as correct which the spell checker did not change. We are doing isolated-word spell correction [8] which does not allow for syntactic context analysis.

## Results

### Data source

The training and test data sets extracted from the 100 AEFI reports served as inputs to the spell checker algorithms which are described below. The training data set consisted of 12,056 words while the test data set had 8,131 words.

### Use of UMLS and spell checker development

We built a custom dictionary as part of the spell checker using the UMLS SL and WordNet with specialized columns (Table 1). This dictionary helped in error detection and correction by providing lexical coverage and the maximum the number of words that can be included in a word list from which to choose the correction for a misspelled word. This dictionary was large as it contained close to 498,000 terms and took approximately 4–5 minutes to build using a Pentium 4 1 GHz laptop computer with 512 MB RAM and a 60 GB 7200-RPM hard disk. We stored this custom dictionary in a MySQL database. The smaller, partitioned dictionary helped us to rapidly look up potentially misspelled words by providing words that have been corrected and are found in the training set.

### Cleaning with regular expressions

The regular expressions transformed 10% of words from the training set and 9% from the test set. This means many abbreviations and contractions, which the spell checker could have potentially detected as spelling errors, were eliminated before spell-checking was done. It is possible for regular expressions to transform text incorrectly so they were only applied to abbreviations and contractions which could be unambiguously transformed.

### Spell checker steps 1–4

After developing the algorithms, we trained the spell checker using the training data set. To train the spell checker, we developed a manual spelling correction tool that allowed us to correct any errors made in candidate selection from the list of potential spelling corrections. If the correct word is not in the database, this tool also enabled us to enter the correct spelling so it became part of the custom dictionary and was used in the selection process if the misspelled word was encountered by the spell checker again. This tool also enabled us to instruct the spell checker to ignore a word that was incorrectly marked as a misspelling. After training, we applied the algorithms on the test set and compared the outputs with the results of our manual correction.

To help us assess the effect of each algorithm during the generation of word lists and word sense disambiguation, we incorporated data collection algorithms to store internal counts, i.e., number of terms generated from each word list algorithm, and the percentage contribution of disambiguation algorithms towards the final decision. Table 2 shows the mean relative contributions of the different word list-generation algorithms. More than half of the word list was generated by the header algorithm (training set value is 55%), which searched the database using the first four letters of the misspelled word. The N-Gram algorithm, which uses character-level matching, contributed the next highest mean proportion (training set value is 20%). The rest of the algorithms simulated the four sources of spelling errors (insertion, deletion, transposition and substitution) and contributed their own lists depending on the morphology of the misspelled word. For example, if there was a potential insertion, as in "wretch" vs. "retch," the spell checker detects the misspelling and generates a word list using the insertion algorithm. Table 3 shows the mean contribution of the word sense disambiguation algorithms. Here, the N-Gram algorithm contributed the highest proportion to smoothing the Levenshtein score.

**Table 2:** Mean contribution of algorithms to word list generation

**WORD LIST ALGORITHM**	**TRAINING SET n = 12,056**	**TEST SET n = 8,131**
N-Gram	20%	13%
Header	55%	59%
Metaphone	8%	4%
Transposition	1%	3%
Deletion	5%	6%
Substitution	5%	5%
Insertion	6%	10%

TOTAL	100%	100%

**Table 3:** Mean contribution of algorithms to word sense disambiguation (smoothing) and ranking

**SMOOTHING ALGORITHM**	**TRAINING SET n = 12,056**	**TEST SET n = 8,131**
Concept	12%	13%
Homonym	1%	1%
N-Gram	55%	53%
Metaphone	5%	4%
Length	14%	14%
Part-of-speech	10%	11%
History	3%	4%

TOTAL	100%	100%

### Performance measurements

We determined sensitivity, specificity and positive predictive value by running the spell checker on 12,056 words from the training set and on 8,131 words for the test set, and comparing these results with manual correction for misspelled words. The results of this comparison between manual correction and the spell checker are shown in Table 4. During training, the spell checker had a sensitivity of 93% (95% CI: 93%–94%), a specificity of 100% (95% CI: 100%–100%), and a PPV of 64% (95% CI: 63%–65%). The spell checker as applied to the test data set had a sensitivity of 74% (95% CI: 74%–75%), a specificity of 100% (95% CI: 100%–100%), and a PPV of 47% (95% CI: 46%–48%). The prevalence of spelling errors in the test set is 0.5%. The spell checker performed 770 regular expression transformations (9%) and 68 spelling corrections (1%) out of 8,131 words in the test set and had a mean processing time of 0.0006 second per word for transformation with regular expressions and 0.06 second per word for spelling correction.

**Table 4:** Spell checker performance measures

**PARAMETERS**	**TRAINING SET n = 12, 056**			**TEST SET n = 8,131**		
	
	**Value**	**95% CI**		**Value**	**95% CI**	
				
		**Lower**	**Higher**		**Lower**	**Higher**
Sensitivity (%)	93	93	94	74	74	75
Specificity (%)	100	100	100	100	100	100
Recovery (%)	85	84	85	68	67	69
Positive Predictive Value	64	63	65	47	46	48
Regular expression transformations	1,217 (10%)			770 (9%)		
Words corrected	105 (1%)			68 (1%)		
Processing time per word (second)	0.07			0.06		

## Discussion

The use of NLP to extract information from free-text reports has previously been reported in pathology, radiology, and emergency medicine. Notable examples include the early work of Friedman et al. on the MedLee natural language processor [24], Mamlin et al. with cancer-related free text [25] and Mendonca et al. for detection of pneumonia in infants from radiology reports [26]. Mitchell, et al. describe information extraction using NLP for pathology reports [27]. Travers and Haas, and Chapman et al. separately described the use of free-text processing for chief complaints in emergency department narrative reports [28,29], ushering the use of NLP in advanced disease surveillance.

Towards the use of NLP to extract information from AEFI reports, we utilized the UMLS MT and SL for word-sense disambiguation during spell checking of free text. Although the UMLS SL has a spell suggestion tool called GSPELL [30-32], we decided not to use it because of its known limitations, e.g., if the spelling error is the first or last character of the word the spell-correction suggestions will most likely be wrong. Crowell et al. attributed this to the use of a "bathtub" heuristic and recommended in a future release for it to use two additional techniques: truncation retrieval and a conservative stemming heuristic [30]. In our spell-correction tool, we used a technique similar to truncation retrieval and forward and backward stemming techniques to ameliorate the pitfalls of GSPELL.

In addition, GSPELL using a domain-specific dictionary was paradoxically outperformed by ASPELL [33] using a generic dictionary. ASPELL performed better on three areas of performance: (1) whether the correct suggestion was ranked number one; (2) whether the correct suggestion was ranked in the top ten; and (3) whether the correct suggestion was found at all. [30]. However, as we integrated ASPELL into our spelling correction tool, a cursory examination of the word lists generated by ASPELL pointed out that it would not effectively retrieve domain-specific terms that are misspelled since it used a generic dictionary.

As a first step in our NLP pipeline for processing AEFI reports, we have already used the tool to feed spell-checked data into the second step, which involves tagging the free-text reports with UMLS concepts so we could classify reports later. We described the initial results of the concept tagging in a conference poster [20]. The processing time of 0.06 second per word is slow and this system would probably be too slow for much larger data sets.

The spell checker as applied to the test data set had a sensitivity of 74% (95% CI: 74%–75%), a specificity of 100% (95% CI: 100%–100%), and a PPV of 47% (95% CI: 46%–48%). A sensitivity of 74% means the spell checker missed 26% of the errors or 11 out of 43 errors humans identified. Of the 68 errors identified by the spell checker only 32 were identified as errors by humans, hence a PPV of 47%. In other words, the system potentially put in more errors than it corrected. However, since the prevalence of errors was only 0.5%, this partially explains the low PPV, higher sensitivity and the high specificity. Contrary to what we assumed earlier, this turned out to be a relatively clean data set.

We built in algorithms to collect measurements on processing times and algorithm contribution to word list generation and word selection. These measurements reveal that though we expected regular expressions to consume a lot of time, the actual time spent by the spell checker for processing the test set with regular expressions is 0.0006 second per word. The rest of the algorithms took 0.06 second per word.

### Future directions

The regular expressions that removed abbreviations and shortcuts are tiny pieces of code that can actually be stored in the database and updated as needed to accommodate a different set of abbreviations and shortcuts existing in a different context. This makes the spell checker easily configurable and adaptable to different settings such as in syndromic surveillance from chief complaints where substantial word variation exists.

As AEFI surveillance systems are converted to electronic formats, unique opportunities arise for large-scale processing of their free-text components. Together with the use of structured data in AEFI reports, the extraction of information as concepts from free-text components augments the pool of data for analysis and subsequently enables more complete use of these reports for pharmacovigilance studies. Once concepts have been identified in free text, we could retrieve conceptually relevant journal articles from PubMed as suggested by the work of Brennan et al. [34] and electronically annotate AEFI reports with these to facilitate review and adjudication.

### Limitations

We encountered several challenges in implementing this project. First, a large number of non-standard abbreviations and contractions were used in the clinical setting, so here we used manual crafting of regular expressions to expand abbreviations that would otherwise be missed by other algorithms. Second, we also encountered culturally-bound words, for example, "loonie," which is the nickname for the Canadian dollar coin. This word came up as misspelled when actually it is used as a clinical measure of size for rashes or swelling (as Americans would use the size of a quarter). Third, because we obtained paper-based free-text reports, we had to manually transcribe them into electronic form. This may have introduced recognition or perceptual errors despite our attempted data quality measures such as double-entry of free text.

In her extensive review, Kukich described five levels of natural language processing constraints: (1) a lexical level; (2) a syntactic level; (3) a semantic level; (4) a discourse-structure level; and, (5) a pragmatic level [8]. We could map these constraints to the type of correction that needed to be done on free text. This spelling checker performed corrections at the lexical level and did rudimentary syntactic analysis based only on part-of-speech similarity between the misspelled word and the candidate word. This technique is a type of syntactic correction because it corrects the substitution of a word whose syntactic category did not fit its context. We did not do semantic-, discourse-structure and pragmatic-level correction since our overall goal was to be able to extract concepts from correctly spelled words using algorithms, not necessarily to create syntactically correct text. Moreover, it was extremely difficult to standardize syntactic correction with the way the free text was written. Some reports lacked complete sentences and included phrases with bullet points, making it difficult to assess subject verb agreement.

A very large dictionary from which to generate word lists for correction is useful but may become a limitation in the following ways. First, the probability of the dictionary containing a misspelled word itself becomes higher with increasing size. For example, we encountered at least one instance of a misspelled word appearing in the candidate word list and being selected as the correct term. Second, it is challenging to clean a large dictionary containing close to half a million entries. Third, database lookup time increases with the number of rows in the dictionary. This may be mitigated by using carefully constructed table indexes for search columns and partitioning the dictionary into two: a smaller dictionary with words that are commonly used and a bigger dictionary from which the words in the smaller dictionary are drawn. The advantage of using this large dictionary, on the other hand, is more complete coverage of the domain content, preventing the spell checker from marking correct words as incorrect.

The smoothing algorithms we applied to the Levenshtein score also have limitations. For example, the concept algorithm may inadvertently point to potential corrections that are actually semantically distant from the misspelled word. Since the UMLS is a large-scale knowledge base of the health care domain, it may contain homographs from domains other than that of AEFIs. In addition, it is difficult to address an abbreviation or acronym with more than one meaning. We addressed this issue by writing regular expressions that make use of context from surrounding words or tokens. Otherwise, when faced with an intractable situation we just left the acronym or abbreviation alone.

## Conclusion

We created a prototype spell checker which we intended to use as a pipeline NLP tool for processing AEFI reports. We used the UMLS SL as the primary source of dictionary terms and the WordNet lexicon as a secondary source. We used the UMLS as a domain-specific source of dictionary terms with which we compared potentially misspelled words in the corpus.

With a speed of 0.06 word per second, the spell checker should not be used for cleaning terabyte-size files assuming there are 150,000 words per megabyte. The speed limitations come from computing-intensive word-list generation and smoothing algorithms, which are meant to correct non-regular variations which resulted from spelling errors. In this data set, these algorithms transform a mere 1% of tokens and entail a 100-fold increase in processing time compared to processing with regular expressions which transform 9% of tokens into terms which can be mapped to UMLS concepts using a concept tagger in the pipeline. The vaccine safety reports we used to test the tool had an average of 152 words per document. A stream of documents going through the pipeline of a surveillance systems means the document processing rate would be 6 documents per minute. This processing rate will accommodate the usual inflow of reports from a passive system such as VAERS, which contains about the same amount of free text as the test documents. During a 10-year period (1991–2001) VAERS received 128,717 reports out of more than 1.9 billion doses of vaccines distributed [35]. In addition, a PPV of 47% indicates that these algorithms introduce more errors into the process than they correct. In other corpora with higher spelling error rates, these spelling correction algorithms would be more useful. Our next step will be to trim down the time- and processor-intensive algorithms to the most useful components and rely primarily on transformations using regular expressions since this approach offers the best return on investment of computing resources.

## Competing interests

The author(s) declare that they have no competing interests.

## Authors' contributions

HT developed the concept extraction project, developed and tested the algorithms and contributed to the processing of free-text data, and created draft report. MM contributed to research design, linguistics support, and algorithm testing and processing of free-text data. WW extracted the sample of AEFI reports system and contributed immunization safety expertise. BL supervised the sampling and contributed domain and locale-specific immunization safety expertise. WT assisted with sample extraction and performed preliminary processing of AEFI reports. PF contributed to research design, shared UMLS expertise, and supervised the NLM portion of the research. FL assisted in refining the algorithms interacting with the UMLS. KK provided support and supervision for the BC collaboration and contributed to research design. DP provided support and supervision and contributed to research design. All authors read and approved the final manuscript.

## Pre-publication history

The pre-publication history for this paper can be accessed here:



## Supplementary Material

Additional file 1 Tables. Document contains tables: column descriptions of the custom dictionary, mean contribution of algorithms to word list generation, mean contribution of algorithms to word sense disambiguation (smoothing) and ranking, and spell checker performance measures. Click here for file

Additional file 2 Raw data. Contains text files used for training and evaluation of algorithms Click here for file

Additional file 3 README text file. Instructions for setting up the database and application Click here for file

Additional file 4 Source code. Software platform used to launch modules for spell checker Click here for file

Additional file 5 Graphviz application directory. Sets up the graphviz application directory for storing outputs of graphing application Click here for file

Additional file 6 Images application directory. Sets up directory for storing images related to software platform Click here for file

Additional file 7 Index file for software platform. Index file for software platform Click here for file

Additional file 8 JPGRAPH application directory. Sets up JPGRAPH directory and source code used to draw graphs for software platform Click here for file

Additional file 9 MEDPOST directory. Sets up MEDPOST part-of-speech tagger and related files Click here for file

Additional file 10 Database tables – NLP1. Database tables have been subdivided into smaller files for uploading to BMC Click here for file

Additional file 11 Database tables – NLP2. Database tables have been subdivided into smaller files for uploading to BMC Click here for file

Additional file 12 OWL application directory. Sets up directory for storing OWL (Ontology Web Language) files Click here for file

Additional file 13 RAP application directory. Sets up directory for RAP (RDF application for PHP) source code Click here for file

Additional file 14 Database tables – UMLS Semantic Network. Database tables have been subdivided into smaller files for uploading to BMC Click here for file

Additional file 15 TEXDIFF application directory. Sets up directory for TEXTDIFF (highlights difference between two texts) source code Click here for file

Additional file 16 Wordnet database tables. Database tables have been subdivided into smaller files for uploading to BMC Click here for file

Additional file 17 Database tables – UMLS Lexicon Part 1. Database tables have been subdivided into smaller files for uploading to BMC Click here for file

Additional file 18 Database tables – UMLS Lexicon Part 2. Database tables have been subdivided into smaller files for uploading to BMC Click here for file
