# Supplementary material for: A UMLS-based spell checker for natural language processing in vaccine safety
Source: BMC Med Inform Decis Mak. 2007 Feb 12;7:3. doi: 10.1186/1472-6947-7-3 (PMC1805499; doi:10.1186/1472-6947-7-3)
Supplement: Additional file 1 — Tables. Document contains tables: column descriptions of the custom dictionary, mean contribution of algorithms to word list generation, mean contribution of algorithms to word sense disambiguation (smoothing) and ranking, and spell checker performance measures. [file 1472-6947-7-3-S1.doc]

Table 1. Column descriptions of the custom dictionary

| **Column** | **Description** |
| --- | --- |
| word_id | Unique identifier |
| word_str | Dictionary word |
| word_ngram | Bigrams of the dictionary word. Example: “pediatrician” would have the following bigrams: *pe, ed, di, ia, at, tr, ri, ic, ci, ia, an* |
| word_metaphone | The metaphone value of the dictionary word. Example: pediatrician would have the metaphone *PTTRXN* |
| word_header | The first 4 characters of the word. Example: “pediatrician” would have the header *pedi* |
| word_anterior | The 4 characters after the first character of the dictionary word |
| word_posterior | The 4 characters before the last character of the dictionary word |
| word_fragment | If the dictionary word is longer than 10 characters the first 10 characters of the dictionary word |

Table 2. Mean contribution of algorithms to word list generation

| **WORD LIST ALGORITHM** | **TRAINING SET**  **n = 12,056** | **TEST SET**  **n = 8,131** |
| --- | --- | --- |
| N-Gram | 20% | 13% |
| Header | 55% | 59% |
| Metaphone | 8% | 4% |
| Transposition | 1% | 3% |
| Deletion | 5% | 6% |
| Substitution | 5% | 5% |
| Insertion | 6% | 10% |
| TOTAL | 100% | 100% |

Table 3. Mean contribution of algorithms to word sense disambiguation (smoothing) and ranking

| **SMOOTHING ALGORITHM** | **TRAINING SET**  **n = 12,056** | **TEST SET**  **n = 8,131** |
| --- | --- | --- |
| Concept | 12% | 13% |
| Homonym | 1% | 1% |
| N-Gram | 55% | 53% |
| Metaphone | 5% | 4% |
| Length | 14% | 14% |
| Part-of-speech | 10% | 11% |
| History | 3% | 4% |
| TOTAL | 100% | 100% |

Table 4. Spelling checker performance measures

| **PARAMETERS** | **TRAINING SET**  **n = 12, 056** | | | **TEST SET**  **n=8,131** | | |
| --- | --- | --- | --- | --- | --- | --- |
|  | **Value** | **95% CI** | | **Value** | **95% CI** | |
|  |  | **Lower** | **Higher** |  | **Lower** | **Higher** |
| Sensitivity (%) | 93 | 93 | 94 | 74 | 74 | 75 |
| Specificity (%) | 100 | 100 | 100 | 100 | 100 | 100 |
| Recovery (%) | 85 | 84 | 85 | 68 | 67 | 69 |
| Positive Predictive Value | 64 | 63 | 65 | 47 | 46 | 48 |
| Regular expression transformations | 1,217 (10%) |  |  | 770 (9%) |  |  |
| Words corrected | 105 (1%) |  |  | 68 (1%) |  |  |
| Processing time per word (second) | 0.07 |  |  | 0.06 |  |  |
