# Supplementary figures and images for: A UMLS-based spell checker for natural language processing in vaccine safety
Source: BMC Med Inform Decis Mak. 2007 Feb 12;7:3. doi: 10.1186/1472-6947-7-3 (PMC1805499; doi:10.1186/1472-6947-7-3)

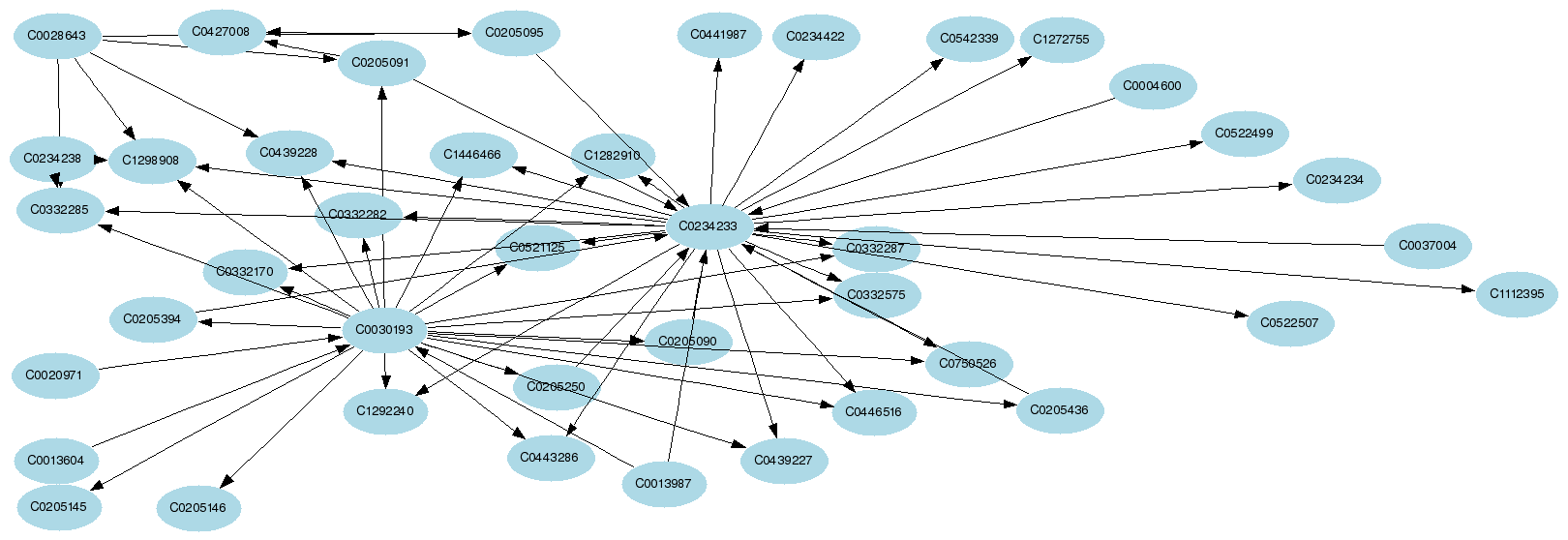

Supplement: Additional file 4 — Source code. Software platform used to launch modules for spell checker [file 1472-6947-7-3-S4.gz › game/graph/0119755.dot.png]

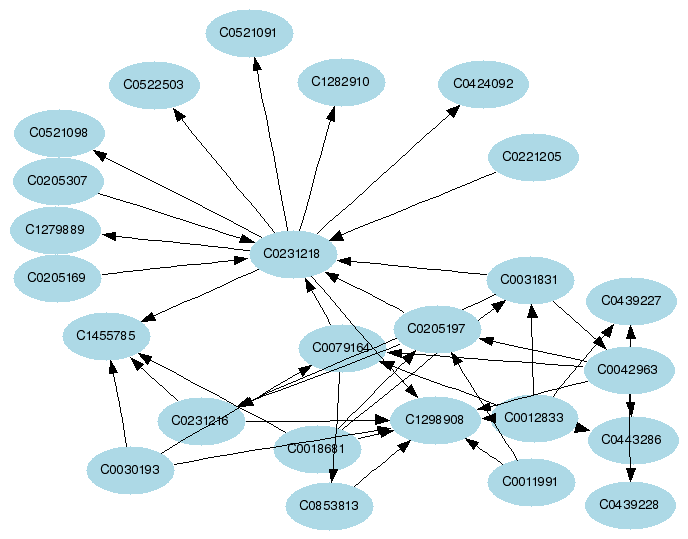

Supplement: Additional file 4 — Source code. Software platform used to launch modules for spell checker [file 1472-6947-7-3-S4.gz › game/graph/0119904.dot.png]

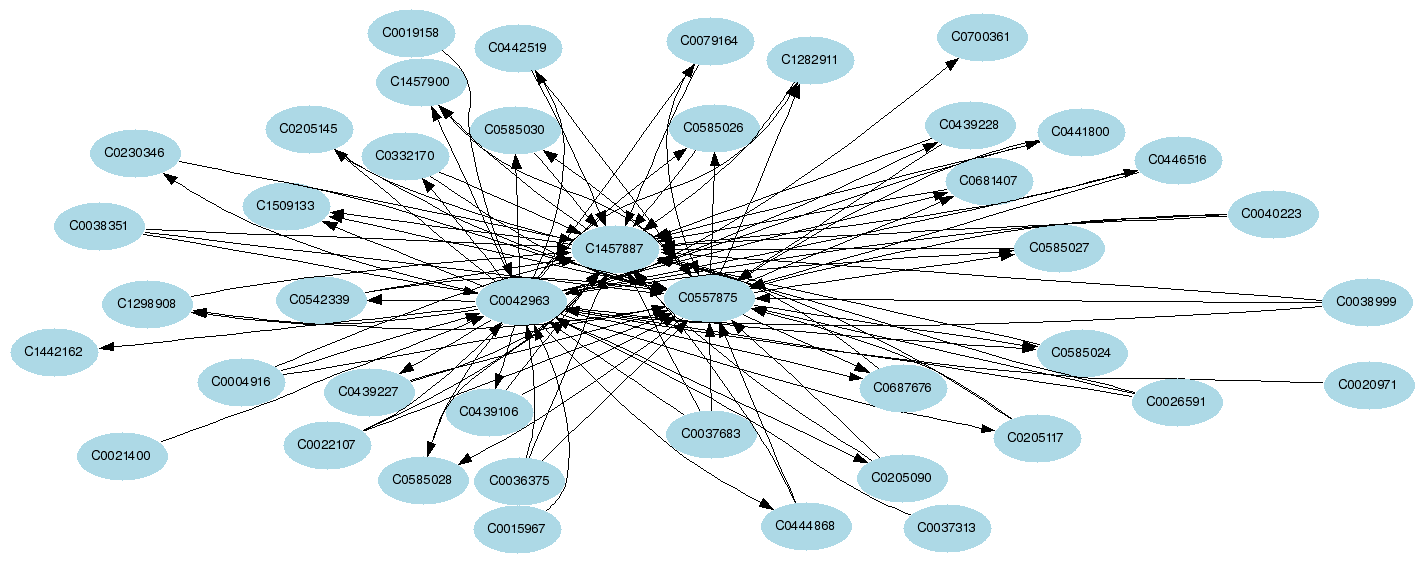

Supplement: Additional file 4 — Source code. Software platform used to launch modules for spell checker [file 1472-6947-7-3-S4.gz › game/graph/0400034.dot.png]

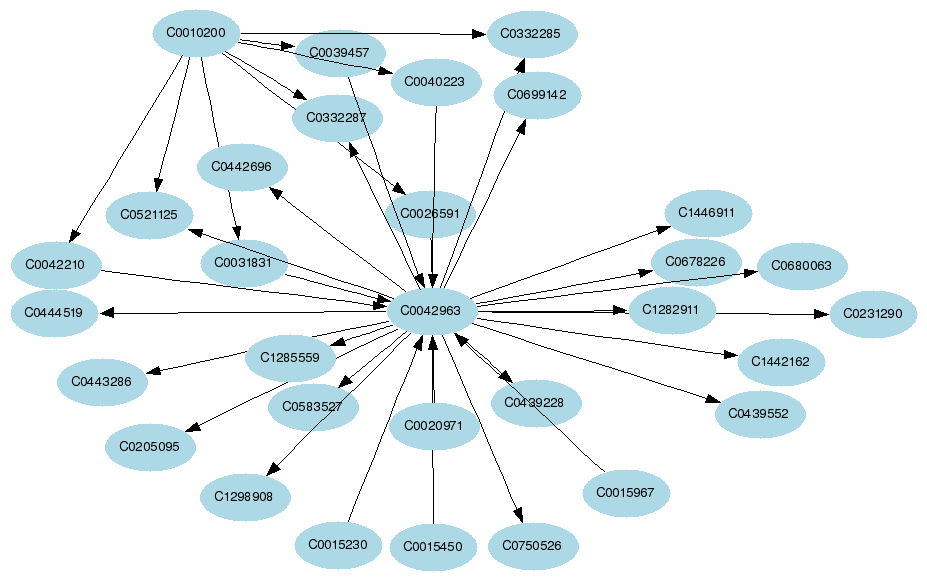

Supplement: Additional file 4 — Source code. Software platform used to launch modules for spell checker [file 1472-6947-7-3-S4.gz › game/graph/10.dot.png]

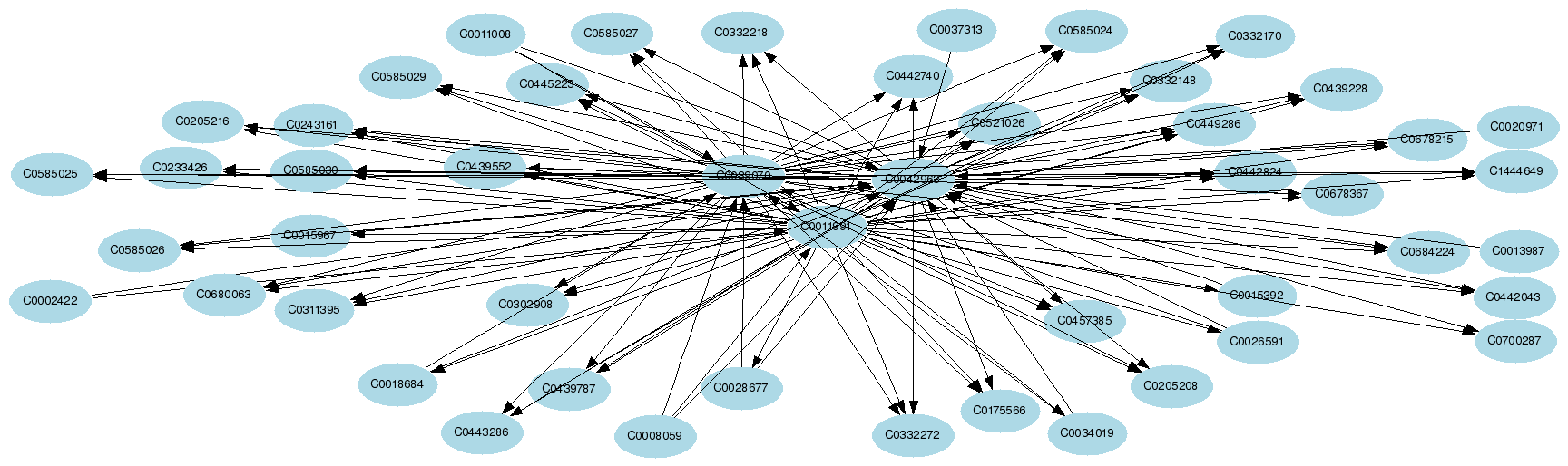

Supplement: Additional file 4 — Source code. Software platform used to launch modules for spell checker [file 1472-6947-7-3-S4.gz › game/graph/103.dot.png]

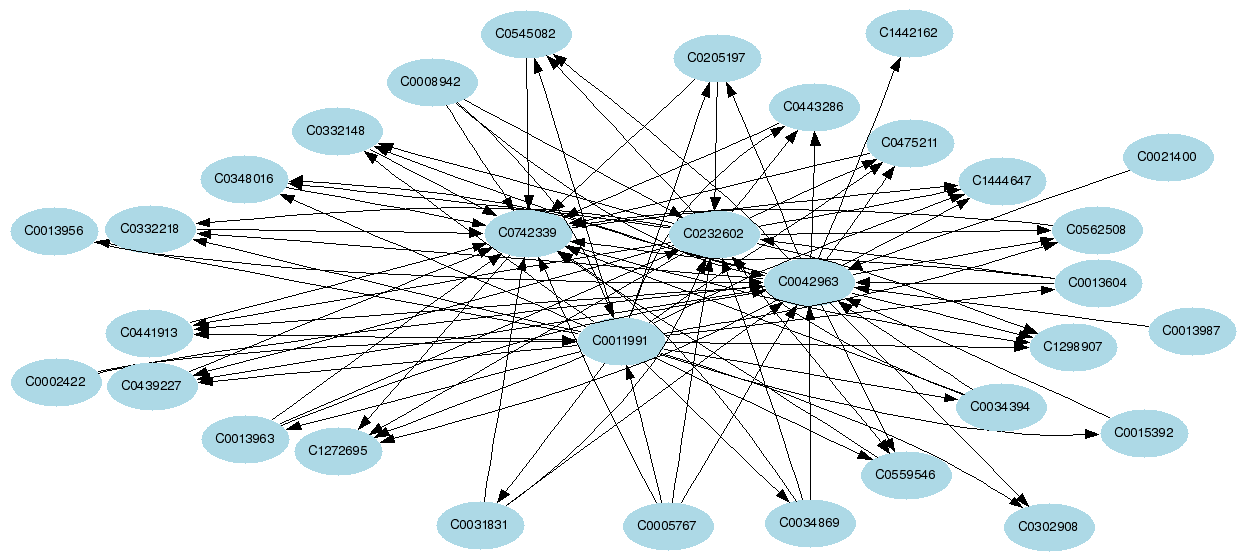

Supplement: Additional file 4 — Source code. Software platform used to launch modules for spell checker [file 1472-6947-7-3-S4.gz › game/graph/10394.dot.png]

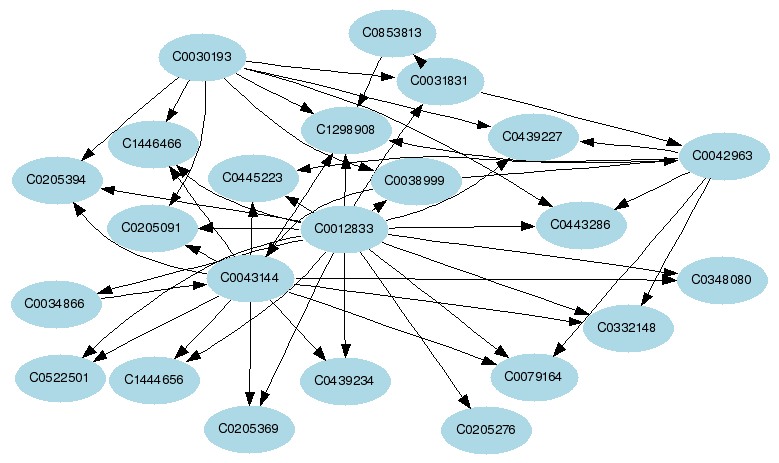

Supplement: Additional file 4 — Source code. Software platform used to launch modules for spell checker [file 1472-6947-7-3-S4.gz › game/graph/106.dot.png]

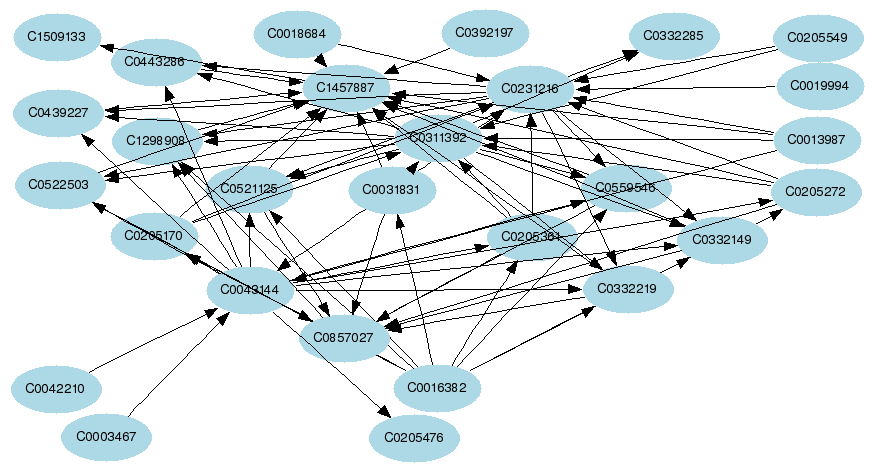

Supplement: Additional file 4 — Source code. Software platform used to launch modules for spell checker [file 1472-6947-7-3-S4.gz › game/graph/113.dot.png]

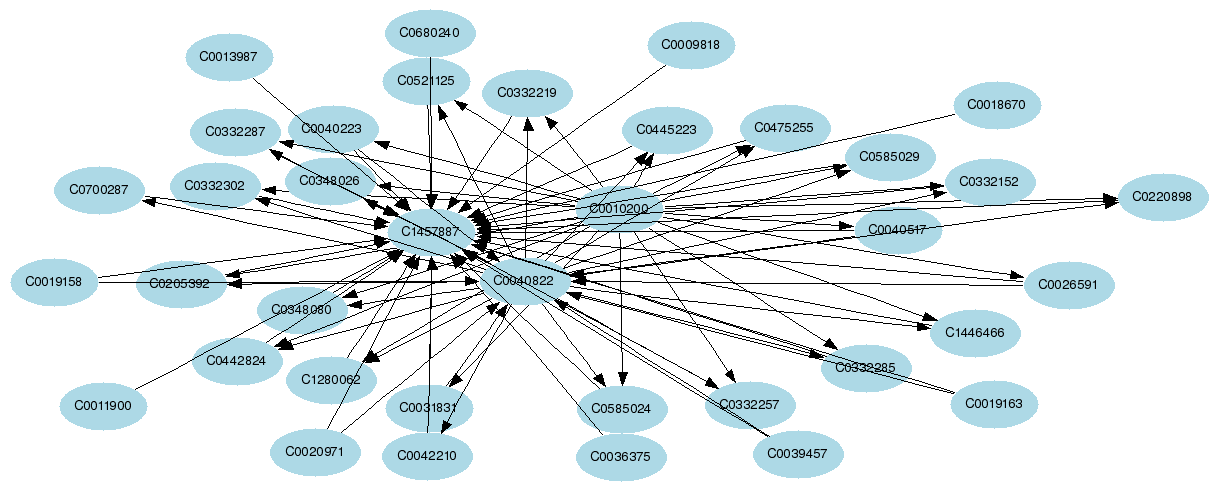

Supplement: Additional file 4 — Source code. Software platform used to launch modules for spell checker [file 1472-6947-7-3-S4.gz › game/graph/121.dot.png]

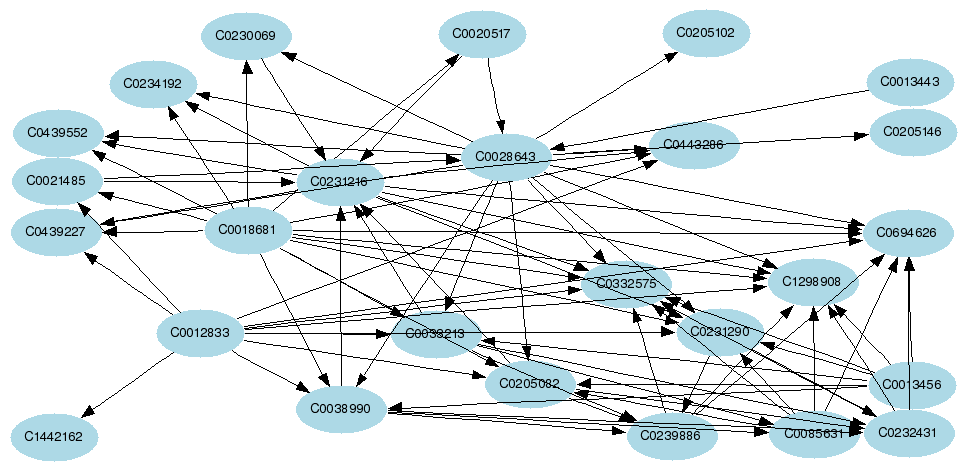

Supplement: Additional file 4 — Source code. Software platform used to launch modules for spell checker [file 1472-6947-7-3-S4.gz › game/graph/99.dot.png]

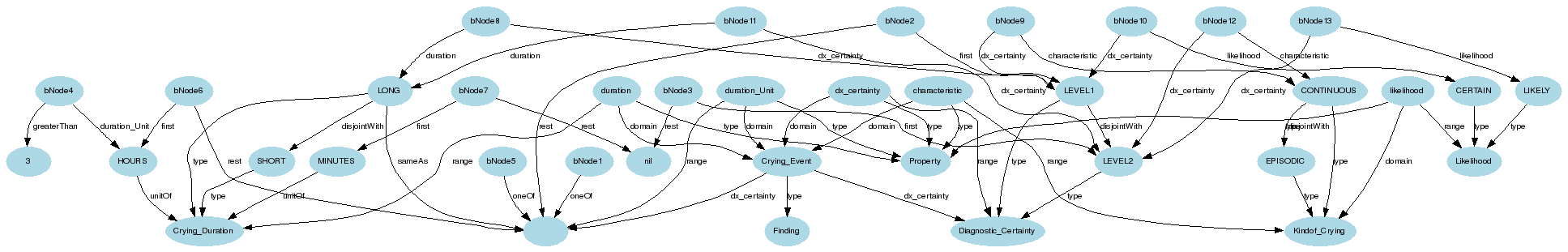

Supplement: Additional file 4 — Source code. Software platform used to launch modules for spell checker [file 1472-6947-7-3-S4.gz › game/graph/crying.dot.png]

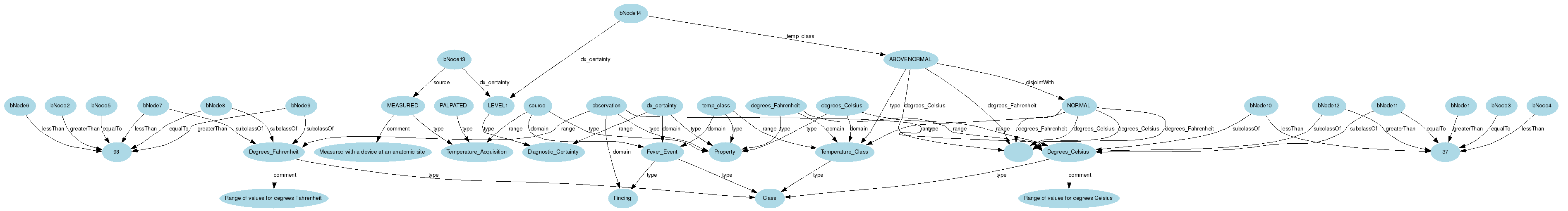

Supplement: Additional file 4 — Source code. Software platform used to launch modules for spell checker [file 1472-6947-7-3-S4.gz › game/graph/fever.dot.png]

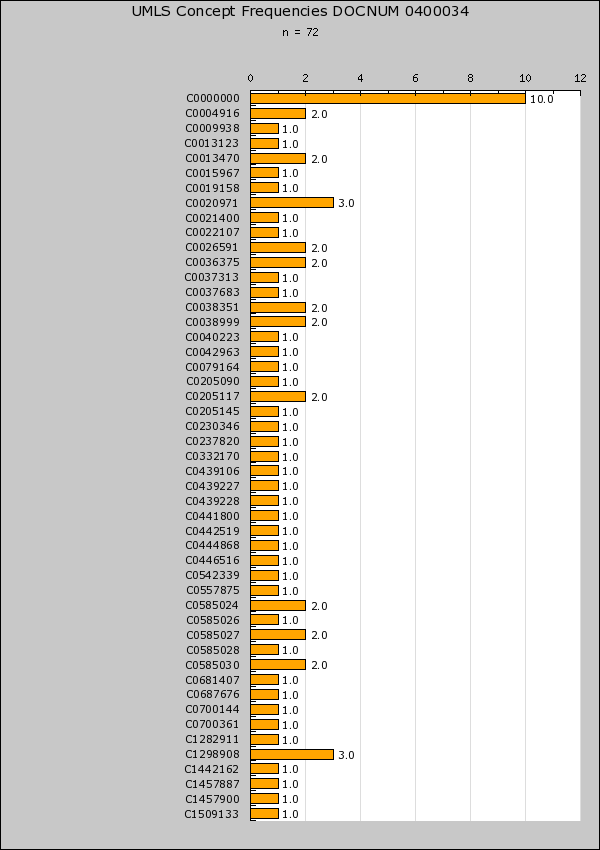

Supplement: Additional file 4 — Source code. Software platform used to launch modules for spell checker [file 1472-6947-7-3-S4.gz › game/graph/HBAR1_CONCEPT_0400034.png]

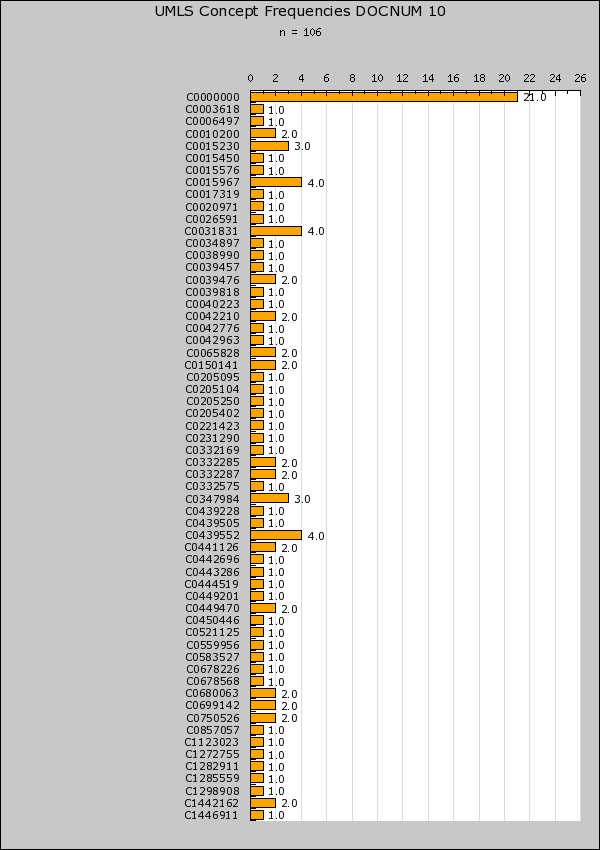

Supplement: Additional file 4 — Source code. Software platform used to launch modules for spell checker [file 1472-6947-7-3-S4.gz › game/graph/HBAR1_CONCEPT_10.png]

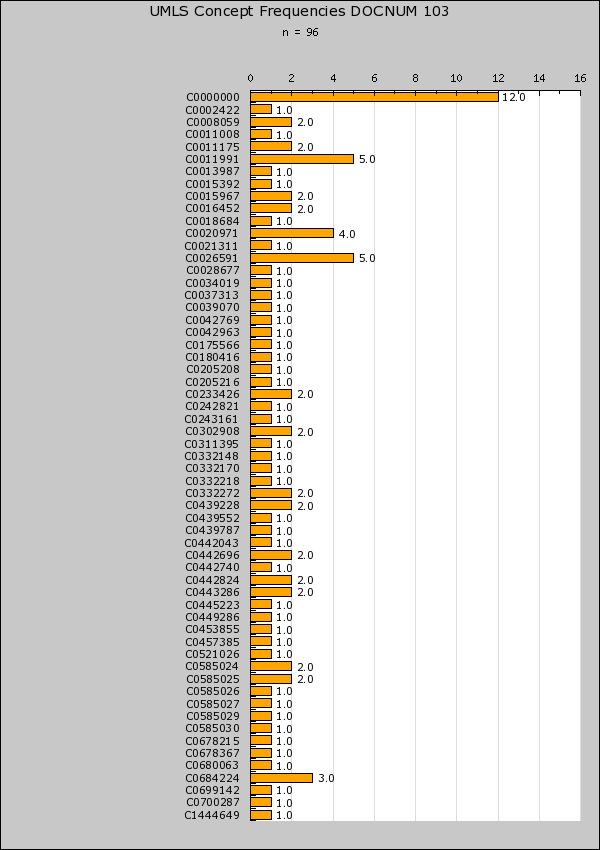

Supplement: Additional file 4 — Source code. Software platform used to launch modules for spell checker [file 1472-6947-7-3-S4.gz › game/graph/HBAR1_CONCEPT_103.png]

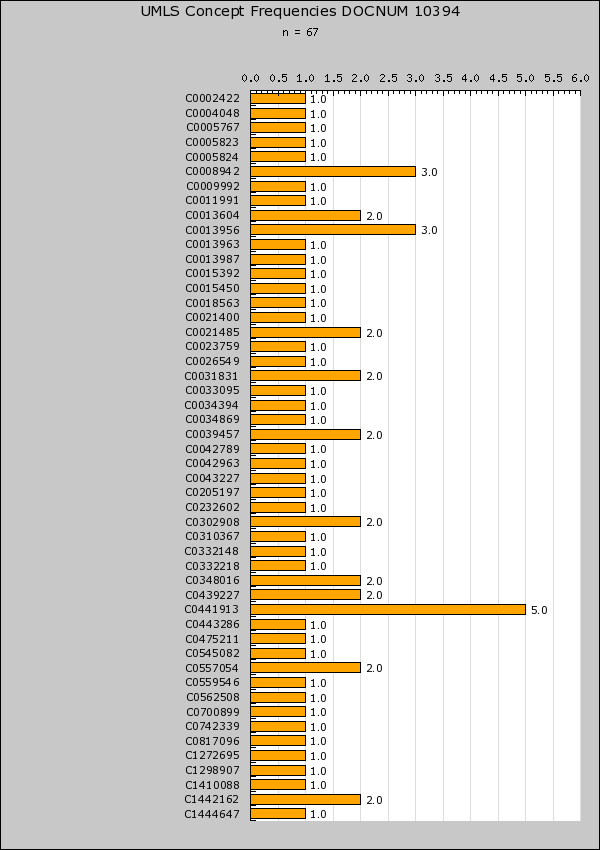

Supplement: Additional file 4 — Source code. Software platform used to launch modules for spell checker [file 1472-6947-7-3-S4.gz › game/graph/HBAR1_CONCEPT_10394.png]

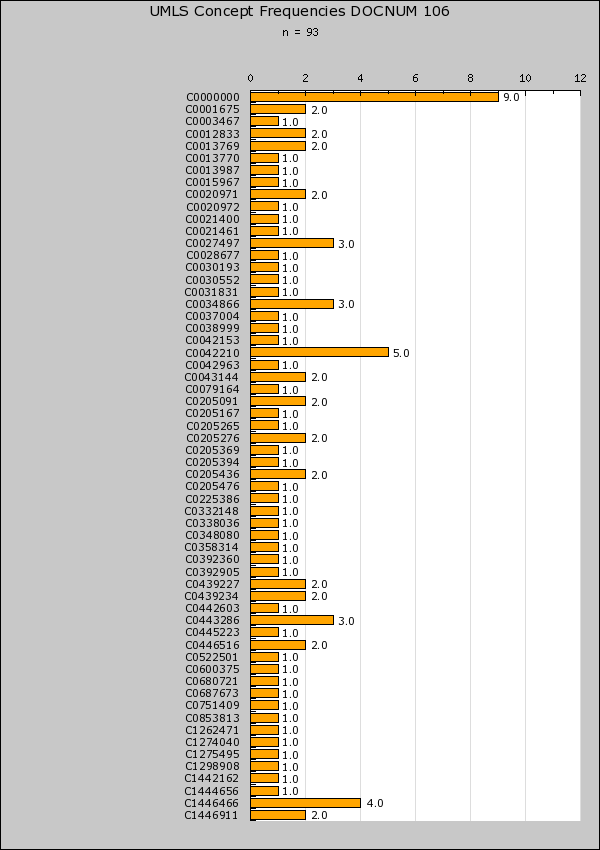

Supplement: Additional file 4 — Source code. Software platform used to launch modules for spell checker [file 1472-6947-7-3-S4.gz › game/graph/HBAR1_CONCEPT_106.png]

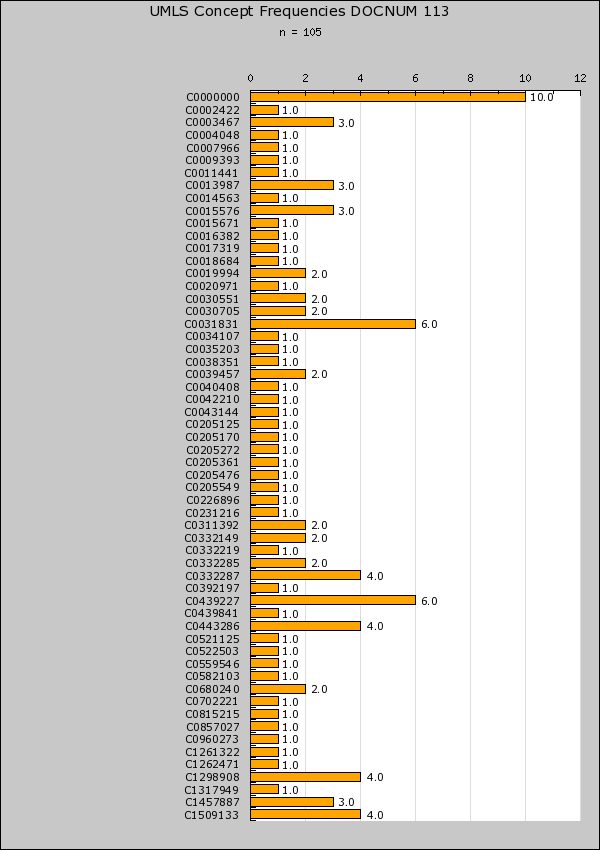

Supplement: Additional file 4 — Source code. Software platform used to launch modules for spell checker [file 1472-6947-7-3-S4.gz › game/graph/HBAR1_CONCEPT_113.png]

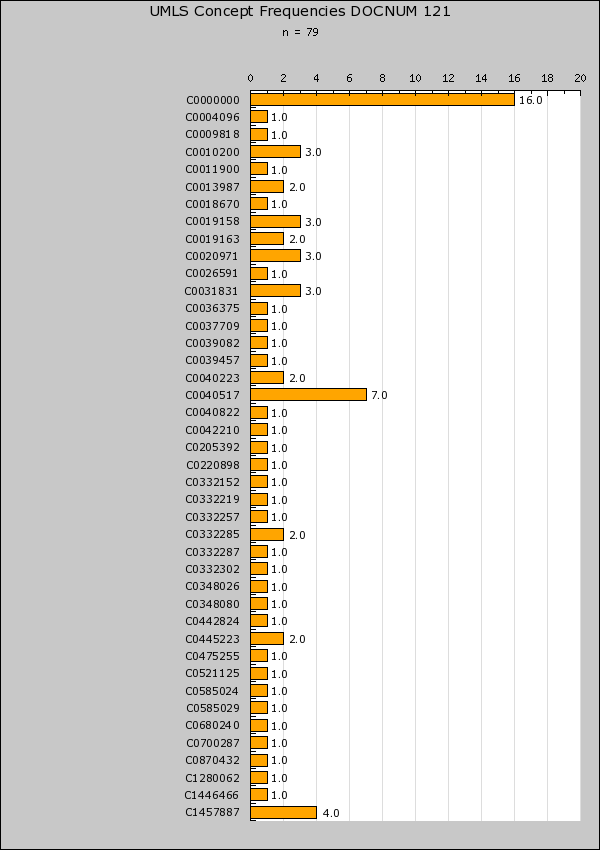

Supplement: Additional file 4 — Source code. Software platform used to launch modules for spell checker [file 1472-6947-7-3-S4.gz › game/graph/HBAR1_CONCEPT_121.png]

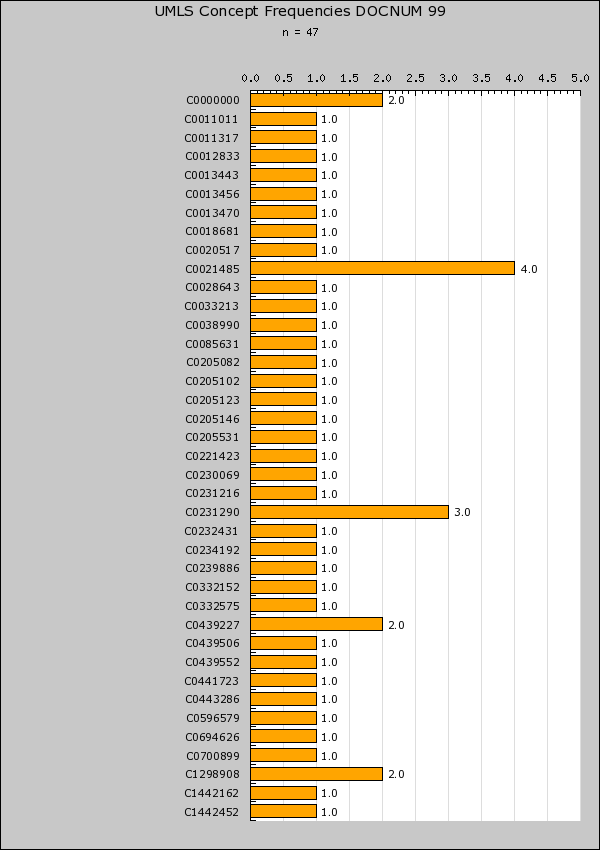

Supplement: Additional file 4 — Source code. Software platform used to launch modules for spell checker [file 1472-6947-7-3-S4.gz › game/graph/HBAR1_CONCEPT_99.png]

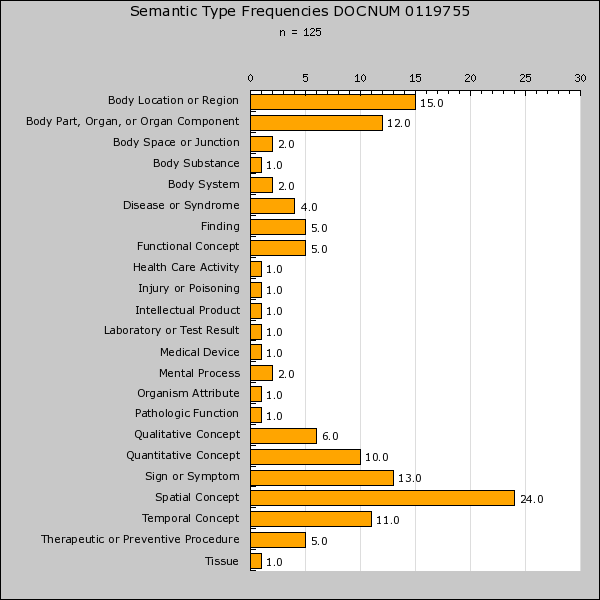

Supplement: Additional file 4 — Source code. Software platform used to launch modules for spell checker [file 1472-6947-7-3-S4.gz › game/graph/HBAR1_STY_0119755.png]

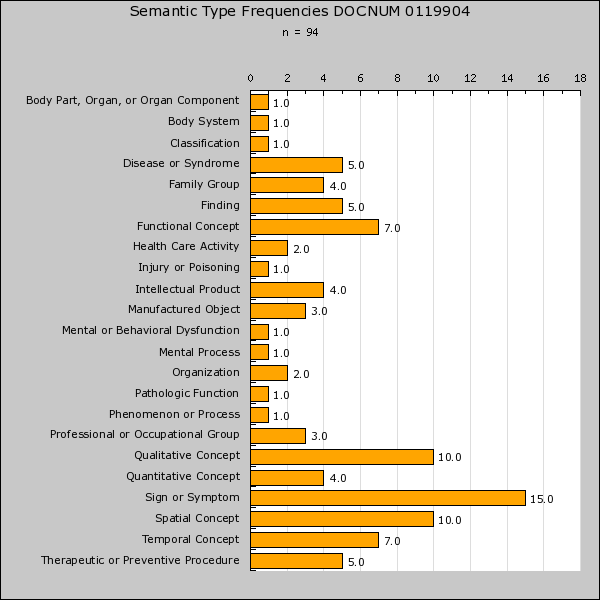

Supplement: Additional file 4 — Source code. Software platform used to launch modules for spell checker [file 1472-6947-7-3-S4.gz › game/graph/HBAR1_STY_0119904.png]

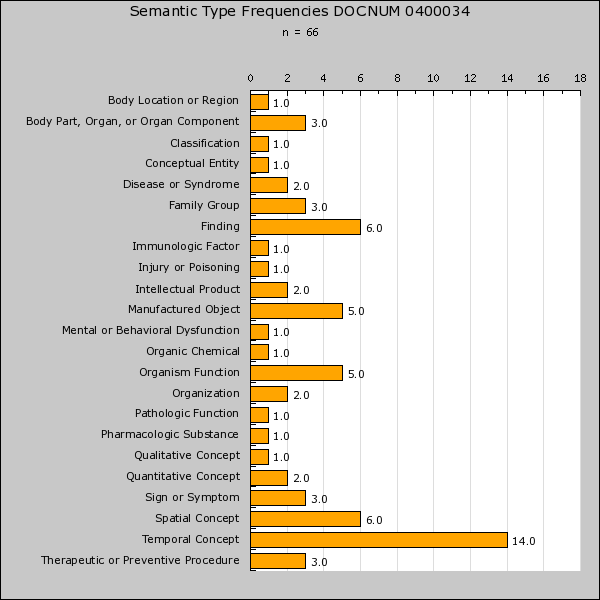

Supplement: Additional file 4 — Source code. Software platform used to launch modules for spell checker [file 1472-6947-7-3-S4.gz › game/graph/HBAR1_STY_0400034.png]

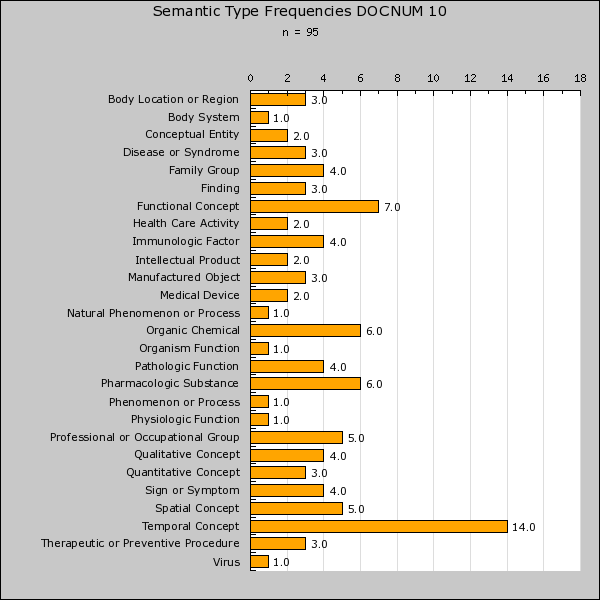

Supplement: Additional file 4 — Source code. Software platform used to launch modules for spell checker [file 1472-6947-7-3-S4.gz › game/graph/HBAR1_STY_10.png]

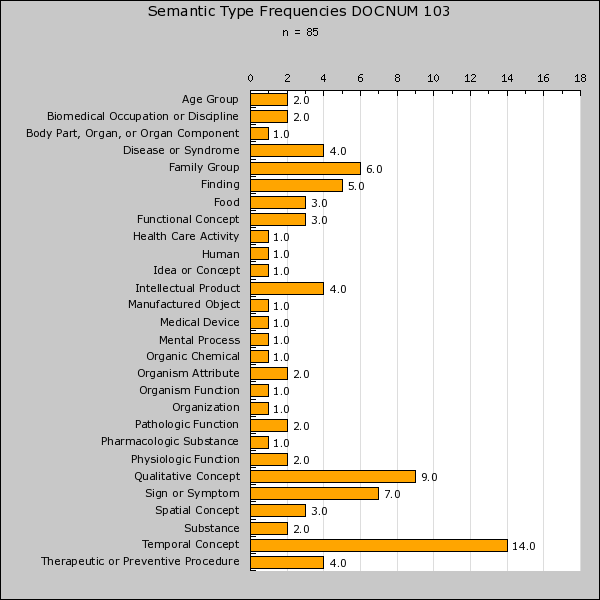

Supplement: Additional file 4 — Source code. Software platform used to launch modules for spell checker [file 1472-6947-7-3-S4.gz › game/graph/HBAR1_STY_103.png]

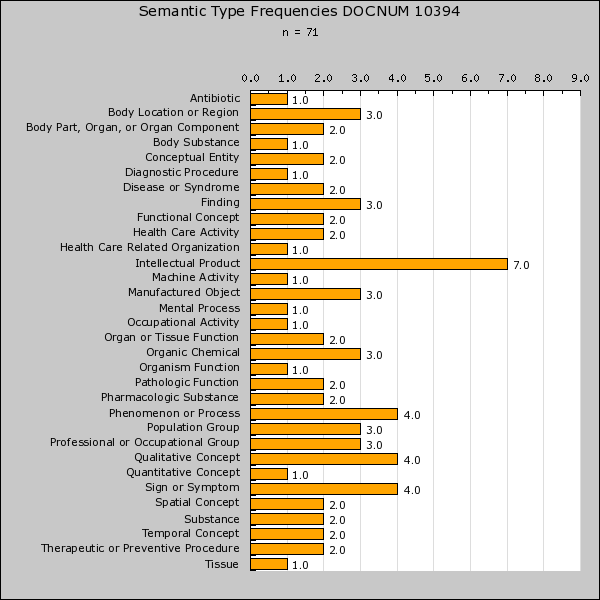

Supplement: Additional file 4 — Source code. Software platform used to launch modules for spell checker [file 1472-6947-7-3-S4.gz › game/graph/HBAR1_STY_10394.png]

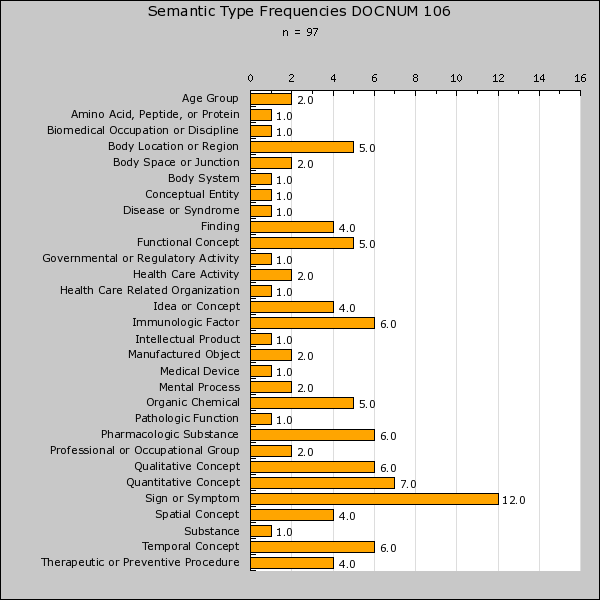

Supplement: Additional file 4 — Source code. Software platform used to launch modules for spell checker [file 1472-6947-7-3-S4.gz › game/graph/HBAR1_STY_106.png]

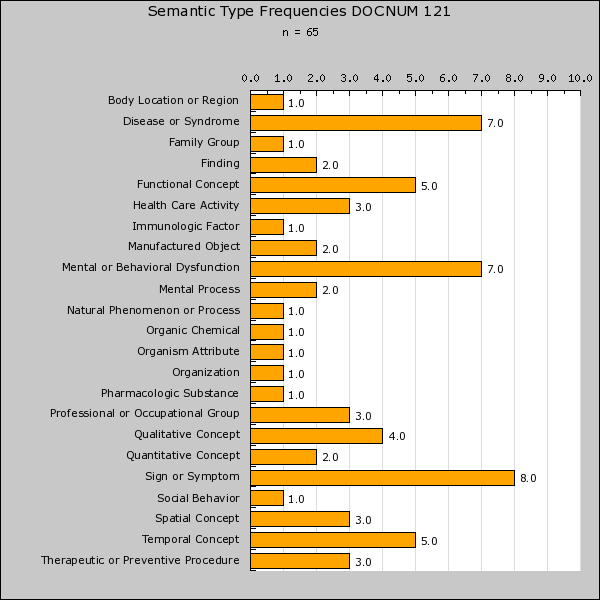

Supplement: Additional file 4 — Source code. Software platform used to launch modules for spell checker [file 1472-6947-7-3-S4.gz › game/graph/HBAR1_STY_121.png]

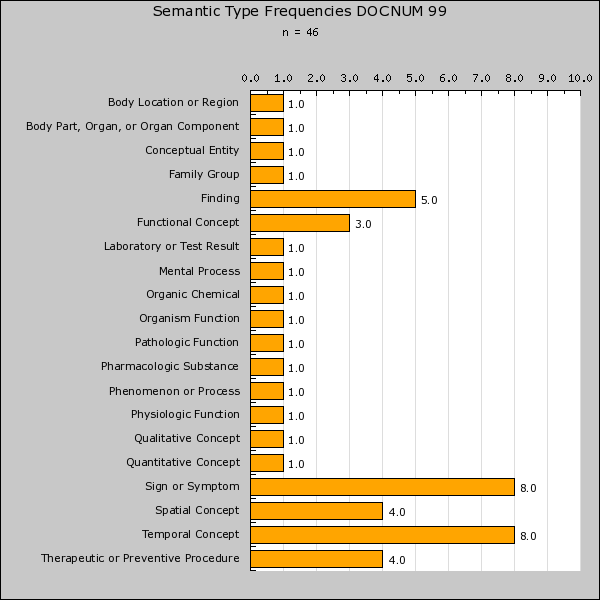

Supplement: Additional file 4 — Source code. Software platform used to launch modules for spell checker [file 1472-6947-7-3-S4.gz › game/graph/HBAR1_STY_99.png]

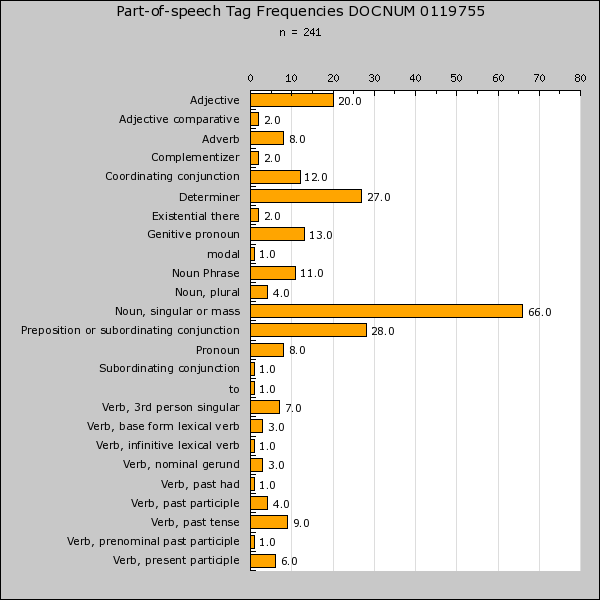

Supplement: Additional file 4 — Source code. Software platform used to launch modules for spell checker [file 1472-6947-7-3-S4.gz › game/graph/HBAR1_TAG_0119755.png]

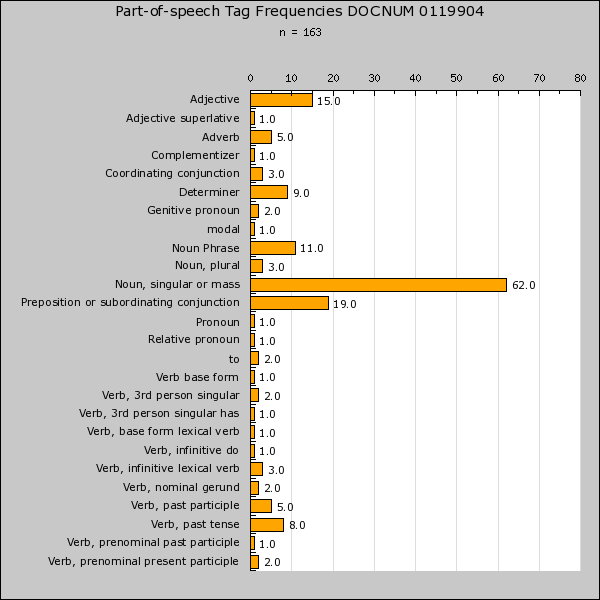

Supplement: Additional file 4 — Source code. Software platform used to launch modules for spell checker [file 1472-6947-7-3-S4.gz › game/graph/HBAR1_TAG_0119904.png]

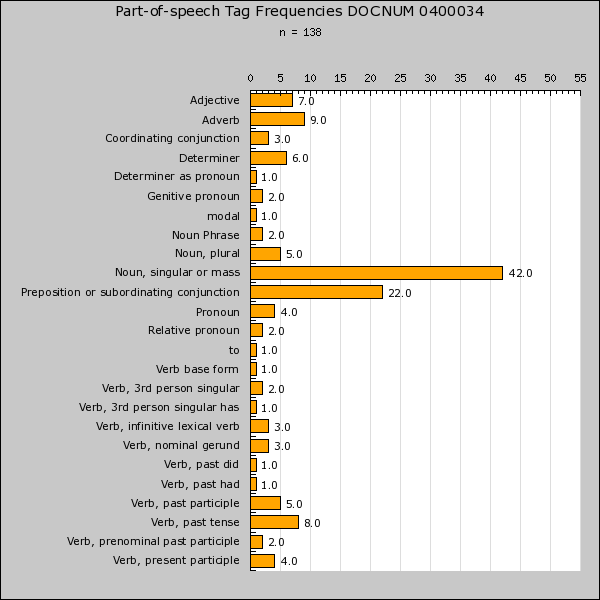

Supplement: Additional file 4 — Source code. Software platform used to launch modules for spell checker [file 1472-6947-7-3-S4.gz › game/graph/HBAR1_TAG_0400034.png]

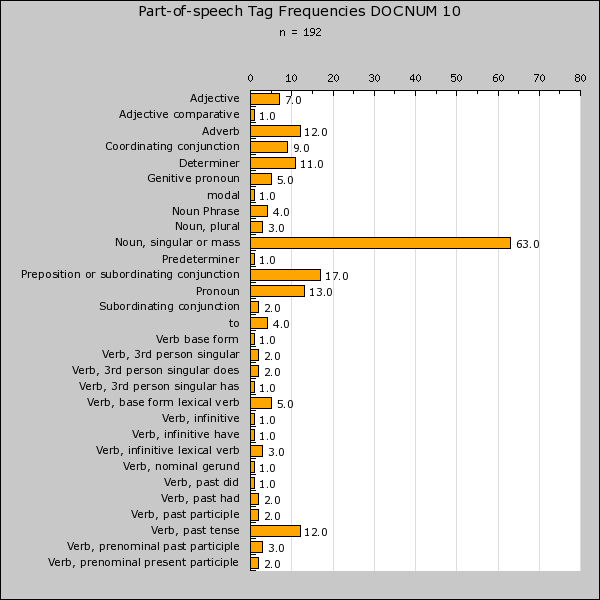

Supplement: Additional file 4 — Source code. Software platform used to launch modules for spell checker [file 1472-6947-7-3-S4.gz › game/graph/HBAR1_TAG_10.png]

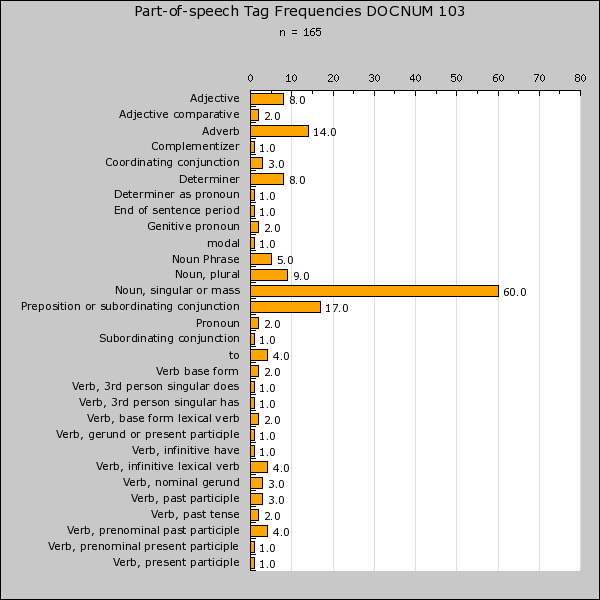

Supplement: Additional file 4 — Source code. Software platform used to launch modules for spell checker [file 1472-6947-7-3-S4.gz › game/graph/HBAR1_TAG_103.png]

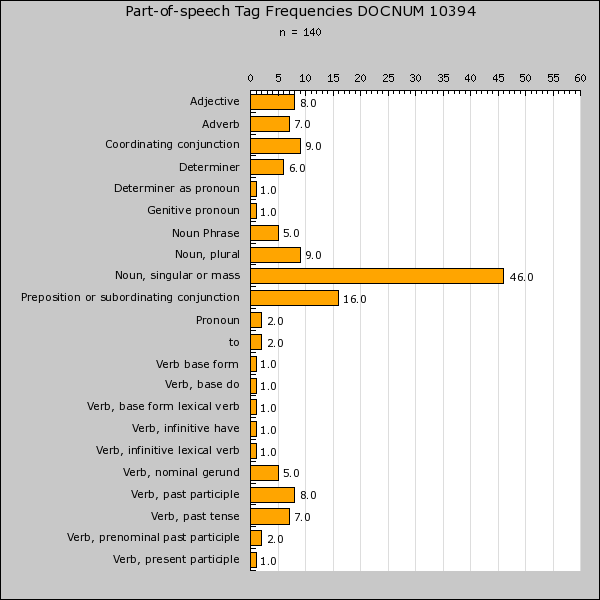

Supplement: Additional file 4 — Source code. Software platform used to launch modules for spell checker [file 1472-6947-7-3-S4.gz › game/graph/HBAR1_TAG_10394.png]

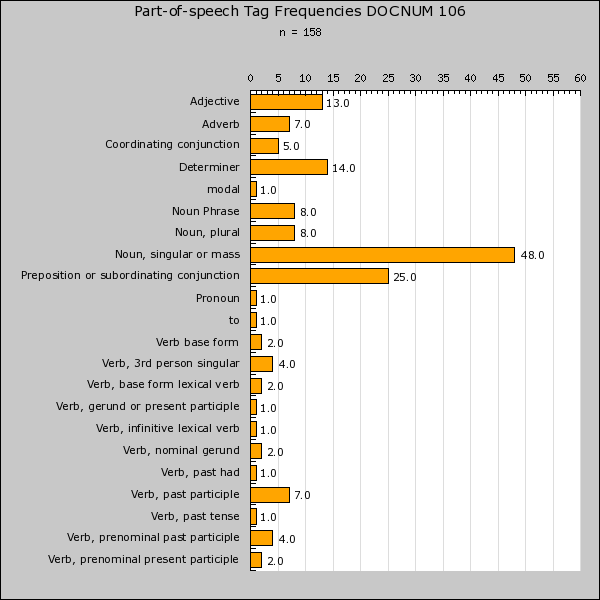

Supplement: Additional file 4 — Source code. Software platform used to launch modules for spell checker [file 1472-6947-7-3-S4.gz › game/graph/HBAR1_TAG_106.png]

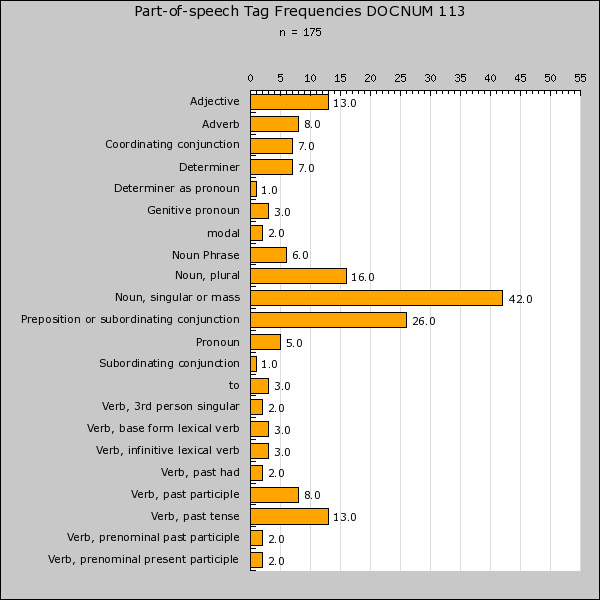

Supplement: Additional file 4 — Source code. Software platform used to launch modules for spell checker [file 1472-6947-7-3-S4.gz › game/graph/HBAR1_TAG_113.png]

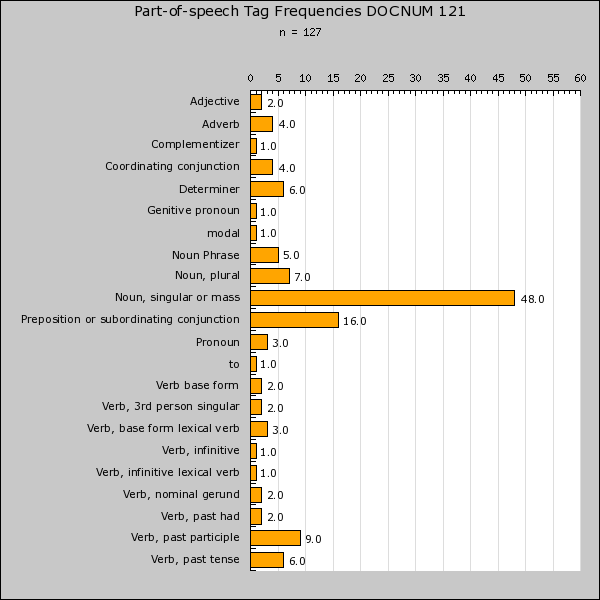

Supplement: Additional file 4 — Source code. Software platform used to launch modules for spell checker [file 1472-6947-7-3-S4.gz › game/graph/HBAR1_TAG_121.png]

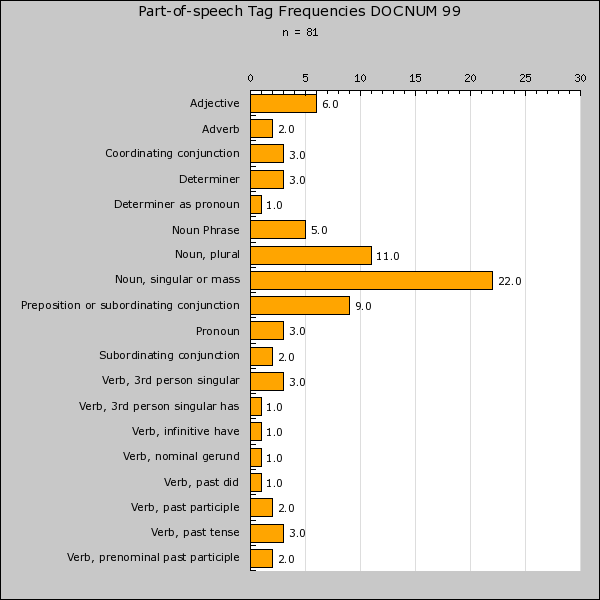

Supplement: Additional file 4 — Source code. Software platform used to launch modules for spell checker [file 1472-6947-7-3-S4.gz › game/graph/HBAR1_TAG_99.png]

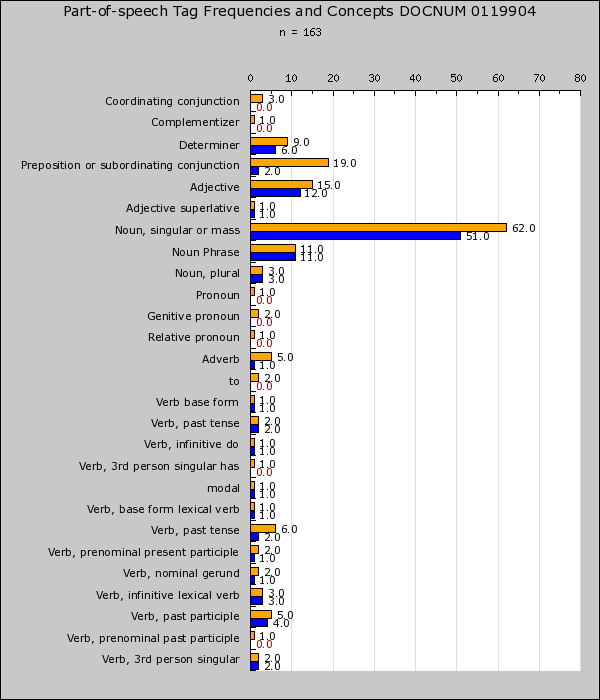

Supplement: Additional file 4 — Source code. Software platform used to launch modules for spell checker [file 1472-6947-7-3-S4.gz › game/graph/HBAR2_TAG_0119904.png]

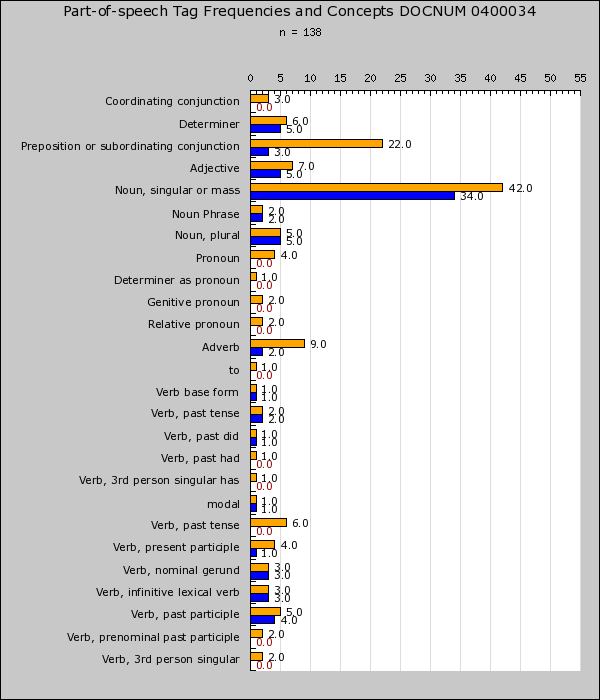

Supplement: Additional file 4 — Source code. Software platform used to launch modules for spell checker [file 1472-6947-7-3-S4.gz › game/graph/HBAR2_TAG_0400034.png]

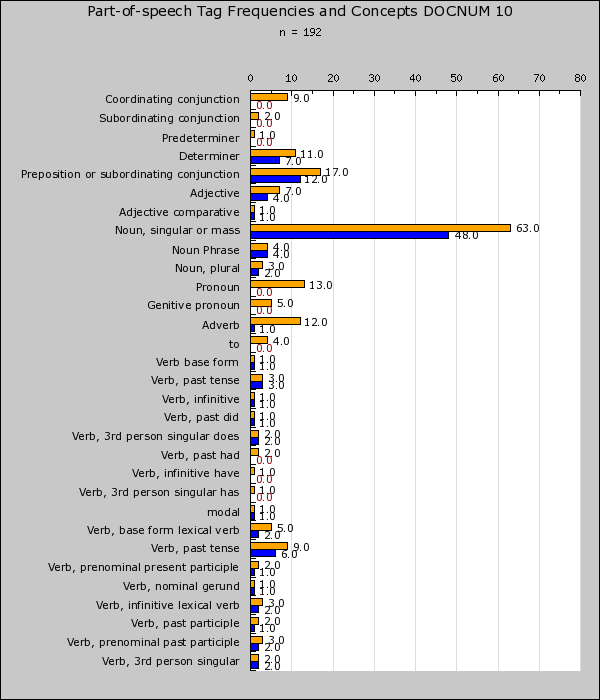

Supplement: Additional file 4 — Source code. Software platform used to launch modules for spell checker [file 1472-6947-7-3-S4.gz › game/graph/HBAR2_TAG_10.png]

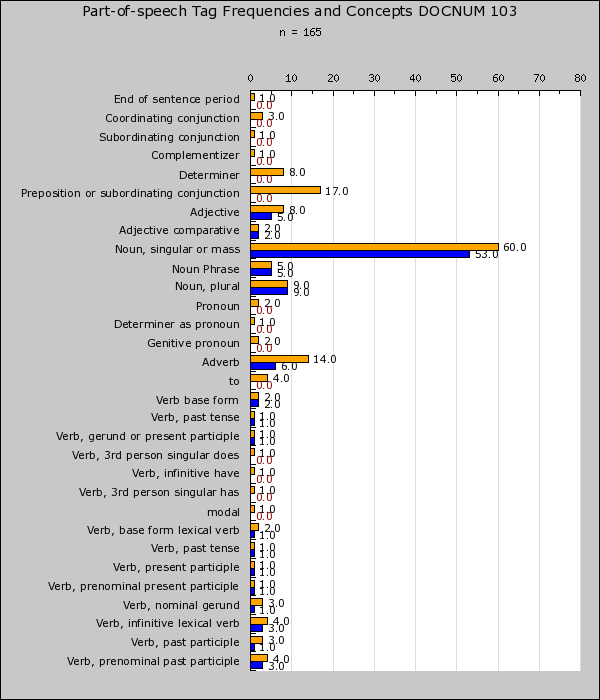

Supplement: Additional file 4 — Source code. Software platform used to launch modules for spell checker [file 1472-6947-7-3-S4.gz › game/graph/HBAR2_TAG_103.png]

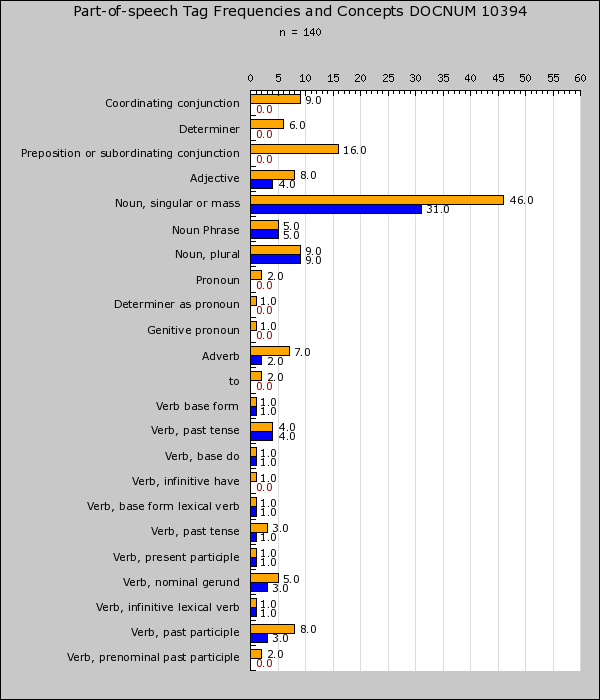

Supplement: Additional file 4 — Source code. Software platform used to launch modules for spell checker [file 1472-6947-7-3-S4.gz › game/graph/HBAR2_TAG_10394.png]

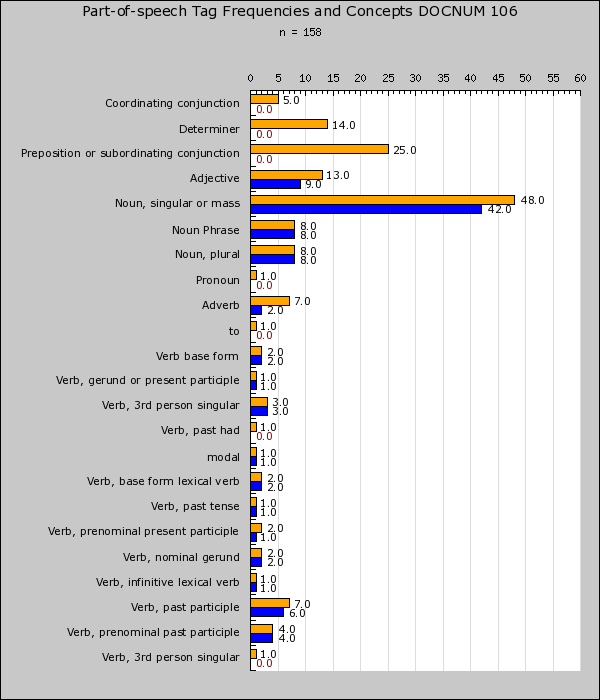

Supplement: Additional file 4 — Source code. Software platform used to launch modules for spell checker [file 1472-6947-7-3-S4.gz › game/graph/HBAR2_TAG_106.png]

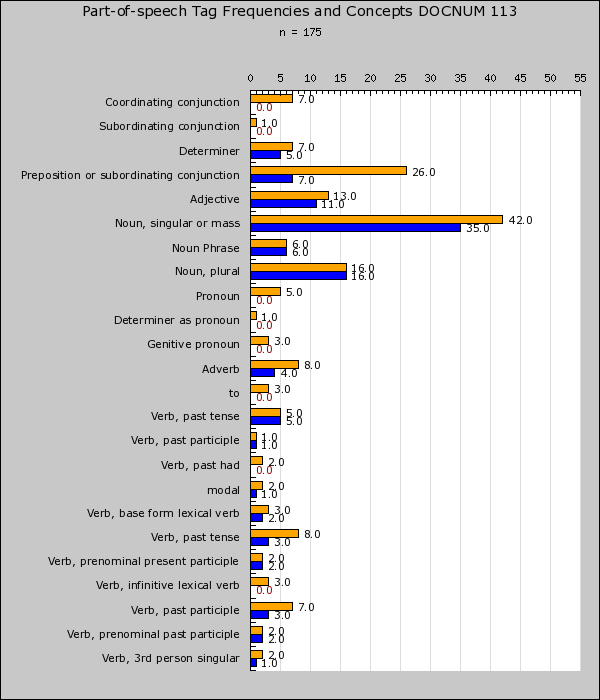

Supplement: Additional file 4 — Source code. Software platform used to launch modules for spell checker [file 1472-6947-7-3-S4.gz › game/graph/HBAR2_TAG_113.png]

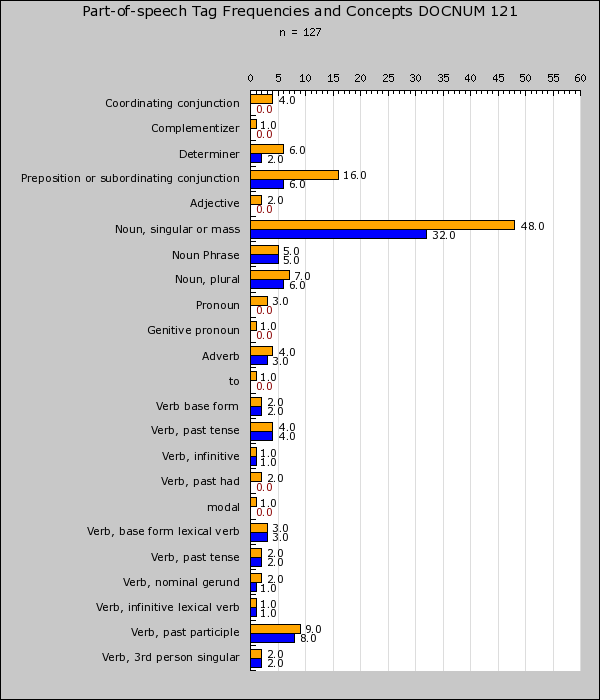

Supplement: Additional file 4 — Source code. Software platform used to launch modules for spell checker [file 1472-6947-7-3-S4.gz › game/graph/HBAR2_TAG_121.png]

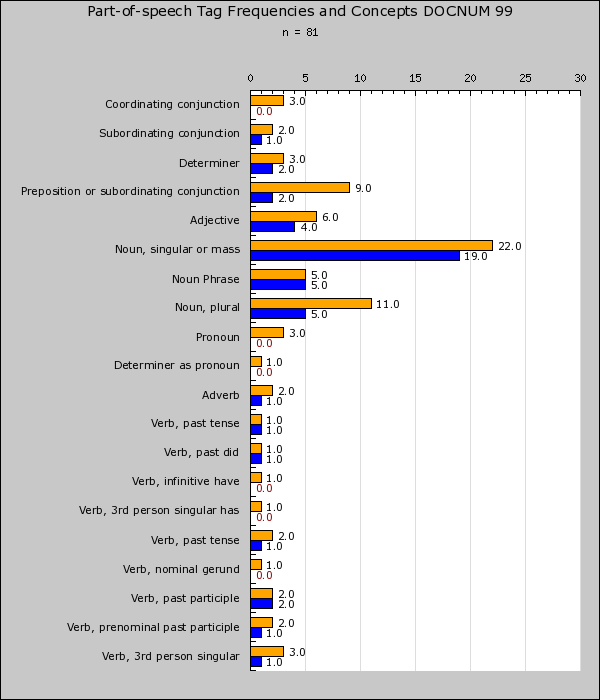

Supplement: Additional file 4 — Source code. Software platform used to launch modules for spell checker [file 1472-6947-7-3-S4.gz › game/graph/HBAR2_TAG_99.png]

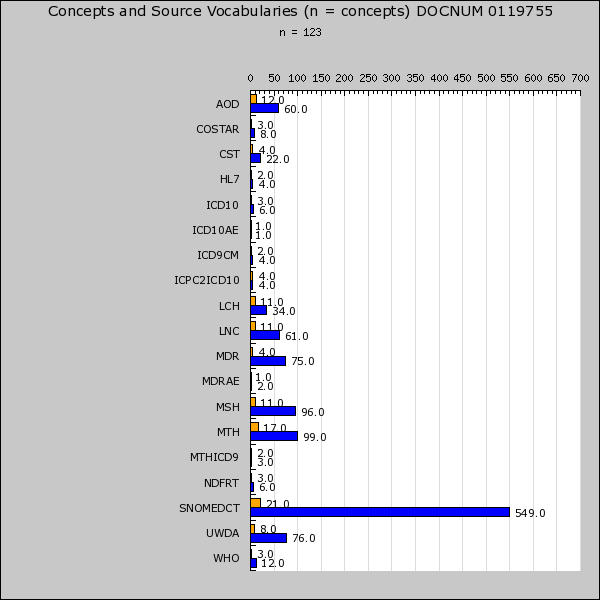

Supplement: Additional file 4 — Source code. Software platform used to launch modules for spell checker [file 1472-6947-7-3-S4.gz › game/graph/HBAR2_VOCABULARY_0119755.png]

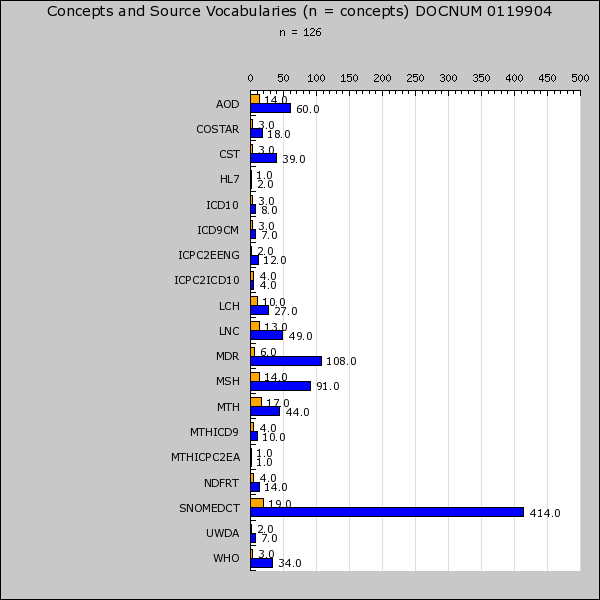

Supplement: Additional file 4 — Source code. Software platform used to launch modules for spell checker [file 1472-6947-7-3-S4.gz › game/graph/HBAR2_VOCABULARY_0119904.png]

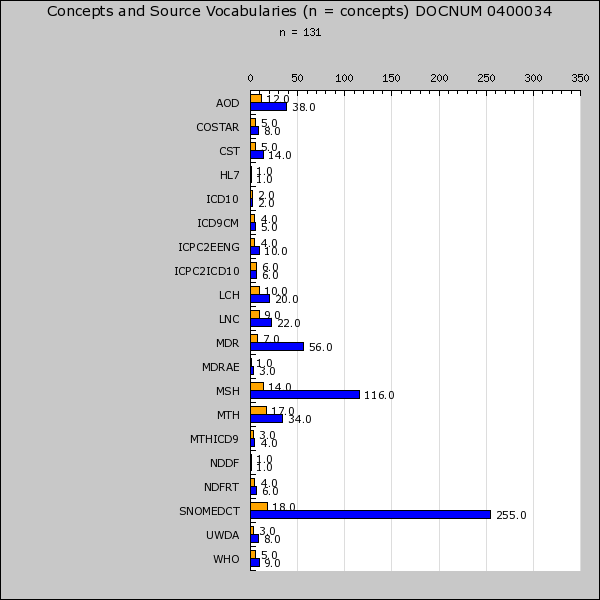

Supplement: Additional file 4 — Source code. Software platform used to launch modules for spell checker [file 1472-6947-7-3-S4.gz › game/graph/HBAR2_VOCABULARY_0400034.png]

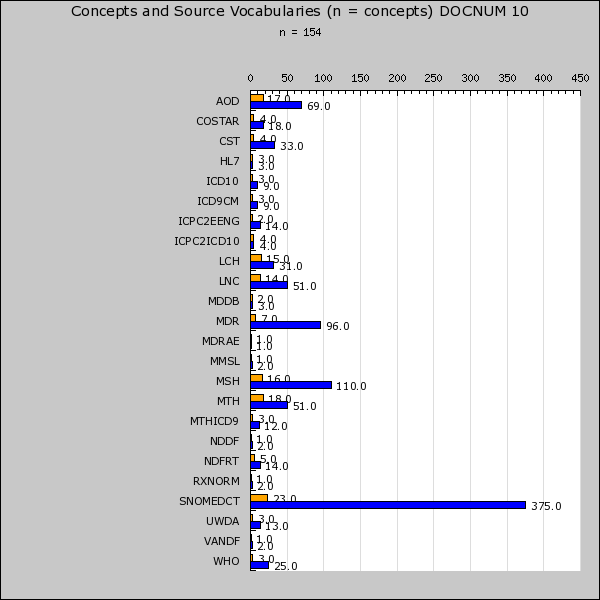

Supplement: Additional file 4 — Source code. Software platform used to launch modules for spell checker [file 1472-6947-7-3-S4.gz › game/graph/HBAR2_VOCABULARY_10.png]

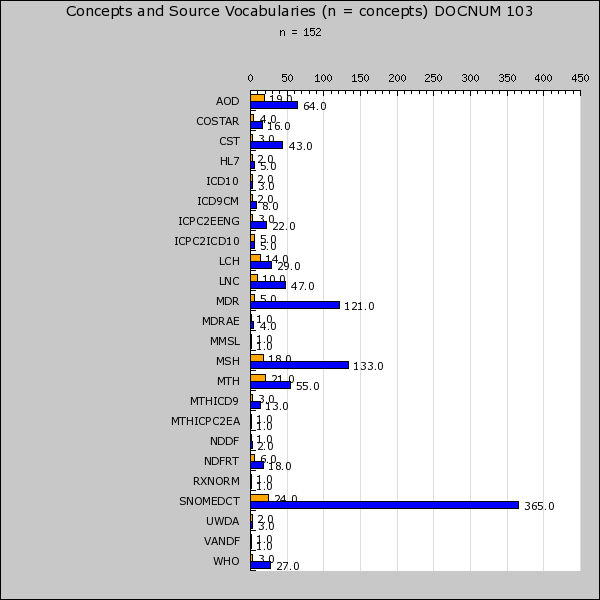

Supplement: Additional file 4 — Source code. Software platform used to launch modules for spell checker [file 1472-6947-7-3-S4.gz › game/graph/HBAR2_VOCABULARY_103.png]

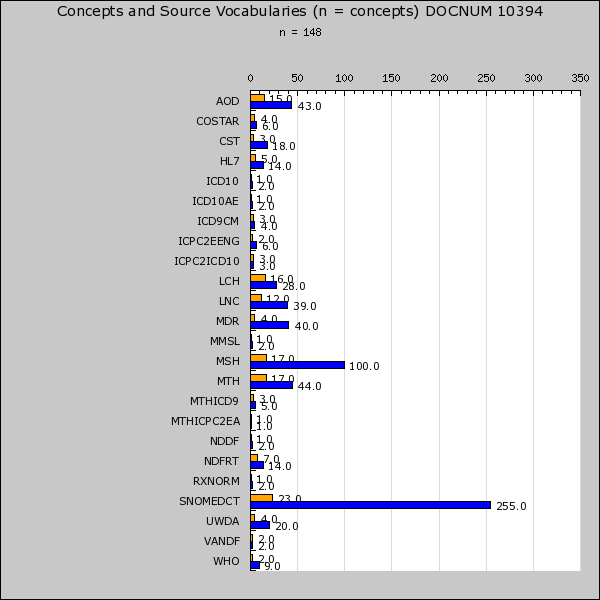

Supplement: Additional file 4 — Source code. Software platform used to launch modules for spell checker [file 1472-6947-7-3-S4.gz › game/graph/HBAR2_VOCABULARY_10394.png]

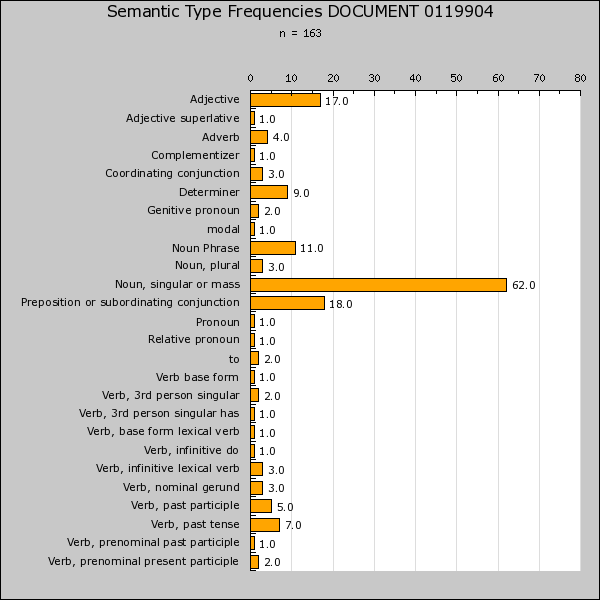

Supplement: Additional file 4 — Source code. Software platform used to launch modules for spell checker [file 1472-6947-7-3-S4.gz › game/graph/HBAR1_0119904.png]

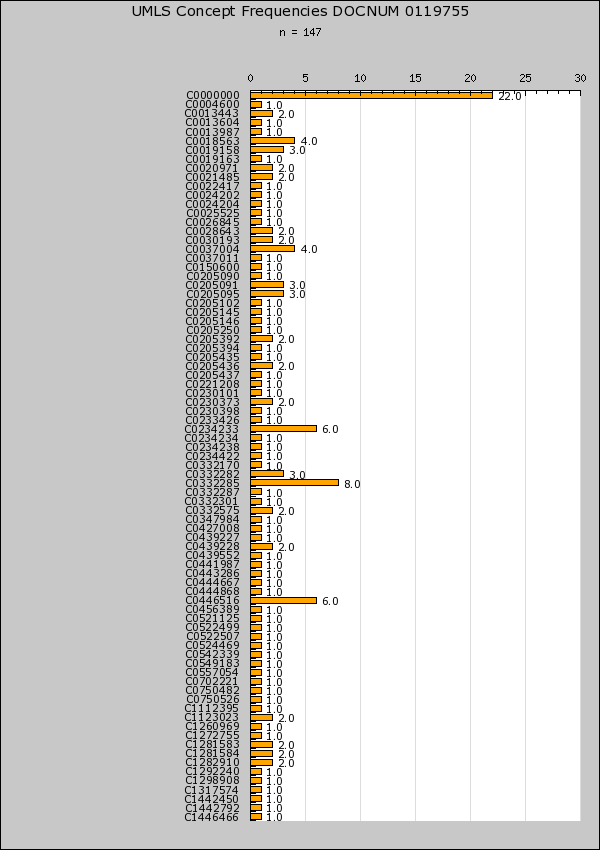

Supplement: Additional file 4 — Source code. Software platform used to launch modules for spell checker [file 1472-6947-7-3-S4.gz › game/graph/HBAR1_CONCEPT_0119755.png]

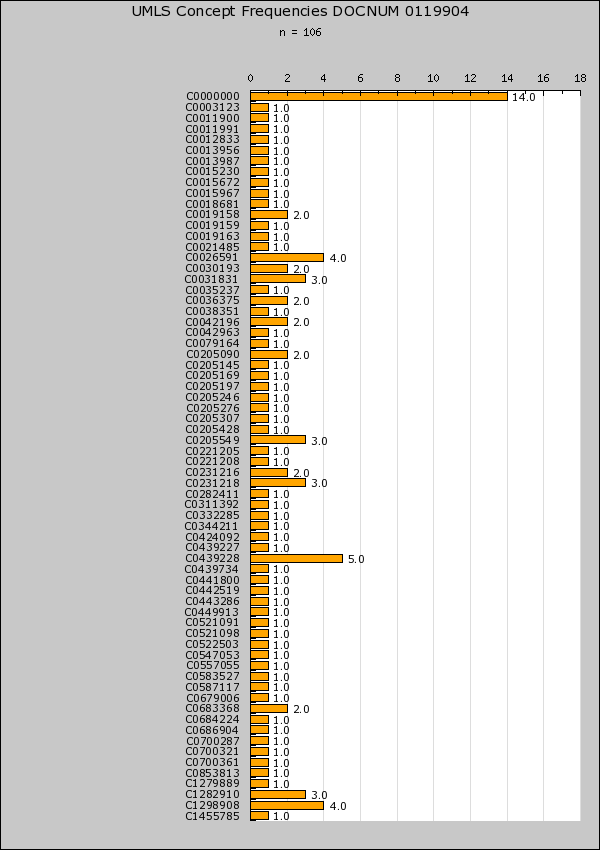

Supplement: Additional file 4 — Source code. Software platform used to launch modules for spell checker [file 1472-6947-7-3-S4.gz › game/graph/HBAR1_CONCEPT_0119904.png]

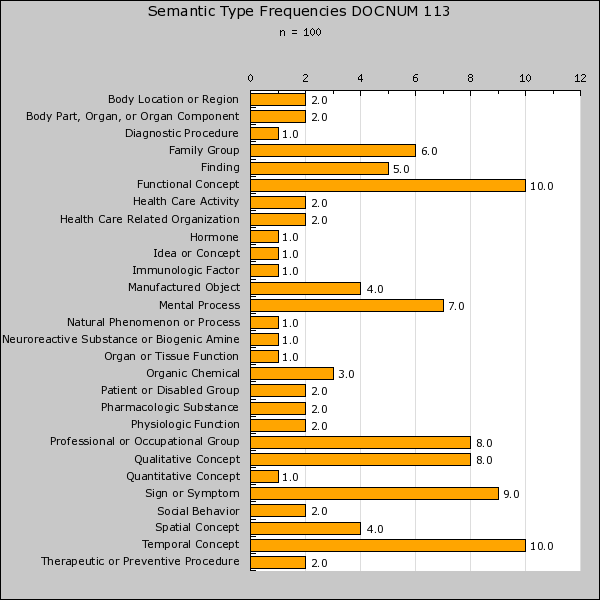

Supplement: Additional file 4 — Source code. Software platform used to launch modules for spell checker [file 1472-6947-7-3-S4.gz › game/graph/HBAR1_STY_113.png]

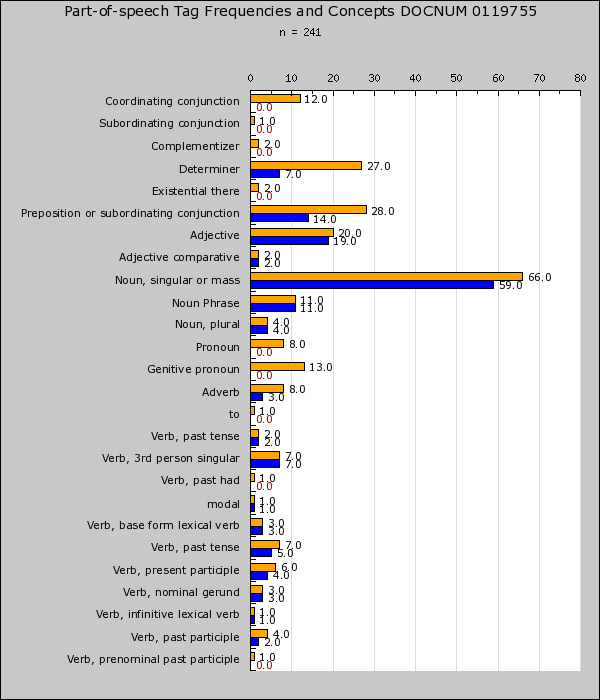

Supplement: Additional file 4 — Source code. Software platform used to launch modules for spell checker [file 1472-6947-7-3-S4.gz › game/graph/HBAR2_TAG_0119755.png]

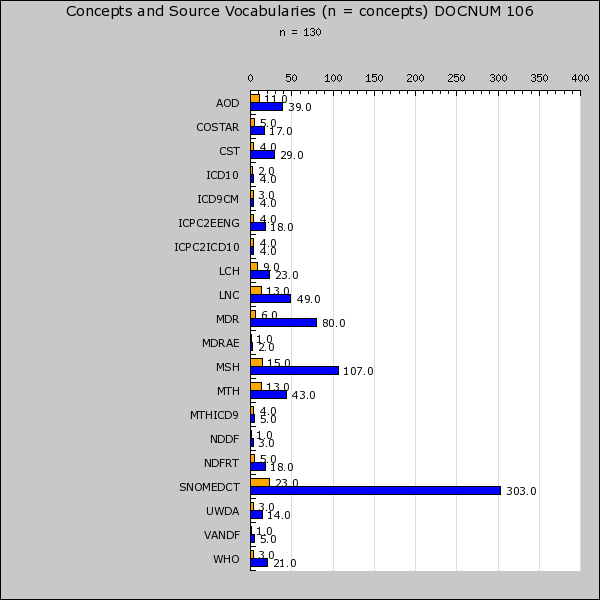

Supplement: Additional file 4 — Source code. Software platform used to launch modules for spell checker [file 1472-6947-7-3-S4.gz › game/graph/HBAR2_VOCABULARY_106.png]

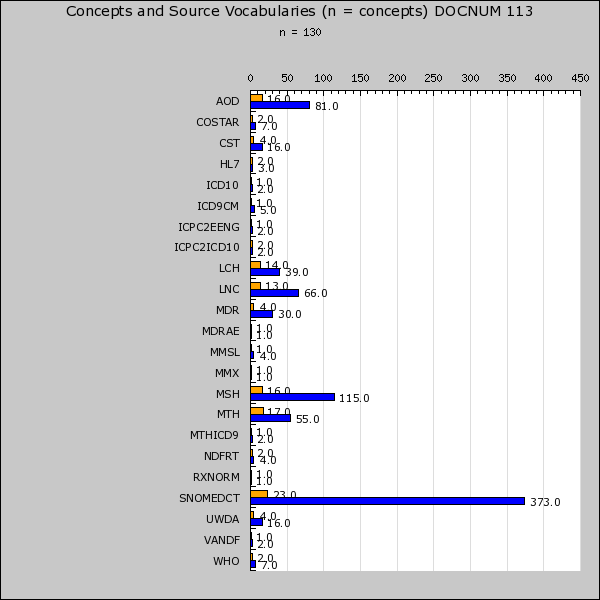

Supplement: Additional file 4 — Source code. Software platform used to launch modules for spell checker [file 1472-6947-7-3-S4.gz › game/graph/HBAR2_VOCABULARY_113.png]

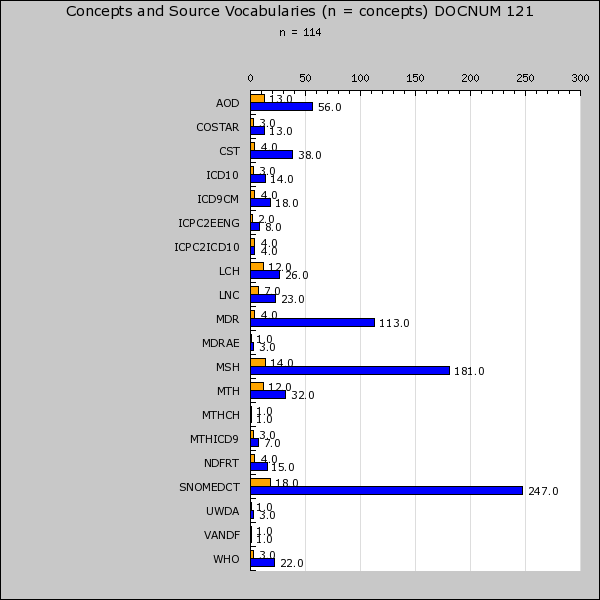

Supplement: Additional file 4 — Source code. Software platform used to launch modules for spell checker [file 1472-6947-7-3-S4.gz › game/graph/HBAR2_VOCABULARY_121.png]

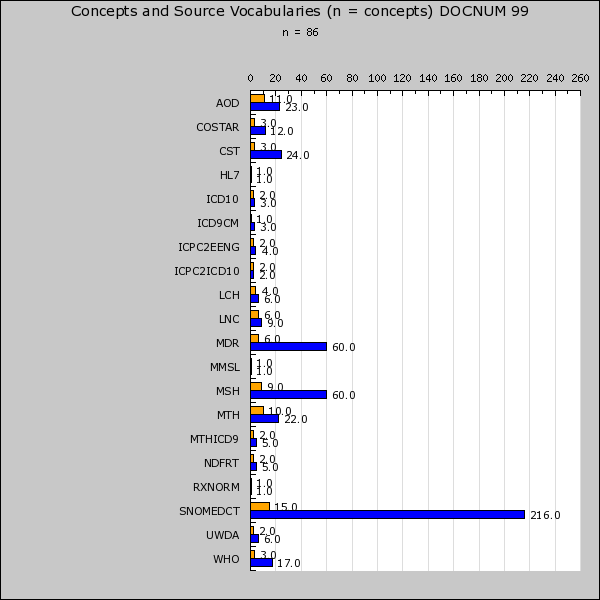

Supplement: Additional file 4 — Source code. Software platform used to launch modules for spell checker [file 1472-6947-7-3-S4.gz › game/graph/HBAR2_VOCABULARY_99.png]

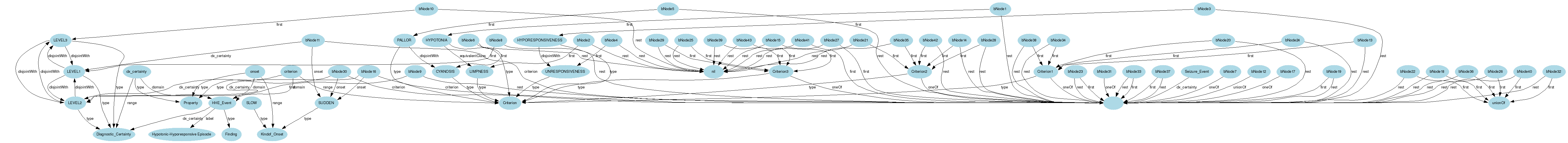

Supplement: Additional file 4 — Source code. Software platform used to launch modules for spell checker [file 1472-6947-7-3-S4.gz › game/graph/hhe.dot.png]

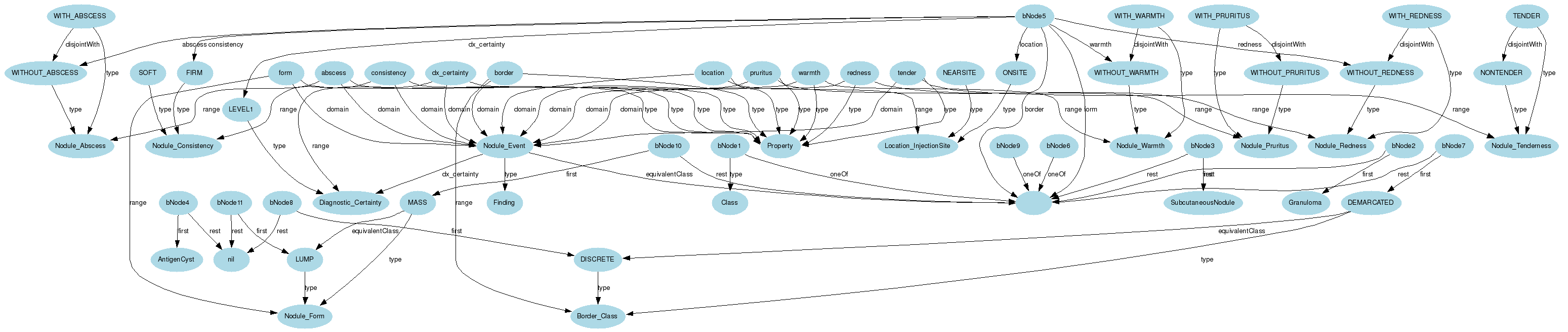

Supplement: Additional file 4 — Source code. Software platform used to launch modules for spell checker [file 1472-6947-7-3-S4.gz › game/graph/nodule.dot.png]

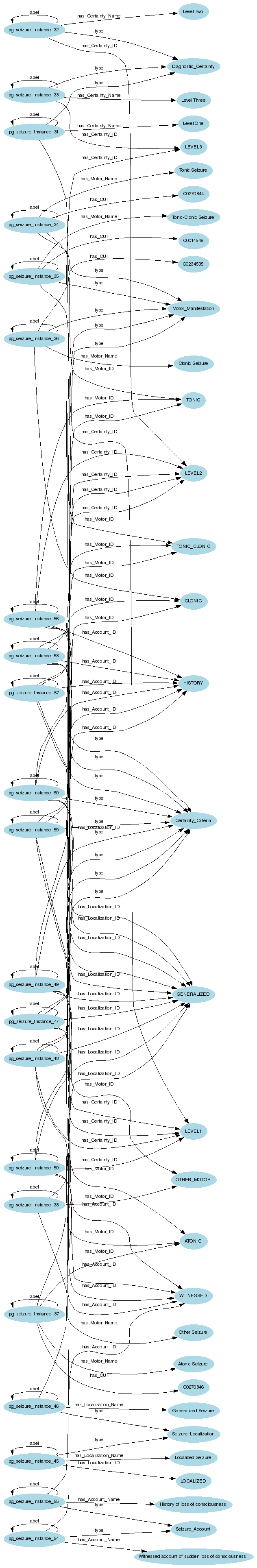

Supplement: Additional file 4 — Source code. Software platform used to launch modules for spell checker [file 1472-6947-7-3-S4.gz › game/graph/pg_seizure.dot.png]

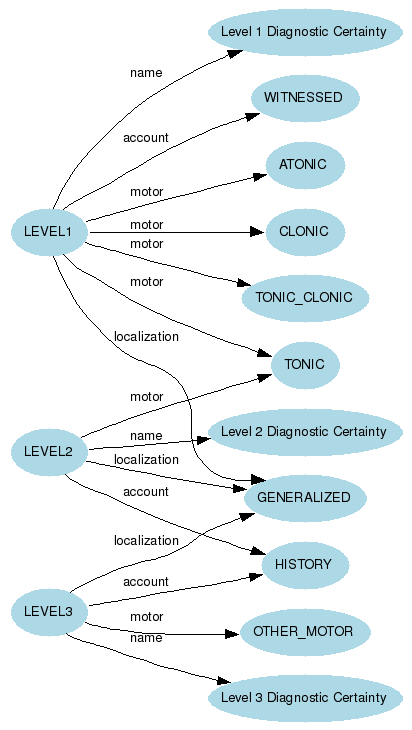

Supplement: Additional file 4 — Source code. Software platform used to launch modules for spell checker [file 1472-6947-7-3-S4.gz › game/graph/pg_seizure2.dot.png]

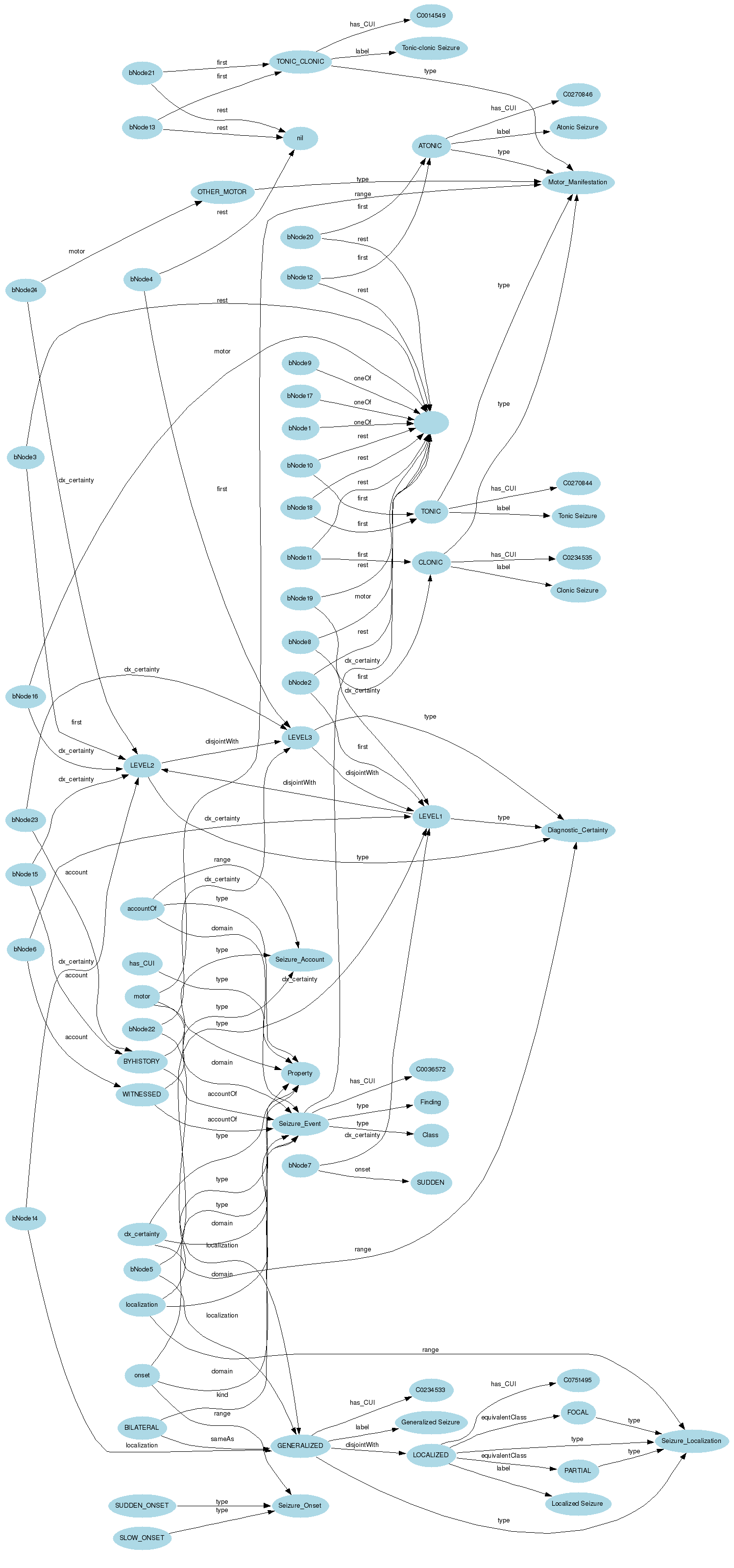

Supplement: Additional file 4 — Source code. Software platform used to launch modules for spell checker [file 1472-6947-7-3-S4.gz › game/graph/seizure.dot.png]

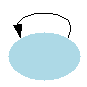

Supplement: Additional file 4 — Source code. Software platform used to launch modules for spell checker [file 1472-6947-7-3-S4.gz › game/graph/seizure2.dot.png]

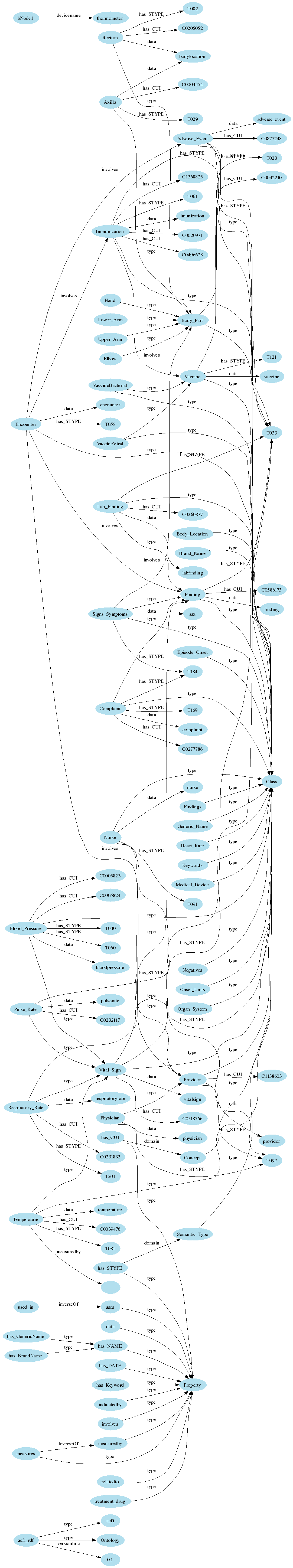

Supplement: Additional file 4 — Source code. Software platform used to launch modules for spell checker [file 1472-6947-7-3-S4.gz › game/graph/test.png]

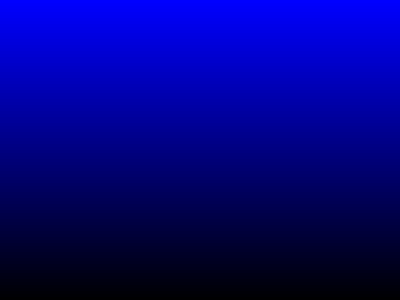

Supplement: Additional file 8 — JPGRAPH application directory. Sets up JPGRAPH directory and source code used to draw graphs for software platform [file 1472-6947-7-3-S8.gz › jpgraph/Examples/blueblack400x300grad.png]

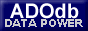

Supplement: Additional file 13 — RAP application directory. Sets up directory for RAP (RDF application for PHP) source code [file 1472-6947-7-3-S13.gz › rap/api/util/adodb/cute_icons_for_site/adodb.gif]

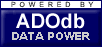

Supplement: Additional file 13 — RAP application directory. Sets up directory for RAP (RDF application for PHP) source code [file 1472-6947-7-3-S13.gz › rap/api/util/adodb/cute_icons_for_site/adodb2.gif]
