# Supplementary material for: A UMLS-based spell checker for natural language processing in vaccine safety
Source: BMC Med Inform Decis Mak. 2007 Feb 12;7:3. doi: 10.1186/1472-6947-7-3 (PMC1805499; doi:10.1186/1472-6947-7-3)
Supplement: Additional file 13 — RAP application directory. Sets up directory for RAP (RDF application for PHP) source code [file 1472-6947-7-3-S13.gz › rap/api/util/adodb/docs/docs-adodb.htm]

ADODB Manual


## ADOdb Library for PHP

V4.50 6 July 2004 (c) 2000-2004 John Lim (jlim#natsoft.com)

This software is dual licensed using BSD-Style and LGPL. This
means you can use it in compiled proprietary and commercial products.

|  |
| --- |
| Kindly note that the ADOdb home page has moved to http://adodb.sourceforge.net/ because of the persistent unreliability of http://php.weblogs.com. **Please change your links**! |

Useful ADOdb links: Download   Other Docs

**Introduction** **Unique Features  
How People are using ADOdb  
Feature Requests and Bug Reports****Installation  
Minimum Install  
Initializing Code and Connectioning to Databases**   Data Source Name (DSN) Support   Connection Examples   
**High Speed ADOdb - tuning tips**  
**Hacking and Modifying ADOdb Safely  
PHP5 Features**  
foreach iterators exceptions  
 **Supported Databases**  
 **Tutorials**  
Example 1: Select  
Example 2: Advanced Select  
Example 3: Insert  
Example 4: Debugging  rs2html
example  
Example 5: MySQL and Menus  
Example 6: Connecting to Multiple Databases at once   
Example 7: Generating Update and Insert SQL   
Example 8: Implementing Scrolling with Next and Previous  
Example 9: Exporting in CSV or Tab-Delimited Format   
Example 10: Custom filters  
Example 11: Smart Transactions  
  
 **Using Custom Error Handlers and PEAR\_Error  
Data Source Names  
Caching  
Pivot Tables**

**REFERENCE**

Variables: $ADODB\_COUNTRECS
$ADODB\_ANSI\_PADDING\_OFF
$ADODB\_CACHE\_DIR   
        $ADODB\_FORCE\_TYPE
$ADODB\_FETCH\_MODE
$ADODB\_LANG   
Constants: ADODB\_ASSOC\_CASE
  
 **ADOConnection**  
Connections: Connect PConnect
NConnect   
Executing SQL: Execute *CacheExecute*
SelectLimit *CacheSelectLimit*
Param Prepare PrepareSP
InParameter OutParameter
  
              GetOne
*CacheGetOne* GetRow *CacheGetRow*
GetAll *CacheGetAll* GetCol
*CacheGetCol* GetAssoc *CacheGetAssoc* Replace
  
               ExecuteCursor
(oci8 only)  
Generates SQL strings: GetUpdateSQL GetInsertSQL
Concat IfNull length random substr
qstr Param
OffsetDate SQLDate
DBDate  DBTimeStamp
  
Blobs: UpdateBlob UpdateClob
UpdateBlobFile BlobEncode
BlobDecode  
Paging/Scrolling: PageExecute CachePageExecute  
Cleanup: CacheFlush Close  
Transactions: StartTrans CompleteTrans
FailTrans HasFailedTrans
BeginTrans CommitTrans
RollbackTrans   
Fetching Data:  SetFetchMode  
Strings: concat length qstr quote substr  
Dates: DBDate DBTimeStamp UnixDate
UnixTimeStamp OffsetDate
SQLDate   
Row Management: Affected\_Rows Insert\_ID RowLock
GenID CreateSequence DropSequence
  
Error Handling: ErrorMsg ErrorNo
MetaError MetaErrorMsg  
Data Dictionary (metadata): MetaDatabases MetaTables
MetaColumns MetaColumnNames
MetaPrimaryKeys MetaForeignKeys
ServerInfo   
Statistics and Query-Rewriting: LogSQL fnExecute
and fnCacheExecute  
Deprecated: Bind BlankRecordSet
Parameter
 **ADORecordSet**  

Returns one field: Fields  
Returns one row:FetchRow FetchInto
FetchObject FetchNextObject
FetchObj FetchNextObj
GetRowAssoc   
Returns all rows:GetArray GetRows
GetAssoc  
Scrolling:Move MoveNext MoveFirst
MoveLast AbsolutePosition CurrentRow
AtFirstPage AtLastPage
AbsolutePage    
Menu generation:GetMenu GetMenu2  
Dates:UserDate UserTimeStamp
UnixDate UnixTimeStamp  
Recordset Info:RecordCount PO\_RecordSet
NextRecordSet  
Field Info:FieldCount FetchField
MetaType  
Cleanup: Close 

**rs2html**  example  
Differences between ADOdb and ADO  
**Database Driver Guide****Change Log**

## Introduction

PHP's database access functions are not standardised. This creates a need for
a database class library to hide the differences between the different database
API's (encapsulate the differences) so we can easily switch databases. PHP 4.0.5 or later
is now required (because we use array-based str\_replace).

We currently support MySQL, Oracle, Microsoft SQL Server, Sybase, Sybase SQL Anywhere, Informix,
PostgreSQL, FrontBase, SQLite, Interbase (Firebird and Borland variants), Foxpro, Access, ADO, DB2, SAP DB and ODBC.
We have had successful reports of connecting to Progress and CacheLite via ODBC. We hope more people
will contribute drivers to support other databases.

PHP4 supports session variables. You can store your session information using
ADOdb for true portability and scalability. See adodb-session.php for more information.

Also read http://php.weblogs.com/portable\_sql
(also available as tips\_portable\_sql.htm in the release) for tips on writing
portable SQL.

## Unique Features of ADOdb

- **Easy for Windows programmers** to adapt to because many of the conventions
  are similar to Microsoft's ADO.
- Unlike other PHP database classes which focus only on select statements,
  **we provide support code to handle inserts and updates which can be adapted
  to multiple databases quickly.** Methods are provided for date handling,
  string concatenation and string quoting characters for differing databases.
- A **metatype system** is built in so that we can figure out that types
  such as CHAR, TEXT and STRING are equivalent in different databases.
- **Easy to port** because all the database dependant code are stored in
  stub functions. You do not need to port the core logic of the classes.
- **Portable table and index creation** with the datadict classes.- **Database performance monitoring and SQL tuning** with the performance monitoring classes.- **Database-backed sessions** with the session management classes. Supports session expiry notification.

## How People are using ADOdb

Here are some examples of how people are using ADOdb (for a much longer list,
visit http://php.weblogs.com/adodb-cool-applications):

- PhpLens is a commercial data grid component that allows both cool Web designers and serious unshaved programmers to develop and maintain databases on the Web easily. Developed by the author of ADOdb.

  - PHAkt: PHP Extension for DreamWeaver Ultradev allows you to script PHP in the popular Web page editor. Database handling provided by ADOdb.

    - Analysis Console for Intrusion Databases (ACID): PHP-based analysis engine to search and process a database of security incidents generated by security-related software such as IDSes and firewalls (e.g. Snort, ipchains). By Roman Danyliw.

      - PostNuke is a very popular free content management
        system and weblog system. It offers full CSS support, HTML 4.01 transitional compliance throughout, an advanced blocks system, and is fully multi-lingual enabled.

        - EasyPublish CMS is another free content management system for managing information and integrated modules on your internet, intranet- and extranet-sites. From Norway.

          - NOLA is a full featured accounting, inventory, and job tracking application. It is licensed under the GPL, and developed by Noguska.

## Feature Requests and Bug Reports

Feature requests and bug reports can be emailed to jlim#natsoft.com.my
or posted to the ADOdb Help forums at http://phplens.com/lens/lensforum/topics.php?id=4.

## Installation Guide

Make sure you are running PHP 4.0.5 or later.
Unpack all the files into a directory accessible by your webserver.

To test, try modifying some of the tutorial examples. Make sure you customize
the connection settings correctly. You can debug using *$db->debug = true* as shown below:

```
<?php
	include('adodb/adodb.inc.php');
	$db = ADONewConnection($dbdriver); # eg 'mysql' or 'postgres'
	$db->debug = true;
	$db->Connect($server, $user, $password, $database);
	$rs = $db->Execute('select * from some_small_table');
	print "<pre>";
	print_r($rs->GetRows());
	print "</pre>";
?>
```

### Minimum Install

For developers who want to release a minimal install of ADOdb, you will need:

- adodb.inc.php- adodb-lib.inc.php- adodb-time.inc.php- drivers/adodb-$database.inc.php- license.txt (for legal reasons)- adodb-php4.inc.php- adodb-iterator.inc.php

Optional:

- adodb-error.inc.php and lang/adodb-$lang.inc.php (if you use MetaError())- adodb-csvlib.inc.php (if you use cached recordsets - CacheExecute(), etc)- adodb-exceptions.inc.php and adodb-errorhandler.inc.php (if you use adodb error handler or php5 exceptions).

### Code Initialization Examples

When running ADOdb, at least two files are loaded. First is adodb/adodb.inc.php,
which contains all functions used by all database classes. The code specific
to a particular database is in the adodb/driver/adodb-????.inc.php file.

For example, to connect to a mysql database:

```
include('/path/to/set/here/adodb.inc.php');
$conn = &ADONewConnection('mysql');
```

Whenever you need to connect to a database, you create a Connection object
using the **ADONewConnection**($driver) function.
**NewADOConnection**($driver) is an alternative name for the same function.

At this point, you are not connected to the database (no longer true if you pass in a dsn). You will first need to decide
whether to use *persistent* or *non-persistent* connections. The advantage of *persistent*
connections is that they are faster, as the database connection is never closed (even
when you call Close()). *Non-persistent* connections take up much fewer resources though,
reducing the risk of your database and your web-server becoming overloaded.

For persistent connections,
use $conn->PConnect(),
or $conn->Connect() for non-persistent connections.
Some database drivers also support NConnect(), which forces
the creation of a new connection.

**Connection Gotcha**: If you create two connections, but both use the same userid and password,
PHP will share the same connection. This can cause problems if the connections are meant to
different databases. The solution is to always use different userid's for different databases,
or use NConnect().

### Data Source Name (DSN) Support

Since ADOdb 4.51, you can connect to a database by passing a dsn to NewADOConnection() (or ADONewConnection, which is
the same function). The dsn format is:

```
	$driver://$username:$password@hostname/$database?options[=value]
```

NewADOConnection() calls Connect() or PConnect() internally for you. If the connection fails, false is returned.

```
	# non-persistent connection
	$dsn = 'mysql://root:pwd@localhost/mydb'; 
	$db = NewADOConnection($dsn);
	if (!$db) die("Connection failed");   
	
	# no need to call connect/pconnect!
	$arr = $db->GetArray("select * from table");
	
	# persistent connection
	$dsn2 = 'mysql://root:pwd@localhost/mydb?persist';
```

If you have special characters such as /:? in your dsn, then you need to rawurlencode them first:

```
	$pwd = rawurlencode($pwd);
	$dsn = "mysql://root:$pwd@localhost/mydb";
```

Legal options are:

|  |  |  |  |  |  |  |  |  |  |
| --- | --- | --- | --- | --- | --- | --- | --- | --- | --- |
| For all drivers 'persist', 'persistent', 'debug', 'fetchmode'|  |  |  |  |  |  |  |  | | --- | --- | --- | --- | --- | --- | --- | --- | | Interbase/Firebird 'dialect', 'charset', 'buffers'|  |  |  |  |  |  | | --- | --- | --- | --- | --- | --- | | M'soft ADO 'charpage'|  |  |  |  | | --- | --- | --- | --- | | MySQL 'clientflags'|  |  | | --- | --- | | MySQLi 'port', 'socket', 'clientflags' | | | | | | | | | |

For all drivers, when the options *persist* or *persistent* are set, a persistent connection is forced.
The *debug* option enables debugging. The *fetchmode* calls SetFetchMode().
If no value is defined for an option, then the value is set to 1.

ADOdb DSN's are compatible with version 1.0 of PEAR DB's DSN format.

### Examples of Connecting to Databases

#### MySQL and Most Other Database Drivers

MySQL connections are very straightforward, and the parameters are identical
to mysql\_connect:

```
	$conn = &ADONewConnection('mysql'); 
	$conn->PConnect('localhost','userid','password','database');
	
	# or dsn 
	$dsn = 'mysql://user:pwd@localhost/mydb'; 
	$conn = ADONewConnection($dsn);  # no need for Connect()
	
	# or persistent dsn
	$dsn = 'mysql://user:pwd@localhost/mydb?persist'; 
	$conn = ADONewConnection($dsn);  # no need for PConnect()
	
	# a more complex example:
	$pwd = urlencode($pwd);
	$flags =  MYSQL_CLIENT_COMPRESS;
	$dsn = "mysql://user:$pwd@localhost/mydb?persist&clientflags=$flags";
	$conn = ADONewConnection($dsn);  # no need for PConnect()
```

For most drivers, you can use the standard function: Connect($server, $user, $password, $database), or
a DSN since ADOdb 4.51. Exceptions to this are listed below.

#### PostgreSQL

PostgreSQL accepts connections using:

a. the standard connection string:

```
	$conn = &ADONewConnection('postgres7'); 
	$conn->PConnect('host=localhost port=5432 dbname=mary');
```

b. the classical 4 parameters:

```
	$conn->PConnect('localhost','userid','password','database');
```

c. dsn:

```
	$dsn = 'postgres7://user:pwd@localhost/mydb?persist';  # persist is optional
	$conn = ADONewConnection($dsn);  # no need for Connect/PConnect
```

#### LDAP

Here is an example of querying a LDAP server. Thanks to Josh Eldridge for the driver and this example:

```
 <?php
require('/path/to/adodb.inc.php');

$host = 'ldap.baylor.edu';
$ldapbase = 'ou=People,o=Baylor University,c=US';

$ldap = NewADOConnection( 'ldap' );
$ldap->Connect( $host, $user_name='', $password='', $ldapbase );

echo "<pre>";

print_r( $ldap->ServerInfo() );
$ldap->SetFetchMode(ADODB_FETCH_ASSOC);
$userName = 'eldridge';
$filter="(|(CN=$userName*)(sn=$userName*)(givenname=$userName*)(uid=$userName*))";

$rs = $ldap->Execute( $filter );
if ($rs)
	while ($arr = $rs->FetchRow()) {
	     print_r($arr);	
	}

$rs = $ldap->Execute( $filter );
if ($rs) 
	while (!$rs->EOF) {
 		print_r($rs->fields);	
		$rs->MoveNext();
	} 
	
print_r( $ldap->GetArray( $filter ) );

print_r( $ldap->GetRow( $filter ) );

$ldap->Close();
echo "</pre>";
?>
```

#### Interbase/Firebird

You define the database in the $host parameter:

```
	$conn = &ADONewConnection('ibase'); 
	$conn->PConnect('localhost:c:\ibase\employee.gdb','sysdba','masterkey');
```

Or dsn:

```
	$dsn = 'firebird://user:pwd@localhost/mydb?persist&dialect=3';  # persist is optional
	$conn = ADONewConnection($dsn);  # no need for Connect/PConnect
```

#### SQLite

Sqlite will create the database file if it does not exist.

```
	$conn = &ADONewConnection('sqlite'); 
	$conn->PConnect('c:\path\to\sqlite.db'); # sqlite will create if does not exist
```

Or dsn:

```
	$dsn = 'sqlite://user:pwd@localhost/mydb?persist';  # persist is optional
	$conn = ADONewConnection($dsn);  # no need for Connect/PConnect
```

#### Oracle (oci8)

With oci8, you can connect in multiple ways. Note that oci8 works fine with
newer versions of the Oracle, eg. 9i and 10g.

a. PHP and Oracle reside on the same machine, use default SID.

```
	$conn->Connect(false, 'scott', 'tiger');
```

b. TNS Name defined in tnsnames.ora (or ONAMES or HOSTNAMES), eg. 'myTNS'

```
	$conn->PConnect(false, 'scott', 'tiger', 'myTNS');
```

or

```
 	$conn->PConnect('myTNS', 'scott', 'tiger');
```

c. Host Address and SID

```
	$conn->Connect('192.168.0.1', 'scott', 'tiger', 'SID');
```

d. Host Address and Service Name

```
	$conn->Connect('192.168.0.1', 'scott', 'tiger', 'servicename');
```

e. Oracle connection string:

```
	$cstr = "(DESCRIPTION=(ADDRESS=(PROTOCOL=TCP)(HOST=$host)(PORT=$port))
			(CONNECT_DATA=(SID=$sid)))";
	$conn->Connect($cstr, 'scott', 'tiger');
```

f. ADOdb dsn:

```
	$dsn = 'oci8://user:pwd@tnsname?persist';  # persist is optional
	$conn = ADONewConnection($dsn);  # no need for Connect/PConnect
	
	$dsn = 'oci8://user:pwd@host/sid';
	$conn = ADONewConnection($dsn);
	
	$dsn = 'oci8://user:pwd@/';   # oracle on local machine
	$conn = ADONewConnection($dsn);
```

#### DSN-less ODBC (access and mssql examples)

ODBC DSN's can be created in the ODBC control panel, or you can use a DSN-less
connection.To use DSN-less connections with ODBC you need PHP 4.3 or later.

For Microsoft Access:

```
	$db =& ADONewConnection('access');
	$dsn = "Driver={Microsoft Access Driver (*.mdb)};Dbq=d:\\northwind.mdb;Uid=Admin;Pwd=;";
	$db->Connect($dsn);
```

For Microsoft SQL Server:

```
	$db =& ADONewConnection('odbc_mssql');
	$dsn = "Driver={SQL Server};Server=localhost;Database=northwind;";
	$db->Connect($dsn,'userid','password');
```

or if you prefer to use the mssql extension (which is limited to mssql 6.5 functionality):

```
	$db =& ADONewConnection('mssql');
	$db->Execute("localhost', 'userid', 'password', 'northwind');
```

**DSN-less Connections with ADO**  
If you are using versions of PHP earlier than PHP 4.3.0, DSN-less connections
only work with Microsoft's ADO, which is Microsoft's COM based API. An example
using the ADOdb library and Microsoft's ADO:

```
<?php
	include('adodb.inc.php'); 
	$db = &ADONewConnection("ado_mssql");
	print "<h1>Connecting DSN-less $db->databaseType...</h1>";
		
	$myDSN="PROVIDER=MSDASQL;DRIVER={SQL Server};"
		. "SERVER=flipper;DATABASE=ai;UID=sa;PWD=;"  ;
	$db->Connect($myDSN);
	
	$rs = $db->Execute("select * from table");
	$arr = $rs->GetArray();
	print_r($arr);
?>
```

## High Speed ADOdb - tuning tips

ADOdb is a big class library, yet it consistently beats all other PHP class
libraries in performance. This is because it is designed in a layered fashion,
like an onion, with the fastest functions in the innermost layer. Stick to the
following functions for best performance:

|  |
| --- |
| **Innermost Layer** |
| Connect, PConnect, NConnect  Execute, CacheExecute  SelectLimit, CacheSelectLimit  MoveNext, Close   qstr, Affected\_Rows, Insert\_ID |

The fastest way to access the field data is by accessing the array $recordset->fields
directly. Also set the global variables $ADODB\_FETCH\_MODE
= ADODB\_FETCH\_NUM, and (for oci8, ibase/firebird and odbc) $ADODB\_COUNTRECS = false
before you connect to your database.

Consider using bind parameters if your database supports it, as it improves
query plan reuse. Use ADOdb's performance tuning system to identify bottlenecks
quickly. At the time of writing (Dec 2003), this means oci8 and odbc drivers.

Lastly make sure you have a PHP accelerator cache installed such as APC, Turck
MMCache, Zend Accelerator or ionCube.

**Advanced Tips**

If you have the ADOdb C extension installed,
you can replace your calls to $rs->MoveNext() with adodb\_movenext($rs).
This doubles the speed of this operation. For retrieving entire recordsets at once,
use GetArray(), which uses the high speed extension function adodb\_getall($rs) internally.

Execute() is the default way to run queries. You can use the low-level functions \_Execute() and \_query()
to reduce query overhead. Both these functions share the same parameters as Execute().

If you do not have any bind parameters or your database supports binding (without emulation),
then you can call \_Execute() directly. Calling this function bypasses bind emulation. Debugging is still supported in \_Execute().

If you do not require debugging facilities nor emulated binding, and do not require a recordset to be returned, then you can call \_query. This is great for inserts, updates and deletes. Calling this function
bypasses emulated binding, debugging, and recordset handling. Either the resultid, true or false are returned by \_query().

For Informix, you can disable scrollable cursors with $db->cursorType = 0.

## Hacking ADOdb Safely

You might want to modify ADOdb for your own purposes. Luckily you can
still maintain backward compatibility by sub-classing ADOdb and using the $ADODB\_NEWCONNECTION
variable. $ADODB\_NEWCONNECTION allows you to override the behaviour of ADONewConnection().
ADOConnection() checks for this variable and will call
the function-name stored in this variable if it is defined.

In the following example, new functionality for the connection object
is placed in the *hack\_mysql* and *hack\_postgres7* classes. The recordset class naming convention
can be controlled using $rsPrefix. Here we set it to 'hack\_rs\_', which will make ADOdb use
*hack\_rs\_mysql* and *hack\_rs\_postgres7* as the recordset classes.

```
class hack_mysql extends adodb_mysql {
var $rsPrefix = 'hack_rs_';
  /* Your mods here */
}

class hack_rs_mysql extends ADORecordSet_mysql {
	 /* Your mods here */
}

class hack_postgres7 extends adodb_postgres7 {
var $rsPrefix = 'hack_rs_';
  /* Your mods here */
}

class hack_rs_postgres7 extends ADORecordSet_postgres7 {
 /* Your mods here */
}

$ADODB_NEWCONNECTION = 'hack_factory';

function& hack_factory($driver)
{
	if ($driver !== 'mysql' && $driver !== 'postgres7') return false;
	
	$driver = 'hack_'.$driver;
	$obj = new $driver();
	return $obj;
}

include_once('adodb.inc.php');
```

Don't forget to call the constructor of the parent class in your constructor. If you want to use the default ADOdb drivers return false in the above hack\_factory() function.

## PHP5 Features

ADOdb 4.02 or later will transparently determine which version of PHP you are using.
If PHP5 is detected, the following features become available:

- **Foreach iterators**: This is a very natural way of going through a recordset:

  ```
  	$ADODB_FETCH_MODE = ADODB_FETCH_NUM;
  	$rs = $db->Execute($sql);
  	foreach($rs as $k => $row) {
  		echo "r1=".$row[0]." r2=".$row[1]."<br>";
  	}
  ```

  - **Exceptions**: Just include *adodb-exceptions.inc.php* and you can now
    catch exceptions on errors as they occur.

    ```
    	include("../adodb-exceptions.inc.php"); 
    	include("../adodb.inc.php");	 
    	try { 
    		$db = NewADOConnection("oci8"); 
    		$db->Connect('','scott','bad-password'); 
    	} catch (exception $e) { 
    		var_dump($e); 
    		adodb_backtrace($e->gettrace());
    	}
    ```

    Note that reaching EOF is **not** considered an error nor an exception.
    If you want to use the default ADOdb drivers return false.

### Databases Supported

The *name* below is the value you pass to NewADOConnection($name) to create a connection object for that database.

|  |  |  |  |  |  |
| --- | --- | --- | --- | --- | --- |
| **Name** | **Tested** | **Database** | **RecordCount() usable** | **Prerequisites** | **Operating Systems** |
| **access** | B | Microsoft Access/Jet. You need to create an ODBC DSN. | Y/N | ODBC | Windows only |
| **ado** | B | Generic ADO, not tuned for specific databases. Allows DSN-less connections. For best performance, use an OLEDB provider. This is the base class for all ado drivers.  You can set $db->codePage before connecting. | ? depends on database | ADO or OLEDB provider | Windows only |
| **ado\_access** | B | Microsoft Access/Jet using ADO. Allows DSN-less connections. For best performance, use an OLEDB provider. | Y/N | ADO or OLEDB provider | Windows only |
| **ado\_mssql** | B | Microsoft SQL Server using ADO. Allows DSN-less connections. For best performance, use an OLEDB provider. | Y/N | ADO or OLEDB provider | Windows only |
| **db2** | A | DB2. Should work reliably as based on ODBC driver. | Y/N | DB2 CLI/ODBC interface | Unix and Windows. Unix install hints. I have had reports that the $host and $database params have to be reversed in Connect() when using the CLI interface. |
| **vfp** | A | Microsoft Visual FoxPro. You need to create an ODBC DSN. | Y/N | ODBC | Windows only |
| **fbsql** | C | FrontBase. | Y | ? | Unix and Windows |
| **ibase** | B | Interbase 6 or earlier. Some users report you might need to use this  $db->PConnect('localhost:c:/ibase/employee.gdb', "sysdba", "masterkey") to connect. Lacks Affected\_Rows currently.    You can set $db->dialect, $db->buffers and $db->charSet before connecting. | Y/N | Interbase client | Unix and Windows |
| ***firebird*** | C | Firebird version of interbase. | Y/N | Interbase client | Unix and Windows |
| ***borland\_ibase*** | C | Borland version of Interbase 6.5 or later. Very sad that the forks differ. | Y/N | Interbase client | Unix and Windows |
| **informix** | C | Generic informix driver. Use this if you are using Informix 7.3 or later. | Y/N | Informix client | Unix and Windows |
| **informix72** | C | Informix databases before Informix 7.3 that do no support SELECT FIRST. | Y/N | Informix client | Unix and Windows |
| **ldap** | C | LDAP driver. See this example for usage information. |  | LDAP extension | ? |
| **mssql** | A | Microsoft SQL Server 7 and later. Works with Microsoft SQL Server 2000 also. Note that date formating is problematic with this driver. For example, the PHP mssql extension does not return the seconds for datetime! | Y/N | Mssql client | Unix and Windows.   Unix install howto and another one. |
| **mssqlpo** | A | Portable mssql driver. Identical to above mssql driver, except that '||', the concatenation operator, is converted to '+'. Useful for porting scripts from most other sql variants that use ||. | Y/N | Mssql client | Unix and Windows.   Unix install howto. |
| **mysql** | A | MySQL without transaction support. You can also set $db->clientFlags before connecting. | Y | MySQL client | Unix and Windows |
| **mysqlt** or **maxsql** | A | MySQL with transaction support. We recommend using || as the concat operator for best portability. This can be done by running MySQL using:   *mysqld --ansi* or *mysqld --sql-mode=PIPES\_AS\_CONCAT* | Y/N | MySQL client | Unix and Windows |
| **oci8** | A | Oracle 8/9. Has more functionality than *oracle* driver (eg. Affected\_Rows). You might have to putenv('ORACLE\_HOME=...') before Connect/PConnect.  There are 2 ways of connecting - with server IP and service name:   *PConnect('serverip:1521','scott','tiger','service'*)  or using an entry in TNSNAMES.ORA or ONAMES or HOSTNAMES:   *PConnect(false, 'scott', 'tiger', $oraname)*.  Since 2.31, we support Oracle REF cursor variables directly (see ExecuteCursor). | Y/N | Oracle client | Unix and Windows |
| **oci805** | C | Supports reduced Oracle functionality for Oracle 8.0.5. SelectLimit is not as efficient as in the oci8 or oci8po drivers. | Y/N | Oracle client | Unix and Windows |
| **oci8po** | A | Oracle 8/9 portable driver. This is nearly identical with the oci8 driver except (a) bind variables in Prepare() use the ? convention, instead of :bindvar, (b) field names use the more common PHP convention of lowercase names.  Use this driver if porting from other databases is important. Otherwise the oci8 driver offers better performance. | Y/N | Oracle client | Unix and Windows |
| **odbc** | A | Generic ODBC, not tuned for specific databases. To connect, use   PConnect('DSN','user','pwd'). This is the base class for all odbc derived drivers. | ? depends on database | ODBC | Unix and Windows. Unix hints. |
| **odbc\_mssql** | C | Uses ODBC to connect to MSSQL | Y/N | ODBC | Unix and Windows. |
| **odbc\_oracle** | C | Uses ODBC to connect to Oracle | Y/N | ODBC | Unix and Windows. |
| **odbtp** | C | Generic odbtp driver. Odbtp is a software for accessing Windows ODBC data sources from other operating systems. | Y/N | odbtp | Unix and Windows |
| **odbtp\_unicode** | C | Odtbp with unicode support | Y/N | odbtp | Unix and Windows |
| **oracle** | C | Implements old Oracle 7 client API. Use oci8 driver if possible for better performance. | Y/N | Oracle client | Unix and Windows |
| **netezza** | C | Netezza driver. Netezza is based on postgres code-base. | Y | ? | ? |
| **postgres** | A | Generic PostgreSQL driver. Currently identical to postgres7 driver. | Y | PostgreSQL client | Unix and Windows. |
| **postgres64** | A | For PostgreSQL 6.4 and earlier which does not support LIMIT internally. | Y | PostgreSQL client | Unix and Windows. |
| **postgres7** | A | PostgreSQL which supports LIMIT and other version 7 functionality. | Y | PostgreSQL client | Unix and Windows. |
| **sapdb** | C | SAP DB. Should work reliably as based on ODBC driver. | Y/N | SAP ODBC client | ? |
| **sqlanywhere** | C | Sybase SQL Anywhere. Should work reliably as based on ODBC driver. | Y/N | SQL Anywhere ODBC client | ? |
| **sqlite** | B | SQLite. | Y | - | Unix and Windows. |
| **sqlitepo** | B | Portable SQLite driver. This is because assoc mode does not work like other drivers in sqlite. Namely, when selecting (joining) multiple tables, the table names are included in the assoc keys in the "sqlite" driver. In "sqlitepo" driver, the table names are stripped from the returned column names. When this results in a conflict, the first field get preference. | Y | - | Unix and Windows. |
| **sybase** | C | Sybase. | Y/N | Sybase client | Unix and Windows. |

The "Tested" column indicates how extensively the code has been tested
and used.   
A = well tested and used by many people  
B = tested and usable, but some features might not be implemented  
C = user contributed or experimental driver. Might not fully support all of
the latest features of ADOdb.

The column "RecordCount() usable" indicates whether RecordCount()
return the number of rows, or returns -1 when a SELECT statement is executed.
If this column displays Y/N then the RecordCount() is emulated when the global
variable $ADODB\_COUNTRECS=true (this is the default). Note that for large recordsets,
it might be better to disable RecordCount() emulation because substantial amounts
of memory are required to cache the recordset for counting. Also there is a
speed penalty of 40-50% if emulation is required. This is emulated in most databases
except for PostgreSQL and MySQL. This variable is checked every time a query
is executed, so you can selectively choose which recordsets to count.

---

# Tutorials

### Example 1: Select Statement

Task: Connect to the Access Northwind DSN, display the first 2 columns of each
row.

In this example, we create a ADOConnection object, which represents the connection
to the database. The connection is initiated with PConnect,
which is a persistent connection. Whenever we want to query the database, we
call the ADOConnection.Execute()
function. This returns an ADORecordSet object which is actually a cursor that
holds the current row in the array fields[].
We use MoveNext()
to move from row to row.

NB: A useful function that is not used in this example is SelectLimit,
which allows us to limit the number of rows shown.

```
<?
include('adodb.inc.php');	   # load code common to ADOdb
$conn = &ADONewConnection('access');	# create a connection
$conn->PConnect('northwind');   # connect to MS-Access, northwind DSN
$recordSet = &$conn->Execute('select * from products');
if (!$recordSet) 
	print $conn->ErrorMsg();
else
while (!$recordSet->EOF) {
	print $recordSet->fields[0].' '.$recordSet->fields[1].'<BR>';
	$recordSet->MoveNext();
}

$recordSet->Close(); # optional
$conn->Close(); # optional

?>
```

The $recordSet returned stores
the current row in the $recordSet->fields
array, indexed by column number (starting from zero). We use the MoveNext()
function to move to the next row. The EOF
property is set to true when end-of-file is reached. If an error occurs in Execute(),
we return false instead of a recordset.

The `$recordSet->fields[]` array is generated by the PHP database
extension. Some database extensions only index by number and do not index the
array by field name. To force indexing by name - that is associative arrays
- use the SetFetchMode function. Each recordset saves and uses whatever fetch
mode was set when the recordset was created in Execute() or SelectLimit().

```
	$db->SetFetchMode(ADODB_FETCH_NUM);
	$rs1 = $db->Execute('select * from table');
	$db->SetFetchMode(ADODB_FETCH_ASSOC);
	$rs2 = $db->Execute('select * from table');
	print_r($rs1->fields); # shows array([0]=>'v0',[1] =>'v1')
	print_r($rs2->fields); # shows array(['col1']=>'v0',['col2'] =>'v1')
```

To get the number of rows in the select statement, you can use $recordSet->RecordCount().
Note that it can return -1 if the number of rows returned cannot be determined.

### Example 2: Advanced Select with Field Objects

Select a table, display the first two columns. If the second column is a date
or timestamp, reformat the date to US format.

```
<?
include('adodb.inc.php');	   # load code common to ADOdb
$conn = &ADONewConnection('access');	# create a connection
$conn->PConnect('northwind');   # connect to MS-Access, northwind dsn
$recordSet = &$conn->Execute('select CustomerID,OrderDate from Orders');
if (!$recordSet) 
	print $conn->ErrorMsg();
else
while (!$recordSet->EOF) {
	$fld = $recordSet->FetchField(1);
	$type = $recordSet->MetaType($fld->type);

	if ( $type == 'D' || $type == 'T') 
		print $recordSet->fields[0].' '.
			$recordSet->UserDate($recordSet->fields[1],'m/d/Y').'<BR>';
	else 
		print $recordSet->fields[0].' '.$recordSet->fields[1].'<BR>';

	$recordSet->MoveNext();
}
$recordSet->Close(); # optional
$conn->Close(); # optional

?>
```

In this example, we check the field type of the second column using FetchField().
This returns an object with at least 3 fields.

- **name**: name of column
- **type**: native field type of column
- **max\_length**: maximum length of field. Some databases such as MySQL
  do not return the maximum length of the field correctly. In these cases max\_length
  will be set to -1.

We then use MetaType()
to translate the native type to a *generic* type. Currently the following
*generic* types are defined:

- **C**: character fields that should be shown in a <input type="text">
  tag.
- **X**: TeXt, large text fields that should be shown in a <textarea>
- **B**: Blobs, or Binary Large Objects. Typically images.- **D**: Date field
  - **T**: Timestamp field
  - **L**: Logical field (boolean or bit-field)
  - **I**:  Integer field
  - **N**: Numeric field. Includes autoincrement, numeric, floating point,
    real and integer.
  - **R**: Serial field. Includes serial, autoincrement integers. This works
    for selected databases.

If the metatype is of type date or timestamp, then we print it using the user
defined date format with UserDate(),
which converts the PHP SQL date string format to a user defined one. Another
use for MetaType()
is data validation before doing an SQL insert or update.

### Example 3: Inserting

Insert a row to the Orders table containing dates and strings that need to
be quoted before they can be accepted by the database, eg: the single-quote
in the word *John's*.

```
<?
include('adodb.inc.php');	   # load code common to ADOdb
$conn = &ADONewConnection('access');	# create a connection

$conn->PConnect('northwind');   # connect to MS-Access, northwind dsn
$shipto = $conn->qstr("John's Old Shoppe");

$sql = "insert into orders (customerID,EmployeeID,OrderDate,ShipName) ";
$sql .= "values ('ANATR',2,".$conn->DBDate(time()).",$shipto)";

if ($conn->Execute($sql) === false) {
	print 'error inserting: '.$conn->ErrorMsg().'<BR>';
}
?>
```

In this example, we see the advanced date and quote handling facilities of
ADOdb. The unix timestamp (which is a long integer) is appropriately formated
for Access with DBDate(),
and the right escape character is used for quoting the *John's Old Shoppe*,
which is*John'**'**s Old Shoppe* and not PHP's default *John**'**s
Old Shoppe* with qstr().

Observe the error-handling of the Execute statement. False is returned by
Execute() if an error occured. The error message
for the last error that occurred is displayed in ErrorMsg().
Note: *php\_track\_errors* might have to be enabled for error messages to
be saved.

### Example 4: Debugging

```
<?
include('adodb.inc.php');	   # load code common to ADOdb
$conn = &ADONewConnection('access');	# create a connection
$conn->PConnect('northwind');   # connect to MS-Access, northwind dsn
$shipto = $conn->qstr("John's Old Shoppe");
$sql = "insert into orders (customerID,EmployeeID,OrderDate,ShipName) ";
$sql .= "values ('ANATR',2,".$conn->FormatDate(time()).",$shipto)";
$conn->debug = true;
if ($conn->Execute($sql) === false) print 'error inserting';
?>
```

In the above example, we have turned on debugging by setting **debug = true**.
This will display the SQL statement before execution, and also show any error
messages. There is no need to call ErrorMsg()
in this case. For displaying the recordset, see the rs2html()
example.

Also see the section on Custom Error Handlers.

### Example 5: MySQL and Menus

Connect to MySQL database *agora*, and generate a <select> menu
from an SQL statement where the <option> captions are in the 1st column,
and the value to send back to the server is in the 2nd column.

```
<?
include('adodb.inc.php'); # load code common to ADOdb
$conn = &ADONewConnection('mysql');  # create a connection
$conn->PConnect('localhost','userid','','agora');# connect to MySQL, agora db
$sql = 'select CustomerName, CustomerID from customers';
$rs = $conn->Execute($sql);
print $rs->GetMenu('GetCust','Mary Rosli');
?>
```

Here we define a menu named GetCust, with the menu option 'Mary Rosli' selected.
See GetMenu().
We also have functions that return the recordset as an array: GetArray(),
and as an associative array with the key being the first column: GetAssoc().

### Example 6: Connecting to 2 Databases At Once

```
<?
include('adodb.inc.php');	 # load code common to ADOdb
$conn1 = &ADONewConnection('mysql');  # create a mysql connection
$conn2 = &ADONewConnection('oracle');  # create a oracle connection

$conn1->PConnect($server, $userid, $password, $database);
$conn2->PConnect(false, $ora_userid, $ora_pwd, $oraname);

$conn1->Execute('insert ...');
$conn2->Execute('update ...');
?>
```

### Example 7: Generating Update and Insert SQL

ADOdb 1.31 and later supports two new recordset functions: GetUpdateSQL( ) and
GetInsertSQL( ). This allow you to perform a "SELECT \* FROM table query WHERE...",
make a copy of the $rs->fields, modify the fields, and then generate the SQL to
update or insert into the table automatically.

We show how the functions can be used when accessing a table with the following
fields: (ID, FirstName, LastName, Created).

Before these functions can be called, you need to initialize the recordset
by performing a select on the table. Idea and code by Jonathan Younger jyounger#unilab.com.
Since ADOdb 2.42, you can pass a table name instead of a recordset into
GetInsertSQL (in $rs), and it will generate an insert statement for that table.

```
<?
#==============================================
# SAMPLE GetUpdateSQL() and GetInsertSQL() code
#==============================================
include('adodb.inc.php');
include('tohtml.inc.php');

#==========================
# This code tests an insert

$sql = "SELECT * FROM ADOXYZ WHERE id = -1"; 
# Select an empty record from the database

$conn = &ADONewConnection("mysql");  # create a connection
$conn->debug=1;
$conn->PConnect("localhost", "admin", "", "test"); # connect to MySQL, testdb
$rs = $conn->Execute($sql); # Execute the query and get the empty recordset

$record = array(); # Initialize an array to hold the record data to insert

# Set the values for the fields in the record
# Note that field names are case-insensitive
$record["firstname"] = "Bob";
$record["lastNamE"] = "Smith";
$record["creaTed"] = time();

# Pass the empty recordset and the array containing the data to insert
# into the GetInsertSQL function. The function will process the data and return
# a fully formatted insert sql statement.
$insertSQL = $conn->GetInsertSQL($rs, $record);

$conn->Execute($insertSQL); # Insert the record into the database

#==========================
# This code tests an update

$sql = "SELECT * FROM ADOXYZ WHERE id = 1"; 
# Select a record to update

$rs = $conn->Execute($sql); # Execute the query and get the existing record to update

$record = array(); # Initialize an array to hold the record data to update

# Set the values for the fields in the record
# Note that field names are case-insensitive
$record["firstname"] = "Caroline";
$record["LasTnAme"] = "Smith"; # Update Caroline's lastname from Miranda to Smith

# Pass the single record recordset and the array containing the data to update
# into the GetUpdateSQL function. The function will process the data and return
# a fully formatted update sql statement with the correct WHERE clause.
# If the data has not changed, no recordset is returned
$updateSQL = $conn->GetUpdateSQL($rs, $record);

$conn->Execute($updateSQL); # Update the record in the database
$conn->Close();
?>
```

**$ADODB\_FORCE\_TYPE**

The behaviour of GetUpdateSQL() and GetInsertSQL()
when converting empty or null PHP variables to SQL is controlled by the
global $ADODB\_FORCE\_TYPE variable. Set it to one of the values below. Default
is ADODB\_FORCE\_VALUE (3):

```
0 = ignore empty fields. All empty fields in array are ignored.
1 = force null. All empty, php null and string 'null' fields are changed to sql NULL values.
2 = force empty. All empty, php null and string 'null' fields are changed to sql empty '' or 0 values.
3 = force value. Value is left as it is. Php null and string 'null' are set to sql NULL values and 
    empty fields '' are set to empty '' sql values.

define('ADODB_FORCE_IGNORE',0);
define('ADODB_FORCE_NULL',1);
define('ADODB_FORCE_EMPTY',2);
define('ADODB_FORCE_VALUE',3);
```

Thanks to Niko (nuko#mbnet.fi) for the $ADODB\_FORCE\_TYPE code.

Note: the constant ADODB\_FORCE\_NULLS is obsolete since 4.52 and is ignored. Set $ADODB\_FORCE\_TYPE = ADODB\_FORCE\_NULL
for equivalent behaviour.

### Example 8: Implementing Scrolling with Next and Previous

The following code creates a very simple recordset pager, where you can scroll
from page to page of a recordset.

```
include_once('../adodb.inc.php');
include_once('../adodb-pager.inc.php');
session_start();

$db = NewADOConnection('mysql');

$db->Connect('localhost','root','','xphplens');

$sql = "select * from adoxyz ";

$pager = new ADODB_Pager($db,$sql);
$pager->Render($rows_per_page=5);
```

This will create a basic record pager that looks like this: 

|  |
| --- |
| `|<`   `<<`   `>>`   `>|` |
| ID | First Name | Last Name | Date Created || 36 | Alan | Turing | Sat 06, Oct 2001 | | 37 | Serena | Williams | Sat 06, Oct 2001 | | 38 | Yat Sun | Sun | Sat 06, Oct 2001 | | 39 | Wai Hun | See | Sat 06, Oct 2001 | | 40 | Steven | Oey | Sat 06, Oct 2001 | |
| Page 8/10 |

The number of rows to display at one time is controled by the Render($rows)
method. If you do not pass any value to Render(), ADODB\_Pager will default to
10 records per page.

You can control the column titles by modifying your SQL (supported by most
databases):

```
$sql = 'select id as "ID", firstname as "First Name", 
		  lastname as "Last Name", created as "Date Created"   
		from adoxyz';
```

The above code can be found in the *adodb/tests/testpaging.php* example
included with this release, and the class ADODB\_Pager in *adodb/adodb-pager.inc.php*.
The ADODB\_Pager code can be adapted by a programmer so that the text links can
be replaced by images, and the dull white background be replaced with more interesting
colors.

You can also allow display of html by setting $pager->htmlSpecialChars = false.

Some of the code used here was contributed by Iván Oliva and Cornel
G.

### Example 9: Exporting in CSV or Tab-Delimited Format

We provide some helper functions to export in comma-separated-value (CSV) and
tab-delimited formats:

```
include_once('/path/to/adodb/toexport.inc.php');  
include_once('/path/to/adodb/adodb.inc.php');  

$db = &NewADOConnection('mysql');  
$db->Connect($server, $userid, $password, $database);  
  
$rs = $db->Execute('select fname as "First Name", surname as "Surname" from table');

print "<pre>";  
print rs2csv($rs); # return a string, CSV format

print '<hr>';
  
$rs->MoveFirst(); # note, some databases do not support MoveFirst  
print rs2tab($rs,false); # return a string, tab-delimited
						 # false == suppress field names in first line

print '<hr>';  
$rs->MoveFirst();  
rs2tabout($rs); # send to stdout directly (there is also an rs2csvout function)
print "</pre>";

$rs->MoveFirst();  
$fp = fopen($path, "w");
if ($fp) {  
  rs2csvfile($rs, $fp); # write to file (there is also an rs2tabfile function)
  fclose($fp);  
}
```

Carriage-returns or newlines are converted to spaces. Field names are returned
in the first line of text. Strings containing the delimiter character are quoted
with double-quotes. Double-quotes are double-quoted again. This conforms to
Excel import and export guide-lines.

All the above functions take as an optional last parameter, $addtitles which
defaults to *true*. When set to *false* field names in the first line
are suppressed.   

### Example 10: Recordset Filters

Sometimes we want to pre-process all rows in a recordset before we use it.
For example, we want to ucwords all text in recordset.

```
include_once('adodb/rsfilter.inc.php');
include_once('adodb/adodb.inc.php');

// ucwords() every element in the recordset
function do_ucwords(&$arr,$rs)
{
	foreach($arr as $k => $v) {
		$arr[$k] = ucwords($v);
	}
}

$db = NewADOConnection('mysql');
$db->PConnect('server','user','pwd','db');

$rs = $db->Execute('select ... from table');
$rs = RSFilter($rs,'do_ucwords');
```

The *RSFilter* function takes 2 parameters, the recordset, and the name
of the *filter* function. It returns the processed recordset scrolled to
the first record. The *filter* function takes two parameters, the current
row as an array, and the recordset object. For future compatibility, you should
not use the original recordset object.

### Example 11: Smart Transactions

The old way of doing transactions required you to use

```
$conn->BeginTrans();
$ok = $conn->Execute($sql);
if ($ok) $ok = $conn->Execute($sql2);
if (!$ok) $conn->RollbackTrans();
else $conn->CommitTrans();
```

This is very complicated for large projects because you have to track the error
status. Smart Transactions is much simpler. You start a smart transaction by calling
StartTrans():

```
$conn->StartTrans();
$conn->Execute($sql);
$conn->Execute($Sql2);
$conn->CompleteTrans();
```

CompleteTrans() detects when an SQL error occurs, and will Rollback/Commit as
appropriate. To specificly force a rollback even if no error occured, use FailTrans().
Note that the rollback is done in CompleteTrans(), and not in FailTrans().

```
$conn->StartTrans();
$conn->Execute($sql);
if (!CheckRecords()) $conn->FailTrans();
$conn->Execute($Sql2);
$conn->CompleteTrans();
```

You can also check if a transaction has failed, using HasFailedTrans(), which
returns true if FailTrans() was called, or there was an error in the SQL execution.
Make sure you call HasFailedTrans() before you call CompleteTrans(), as it is
only works between StartTrans/CompleteTrans.

Lastly, StartTrans/CompleteTrans is nestable, and only the outermost block
is executed. In contrast, BeginTrans/CommitTrans/RollbackTrans is NOT nestable.

```
$conn->StartTrans();
$conn->Execute($sql);
  $conn->StartTrans();    # ignored
  if (!CheckRecords()) $conn->FailTrans();
  $conn->CompleteTrans(); # ignored
$conn->Execute($Sql2);
$conn->CompleteTrans();
```

Note: Savepoints are currently not supported.

## Using Custom Error Handlers and PEAR\_Error

ADOdb supports PHP5 exceptions. Just include *adodb-exceptions.inc.php* and you can now
catch exceptions on errors as they occur.

```
	include("../adodb-exceptions.inc.php"); 
	include("../adodb.inc.php");	 
	try { 
		$db = NewADOConnection("oci8://scott:bad-password@mytns/"); 
	} catch (exception $e) { 
		var_dump($e); 
		adodb_backtrace($e->gettrace());
	}
```

ADOdb also provides two custom handlers which you can modify for your needs. The
first one is in the **adodb-errorhandler.inc.php** file. This makes use of
the standard PHP functions error\_reporting
to control what error messages types to display, and trigger\_error
which invokes the default PHP error handler.

Including the above file will cause *trigger\_error($errorstring,E\_USER\_ERROR)*
to be called when  
(a) Connect() or PConnect() fails, or   
(b) a function that executes SQL statements such as Execute() or SelectLimit()
has an error.  
(c) GenID() appears to go into an infinite loop.

The $errorstring is generated by ADOdb and will contain useful debugging information
similar to the error.log data generated below. This file adodb-errorhandler.inc.php
should be included before you create any ADOConnection objects.

If you define error\_reporting(0), no errors will be passed to the error handler.
If you set error\_reporting(E\_ALL), all errors will be passed to the error handler.
You still need to use **ini\_set("display\_errors", "0" or "1")** to control
the display of errors.

```
<?php
error_reporting(E_ALL); # pass any error messages triggered to error handler
include('adodb-errorhandler.inc.php');
include('adodb.inc.php');
include('tohtml.inc.php');
$c = NewADOConnection('mysql');
$c->PConnect('localhost','root','','northwind');
$rs=$c->Execute('select * from productsz'); #invalid table productsz');
if ($rs) rs2html($rs);
?>
```

If you want to log the error message, you can do so by defining the following
optional constants ADODB\_ERROR\_LOG\_TYPE and ADODB\_ERROR\_LOG\_DEST. ADODB\_ERROR\_LOG\_TYPE
is the error log message type (see error\_log
in the PHP manual). In this case we set it to 3, which means log to the file
defined by the constant ADODB\_ERROR\_LOG\_DEST.

```
<?php
error_reporting(E_ALL); # report all errors
ini_set("display_errors", "0"); # but do not echo the errors
define('ADODB_ERROR_LOG_TYPE',3);
define('ADODB_ERROR_LOG_DEST','C:/errors.log');
include('adodb-errorhandler.inc.php');
include('adodb.inc.php');
include('tohtml.inc.php');

$c = NewADOConnection('mysql');
$c->PConnect('localhost','root','','northwind');
$rs=$c->Execute('select * from productsz'); ## invalid table productsz
if ($rs) rs2html($rs);
?>
```

The following message will be logged in the error.log file:

```
(2001-10-28 14:20:38) mysql error: [1146: Table 'northwind.productsz' doesn't exist] in
 EXECUTE("select * from productsz")
```

The second error handler is **adodb-errorpear.inc.php**. This will create a
PEAR\_Error derived object whenever an error occurs. The last PEAR\_Error object
created can be retrieved using ADODB\_Pear\_Error().

```
<?php
include('adodb-errorpear.inc.php');
include('adodb.inc.php');
include('tohtml.inc.php');
$c = NewADOConnection('mysql');
$c->PConnect('localhost','root','','northwind');
$rs=$c->Execute('select * from productsz'); #invalid table productsz');
if ($rs) rs2html($rs);
else {
	$e = ADODB_Pear_Error();
	echo '<p>',$e->message,'</p>';
}
?>
```

You can use a PEAR\_Error derived class by defining the constant ADODB\_PEAR\_ERROR\_CLASS
before the adodb-errorpear.inc.php file is included. For easy debugging, you
can set the default error handler in the beginning of the PHP script to PEAR\_ERROR\_DIE,
which will cause an error message to be printed, then halt script execution:

```
include('PEAR.php');
PEAR::setErrorHandling('PEAR_ERROR_DIE');
```

Note that we do not explicitly return a PEAR\_Error object to you when an error
occurs. We return false instead. You have to call ADODB\_Pear\_Error() to get
the last error or use the PEAR\_ERROR\_DIE technique.

#### Error Messages

Error messages are outputted using the static method ADOConnnection::outp($msg,$newline=true).
By default, it sends the messages to the client. You can override this to perform
error-logging.

## Data Source Names

We now support connecting using PEAR style DSN's. A DSN is a connection string
of the form:

$dsn = *"$driver://$username:$password@$hostname/$databasename"*;

An example:

```
   $username = 'root';
   $password = '';
   $hostname = 'localhost';
   $databasename = 'xphplens';
   $driver = 'mysql';
   $dsn = "$driver://$username:$password@$hostname/$databasename"
   $db = NewADOConnection(); 
   # DB::Connect($dsn) also works if you include 'adodb/adodb-pear.inc.php' at the top
   $rs = $db->query('select firstname,lastname from adoxyz');
   $cnt = 0;
   while ($arr = $rs->fetchRow()) {
		print_r($arr); print "<br>";
   }
```

More info and connection examples on the DSN format.

## PEAR Compatibility

We support DSN's (see above), and the following functions:

```
 DB_Common
 	query - returns PEAR_Error on error
	limitQuery - return PEAR_Error on error
	prepare - does not return PEAR_Error on error
	execute - does not return PEAR_Error on error
	setFetchMode - supports ASSOC and ORDERED
	errorNative
	quote
	nextID
	disconnect
	
	getOne
	getAssoc
	getRow
	getCol
	
 DB_Result
 	numRows - returns -1 if not supported
	numCols
	fetchInto - does not support passing of fetchmode
	fetchRows - does not support passing of fetchmode
	free
```

## Caching of Recordsets

ADOdb now supports caching of recordsets using the CacheExecute( ), CachePageExecute(
) and CacheSelectLimit( ) functions. There are similar to the non-cache functions,
except that they take a new first parameter, $secs2cache.

An example:

```
include('adodb.inc.php'); # load code common to ADOdb
$ADODB_CACHE_DIR = '/usr/ADODB_cache';
$conn = &ADONewConnection('mysql');  # create a connection
$conn->PConnect('localhost','userid','','agora');# connect to MySQL, agora db
$sql = 'select CustomerName, CustomerID from customers';
$rs = $conn->CacheExecute(15,$sql);
```

The first parameter is the number of seconds to cache
the query. Subsequent calls to that query will used the cached version stored
in $ADODB\_CACHE\_DIR. To force a query to execute and flush the cache, call CacheExecute()
with the first parameter set to zero. Alternatively, use the CacheFlush($sql)
call.

For the sake of security, we recommend you set *register\_globals=off*
in php.ini if you are using $ADODB\_CACHE\_DIR.

In ADOdb 1.80 onwards, the secs2cache parameter is optional in CacheSelectLimit()
and CacheExecute(). If you leave it out, it will use the $connection->cacheSecs
parameter, which defaults to 60 minutes.

```
	$conn->Connect(...);
	$conn->cacheSecs = 3600*24; # cache 24 hours
	$rs = $conn->CacheExecute('select * from table');
```

Please note that magic\_quotes\_runtime should be turned off. More
info, and do not change $ADODB\_FETCH\_MODE (or SetFetchMode)
as the cached recordset will use the $ADODB\_FETCH\_MODE set when the query was executed. 

## Pivot Tables

Since ADOdb 2.30, we support the generation of
SQL to create pivot tables, also known as cross-tabulations. For further explanation
read this DevShed Cross-Tabulation
tutorial. We assume that your database supports the SQL case-when expression.

In this example, we will use the Northwind database from Microsoft. In the
database, we have a products table, and we want to analyze this table by *suppliers
versus product categories*. We will place the suppliers on each row, and
pivot on categories. So from the table on the left, we generate the pivot-table
on the right:

|  |  |  |  |  |  |  |  |  |  |  |  |  |  |  |  |  |  |  |  |  |  |  |
| --- | --- | --- | --- | --- | --- | --- | --- | --- | --- | --- | --- | --- | --- | --- | --- | --- | --- | --- | --- | --- | --- | --- |
| |  |  | | --- | --- | | *Supplier* | *Category* | | supplier1 | category1 | | supplier2 | category1 | | supplier2 | category2 | | --> | |  |  |  |  | | --- | --- | --- | --- | |  | *category1* | *category2* | *total* | | *supplier1* | 1 | 0 | 1 | | *supplier2* | 1 | 1 | 2 | |

The following code will generate the SQL for a cross-tabulation:

```
# Query the main "product" table
# Set the rows to CompanyName
# and the columns to the values of Categories
# and define the joins to link to lookup tables 
# "categories" and "suppliers"
#
 include "adodb/pivottable.php";
 $sql = PivotTableSQL(
 	$gDB,                                      # adodb connection
 	'products p ,categories c ,suppliers s',   # tables
	'CompanyName',                             # rows (multiple fields allowed)
	'CategoryName',                            # column to pivot on 
	'p.CategoryID = c.CategoryID and s.SupplierID= p.SupplierID' # joins/where
);
```

This will generate the following SQL:

`SELECT CompanyName,   
SUM(CASE WHEN CategoryName='Beverages' THEN 1 ELSE 0 END) AS "Beverages",
  
SUM(CASE WHEN CategoryName='Condiments' THEN 1 ELSE 0 END) AS "Condiments",
  
SUM(CASE WHEN CategoryName='Confections' THEN 1 ELSE 0 END) AS "Confections",
  
SUM(CASE WHEN CategoryName='Dairy Products' THEN 1 ELSE 0 END) AS "Dairy
Products",   
SUM(CASE WHEN CategoryName='Grains/Cereals' THEN 1 ELSE 0 END) AS "Grains/Cereals",
  
SUM(CASE WHEN CategoryName='Meat/Poultry' THEN 1 ELSE 0 END) AS "Meat/Poultry",
  
SUM(CASE WHEN CategoryName='Produce' THEN 1 ELSE 0 END) AS "Produce",
  
SUM(CASE WHEN CategoryName='Seafood' THEN 1 ELSE 0 END) AS "Seafood",
  
SUM(1) as Total   
FROM products p ,categories c ,suppliers s WHERE p.CategoryID = c.CategoryID
and s.SupplierID= p.SupplierID   
GROUP BY CompanyName`

You can also pivot on *numerical columns* and *generate totals*
by using ranges. This code was revised in ADODB 2.41
and is not backward compatible. The second example shows this:

```
 $sql = PivotTableSQL(
 	$gDB,                                       # adodb connection
 	'products p ,categories c ,suppliers s',    # tables
	'CompanyName',                              # rows (multiple fields allowed)
   array(                                       # column ranges
	' 0 '      => 'UnitsInStock <= 0',
	"1 to 5"   => '0 < UnitsInStock and UnitsInStock <= 5',
	"6 to 10"  => '5 < UnitsInStock and UnitsInStock <= 10',
	"11 to 15" => '10 < UnitsInStock and UnitsInStock <= 15',
	"16+"      => '15 < UnitsInStock'
	),
	' p.CategoryID = c.CategoryID and s.SupplierID= p.SupplierID', # joins/where
	'UnitsInStock',                             # sum this field
	'Sum '                                      # sum label prefix
);
```

Which generates:

`SELECT CompanyName,   
SUM(CASE WHEN UnitsInStock <= 0 THEN UnitsInStock ELSE 0 END) AS "Sum
0 ",   
SUM(CASE WHEN 0 < UnitsInStock and UnitsInStock <= 5 THEN UnitsInStock
ELSE 0 END) AS "Sum 1 to 5",  
SUM(CASE WHEN 5 < UnitsInStock and UnitsInStock <= 10 THEN UnitsInStock
ELSE 0 END) AS "Sum 6 to 10",  
SUM(CASE WHEN 10 < UnitsInStock and UnitsInStock <= 15 THEN UnitsInStock
ELSE 0 END) AS "Sum 11 to 15",   
SUM(CASE WHEN 15 < UnitsInStock THEN UnitsInStock ELSE 0 END) AS "Sum
16+",   
SUM(UnitsInStock) AS "Sum UnitsInStock",   
SUM(1) as Total,  
FROM products p ,categories c ,suppliers s WHERE p.CategoryID = c.CategoryID
and s.SupplierID= p.SupplierID   
GROUP BY CompanyName`

---

# Class Reference

Function parameters with [ ] around them are optional.

## Global Variables

### $ADODB\_COUNTRECS

If the database driver API does not support counting the number of records
returned in a SELECT statement, the function RecordCount() is emulated when
the global variable $ADODB\_COUNTRECS is set to true, which is the default.
We emulate this by buffering the records, which can take up large amounts
of memory for big recordsets. Set this variable to false for the best performance.
This variable is checked every time a query is executed, so you can selectively
choose which recordsets to count.

### $ADODB\_CACHE\_DIR

If you are using recordset caching, this is the directory to save your recordsets
in. Define this before you call any caching functions such as CacheExecute(
). We recommend setting *register\_globals=off* in php.ini if you use this
feature for security reasons.

If you are using Unix and apache, you might need to set your cache directory
permissions to something similar to the following:

chown -R apache /path/to/adodb/cache  
chgrp -R apache /path/to/adodb/cache

### $ADODB\_ANSI\_PADDING\_OFF

Determines whether to right trim CHAR fields (and also VARCHAR for ibase/firebird).
Set to true to trim. Default is false. Currently works for oci8po, ibase and firebird
drivers. Added in ADOdb 4.01.

### $ADODB\_LANG

Determines the language used in MetaErrorMsg(). The default is 'en', for English.
To find out what languages are supported, see the files
in adodb/lang/adodb-$lang.inc.php, where $lang is the supported langauge.

### $ADODB\_FETCH\_MODE

This is a global variable that determines how arrays are retrieved by recordsets.
The recordset saves this value on creation (eg. in Execute( ) or SelectLimit(
)), and any subsequent changes to $ADODB\_FETCH\_MODE have no affect on existing
recordsets, only on recordsets created in the future.

The following constants are defined:

define('ADODB\_FETCH\_DEFAULT',0);  
define('ADODB\_FETCH\_NUM',1);  
define('ADODB\_FETCH\_ASSOC',2);  
define('ADODB\_FETCH\_BOTH',3);

An example:

```
	$ADODB_FETCH_MODE = ADODB_FETCH_NUM;
	$rs1 = $db->Execute('select * from table');
	$ADODB_FETCH_MODE = ADODB_FETCH_ASSOC;
	$rs2 = $db->Execute('select * from table');
	print_r($rs1->fields); # shows array([0]=>'v0',[1] =>'v1')
	print_r($rs2->fields); # shows array(['col1']=>'v0',['col2'] =>'v1')
```

As you can see in the above example, both recordsets store and use different
fetch modes based on the $ADODB\_FETCH\_MODE setting when the recordset was
created by Execute().

If no fetch mode is predefined, the fetch mode defaults to ADODB\_FETCH\_DEFAULT.
The behaviour of this default mode varies from driver to driver, so do not
rely on ADODB\_FETCH\_DEFAULT. For portability, we recommend sticking to ADODB\_FETCH\_NUM
or ADODB\_FETCH\_ASSOC. Many drivers do not support ADODB\_FETCH\_BOTH.

**SetFetchMode Function**

Some programmers prefer to use a more object-oriented solution, where the fetch
mode is set by a object function, SetFetchMode.
Once this function is called for a connection object, that connection object
will ignore the global variable $ADODB\_FETCH\_MODE and will use the internal
fetchMode property exclusively.

```
	$db->SetFetchMode(ADODB_FETCH_NUM);
	$rs1 = $db->Execute('select * from table');
	$db->SetFetchMode(ADODB_FETCH_ASSOC);
	$rs2 = $db->Execute('select * from table');
	print_r($rs1->fields); # shows array([0]=>'v0',[1] =>'v1')
	print_r($rs2->fields); # shows array(['col1']=>'v0',['col2'] =>'v1')
```

To retrieve the previous fetch mode, you can use check the $db->fetchMode
property, or use the return value of SetFetchMode( ).

**ADODB\_ASSOC\_CASE**

You can control the associative fetch case for certain drivers which behave
differently. For the sybase, oci8po, mssql, odbc and ibase drivers and all
drivers derived from them, ADODB\_ASSOC\_CASE will by default generate recordsets
where the field name keys are lower-cased. Use the constant ADODB\_ASSOC\_CASE
to change the case of the keys. There are 3 possible values:

0 = assoc lowercase field names. $rs->fields['orderid']  
1 = assoc uppercase field names. $rs->fields['ORDERID']  
2 = use native-case field names. $rs->fields['OrderID'] -- this is the
default since ADOdb 2.90

To use it, declare it before you incldue adodb.inc.php.

define('ADODB\_ASSOC\_CASE', 2); # use native-case for ADODB\_FETCH\_ASSOC  
include('adodb.inc.php');

### $ADODB\_FORCE\_TYPE

See the GetUpdateSQL tutorial.

---

## ADOConnection

Object that performs the connection to the database, executes SQL statements
and has a set of utility functions for standardising the format of SQL statements
for issues such as concatenation and date formats.

### ADOConnection Fields

**databaseType**: Name of the database system we are connecting to. Eg.
**odbc** or **mssql** or **mysql**.

**dataProvider**: The underlying mechanism used to connect to the database.
Normally set to **native**, unless using **odbc** or **ado**.

**host:** Name of server or data source name (DSN) to connect to.

**database**: Name of the database or to connect to. If ado is used, it
will hold the ado data provider.

**user**: Login id to connect to database. Password is not saved for security
reasons.

**raiseErrorFn**: Allows you to define an error handling function. See adodb-errorhandler.inc.php
for an example.

**debug**: Set to *true* to make debug statements to appear.

**concat\_operator**: Set to '+' or '||' normally. The operator used to concatenate
strings in SQL. Used by the **Concat** function.

**fmtDate**: The format used by the **DBDate**
function to send dates to the database. is '#Y-m-d#' for Microsoft Access,
and ''Y-m-d'' for MySQL.

**fmtTimeStamp:** The format used by the **DBTimeStamp**
function to send timestamps to the database.

**true**: The value used to represent true.Eg. '.T.'. for Foxpro, '1' for
Microsoft SQL.

**false:**  The value used to represent false. Eg. '.F.'. for Foxpro, '0'
for Microsoft SQL.

**replaceQuote**: The string used to escape quotes. Eg. double single-quotes
for Microsoft SQL, and backslash-quote for MySQL. Used by qstr.

**autoCommit**: indicates whether automatic commit is enabled. Default is
true.

**charSet**: set the default charset to use. Currently only interbase supports
this.

**dialect**: set the default sql dialect to use. Currently only interbase
supports this.

**metaTablesSQL**: SQL statement to return a list of available tables. Eg.
*SHOW TABLES* in MySQL.

**genID**: The latest id generated by GenID() if supported by the database.

**cacheSecs**: The number of seconds to cache recordsets if CacheExecute()
or CacheSelectLimit() omit the $secs2cache parameter. Defaults to 60 minutes.

**sysDate**: String that holds the name of the database function to call
to get the current date. Useful for inserts and updates.

**sysTimeStamp**: String that holds the name of the database function to
call to get the current timestamp/datetime value.

**leftOuter**: String that holds operator for left outer join, if known.
Otherwise set to false.

**rightOuter**: String that holds operator for left outer join, if known.
Otherwise set to false.

**ansiOuter**: Boolean that if true indicates that ANSI style outer joins
are permitted. Eg. *select \* from table1 left join table2 on p1=p2.*

**connectSID**: Boolean that indicates whether to treat the $database parameter
in connects as the SID for the oci8 driver. Defaults to false. Useful for
Oracle 8.0.5 and earlier.

**autoRollback**: Persistent connections are auto-rollbacked in PConnect(
) if this is set to true. Default is false.

---

### ADOConnection Main Functions

**ADOConnection( )**

Constructor function. Do not call this directly. Use ADONewConnection( ) instead.

**Connect($host,[$user],[$password],[$database])**

Non-persistent connect to data source or server $**host**, using userid
$**user** and password $**password**. If the server supports multiple
databases, connect to database $**database**.

Returns true/false depending on connection success. Since 4.23, null is returned if the extension is not loaded.

ADO Note: If you are using a Microsoft ADO and not OLEDB, you can set the $database
parameter to the OLEDB data provider you are using.

PostgreSQL: An alternative way of connecting to the database is to pass the
standard PostgreSQL connection string in the first parameter $host, and the
other parameters will be ignored.

For Oracle and Oci8, there are two ways to connect. First is to use the TNS
name defined in your local tnsnames.ora (or ONAMES or HOSTNAMES). Place the
name in the $database field, and set the $host field to false. Alternatively,
set $host to the server, and $database to the database SID, this bypassed
tnsnames.ora.

Examples:

```
 # $oraname in tnsnames.ora/ONAMES/HOSTNAMES
 $conn->Connect(false, 'scott', 'tiger', $oraname); 
 $conn->Connect('server:1521', 'scott', 'tiger', 'ServiceName'); # bypass tnsnames.ora
```

There are many examples of connecting to a database.
See Connection Examples, php.weblogs.com/ADOdb,
and in the testdatabases.inc.php file included in the release.

**PConnect($host,[$user],[$password],[$database])**

Persistent connect to data source or server $**host**, using userid $**user**
and password $**password**. If the server supports multiple databases,
connect to database $**database**.

We now perform a rollback on persistent connection for selected databases since
2.21, as advised in the PHP manual. See change log or source code for which
databases are affected.

Returns true/false depending on connection. Since 4.23, null is returned if the extension is not loaded.
See Connect( ) above for more info.

Since ADOdb 2.21, we also support autoRollback. If you set:

```
 $conn = &NewADOConnection('mysql');
 $conn->autoRollback = true; # default is false
 $conn->PConnect(...); # rollback here
```

Then when doing a persistent connection with PConnect( ), ADOdb will
perform a rollback first. This is because it is documented that PHP is
not guaranteed to rollback existing failed transactions when
persistent connections are used. This is implemented in Oracle,
MySQL, PgSQL, MSSQL, ODBC currently.

Since ADOdb 3.11, you can force non-persistent
connections even if PConnect is called by defining the constant
ADODB\_NEVER\_PERSIST before you call PConnect.

Since 4.23, null is returned if the extension is not loaded.

**NConnect($host,[$user],[$password],[$database])**

Always force a new connection. In contrast, PHP sometimes reuses connections
when you use Connect() or PConnect(). Currently works only on mysql (PHP 4.3.0
or later), postgresql and oci8-derived drivers. For other drivers, NConnect() works like
Connect().

**Execute($sql,$inputarr=false)**

Execute SQL statement $**sql** and return derived class of ADORecordSet
if successful. Note that a record set is always returned on success, even
if we are executing an insert or update statement. You can also pass in $sql a statement prepared
in Prepare().

Returns derived class of ADORecordSet. Eg. if connecting via mysql, then ADORecordSet\_mysql
would be returned. False is returned if there was an error in executing the
sql.

The $inputarr parameter can be used for binding variables to parameters. Below
is an Oracle example:

```
 $conn->Execute("SELECT * FROM TABLE WHERE COND=:val", array('val'=> $val));
```

Another example, using ODBC,which uses the ? convention:

```
  $conn->Execute("SELECT * FROM TABLE WHERE COND=?", array($val));
```

*Binding variables*

Variable binding speeds the compilation and caching of SQL statements, leading
to higher performance. Currently Oracle, Interbase and ODBC supports variable binding.
Interbase/ODBC style ? binding is emulated in databases that do not support binding.
Note that you do not have to quote strings if you use binding.

Variable binding in the odbc, interbase and oci8po drivers.

```
$rs = $db->Execute('select * from table where val=?', array('10'));
```

Variable binding in the oci8 driver:

```
$rs = $db->Execute('select name from table where val=:key', 
  array('key' => 10));
```

*Bulk binding*

Since ADOdb 3.80, we support bulk binding in Execute(), in which you pass in a 2-dimensional array to
be bound to an INSERT/UPDATE or DELETE statement.

```
$arr = array(
	array('Ahmad',32),
	array('Zulkifli', 24),
	array('Rosnah', 21)
	);
$ok = $db->Execute('insert into table (name,age) values (?,?)',$arr);
```

This provides very high performance as the SQL statement is prepared first.
The prepared statement is executed repeatedly for each array row until all rows are completed,
or until the first error. Very useful for importing data.

**CacheExecute([$secs2cache,]$sql,$inputarr=false)**

Similar to Execute, except that the recordset is cached for $secs2cache seconds
in the $ADODB\_CACHE\_DIR directory, and $inputarr only accepts 1-dimensional arrays.
If CacheExecute() is called again with the same $sql, $inputarr,
and also the same database, same userid, and the cached recordset
has not expired, the cached recordset is returned.

```
  include('adodb.inc.php'); 
  include('tohtml.inc.php');
  $ADODB_CACHE_DIR = '/usr/local/ADOdbcache';
  $conn = &ADONewConnection('mysql'); 
  $conn->PConnect('localhost','userid','password','database');
  $rs = $conn->CacheExecute(15, 'select * from table'); # cache 15 secs
  rs2html($rs); /* recordset to html table */
```

Alternatively, since ADOdb 1.80, the $secs2cache parameter is optional:

```
	$conn->Connect(...);
   	$conn->cacheSecs = 3600*24; // cache 24 hours
	$rs = $conn->CacheExecute('select * from table');
```

If $secs2cache is omitted, we use the value
in $connection->cacheSecs (default is 3600 seconds, or 1 hour). Use CacheExecute()
only with SELECT statements.

Performance note: I have done some benchmarks and found that they vary so greatly
that it's better to talk about when caching is of benefit. When your database
server is *much slower* than your Web server or the database is *very
overloaded* then ADOdb's caching is good because it reduces the load on
your database server. If your database server is lightly loaded or much faster
than your Web server, then caching could actually reduce performance.

**ExecuteCursor($sql,$cursorName='rs',$parameters=false)**

Execute an Oracle stored procedure, and returns an Oracle REF cursor variable as
a regular ADOdb recordset. Does not work with any other database except oci8.
Thanks to Robert Tuttle for the design.

```
    $db = ADONewConnection("oci8"); 
    $db->Connect("foo.com:1521", "uid", "pwd", "FOO"); 
    $rs = $db->ExecuteCursor("begin :cursorvar := getdata(:param1); end;", 
					'cursorvar',
					array('param1'=>10)); 
    # $rs is now just like any other ADOdb recordset object  
    rs2html($rs);
```

ExecuteCursor() is a helper function that does the following internally:

```
	$stmt = $db->Prepare("begin :cursorvar := getdata(:param1); end;", true); 
	$db->Parameter($stmt, $cur, 'cursorvar', false, -1, OCI_B_CURSOR);
	$rs = $db->Execute($stmt,$bindarr);
```

ExecuteCursor only accepts 1 out parameter. So if you have 2 out parameters, use:

```
	$vv = 'A%';
	$stmt = $db->PrepareSP("BEGIN list_tabs(:crsr,:tt); END;");
	$db->OutParameter($stmt, $cur, 'crsr', -1, OCI_B_CURSOR);
	$db->OutParameter($stmt, $vv, 'tt', 32); # return varchar(32)
	$arr = $db->GetArray($stmt);
	print_r($arr);
	echo " val = $vv"; ## outputs 'TEST'
```

for the following PL/SQL:

```
	TYPE TabType IS REF CURSOR RETURN TAB%ROWTYPE;

	PROCEDURE list_tabs(tabcursor IN OUT TabType,tablenames IN OUT VARCHAR) IS
	BEGIN
		OPEN tabcursor FOR SELECT * FROM TAB WHERE tname LIKE tablenames;
		tablenames := 'TEST';
	END list_tabs;
```

**SelectLimit($sql,$numrows=-1,$offset=-1,$inputarr=false)**

Returns a recordset if successful. Returns false otherwise. Performs a select
statement, simulating PostgreSQL's SELECT statement, LIMIT $numrows OFFSET
$offset clause.

In PostgreSQL, SELECT \* FROM TABLE LIMIT 3 will return the first 3 records
only. The equivalent is `$connection->SelectLimit('SELECT * FROM TABLE',3)`.
This functionality is simulated for databases that do not possess this feature.

And SELECT \* FROM TABLE LIMIT 3 OFFSET 2 will return records 3, 4 and 5 (eg.
after record 2, return 3 rows). The equivalent in ADOdb is `$connection->SelectLimit('SELECT
* FROM TABLE',3,2)`.

Note that this is the *opposite* of MySQL's LIMIT clause. You can also
set `$connection->SelectLimit('SELECT * FROM TABLE',-1,10)` to
get rows 11 to the last row.

The last parameter $inputarr is for databases that support variable binding
such as Oracle oci8. This substantially reduces SQL compilation overhead.
Below is an Oracle example:

```
 $conn->SelectLimit("SELECT * FROM TABLE WHERE COND=:val", 100,-1,array('val'=> $val));
```

The oci8po driver (oracle portable driver) uses the more standard bind variable
of ?:

```
 $conn->SelectLimit("SELECT * FROM TABLE WHERE COND=?", 100,-1,array('val'=> $val));
```

Ron Wilson reports that SelectLimit does not work with UNIONs.

**CacheSelectLimit([$secs2cache,] $sql, $numrows=-1,$offset=-1,$inputarr=false)**

Similar to SelectLimit, except that the recordset returned is cached for $secs2cache
seconds in the $ADODB\_CACHE\_DIR directory.

Since 1.80, $secs2cache has been optional, and you can define the caching time
in $connection->cacheSecs.

```
	$conn->Connect(...);
   $conn->cacheSecs = 3600*24; // cache 24 hours
	$rs = $conn->CacheSelectLimit('select * from table',10);
```

**CacheFlush($sql=false,$inputarr=false)**

Flush (delete) any cached recordsets for the SQL statement $sql in $ADODB\_CACHE\_DIR.

If no parameter is passed in, then all adodb\_\*.cache files are deleted.

If you want to flush all cached recordsets manually, execute the following
PHP code (works only under Unix):   
 `` system("rm -f `find ".$ADODB_CACHE_DIR." -name
adodb_*.cache`"); ``

For general cleanup of all expired files, you should use crontab
on Unix, or at.exe on Windows, and a shell script similar to the following:  
#------------------------------------------------------   
# This particular example deletes files in the TMPPATH   
# directory with the string ".cache" in their name that   
# are more than 7 days old.   
#------------------------------------------------------   
AGED=7   
find ${TMPPATH} -mtime +$AGED | grep "\.cache" | xargs rm -f

**MetaError($errno=false)**

Returns a virtualized error number, based on PEAR DB's error number system. You might
need to include adodb-error.inc.php before you call this function. The parameter $errno
is the native error number you want to convert. If you do not pass any parameter, MetaError
will call ErrorNo() for you and convert it. If the error number cannot be virtualized, MetaError
will return -1 (DB\_ERROR).

**MetaErrorMsg($errno)**

Pass the error number returned by MetaError() for the equivalent textual error message.

**ErrorMsg()**

Returns the last status or error message. The error message is reset after every
call to Execute().

This can return a string even if
no error occurs. In general you do not need to call this function unless an
ADOdb function returns false on an error.

Note: If **debug** is enabled, the SQL error message is always displayed
when the **Execute** function is called.

**ErrorNo()**

Returns the last error number. The error number is reset after every call to Execute().
If 0 is returned, no error occurred.

Note that old versions of PHP (pre 4.0.6) do
not support error number for ODBC. In general you do not need to call this
function unless an ADOdb function returns false on an error.

**SetFetchMode($mode)**

Sets the current fetch mode for the connection and stores
it in $db->fetchMode. Legal modes are ADODB\_FETCH\_ASSOC and ADODB\_FETCH\_NUM.
For more info, see $ADODB\_FETCH\_MODE.

Returns the previous fetch mode, which could be false
if SetFetchMode( ) has not been called before.

**CreateSequence($seqName = 'adodbseq',$startID=1)**

Create a sequence. The next time GenID( ) is called, the value returned will
be $startID. Added in 2.60.

**DropSequenceD($seqName = 'adodbseq')**

Delete a sequence. Added in 2.60.

**GenID($seqName = 'adodbseq',$startID=1)**

Generate a sequence number . Works for interbase,
mysql, postgresql, oci8, oci8po, mssql, ODBC based (access,vfp,db2,etc) drivers
currently. Uses $seqName as the name of the sequence. GenID() will automatically
create the sequence for you if it does not exist (provided the userid has
permission to do so). Otherwise you will have to create the sequence yourself.

If your database driver emulates sequences, the name of the table is the sequence
name. The table has one column, "id" which should be of type integer, or if
you need something larger - numeric(16).

For ODBC and databases that do not support sequences natively (eg mssql, mysql),
we create a table for each sequence. If the sequence has not been defined
earlier, it is created with the starting value set in $startID.

Note that the mssql driver's GenID() before 1.90 used to generate 16 byte GUID's.

**UpdateBlob($table,$column,$val,$where)**

Allows you to store a blob (in $val) into $table into $column in a row at $where.

Usage:

```
	# for oracle
	$conn->Execute('INSERT INTO blobtable (id, blobcol) VALUES (1, empty_blob())');
	$conn->UpdateBlob('blobtable','blobcol',$blobvalue,'id=1');
	
	# non oracle databases
	$conn->Execute('INSERT INTO blobtable (id, blobcol) VALUES (1, null)');
	$conn->UpdateBlob('blobtable','blobcol',$blobvalue,'id=1');
```

Returns true if succesful, false otherwise. Supported by MySQL, PostgreSQL,
Oci8, Oci8po and Interbase drivers. Other drivers might work, depending on
the state of development.

Note that when an Interbase blob is retrieved using SELECT, it still needs
to be decoded using $connection->DecodeBlob($blob); to derive the original
value in versions of PHP before 4.1.0.

For PostgreSQL, you can store your blob using blob oid's or as a bytea field.
You can use bytea fields but not blob oid's currently with UpdateBlob( ).
Conversely UpdateBlobFile( ) supports oid's, but not bytea data.  
  
If you do not pass in an oid, then UpdateBlob() assumes that you are storing
in bytea fields.

**UpdateClob($table,$column,$val,$where)**

Allows you to store a clob (in $val) into $table into $column in a row at $where.
Similar to UpdateBlob (see above), but for Character Large OBjects.

Usage:

```
	# for oracle
	$conn->Execute('INSERT INTO clobtable (id, clobcol) VALUES (1, empty_clob())');
	$conn->UpdateBlob('clobtable','clobcol',$clobvalue,'id=1');
	
	# non oracle databases
	$conn->Execute('INSERT INTO clobtable (id, clobcol) VALUES (1, null)');
	$conn->UpdateBlob('clobtable','clobcol',$clobvalue,'id=1');
```

**UpdateBlobFile($table,$column,$path,$where,$blobtype='BLOB')**

Similar to UpdateBlob, except that we pass in a file path to where the blob
resides.

For PostgreSQL, if you are using blob oid's, use this interface. This interface
does not support bytea fields.

Returns true if successful, false otherwise.

**BlobEncode($blob)**

Some databases require blob's to be encoded manually before upload. Note if
you use UpdateBlob( ) or UpdateBlobFile( ) the conversion is done automatically
for you and you do not have to call this function. For PostgreSQL, currently,
BlobEncode() can only be used for bytea fields.

Returns the encoded blob value.

Note that there is a connection property called *blobEncodeType* which
has 3 legal values:

false - no need to perform encoding or decoding.  
'I' - blob encoding required, and returned encoded blob is a numeric value
(no need to quote).  
'C' - blob encoding required, and returned encoded blob is a character value
(requires quoting).

This is purely for documentation purposes, so that programs that accept multiple
database drivers know what is the right thing to do when processing blobs.

**BlobDecode($blob)**

Some databases require blob's to be decoded manually after doing a select statement.
If the database does not require decoding, then this function will return
the blob unchanged. Currently BlobDecode is only required for one database,
PostgreSQL, and only if you are using blob oid's (if you are using bytea fields,
we auto-decode for you).

```
$rs = $db->Execute("select bloboid from postgres_table where id=$key");
$blob = $db->BlobDecode( reset($rs->fields) );
```

**Replace($table, $arrFields, $keyCols,$autoQuote=false)**

Try to update a record, and if the record is not found, an insert statement
is generated and executed. Returns 0 on failure, 1 if update statement worked,
2 if no record was found and the insert was executed successfully. This differs
from MySQL's replace which deletes the record and inserts a new record. This
also means you cannot update the primary key. The only exception to this is
Interbase and its derivitives, which uses delete and insert because of some
Interbase API limitations.

The parameters are $table which is the table name, the $keyCols which is an
associative array where the keys are the field names, and keyCols is the name
of the primary key, or an array of field names if it is a compound key. If
$autoQuote is set to true, then Replace() will quote all values that are non-numeric;
auto-quoting will not quote nulls. Note that auto-quoting will not work if
you use SQL functions or operators.

Examples:

```
# single field primary key
$ret = $db->Replace('atable', 
	array('id'=>1000,'firstname'=>'Harun','lastname'=>'Al-Rashid'),
	'id',$autoquote = true);	
# generates UPDATE atable SET firstname='Harun',lastname='Al-Rashid' WHERE id=1000
# or INSERT INTO atable (id,firstname,lastname) VALUES (1000,'Harun','Al-Rashid')

# compound key
$ret = $db->Replace('atable2', 
	array('firstname'=>'Harun','lastname'=>'Al-Rashid', 'age' => 33, 'birthday' => 'null'),
	array('lastname','firstname'),
	$autoquote = true);

# no auto-quoting
$ret = $db->Replace('atable2', 
	array('firstname'=>"'Harun'",'lastname'=>"'Al-Rashid'", 'age' => 'null'),
	array('lastname','firstname'));	
```

**GetUpdateSQL(&$rs, $arrFields, $forceUpdate=false,$magicq=false, $force=null)**

Generate SQL to update a table given a recordset $rs, and the modified fields
of the array $arrFields (which must be an associative array holding the column
names and the new values) are compared with the current recordset. If $forceUpdate
is true, then we also generate the SQL even if $arrFields is identical to
$rs->fields. Requires the recordset to be associative. $magicq is used
to indicate whether magic quotes are enabled (see qstr()). The field names in the array
are case-insensitive.

Since 4.52, we allow you to pass the $force type parameter, and this overrides the $ADODB\_FORCE\_TYPE
global variable.

**GetInsertSQL(&$rs, $arrFields,$magicq=false,$force=false)**

Generate SQL to insert into a table given a recordset $rs. Requires the query
to be associative. $magicq is used to indicate whether magic quotes are enabled
(for qstr()). The field names in the array are case-insensitive.

Since 2.42, you can pass a table name instead of a recordset into
GetInsertSQL (in $rs), and it will generate an insert statement for that table.

Since 4.52, we allow you to pass the $force type parameter, and this overrides the $ADODB\_FORCE\_TYPE
global variable.

**PageExecute($sql, $nrows, $page, $inputarr=false)**

Used for pagination of recordset. $page is 1-based. See Example
8.

**CachePageExecute($secs2cache,
$sql, $nrows, $page, $inputarr=false)**

Used for pagination of recordset. $page is 1-based. See
Example 8. Caching version of PageExecute.

**Close( )**

Close the database connection. PHP4 proudly states that we no longer have to
clean up at the end of the connection because the reference counting mechanism
of PHP4 will automatically clean up for us.

**StartTrans( )**

Start a monitored transaction. As SQL statements are executed, ADOdb will monitor
for SQL errors, and if any are detected, when CompleteTrans() is called, we auto-rollback.

To understand why StartTrans() is superior to BeginTrans(),
let us examine a few ways of using BeginTrans().
The following is the wrong way to use transactions:

```
$DB->BeginTrans();
$DB->Execute("update table1 set val=$val1 where id=$id");
$DB->Execute("update table2 set val=$val2 where id=$id");
$DB->CommitTrans();
```

because you perform no error checking. It is possible to update table1 and
for the update on table2 to fail. Here is a better way:

```
$DB->BeginTrans();
$ok = $DB->Execute("update table1 set val=$val1 where id=$id");
if ($ok) $ok = $DB->Execute("update table2 set val=$val2 where id=$id");
if ($ok) $DB->CommitTrans();
else $DB->RollbackTrans();
```

Another way is (since ADOdb 2.0):

```
$DB->BeginTrans();
$ok = $DB->Execute("update table1 set val=$val1 where id=$id");
if ($ok) $ok = $DB->Execute("update table2 set val=$val2 where id=$id");
$DB->CommitTrans($ok);
```

Now it is a headache monitoring $ok all over the place. StartTrans() is an
improvement because it monitors all SQL errors for you. This is particularly
useful if you are calling black-box functions in which SQL queries might be executed.
Also all BeginTrans, CommitTrans and RollbackTrans calls inside a StartTrans block
will be disabled, so even if the black box function does a commit, it will be ignored.

```
$DB->StartTrans();
CallBlackBox();
$DB->Execute("update table1 set val=$val1 where id=$id");
$DB->Execute("update table2 set val=$val2 where id=$id");
$DB->CompleteTrans($ok);
```

Note that a StartTrans blocks are nestable, the inner blocks are ignored.

**CompleteTrans($autoComplete=true)**

Complete a transaction called with StartTrans(). This function monitors
for SQL errors, and will commit if no errors have occured, otherwise it will rollback.
Returns true on commit, false on rollback. If the parameter $autoComplete is true
monitor sql errors and commit and rollback as appropriate. Set $autoComplete to false
to force rollback even if no SQL error detected.

**FailTrans( )**

Fail a transaction started with StartTrans(). The rollback will only occur when
CompleteTrans() is called.

**HasFailedTrans( )**

Check whether smart transaction has failed,
eg. returns true if there was an error in SQL execution or FailTrans() was called.
If not within smart transaction, returns false.

**BeginTrans( )**

Begin a transaction. Turns off autoCommit. Returns true if successful. Some
databases will always return false if transaction support is not available.
Any open transactions will be rolled back when the connection is closed. Among the
databases that support transactions are Oracle, PostgreSQL, Interbase, MSSQL, certain
versions of MySQL, DB2, Informix, Sybase, etc.

Note that StartTrans() and CompleteTrans() is a superior method of
handling transactions, available since ADOdb 3.40. For a explanation, see the StartTrans() documentation.

You can also use the ADOdb error handler to die
and rollback your transactions for you transparently. Some buggy database extensions
are known to commit all outstanding tranasactions, so you might want to explicitly
do a $DB->RollbackTrans() in your error handler for safety.

#### Detecting Transactions

Since ADOdb 2.50, you are able to detect when you are inside a transaction. Check
that $connection->transCnt > 0. This variable is incremented whenever BeginTrans() is called,
and decremented whenever RollbackTrans() or CommitTrans() is called.

**CommitTrans($ok=true)**

End a transaction successfully. Returns true if successful. If the database
does not support transactions, will return true also as data is always committed.

If you pass the parameter $ok=false, the data is rolled back. See example in
BeginTrans().

**RollbackTrans( )**

End a transaction, rollback all changes. Returns true if successful. If the
database does not support transactions, will return false as data is never rollbacked.

**GetAssoc($sql,$inputarr=false,$force\_array=false,$first2cols=false)**

Returns an associative array for the given query $sql with optional bind parameters
in $inputarr. If the number of columns returned is greater to two, a 2-dimensional
array is returned, with the first column of the recordset becomes the keys
to the rest of the rows. If the columns is equal to two, a 1-dimensional array
is created, where the the keys directly map to the values (unless $force\_array
is set to true, when an array is created for each value).

Examples:

We have the following data in a recordset:

row1: Apple, Fruit, Edible  
row2: Cactus, Plant, Inedible  
row3: Rose, Flower, Edible

GetAssoc will generate the following 2-dimensional associative
array:

Apple => array[Fruit, Edible]  
Cactus => array[Plant, Inedible]  
Rose => array[Flower,Edible]

If the dataset is:

row1: Apple,
Fruit  
row2: Cactus, Plant  
row3: Rose, Flower

GetAssoc will generate the following
1-dimensional associative array (with $force\_array==false):

Apple => Fruit  
Cactus=>Plant  
Rose=>Flower

The function returns:

The associative array, or false if an error occurs.

**CacheGetAssoc([$secs2cache,] $sql,$inputarr=false,$force\_array=false,$first2cols=false)**

Caching version of GetAssoc function above.

**GetOne($sql,$inputarr=false)**

Executes the SQL and returns the first field of the first row. The recordset
and remaining rows are discarded for you automatically. If an error occur, false
is returned.

**GetRow($sql,$inputarr=false)**

Executes the SQL and returns the first row as an array. The recordset and remaining
rows are discarded for you automatically. If an error occurs, false is returned.

**GetAll($sql)**

Executes the SQL and returns the all the rows as a 2-dimensional
array. The recordset is discarded for you automatically. If an error occurs,
false is returned.

**GetCol($sql,$inputarr=false,$trim=false)**

Executes the SQL and returns all elements of the first column as a
1-dimensional array. The recordset is discarded for you automatically. If an error occurs,
false is returned.

**CacheGetOne([$secs2cache,]
$sql,$inputarr=false), CacheGetRow([$secs2cache,] $sql,$inputarr=false), CacheGetAll([$secs2cache,]
$sql,$inputarr=false), CacheGetCol([$secs2cache,]
$sql,$inputarr=false,$trim=false)**

Similar to above Get\* functions, except that the recordset is serialized and
cached in the $ADODB\_CACHE\_DIR directory for $secs2cache seconds. Good for speeding
up queries on rarely changing data. Note that the $secs2cache parameter is optional.
If omitted, we use the value in $connection->cacheSecs (default is 3600 seconds,
or 1 hour).

**Prepare($sql )**

Prepares (compiles) an SQL query for repeated execution. Bind parameters
are denoted by ?, except for the oci8 driver, which uses the traditional Oracle :varname
convention.

Returns an array containing the original sql statement
in the first array element; the remaining elements of the array are driver dependent.
If there is an error, or we are emulating Prepare( ), we return the original
$sql string. This is because all error-handling has been centralized in Execute(
).

Prepare( ) cannot be used with functions that use SQL
query rewriting techniques, e.g. PageExecute( ) and SelectLimit( ).

Example:

```
$stmt = $DB->Prepare('insert into table (col1,col2) values (?,?)');
for ($i=0; $i < $max; $i++)  
	$DB->Execute($stmt,array((string) rand(), $i));
```

Also see InParameter(), OutParameter() and PrepareSP() below. Only supported internally by interbase,
oci8 and selected ODBC-based drivers, otherwise it is emulated. There is no
performance advantage to using Prepare() with emulation.

Important: Due to limitations or bugs in PHP, if you are getting errors when
you using prepared queries, try setting $ADODB\_COUNTRECS = false before preparing.
This behaviour has been observed with ODBC.

**IfNull($field, $nullReplacementValue)**

Portable IFNULL function (NVL in Oracle). Returns a string that represents
the function that checks whether a $field is null for the given database, and
if null, change the value returned to $nullReplacementValue. Eg.

```
$sql = 'SELECT '.$db->IfNull('name', "'- unknown -'"). ' FROM table';
```

**length**

This is not a function, but a property. Some databases have "length" and others "len"
as the function to measure the length of a string. To use this property:

```
  $sql = "SELECT ".$db->length."(field) from table";
  $rs = $db->Execute($sql);
```

**random**

This is not a function, but a property. This is a string that holds the sql to
generate a random number between 0.0 and 1.0 inclusive.

**substr**

This is not a function, but a property. Some databases have "substr" and others "substring"
as the function to retrieve a sub-string. To use this property:

```
  $sql = "SELECT ".$db->substr."(field, $offset, $length) from table";
  $rs = $db->Execute($sql);
```

For all databases, the 1st parameter of *substr* is the field, the 2nd is the
offset (1-based) to the beginning of the sub-string, and the 3rd is the length of the sub-string.

**Param($name)**

Generates a bind placeholder portably. For most databases, the bind placeholder
is "?". However some databases use named bind parameters such as Oracle, eg
":somevar". This allows us to portably define an SQL statement with bind parameters:

```
$sql = 'insert into table (col1,col2) values ('.$DB->Param('a').','.$DB->Param('b').')';
# generates 'insert into table (col1,col2) values (?,?)'
# or        'insert into table (col1,col2) values (:a,:b)'
$stmt = $DB->Prepare($sql);
$stmt = $DB->Execute($stmt,array('one','two'));
```

**PrepareSP****($sql, $cursor=false )**

When calling stored procedures in mssql and oci8 (oracle), and you might want
to directly bind to parameters that return values, or for special LOB handling.
PrepareSP() allows you to do so.

Returns the same array or $sql string as Prepare( ) above. If you do not need
to bind to return values, you should use Prepare( ) instead.

The 2nd parameter, $cursor is not used except with oci8. Setting it to true will
force OCINewCursor to be called; this is to support output REF CURSORs.

For examples of usage of PrepareSP( ), see InParameter( ) below.

Note: in the mssql driver, preparing stored procedures requires a special function
call, mssql\_init( ), which is called by this function. PrepareSP( ) is available
in all other drivers, and is emulated by calling Prepare( ).

**InParameter($stmt, $var, $name,
$maxLen = 4000, $type = false )**

Binds a PHP variable as input to a stored procedure variable. The parameter *$stmt*
is the value returned by PrepareSP(), *$var* is the PHP variable you want to bind, $name
is the name of the stored procedure variable. Optional is *$maxLen*, the maximum length of the
data to bind, and $type which is database dependant.
Consult mssql\_bind and ocibindbyname docs
at php.net for more info on legal values for $type.

InParameter() is a wrapper function that calls Parameter() with $isOutput=false.
The advantage of this function is that it is self-documenting, because
the $isOutput parameter is no longer needed. Only for mssql
and oci8 currently.

Here is an example using oci8:

```
# For oracle, Prepare and PrepareSP are identical
$stmt = $db->PrepareSP(
	"declare RETVAL integer; 
	begin
	:RETVAL := SP_RUNSOMETHING(:myid,:group);
	end;");
$db->InParameter($stmt,$id,'myid');
$db->InParameter($stmt,$group,'group',64);
$db->OutParameter($stmt,$ret,'RETVAL');  
$db->Execute($stmt);
```

The same example using mssql:

```
# @RETVAL = SP_RUNSOMETHING @myid,@group
$stmt = $db->PrepareSP('SP_RUNSOMETHING');   
# note that the parameter name does not have @ in front!
$db->InParameter($stmt,$id,'myid');
$db->InParameter($stmt,$group,'group',64);
# return value in mssql - RETVAL is hard-coded name 
$db->OutParameter($stmt,$ret,'RETVAL');
$db->Execute($stmt);
```

Note that the only difference between the oci8 and mssql implementations is $sql.

If $type parameter is set to false, in mssql, $type will be dynamicly determined
based on the type of the PHP variable passed (string
=> SQLCHAR, boolean =>SQLINT1, integer =>SQLINT4 or float/double=>SQLFLT8).

In oci8, $type can be set to OCI\_B\_FILE (Binary-File), OCI\_B\_CFILE (Character-File),
OCI\_B\_CLOB (Character-LOB), OCI\_B\_BLOB (Binary-LOB) and OCI\_B\_ROWID (ROWID). To
pass in a null, use $db->Parameter($stmt,
$null=null, 'param').

**OutParameter($stmt, $var, $name,
$maxLen = 4000, $type = false )**

Binds a PHP variable as output from a stored procedure variable. The parameter *$stmt*
is the value returned by PrepareSP(), *$var* is the PHP variable you want to bind, *$name*
is the name of the stored procedure variable. Optional is *$maxLen*, the maximum length of the
data to bind, and *$type* which is database dependant.

OutParameter() is a wrapper function that calls Parameter() with $isOutput=true.
The advantage of this function is that it is self-documenting, because
the $isOutput parameter is no longer needed. Only for mssql
and oci8 currently.

For an example, see InParameter.

**Parameter($stmt, $var, $name, $isOutput=false,
$maxLen = 4000, $type = false )**

Note: This function is deprecated, because of the new InParameter() and OutParameter() functions.
These are superior because they are self-documenting, unlike Parameter().

Adds a bind parameter suitable for return values or special data handling (eg.
LOBs) after a statement has been prepared using PrepareSP(). Only for mssql
and oci8 currently. The parameters are:  
  
$***stmt*** Statement returned by Prepare() or PrepareSP().  
$***var*** PHP variable to bind to. Make sure you pre-initialize it!  
$***name*** Name of stored procedure variable name to bind to.  
[$***isOutput***] Indicates direction of parameter 0/false=IN 1=OUT
2= IN/OUT. This is ignored in oci8 as this driver auto-detects the direction.  
[$**maxLen**] Maximum length of the parameter variable.  
[$**type**] Consult mssql\_bind and
ocibindbyname docs at php.net for
more info on legal values for type.

Lastly, in oci8, bind parameters can be reused without calling PrepareSP( )
or Parameters again. This is not possible with mssql. An oci8 example:

```
$id = 0; $i = 0;
$stmt = $db->PrepareSP( "update table set val=:i where id=:id");
$db->Parameter($stmt,$id,'id');
$db->Parameter($stmt,$i, 'i');
for ($cnt=0; $cnt < 1000; $cnt++) {
	$id = $cnt;   
	$i = $cnt * $cnt; # works with oci8!
	$db->Execute($stmt);   
}
```

**Bind($stmt, $var, $size=4001, $type=false, $name=false)**

This is a low-level function supported only by the oci8
driver. **Avoid using** unless you only want to support Oracle. The Parameter(
) function is the recommended way to go with bind variables.

Bind( ) allows you to use bind variables in your sql
statement. This binds a PHP variable to a name defined in an Oracle sql statement
that was previously prepared using Prepare(). Oracle named variables begin with
a colon, and ADOdb requires the named variables be called :0, :1, :2, :3, etc.
The first invocation of Bind() will match :0, the second invocation will match
:1, etc. Binding can provide 100% speedups for insert, select and update statements.

The other variables, $size sets the buffer size for data storage, $type is
the optional descriptor type OCI\_B\_FILE (Binary-File), OCI\_B\_CFILE (Character-File),
OCI\_B\_CLOB (Character-LOB), OCI\_B\_BLOB (Binary-LOB) and OCI\_B\_ROWID (ROWID).
Lastly, instead of using the default :0, :1, etc names, you can define your
own bind-name using $name.

The following example shows 3 bind variables being used:
p1, p2 and p3. These variables are bound to :0, :1 and :2.

```
$stmt = $DB->Prepare("insert into table (col0, col1, col2) values (:0, :1, :2)");
$DB->Bind($stmt, $p1);
$DB->Bind($stmt, $p2);
$DB->Bind($stmt, $p3);
for ($i = 0; $i < $max; $i++) { 
   $p1 = ?; $p2 = ?; $p3 = ?;
   $DB->Execute($stmt);
}
```

You can also use named variables:

```
$stmt = $DB->Prepare("insert into table (col0, col1, col2) values (:name0, :name1, :name2)");
$DB->Bind($stmt, $p1, "name0");
$DB->Bind($stmt, $p2, "name1");
$DB->Bind($stmt, $p3, "name2");
for ($i = 0; $i < $max; $i++) { 
   $p1 = ?; $p2 = ?; $p3 = ?;
   $DB->Execute($stmt);
}
```

**LogSQL($enable=true)**

Call this method to install a SQL logging and timing function (using fnExecute).
Then all SQL statements are logged into an adodb\_logsql table in a database. If
the adodb\_logsql table does not exist, ADOdb will create the table if you have
the appropriate permissions. Returns the previous logging value (true for enabled,
false for disabled). Here are samples of the DDL for selected databases:

```
		mysql:
		CREATE TABLE adodb_logsql (
		  created datetime NOT NULL,
		  sql0 varchar(250) NOT NULL,
		  sql1 text NOT NULL,
		  params text NOT NULL,
		  tracer text NOT NULL,
		  timer decimal(16,6) NOT NULL
		)
		
		postgres:
		CREATE TABLE adodb_logsql (
		  created timestamp NOT NULL,
		  sql0 varchar(250) NOT NULL,
		  sql1 text NOT NULL,
		  params text NOT NULL,
		  tracer text NOT NULL,
		  timer decimal(16,6) NOT NULL
		)
		
		mssql:
		CREATE TABLE adodb_logsql (
		  created datetime NOT NULL,
		  sql0 varchar(250) NOT NULL,
		  sql1 varchar(4000) NOT NULL,
		  params varchar(3000) NOT NULL,
		  tracer varchar(500) NOT NULL,
		  timer decimal(16,6) NOT NULL
		)
		
		oci8:
		CREATE TABLE adodb_logsql (
		  created date NOT NULL,
		  sql0 varchar(250) NOT NULL,
		  sql1 varchar(4000) NOT NULL,
		  params varchar(4000),
		  tracer varchar(4000),
		  timer decimal(16,6) NOT NULL
		)
```

Usage:

```
	$conn->LogSQL(); // turn on logging
	  :
	$conn->Execute(...);
	  :
	$conn->LogSQL(false); // turn off logging
	
	# output summary of SQL logging results
	$perf = NewPerfMonitor($conn);
	echo $perf->SuspiciousSQL();
	echo $perf->ExpensiveSQL();
```

One limitation of logging is that rollback also prevents SQL from being logged.

If you prefer to use another name for the table used to store the SQL, you can override it by calling
adodb\_perf::table($tablename), where $tablename is the new table name (you will still need to manually
create the table yourself). An example:

```
	include('adodb.inc.php');
	include('adodb-perf.inc.php');
	adodb_perf::table('my_logsql_table');
```

Also see Performance Monitor.

**fnExecute and fnCacheExecute properties**

These two properties allow you to define bottleneck functions for all sql statements
processed by ADOdb. This allows you to perform statistical analysis and query-rewriting
of your sql.

**Examples of fnExecute**

Here is an example of using fnExecute, to count all cached queries and non-cached
queries, you can do this:

```
# $db is the connection object
function CountExecs($db, $sql, $inputarray)
{
global $EXECS;

if (!is_array(inputarray)) $EXECS++;
# handle 2-dimensional input arrays
else if (is_array(reset($inputarray))) $EXECS += sizeof($inputarray);
else $EXECS++;
}

# $db is the connection object
function CountCachedExecs($db, $secs2cache, $sql, $inputarray)
{  
global $CACHED; $CACHED++;
}
  
$db = NewADOConnection('mysql');
$db->Connect(...);
$db->fnExecute = 'CountExecs';
$db->fnCacheExecute = 'CountCachedExecs';
 :
 :  
# After many sql statements:`
printf("<p>Total queries=%d; total cached=%d</p>",$EXECS+$CACHED, $CACHED);
```

The fnExecute function is called before the sql is parsed and executed, so
you can perform a query rewrite. If you are passing in a prepared statement,
then $sql is an array (see Prepare). The fnCacheExecute
function is only called if the recordset returned was cached.
The function parameters match the Execute and CacheExecute functions respectively,
except that $this (the connection object) is passed as the first parameter.

Since ADOdb 3.91, the behaviour of fnExecute varies depending on whether the
defined function returns a value. If it does not return a value, then the $sql
is executed as before. This is useful for query rewriting or counting sql queries.

On the other hand, you might want to replace the Execute function with one
of your own design. If this is the case, then have your function return a value.
If a value is returned, that value is returned immediately, without any further
processing. This is used internally by ADOdb to implement LogSQL() functionality.

---

### ADOConnection Utility Functions

**BlankRecordSet([$queryid])**

No longer available - removed since 1.99.

**Concat($s1,$s2,....)**

Generates the sql string used to concatenate $s1, $s2, etc together. Uses the
string in the concat\_operator field to generate the concatenation. Override
this function if a concatenation operator is not used, eg. MySQL.

Returns the concatenated string.

**DBDate($date)**

Format the $**date** in the format the database accepts. This is used in
INSERT/UPDATE statements; for SELECT statements, use SQLDate.
The $**date** parameter can be a Unix integer timestamp or an ISO format
Y-m-d. Uses the fmtDate field, which holds the format to use. If null or false
or '' is passed in, it will be converted to an SQL null.

Returns the date as a quoted string.

**DBTimeStamp($ts)**

Format the timestamp $**ts** in the format the database accepts; this can
be a Unix integer timestamp or an ISO format Y-m-d H:i:s. Uses the fmtTimeStamp
field, which holds the format to use. If null or false or '' is passed in, it
will be converted to an SQL null.

Returns the timestamp as a quoted string.

**qstr($s,[$magic\_quotes\_enabled**=false]**)**

Quotes a string to be sent to the database. The $**magic\_quotes\_enabled**
parameter may look funny, but the idea is if you are quoting a string extracted
from a POST/GET variable, then pass get\_magic\_quotes\_gpc() as the second parameter.
This will ensure that the variable is not quoted twice, once by *qstr*
and once by the *magic\_quotes\_gpc*.

Eg. $s = $db->qstr(HTTP\_GET\_VARS['name'],get\_magic\_quotes\_gpc());

Returns the quoted string.

**Quote($s)**

Quotes the string $s, escaping the database specific quote character as appropriate.
Formerly checked magic quotes setting, but this was disabled since 3.31 for
compatibility with PEAR DB.

**Affected\_Rows( )**

Returns the number of rows affected by a update or delete statement. Returns
false if function not supported.

Not supported by interbase/firebird currently.

**Insert\_ID( )**

Returns the last autonumbering ID inserted. Returns false if function not supported.

Only supported by databases that support auto-increment or object id's, such
as PostgreSQL, MySQL and MS SQL Server currently. PostgreSQL returns the OID, which
can change on a database reload.

**RowLock($table,$where)**

Lock a table row for the duration of a transaction. For example to lock record $id in table1:

```
	$DB->StartTrans();
	$DB->RowLock("table1","rowid=$id");
	$DB->Execute($sql1);
	$DB->Execute($sql2);
	$DB->CompleteTrans();
```

Supported in db2, interbase, informix, mssql, oci8, postgres, sybase.

**MetaDatabases()**

Returns a list of databases available on the server as an array. You have to
connect to the server first. Only available for ODBC, MySQL and ADO.

**MetaTables($ttype = false, $showSchema = false,
$mask=false)**

Returns an array of tables and views for the current database as an array.
The array should exclude system catalog tables if possible. To only show tables,
use $db->MetaTables('TABLES'). To show only views, use $db->MetaTables('VIEWS').
The $showSchema parameter currently works only for DB2, and when set to true,
will add the schema name to the table, eg. "SCHEMA.TABLE".

You can define a mask for matching. For example, setting $mask = 'TMP%' will
match all tables that begin with 'TMP'. Currently only mssql, oci8, odbc\_mssql
and postgres\* support $mask.

**MetaColumns($table,$toupper=true)**

Returns an array of ADOFieldObject's, one field object for every column of
$table. A field object is a class instance with (name, type, max\_length) defined.
Currently Sybase does not recognise date types, and ADO cannot identify
the correct data type (so we default to varchar).

The $toupper parameter determines whether we uppercase the table name
(required for some databases).

For schema support, pass in the $table parameter, "$schema.$tablename". This is only
supported for selected databases.

**MetaColumnNames($table,$numericIndex=false)**

Returns an array of column names for $table. Since ADOdb 4.22, this is an associative array, with the
keys in uppercase. Set $numericIndex=true if you want the old behaviour of numeric indexes (since 4.23).

e.g. array('FIELD1' => 'Field1', 'FIELD2'=>'Field2')

**MetaPrimaryKeys($table,
$owner=false)**

Returns an array containing column names that are the
primary keys of $table. Supported by mysql, odbc (including db2, odbc\_mssql,
etc), mssql, postgres, interbase/firebird, oci8 currently. 

Views (and some tables) have primary keys, but sometimes this information is not available from the
database. You can define a function ADODB\_View\_PrimaryKeys($databaseType, $database, $view, $owner) that
should return an array containing the fields that make up the primary key. If that function exists,
it will be called when MetaPrimaryKeys() cannot find a primary key for a table or view.

```
// In this example: dbtype = 'oci8', $db = 'mydb', $view = 'dataView', $owner = false 
function ADODB_View_PrimaryKeys($dbtype,$db,$view,$owner)
{
	switch(strtoupper($view)) {
	case 'DATAVIEW': return array('DATAID');
	default: return false;
	}
}

$db = NewADOConnection('oci8');
$db->Connect('localhost','root','','mydb'); 
$db->MetaPrimaryKeys('dataView');
```

**ServerInfo($table)**

Returns an array of containing two elements 'description'
and 'version'. The 'description' element contains the string description of
the database. The 'version' naturally holds the version number (which is also
a string).

**MetaForeignKeys($table, $owner=false, $upper=false)**

Returns an associate array of foreign keys, or false if not supported. For
example, if table employee has a foreign key where employee.deptkey points to
dept\_table.deptid, and employee.posn=posn\_table.postionid and employee.poscategory=posn\_table.category,
then $conn->MetaForeignKeys('employee') will return

```
	array(
		'dept_table' => array('deptkey=deptid'),
		'posn_table' => array('posn=positionid','poscategory=category')
	)
```

The optional schema or owner can be defined in $owner. If $upper is true, then
the table names (array keys) are upper-cased.

---

## ADORecordSet

When an SQL statement successfully is executed by ADOConnection->Execute($sql),an
ADORecordSet object is returned. This object contains a virtual cursor so we
can move from row to row, functions to obtain information about the columns
and column types, and helper functions to deal with formating the results to
show to the user.

### ADORecordSet Fields

**fields:** Array containing the current row. This is not associative, but
is an indexed array from 0 to columns-1. See also the function **Fields**,
which behaves like an associative array.

**dataProvider**: The underlying mechanism used to connect to the database.
Normally set to **native**, unless using **odbc** or **ado**.

**blobSize**: Maximum size of a char, string or varchar object before it
is treated as a Blob (Blob's should be shown with textarea's). See the MetaType
function.

**sql**: Holds the sql statement used to generate this record set.

**canSeek**: Set to true if Move( ) function works.

**EOF**: True if we have scrolled the cursor past the last record.

### ADORecordSet Functions

**ADORecordSet( )**

Constructer. Normally you never call this function yourself.

**GetAssoc([$force\_array])**

Generates an associative array from the recordset. Note that is this function
is also available in the connection object. More details
can be found there.

 

**GetArray([$number\_of\_rows])**

Generate a 2-dimensional array of records from the current cursor position,
indexed from 0 to $number\_of\_rows - 1. If $number\_of\_rows is undefined, till
EOF.

**GetRows([$number\_of\_rows])**

Generate a 2-dimensional array of records from the current cursor position. Synonym
for GetArray() for compatibility with Microsoft ADO.

**GetMenu($name, [$default\_str=''], [$blank1stItem=true],
[$multiple\_select=false], [$size=0], [$moreAttr=''])**

Generate a HTML menu (<select><option><option></select>).
The first column of the recordset (fields[0]) will hold the string to display
in the option tags. If the recordset has more than 1 column, the second column
(fields[1]) is the value to send back to the web server.. The menu will be given
the name $*name*.

If $*default\_str* is defined, then if $*default\_str* == fields[0],
that field is selected. If $*blank1stItem* is true, the first option is
empty. You can also set the first option strings by setting $blank1stItem =
"$value:$text".

$*Default\_str* can be array for a multiple select listbox.

To get a listbox, set the $*size* to a non-zero value (or pass $default\_str
as an array). If $*multiple\_select* is true then a listbox will be generated
with $*size* items (or if $size==0, then 5 items) visible, and we will
return an array to a server. Lastly use $*moreAttr*  to add additional
attributes such as javascript or styles.

Menu Example 1: `GetMenu('menu1','A',true)` will generate a menu:

A
B
C
for the data (A,1), (B,2), (C,3). Also see example 5.

Menu Example 2: For the same data, `GetMenu('menu1',array('A','B'),false)`
will generate a menu with both A and B selected:   

A
B
C

**GetMenu2($name, [$default\_str=''], [$blank1stItem=true],
[$multiple\_select=false], [$size=0], [$moreAttr=''])**

This is nearly identical to GetMenu, except that the $*default\_str* is
matched to fields[1] (the option values).

Menu Example 3: Given the data in menu example 2, `GetMenu2('menu1',array('1','2'),false)`
will generate a menu with both A and B selected in menu example 2, but this
time the selection is based on the 2nd column, which holds the values to return
to the Web server.

**UserDate($str, [$fmt])**

Converts the date string $*str* to another format. The date format is Y-m-d,
or Unix timestamp format. The default $*fmt* is Y-m-d.

**UserTimeStamp($str, [$fmt])**

Converts the timestamp string $**str** to another format. The timestamp
format is Y-m-d H:i:s, as in '2002-02-28 23:00:12', or Unix timestamp format.
UserTimeStamp calls UnixTimeStamp to parse $*str*, and $*fmt* defaults to Y-m-d H:i:s if not defined.

**UnixDate($str)**

Parses the date string $**str** and returns it in unix mktime format (eg.
a number indicating the seconds after January 1st, 1970). Expects the date to
be in Y-m-d H:i:s format, except for Sybase and Microsoft SQL Server, where
M d Y is also accepted (the 3 letter month strings are controlled by a global
array, which might need localisation).

This function is available in both ADORecordSet and ADOConnection since 1.91.

**UnixTimeStamp($str)**

Parses the timestamp string $**str** and returns it in unix mktime format
(eg. a number indicating the seconds after January 1st, 1970). Expects the date
to be in "Y-m-d, H:i:s" (1970-12-24, 00:00:00) or "Y-m-d H:i:s" (1970-12-24 00:00:00) or "YmdHis" (19701225000000) format, except for Sybase and Microsoft SQL Server, where
"M d Y h:i:sA" (Dec 25 1970 00:00:00AM) is also accepted (the 3 letter month strings are controlled by
a global array, which might need localisation).

This function is available in both ADORecordSet and ADOConnection
since 1.91.

**OffsetDate($dayFraction,
$basedate=false)**

Returns a string with the
native SQL functions to calculate future and past dates based on $basedate in
a portable fashion. If $basedate is not defined, then the current date (at 12
midnight) is used. Returns the SQL string that performs the calculation when
passed to Execute().

For example, in Oracle, to find the date and time that
is 2.5 days from today, you can use:

```
# get date one week from now
$fld = $conn->OffsetDate(7); // returns "(trunc(sysdate)+7")
```

```
# get date and time that is 60 hours from current date and time
$fld = $conn->OffsetDate(2.5, $conn->sysTimeStamp);	// returns "(sysdate+2.5)"

$conn->Execute("UPDATE TABLE SET dodate=$fld WHERE ID=$id");
```

This function is available for mysql, mssql, oracle, oci8 and postgresql drivers
since 2.13. It might work with other drivers provided
they allow performing numeric day arithmetic on dates.

**SQLDate($dateFormat, $basedate=false)**

Returns a string which contains the native SQL functions to format a date or date
column $basedate. This is used in SELECT statements. For INSERT/UPDATE statements,
use DBDate. It uses a case-sensitive $dateFormat, which
supports:

```
 Y: 4-digit Year
 Q: Quarter (1-4)
 M: Month (Jan-Dec)
 m: Month (01-12)
 d: Day (01-31)
 H: Hour (00-23)
 h: Hour (1-12)
 i: Minute (00-59)
 s: Second (00-60)
 A: AM/PM indicator
```

All other characters are treated as strings. You can also use \ to escape characters.
Available on selected databases, including mysql, postgresql, mssql, oci8 and
DB2.

This is useful in writing portable sql statements that GROUP BY on dates. For
example to display total cost of goods sold broken by quarter (dates are stored
in a field called postdate):

```
 $sqlfn = $db->SQLDate('Y-\QQ','postdate'); # get sql that formats postdate to output 2002-Q1
 $sql = "SELECT $sqlfn,SUM(cogs) FROM table GROUP BY $sqlfn ORDER BY 1 desc";
```

**MoveNext( )**

Move the internal cursor to the next row. The *$this->fields* array is
automatically updated. Returns false if unable to do so (normally because EOF
has been reached), otherwise true.

If EOF is reached, then the $this->fields array is set to false (this was
only implemented consistently in ADOdb 3.30). For the pre-3.30 behaviour of
$this->fields (at EOF), set the global variable $ADODB\_COMPAT\_FETCH = true.

Example:

```
$rs = $db->Execute($sql);
if ($rs) 
	while (!$rs->EOF) {
 		ProcessArray($rs->fields);	
		$rs->MoveNext();
	}
```

**Move($to)**

Moves the internal cursor to a specific row $**to**. Rows are zero-based
eg. 0 is the first row. The **fields** array is automatically updated. For
databases that do not support scrolling internally, ADOdb will simulate forward
scrolling. Some databases do not support backward scrolling. If the $**to**
position is after the EOF, $**to** will move to the end of the RecordSet
for most databases. Some obscure databases using odbc might not behave this
way.

Note: This function uses *absolute positioning*, unlike Microsoft's ADO.

Returns true or false. If false, the internal cursor is not moved in most implementations,
so AbsolutePosition( ) will return the last cursor position before the Move(
).

**MoveFirst()**

Internally calls Move(0). Note that some databases do not support this function.

**MoveLast()**

Internally calls Move(RecordCount()-1). Note that some databases do not support
this function.

**GetRowAssoc**($toUpper=true)

Returns an associative array containing the current row. The keys to the array
are the column names. The column names are upper-cased for easy access. To get
the next row, you will still need to call MoveNext().

For example:  
Array ( [ID] => 1 [FIRSTNAME] => Caroline [LASTNAME] => Miranda [CREATED] =>
2001-07-05 )

Note: do not use GetRowAssoc() with $ADODB\_FETCH\_MODE = ADODB\_FETCH\_ASSOC.
Because they have the same functionality, they will interfere with each other.

**AbsolutePage($page=-1)**

Returns the current page. Requires PageExecute()/CachePageExecute() to be called.
See Example 8.

**AtFirstPage($status='')**

Returns true if at first page (1-based). Requires PageExecute()/CachePageExecute()
to be called. See Example 8.

**AtLastPage($status='')**

Returns true if at last page (1-based). Requires PageExecute()/CachePageExecute()
to be called. See Example 8.

**Fields**(**$colname**)

Returns the value of the
associated column $**colname** for the current row. The column name is case-insensitive.

This is a convenience function. For higher performance, use $ADODB\_FETCH\_MODE.

**FetchRow**()

Returns array containing current row, or false if EOF.
FetchRow( ) internally moves to the next record after returning the current
row.

Warning: Do not mix using FetchRow() with MoveNext().

Usage:

```
$rs = $db->Execute($sql);
if ($rs)
	while ($arr = $rs->FetchRow()) {
	     # process $arr	
	}
```

**FetchInto**(**&$array**)

Sets $array to the current row. Returns PEAR\_Error object
if EOF, 1 if ok (DB\_OK constant). If PEAR is undefined, false is returned when
EOF. FetchInto( ) internally moves to the next
record after returning the current row.

FetchRow() is easier to use. See above.

**FetchField($column\_number)**

Returns an object containing the **name**, **type** and **max\_length**
of the associated field. If the max\_length cannot be determined reliably, it
will be set to -1. The column numbers are zero-based. See example
2.

**FieldCount( )**

Returns the number of fields (columns) in the record set.

**RecordCount( )**

Returns the number of rows in the record set. If the number of records returned
cannot be determined from the database driver API, we will buffer all rows and
return a count of the rows after all the records have been retrieved. This buffering
can be disabled (for performance reasons) by setting the global variable $ADODB\_COUNTRECS
= false. When disabled, RecordCount( ) will return -1 for certain databases.
See the supported databases list above for more details.

RowCount is a synonym for RecordCount.

**PO\_RecordCount($table, $where)**

Returns the number of rows in the record set. If the database does not support
this, it will perform a SELECT COUNT(\*) on the table $table, with the given
$where condition to return an estimate of the recordset size.

$numrows = $rs->PO\_RecordCount("articles\_table", "group=$group");

 **NextRecordSet()**

For databases that allow multiple recordsets to be returned in one query, this
function allows you to switch to the next recordset. Currently only supported
by mssql driver.

```
$rs = $db->Execute('execute return_multiple_rs');
$arr1 = $rs->GetArray();
$rs->NextRecordSet();
$arr2 = $rs->GetArray();
```

**FetchObject($toupper=true)**

Returns the current row as an object. If you set $toupper to true, then the
object fields are set to upper-case. Note: The newer FetchNextObject() is the
recommended way of accessing rows as objects. See below.

**FetchNextObject($toupper=true)**

Gets the current row as an object and moves to the next row automatically.
Returns false if at end-of-file. If you set $toupper to true, then the object
fields are set to upper-case.

```
$rs = $db->Execute('select firstname,lastname from table');
if ($rs) {
	while ($o = $rs->FetchNextObject()) {
		print "$o->FIRSTNAME, $o->LASTNAME<BR>";
	}
}
```

There is some trade-off in speed in using FetchNextObject(). If performance
is important, you should access rows with the `fields[]` array. **FetchObj()**

Returns the current record as an object. Fields are not upper-cased, unlike
FetchObject.

**FetchNextObj()**

Returns the current record as an object and moves to
the next record. If EOF, false is returned. Fields are not upper-cased, unlike
FetctNextObject.

**CurrentRow( )**

Returns the current row of the record set. 0 is the first row.

**AbsolutePosition( )**

Synonym for **CurrentRow** for compatibility with ADO. Returns the current
row of the record set. 0 is the first row.

**MetaType($nativeDBType[,$field\_max\_length],[$fieldobj])**

Determine what *generic* meta type a database field type is given its
native type $**nativeDBType** as a string and the length of the field $**field\_max\_length**.
Note that field\_max\_length can be -1 if it is not known. The field object returned
by FetchField() can be passed in $**fieldobj** or as the 1st parameter **$nativeDBType**.
This is useful for databases such as *mysql* which has additional properties
in the field object such as *primary\_key*.

Uses the field **blobSize** and compares it with $**field\_max\_length**
to determine whether the character field is actually a blob.

For example, $db->MetaType('char') will return 'C'.

Returns:

- **C**: Character fields that should be shown in a <input type="text">
  tag.
- **X**: Clob (character large objects), or large text fields that should
  be shown in a <textarea>
- **D**: Date field
- **T**: Timestamp field
- **L**: Logical field (boolean or bit-field)
- **N**: Numeric field. Includes decimal, numeric, floating point, and
  real.
- **I**:  Integer field.
- **R**: Counter or Autoincrement field. Must be numeric.
- **B**: Blob, or binary large objects.

Since ADOdb 3.0, MetaType accepts $fieldobj as the first
parameter, instead of $nativeDBType.

**Close( )**

Closes the recordset, cleaning all memory and resources associated with the recordset.

If memory management is not an issue, you do not need to call this function as recordsets
are closed for you by PHP at the end of the script.
SQL statements such as INSERT/UPDATE/DELETE do not really return a recordset, so you do not have to call Close()
for such SQL statements.

---

### function rs2html($adorecordset,[$tableheader\_attributes], [$col\_titles])

This is a standalone function (rs2html = recordset to html) that is similar
to PHP's *odbc\_result\_all* function, it prints a ADORecordSet, $**adorecordset**
as a HTML table. $**tableheader\_attributes** allow you to control the table
*cellpadding*, *cellspacing* and *border* attributes. Lastly
you can replace the database column names with your own column titles with the
array $**col\_titles**. This is designed more as a quick debugging mechanism,
not a production table recordset viewer.

You will need to include the file *tohtml.inc.php*.

Example of rs2html:

```
<?
include('tohtml.inc.php'); # load code common to ADOdb 
include('adodb.inc.php'); # load code common to ADOdb 
$conn = &ADONewConnection('mysql');   # create a connection 
$conn->PConnect('localhost','userid','','agora');# connect to MySQL, agora db
$sql = 'select CustomerName, CustomerID from customers'; 
$rs   = $conn->Execute($sql); 
rs2html($rs,'border=2 cellpadding=3',array('Customer Name','Customer ID'));
?>
```

---

### Differences between this ADOdb library and Microsoft ADO

1. ADOdb only supports recordsets created by a connection object. Recordsets
   cannot be created independently.
2. ADO properties are implemented as functions in ADOdb. This makes it easier
   to implement any enhanced ADO functionality in the future.
3. ADOdb's ADORecordSet->Move()
   uses absolute positioning, not relative. Bookmarks are not supported.
4. ADORecordSet->AbsolutePosition()
   cannot be used to move the record cursor.
5. ADO Parameter objects are not supported. Instead we have the ADOConnection::Parameter(
   ) function, which provides a simpler interface for calling preparing parameters
   and calling stored procedures.
6. Recordset properties for paging records are available, but implemented as
   in Example 8.

---

# Database Driver Guide

This describes how to create a class to connect to a new database. To ensure
there is no duplication of work, kindly email me at jlim#natsoft.com.my if you
decide to create such a class.

First decide on a name in lower case to call the database type. Let's say we
call it xbase.

Then we need to create two classes ADODB\_xbase and ADORecordSet\_xbase in the
file adodb-xbase.inc.php.

The simplest form of database driver is an adaptation of an existing ODBC driver.
Then we just need to create the class *ADODB\_xbase extends ADODB\_odbc*
to support the new **date** and **timestamp** formats, the **concatenation**
operator used, **true** and **false**. For the *ADORecordSet\_xbase extends
ADORecordSet\_odbc* we need to change the **MetaType** function. See**adodb-vfp.inc.php** as an example.

More complicated is a totally new database driver that connects to a new PHP
extension. Then you will need to implement several functions. Fortunately, you
do not have to modify most of the complex code. You only need to override a
few stub functions. See **adodb-mysql.inc.php** for example.

The default date format of ADOdb internally is YYYY-MM-DD (Ansi-92). All dates
should be converted to that format when passing to an ADOdb date function. See
Oracle for an example how we use ALTER SESSION to change the default date format
in \_pconnect \_connect.

**ADOConnection Functions to Override**

Defining a constructor for your ADOConnection derived function is optional.
There is no need to call the base class constructor.

\_**connect**: Low level implementation of Connect. Returns true or false.
Should set the \_**connectionID**.

\_**pconnect:** Low level implemention of PConnect. Returns true or false.
Should set the \_**connectionID**.

\_**query**: Execute a query. Returns the queryID, or false.

\_**close:** Close the connection -- PHP should clean up all recordsets.

**ErrorMsg**: Stores the error message in the private variable \_errorMsg.

**ADOConnection Fields to Set**

\_**bindInputArray**: Set to true if binding of parameters for SQL inserts
and updates is allowed using ?, eg. as with ODBC.

**fmtDate**

**fmtTimeStamp**

**true**

**false**

**concat\_operator**

**replaceQuote**

**hasLimit** support SELECT \* FROM TABLE LIMIT 10 of MySQL.

**hasTop** support Microsoft style SELECT TOP 10 \* FROM TABLE.

**ADORecordSet Functions to Override**

You will need to define a constructor for your ADORecordSet derived class that
calls the parent class constructor.

**FetchField:**  as documented above in ADORecordSet

\_**initrs**: low level initialization of the recordset: setup the \_**numOfRows**
and \_**numOfFields** fields -- called by the constructor.

\_**seek**: seek to a particular row. Do not load the data into the fields
array. This is done by \_fetch. Returns true or false. Note that some implementations
such as Interbase do not support seek. Set canSeek to false.

\_**fetch**: fetch a row using the database extension function and then move
to the next row. Sets the **fields** array. If the parameter $ignore\_fields
is true then there is no need to populate the **fields** array, just move
to the next row. then Returns true or false.

\_**close**: close the recordset

**Fields**: If the array row returned by the PHP extension is not an associative
one, you will have to override this. See adodb-odbc.inc.php for an example.
For databases such as MySQL and MSSQL where an associative array is returned,
there is no need to override this function.

**ADOConnection Fields to Set**

canSeek: Set to true if the \_seek function works.

## ToDo:

See the RoadMap article.

Also see the ADOdb proxy article
for bridging Windows and Unix databases using http remote procedure calls. For
your education, visit palslib.com for database info,
and read this article on Optimizing
PHP.

## Change Log

**4.52 ?? 2004**

Bug found in Replace() when performance logging enabled, introduced in ADOdb 4.50. Fixed.

Replace() checks update stmt. If update stmt fails, we now return immediately. Thx to alex.

Added support for $ADODB\_FORCE\_TYPE in GetUpdateSQL/GetInsertSQL. Thx to niko.

Added ADODB\_ASSOC\_CASE support to postgres/postgres7 driver.

**4.51 29 July 2004**

Added adodb-xmlschema 1.0.2. Thx dan and richard.

Added new adorecordset\_ext\_\* classes. If ADOdb extension installed for mysql, mysqlt and oci8
(but not oci8po), we use the superfast ADOdb extension code for movenext.

Added schema support to mssql and odbc\_mssql MetaPrimaryKeys().

Patched MSSQL driver to support PHP NULL and Boolean values
while binding the input array parameters in the \_query() function. By Stephen Farmer.

Added support for clob's for mssql, UpdateBlob(). Thx to gfran#directa.com.br

Added normalize support for postgresql (true=lowercase table name, or false=case-sensitive table names)
to MetaColumns($table, $normalize=true).

PHP5 variant dates in ADO not working. Fixed in adodb-ado.inc.php.

Constant ADODB\_FORCE\_NULLS was not working properly for many releases (for GetUpdateSQL). Fixed.
Also GetUpdateSQL strips off ORDER BY now - thx Elieser Le�o.

Perf Monitor for oci8 now dynamically highlights optimizer\_\* params if too high/low.

Added dsn support to NewADOConnection/ADONewConnection.

Fixed out of page bounds bug in \_adodb\_pageexecute\_all\_rows() Thx to "Sergio Strampelli" sergio#rir.it

Speedup of movenext for mysql and oci8 drivers.

Moved debugging code \_adodb\_debug\_execute() to adodb-lib.inc.php.

Fixed postgresql bytea detection bug. See http://phplens.com/lens/lensforum/msgs.php?id=9849.

Fixed ibase datetimestamp typo in PHP5. Thx stefan.

Removed whitespace at end of odbtp drivers.

Added db2 metaprimarykeys fix.

Optimizations to MoveNext() for mysql and oci8. Misc speedups to Get\* functions.

**4.50 6 July 2004**

Bumped it to 4.50 to avoid confusion with PHP 4.3.x series.

Added db2 metatables and metacolumns extensions.

Added alpha PDO driver. Very buggy, only works with odbc.

Tested mysqli. Set poorAffectedRows = true. Cleaned up movenext() and \_fetch().

PageExecute does not work properly with php5 (return val not a variable). Reported Dmytro Sychevsky sych#php.com.ua. Fixed.

MetaTables() for mysql, $showschema parameter was not backward compatible with older versions of adodb. Fixed.

Changed mysql GetOne() to work with mysql 3.23 when using with non-select stmts (e.g. SHOW TABLES).

Changed TRIG\_ prefix to a variable in datadict-oci8.inc.php. Thx to Luca.Gioppo#csi.it.

New to adodb-time code. We allow you to define your own daylights savings function,
adodb\_daylight\_sv for pre-1970 dates. If the function is defined
(somewhere in an include), then you can correct
for daylights savings. See http://phplens.com/phpeverywhere/node/view/16#daylightsavings
for more info.

New sqlitepo driver. This is because assoc mode does not work like other drivers in sqlite.
Namely, when selecting (joining) multiple tables, in assoc mode the table
names are included in the assoc keys in the "sqlite" driver.
In "sqlitepo" driver, the table names are stripped from the returned column names.
When this results in a conflict, the first field get preference.
Contributed by Herman Kuiper herman#ozuzo.net

Added $forcenull parameter to GetInsertSQL/GetUpdateSQL. Idea by Marco Aurelio Silva.

More XHTML changes for GetMenu. By Jeremy Evans.

Fixes some ibase date issues. Thx to stefan bogdan.

Improvements to mysqli driver to support $ADODB\_COUNTRECS.

Fixed adodb-csvlib.inc.php problem when reading stream from socket. We need to poll stream continiously.

**4.23 16 June 2004**

New interbase/firebird fixes thx to Lester Caine.
Driver fixes a problem with getting field names in the result array, and
corrects a couple of data conversions. Also we default to dialect3 for firebird.
Also ibase sysDate property was wrong. Changed to cast as timestamp.

The datadict driver is set up to give quoted tables and fields as this
was the only way round reserved words being used as field names in
TikiWiki. TikiPro is tidying that up, and I hope to be able to produce a
build of THAT which uses what I consider proper UPPERCASE field and
table names. The conversion of TikiWiki to ADOdb helped in that, but
until the database is completely tidied up in TikiPro ...

Modified \_gencachename() to include fetchmode in name hash.
This means you should clear your cache directory after installing this release as the
cache name algorithm has changed.

Now Cache\* functions work in safe mode, because we do not create sub-directories in the $ADODB\_CACHE\_DIR in safe mode. In non-safe mode we still create sub-directories. Done by modifying \_gencachename().

Added $gmt parameter (true/false) to UserDate and UserTimeStamp in connection class, to force conversion of input (in local time) to be converted to UTC/GMT.

Mssql datadict did not support INT types properly (no size param allowed).
Added \_GetSize() to datadict-mssql.inc.php.

For borland\_ibase, BeginTrans(), changed:  

```
   $this->_transactionID = $this->_connectionID;
```

to  

```
   $this->_transactionID = ibase_trans($this->ibasetrans, $this->_connectionID);
```

Fixed typo in mysqi\_field\_seek(). Thx to Sh4dow (sh4dow#php.pl).

LogSQL did not work with Firebird/Interbase. Fixed.

Postgres: made errorno() handling more consistent. Thx to Michael Jahn, Michael.Jahn#mailbox.tu-dresden.de.

Added informix patch to better support metatables, metacolumns by "Cecilio Albero" c-albero#eos-i.com

Cyril Malevanov contributed patch to oci8 to support passing of LOB parameters:

```
	$text = 'test test test';
	$sql = "declare rs clob; begin :rs := lobinout(:sa0); end;";
	$stmt = $conn -> PrepareSP($sql);
	$conn -> InParameter($stmt,$text,'sa0', -1, OCI_B_CLOB);
	$rs = '';
	$conn -> OutParameter($stmt,$rs,'rs', -1, OCI_B_CLOB);
	$conn -> Execute($stmt);
	echo "return = ".$rs."<br>";
```

As he says, the LOBs limitations are:

```
 - use OCINewDescriptor before binding
 - if Param is IN, uses save() before each execute. This is done automatically for you.
 - if Param is OUT, uses load() after each execute. This is done automatically for you.
 - when we bind $var as LOB, we create new descriptor and return it as a
   Bind Result, so if we want to use OUT parameters, we have to store
   somewhere &$var to load() data from LOB to it.
 - IN OUT params are not working now (should not be a big problem to fix it)
 - now mass binding not working too (I've wrote about it before)
```

Simplified Connect() and PConnect() error handling.

When extension not loaded, Connect() and PConnect() will return null. On connect error, the fns will return false.

CacheGetArray() added to code.

Added Init() to adorecordset\_empty().

Changed postgres64 driver, MetaColumns() to not strip off quotes in default value if :: detected (type-casting of default).

Added test: if (!defined('ADODB\_DIR')) die(). Useful to prevent hackers from detecting file paths.

Changed metaTablesSQL to ignore Postgres 7.4 information schemas (sql\_\*).

New polish language file by Grzegorz Pacan

Added support for UNION in \_adodb\_getcount().

Added security check for ADODB\_DIR to limit path disclosure issues. Requested by postnuke team.

Added better error message support to oracle driver. Thx to Gaetano Giunta.

Added showSchema support to mysql.

Bind in oci8 did not handle $name=false properly. Fixed.

If extension not loaded, Connect(), PConnect(), NConnect() will return null.

**4.22 15 Apr 2004**

Moved docs to own adodb/docs folder.

Fixed session bug when quoting compressed/encrypted data in Replace().

Netezza Driver and LDAP drivers contributed by Josh Eldridge.

GetMenu now uses rtrim() on values instead of trim().

Changed MetaColumnNames to return an associative array, keys being the field names in uppercase.

Suggested fix to adodb-ado.inc.php affected\_rows to support PHP5 variants. Thx to Alexios Fakos.

Contributed bulgarian language file by Valentin Sheiretsky valio#valio.eu.org.

Contributed romanian language file by stefan bogdan.

GetInsertSQL now checks for table name (string) in $rs, and will create a recordset for that
table automatically. Contributed by Walt Boring. Also added OCI\_B\_BLOB in bind on Walt's request - hope
it doesn't break anything :-)

Some minor postgres speedups in \_initrs().

ChangeTableSQL checks now if MetaColumns returns empty. Thx Jason Judge.

Added ADOConnection::Time(), returns current database time in unix timestamp format, or false.

**4.21 20 Mar 2004**

We no longer in SelectLimit for VFP driver add SELECT TOP X unless an ORDER BY exists.

Pim Koeman contributed dutch language file adodb-nl.inc.php.

Rick Hickerson added CLOB support to db2 datadict.

Added odbtp driver. Thx to "stefan bogdan" sbogdan#rsb.ro.

Changed PrepareSP() 2nd parameter, $cursor, to default to true (formerly false). Fixes oci8 backward
compat problems with OUT params.

Fixed month calculation error in adodb-time.inc.php. 2102-June-01 appeared as 2102-May-32.

Updated PHP5 RC1 iterator support. API changed, hasMore() renamed to valid().

Changed internal format of serialized cache recordsets. As we store a version number, this should be
backward compatible.

Error handling when driver file not found was flawed in ADOLoadCode(). Fixed.

**4.20 27 Feb 2004**

Updated to AXMLS 1.01.

MetaForeignKeys for postgres7 modified by Edward Jaramilla, works on pg 7.4.

Now numbers accepts function calls or sequences for GetInsertSQL/GetUpdateSQL numeric fields.

Changed quotes of 'delete from $perf\_table' to "". Thx Kehui (webmaster#kehui.net)

Added ServerInfo() for ifx, and putenv trim fix. Thx Fernando Ortiz.

Added addq(), which is analogous to addslashes().

Tested with php5b4. Fix some php5 compat problems with exceptions and sybase.

Carl-Christian Salvesen added patch to mssql \_query to support binds greater than 4000 chars.

Mike suggested patch to PHP5 exception handler. $errno must be numeric.

Added double quotes (") to ADODB\_TABLE\_REGEX.

For oci8, Prepare(...,$cursor), $cursor's meaning was accidentally inverted in 4.11. This causes problems with ExecuteCursor() too, which calls Prepare() internally. Thx to William Lovaton.

Now dateHasTime property in connection object renamed to datetime for consistency. This could break bc.

Csongor Halmai reports that db2 SelectLimit with input array is not working. Fixed..

**4.11 27 Jan 2004**

Csongor Halmai reports db2 binding not working. Reverted back to emulated binding.

Dan Cech modifies datadict code. Adds support for DropIndex. Minor cleanups.

Table misspelt in perf-oci8.inc.php. Changed v$conn\_cache\_advice to v$db\_cache\_advice. Reported by Steve W.

UserTimeStamp and DBTimeStamp did not handle YYYYMMDDHHMMSS format properly. Reported by Mike Muir. Fixed.

Changed oci8 Prepare(). Does not auto-allocate OCINewCursor automatically, unless 2nd param is set to true.
This will break backward compat, if Prepare/Execute is used instead of ExecuteCursor. Reported by Chris Jones.

Added InParameter() and OutParameter(). Wrapper functions to Parameter(), but nicer because they
are self-documenting.

Added 'R' handling in ActualType() to datadict-mysql.inc.php

Added ADOConnection::SerializableRS($rs). Returns a recordset that can be serialized in a session.

Added "Run SQL" to performance UI().

Misc spelling corrections in adodb-mysqli.inc.php, adodb-oci8.inc.php and datadict-oci8.inc.php, from Heinz Hombergs.

MetaIndexes() for ibase contributed by Heinz Hombergs.

**4.10 12 Jan 2004**

Dan Cech contributed extensive changes to data dictionary to support name quoting (with `), and drop table/index.

Informix added cursorType property. Default remains IFX\_SCROLL, but you can change to 0 (non-scrollable cursor) for performance.

Added ADODB\_View\_PrimaryKeys() for returning view primary keys to MetaPrimaryKeys().

Simplified chinese file, adodb-cn.inc.php from cysoft.

Added check for ctype\_alnum in adodb-datadict.inc.php. Thx to Jason Judge.

Added connection parameter to ibase Prepare(). Fix by Daniel Hassan.

Added nameQuote for quoting identifiers and names to connection obj. Requested by Jason Judge. Also the
data dictionary parser now detects `field name` and generates column names with spaces correctly.

BOOL type not recognised correctly as L. Fixed.

Fixed paths in ADODB\_DIR for session files, and back-ported it to 4.05 (15 Dec 2003)

Added Schema to postgresql MetaTables. Thx to col#gear.hu

Empty postgresql recordsets that had blob fields did not set EOF properly. Fixed.

CacheSelectLimit internal parameters to SelectLimit were wrong. Thx to Nio.

Modified adodb\_pr() and adodb\_backtrace() to support command-line usage (eg. no html).

Fixed some fr and it lang errors. Thx to Gaetano G.

Added contrib directory, with adodb rs to xmlrpc convertor by Gaetano G.

Fixed array recordset bugs when \_skiprow1 is true. Thx to Gaetano G.

Fixed pivot table code when count is false.

**4.05 13 Dec 2003** 

Added MetaIndexes - thx to Dan Cech.

Rewritten session code by Ross Smith. Moved code to adodb/session directory.

Added function exists check on connecting to most drivers, so we don't crash with the unknown function error.

Smart Transactions failed with GenID() when it no seq table has been created because the sql
statement fails. Fix by Mark Newnham.

Added $db->length, which holds name of function that returns strlen.

Fixed error handling for bad driver in ADONewConnection - passed too few params to error-handler.

Datadict did not handle types like 16.0 properly in \_GetSize. Fixed.

Oci8 driver SelectLimit() bug &= instead of =& used. Thx to Swen Th�mmler.

Jesse Mullan suggested not flushing outp when output buffering enabled. Due to Apache 2.0 bug. Added.

MetaTables/MetaColumns return ref bug with PHP5 fixed in adodb-datadict.inc.php.

New mysqli driver contributed by Arjen de Rijke. Based on adodb 3.40 driver.
Then jlim added BeginTrans, CommitTrans, RollbackTrans, IfNull, SQLDate. Also fixed return ref bug.

$ADODB\_FLUSH added, if true then force flush in debugging outp. Default is false. In earlier
versions, outp defaulted to flush, which is not compat with apache 2.0.

Mysql driver's GenID() function did not work when when sql logging is on. Fixed.

$ADODB\_SESSION\_TBL not declared as global var. Not available if adodb-session.inc.php included in function. Fixed.

The input array not passed to Execute() in \_adodb\_getcount(). Fixed.

**4.04 13 Nov 2003** 

Switched back to foreach - faster than list-each.

Fixed bug in ado driver - wiping out $this->fields with date fields.

Performance Monitor, View SQL, Explain Plan did not work if strlen($SQL)>max($\_GET length). Fixed.

Performance monitor, oci8 driver added memory sort ratio.

Added random property, returns SQL to generate a floating point number between 0 and 1;

**4.03 6 Nov 2003** 

The path to adodb-php4.inc.php and adodb-iterators.inc.php was not setup properly.

Patched SQLDate in interbase to support hours/mins/secs. Thx to ari kuorikoski.

Force autorollback for pgsql persistent connections -
apparently pgsql did not autorollback properly before 4.3.4. See http://bugs.php.net/bug.php?id=25404

**4.02 5 Nov 2003** 

Some errors in adodb\_error\_pg() fixed. Thx to Styve.

Spurious Insert\_ID() error was generated by LogSQL(). Fixed.

Insert\_ID was interfering with Affected\_Rows() and Replace() when LogSQL() enabled. Fixed.

More foreach loops optimized with list/each.

Null dates not handled properly in ADO driver (it becomes 31 Dec 1969!).

Heinz Hombergs contributed patches for mysql MetaColumns - adding scale, made
interbase MetaColumns work with firebird/interbase, and added lang/adodb-de.inc.php.

Added INFORMIXSERVER environment variable.

Added $ADODB\_ANSI\_PADDING\_OFF for interbase/firebird.

PHP 5 beta 2 compat check. Foreach (Iterator) support. Exceptions support.

**4.01 23 Oct 2003** 

Fixed bug in rs2html(), tohtml.inc.php, that generated blank table cells.

Fixed insert\_id() incorrectly generated when logsql() enabled.

Modified PostgreSQL \_fixblobs to use list/each instead of foreach.

Informix ErrorNo() implemented correctly.

Modified several places to use list/each, including GetRowAssoc().

Added UserTimeStamp() to connection class.

Added $ADODB\_ANSI\_PADDING\_OFF for oci8po.

**4.00 20 Oct 2003** 

Upgraded adodb-xmlschema to 1 Oct 2003 snapshot.

Fix to rs2html warning message. Thx to Filo.

Fix for odbc\_mssql/mssql SQLDate(), hours was wrong.

Added MetaColumns and MetaPrimaryKeys for sybase. Thx to Chris Phillipson.

Added autoquoting to datadict for MySQL and PostgreSQL. Suggestion by Karsten Dambekalns

**3.94 11 Oct 2003** 

Create trigger in datadict-oci8.inc.php did not work, because all cr/lf's must be removed.

ErrorMsg()/ErrorNo() did not work for many databases when logging enabled. Fixed.

Removed global variable $ADODB\_LOGSQL as it does not work properly with multiple connections.

Added SQLDate support for sybase. Thx to Chris Phillipson

Postgresql checking of pgsql resultset resource was incorrect. Fix by Bharat Mediratta bharat#menalto.com.
Same patch applied to \_insertid and \_affectedrows for adodb-postgres64.inc.php.

Added support for NConnect for postgresql.

Added Sybase data dict support. Thx to Chris Phillipson

Extensive improvements in $perf->UI(), eg. Explain now opens in new window, we show scripts
which call sql, etc.

Perf Monitor UI works with magic quotes enabled.

rsPrefix was declared twice. Removed.

Oci8 stored procedure support, eg. "begin func(); end;" was incorrect in \_query. Fixed.

Tiraboschi Massimiliano contributed italian language file.

Fernando Ortiz, fortiz#lacorona.com.mx, contributed informix performance monitor.

Added \_varchar (varchar arrays) support for postgresql. Reported by PREVOT St�phane.

**3.92 22 Sept 2003**

Added GetAssoc and CacheGetAssoc to connection object.

Removed TextMax and CharMax functions from adodb.inc.php.

HasFailedTrans() returned false when trans failed. Fixed.

Moved perf driver classes into adodb/perf/\*.php.

Misc improvements to performance monitoring, including UI().

RETVAL in mssql Parameter(), we do not append @ now.

Added Param($name) to connection class, returns '?' or ":$name", for defining
bind parameters portably.

LogSQL traps affected\_rows() and saves its value properly now. Also fixed oci8
\_stmt and \_affectedrows() bugs.

Session code timestamp check for oci8 works now. Formerly default NLS\_DATE\_FORMAT
stripped off time portion. Thx to Tony Blair (tonanbarbarian#hotmail.com). Also
added new $conn->datetime field to oci8, controls whether MetaType() returns
'D' ($this->datetime==false) or 'T' ($this->datetime == true) for DATE type.

Fixed bugs in adodb-cryptsession.inc.php and adodb-session-clob.inc.php.

Fixed misc bugs in adodb\_key\_exists, GetInsertSQL() and GetUpdateSQL().

Tuned include\_once handling to reduce file-system checking overhead.

**3.91 9 Sept 2003**

Only released to InterAkt

Added LogSQL() for sql logging and $ADODB\_NEWCONNECTION to override factory
for driver instantiation.

Added IfNull($field,$ifNull) function, thx to johnwilk#juno.com

Added portable substr support.

Now rs2html() has new parameter, $echo. Set to false to return $html instead
of echoing it.

**3.90 5 Sept 2003**

First beta of performance monitoring released.

MySQL supports MetaTable() masking.

Fixed key\_exists() bug in adodb-lib.inc.php

Added sp\_executesql Prepare() support to mssql.

Added bind support to db2.

Added swedish language file - Christian Tiberg" christian#commsoft.nu

Bug in drop index for mssql data dict fixed. Thx to Gert-Rainer Bitterlich.

Left join setting for oci8 was wrong. Thx to johnwilk#juno.com

**3.80 27 Aug 2003**

Patch for PHP 4.3.3 cached recordset csv2rs() fread loop incompatibility.

Added matching mask for MetaTables. Only for oci8, mssql and postgres currently.

Rewrite of "oracle" driver connection code, merging with "oci8", by Gaetano.

Added better debugging for Smart Transactions.

Postgres DBTimeStamp() was wrongly using TO\_DATE. Changed to TO\_TIMESTAMP.

ADODB\_FETCH\_CASE check pushed to ADONewConnection to allow people to define
it after including adodb.inc.php.

Added portugese (brazilian) to languages. Thx to "Levi Fukumori".

Removed arg3 parameter from Execute/SelectLimit/Cache\* functions.

Execute() now accepts 2-d array as $inputarray. Also changed docs of fnExecute()
to note change in sql query counting with 2-d arrays.

Added MONEY to MetaType in PostgreSQL.

Added more debugging output to CacheFlush().

**3.72 9 Aug 2003**

Added qmagic($str), which is a qstr($str) that auto-checks for magic quotes
and does the right thing...

Fixed CacheFlush() bug - Thx to martin#gmx.de

Walt Boring contributed MetaForeignKeys for postgres7.

\_fetch() called \_BlobDecode() wrongly in interbase. Fixed.

adodb\_time bug fixed with dates after 2038 fixed by Jason Pell. http://phplens.com/lens/lensforum/msgs.php?id=6980

**3.71 4 Aug 2003**

The oci8 driver, MetaPrimaryKeys() did not check the owner correctly when $owner
== false.

Russian language file contributed by "Cyrill Malevanov" cyrill#malevanov.spb.ru.

Spanish language file contributed by "Horacio Degiorgi" horaciod#codigophp.com.

Error handling in oci8 bugfix - if there was an error in Execute(), then when
calling ErrorNo() and/or ErrorMsg(), the 1st call would return the error, but
the 2nd call would return no error.

Error handling in odbc bugfix. ODBC would always return the last error, even
if it happened 5 queries ago. Now we reset the errormsg to '' and errorno to
0 everytime before CacheExecute() and Execute().

**3.70 29 July 2003**

Added new SQLite driver. Tested on PHP 4.3 and PHP 5.

Added limited "sapdb" driver support - mainly date support.

The oci8 driver did not identify NUMBER with no defined precision correctly.

Added ADODB\_FORCE\_NULLS, if set, then PHP nulls are converted to SQL nulls
in GetInsertSQL/GetUpdateSQL.

DBDate() and DBTimeStamp() format for postgresql had problems. Fixed.

Added tableoptions to ChangeTableSQL(). Thx to Mike Benoit.

Added charset support to postgresql. Thx to Julian Tarkhanov.

Changed OS check for MS-Windows to prevent confusion with darWIN (MacOS)

Timestamp format for db2 was wrong. Changed to yyyy-mm-dd-hh.mm.ss.nnnnnn.

adodb-cryptsession.php includes wrong. Fixed.

Added MetaForeignKeys(). Supported by mssql, odbc\_mssql and oci8.

Fixed some oci8 MetaColumns/MetaPrimaryKeys bugs. Thx to Walt Boring.

adodb\_getcount() did not init qryRecs to 0. Missing "WHERE" clause checking
in GetUpdateSQL fixed. Thx to Sebastiaan van Stijn.

Added support for only 'VIEWS' and "TABLES" in MetaTables. From Walt Boring.

Upgraded to adodb-xmlschema.inc.php 0.0.2.

NConnect for mysql now returns value. Thx to Dennis Verspuij.

ADODB\_FETCH\_BOTH support added to interbase/firebird.

Czech language file contributed by Kamil Jakubovic jake#host.sk.

PostgreSQL BlobDecode did not use \_connectionID properly. Thx to Juraj Chlebec.

Added some new initialization stuff for Informix. Thx to "Andrea Pinnisi" pinnisi#sysnet.it

ADODB\_ASSOC\_CASE constant wrong in sybase \_fetch(). Fixed.

**3.60 16 June 2003**

We now SET CONCAT\_NULL\_YIELDS\_NULL OFF for odbc\_mssql driver to be compat with
mssql driver.

The property $emptyDate missing from connection class. Also changed 1903 to
constant (TIMESTAMP\_FIRST\_YEAR=100). Thx to Sebastiaan van Stijn.

ADOdb speedup optimization - we now return all arrays by reference.

Now DBDate() and DBTimeStamp() now accepts the string 'null' as a parameter.
Suggested by vincent.

Added GetArray() to connection class.

Added not\_null check in informix metacolumns().

Connection parameters for postgresql did not work correctly when port was defined.

DB2 is now a tested driver, making adodb 100% compatible. Extensive changes
to odbc driver for DB2, including implementing serverinfo() and SQLDate(), switching
to SQL\_CUR\_USE\_ODBC as the cursor mode, and lastAffectedRows and SelectLimit()
fixes.

The odbc driver's FetchField() field names did not obey ADODB\_ASSOC\_CASE. Fixed.

Some bugs in adodb\_backtrace() fixed.

Added "INT IDENTITY" type to adorecordset::MetaType() to support odbc\_mssql
properly.

MetaColumns() for oci8, mssql, odbc revised to support scale. Also minor revisions
to odbc MetaColumns() for vfp and db2 compat.

Added unsigned support to mysql datadict class. Thx to iamsure.

Infinite loop in mssql MoveNext() fixed when ADODB\_FETCH\_ASSOC used. Thx to
Josh R, Night\_Wulfe#hotmail.com.

ChangeTableSQL contributed by Florian Buzin.

The odbc\_mssql driver now sets CONCAT\_NULL\_YIELDS\_NULL OFF for compat with
mssql driver.

---

**0.10 Sept 9 2000** First release

### **Old changelog history moved to old-changelog.htm.**
