# Supplementary material for: A UMLS-based spell checker for natural language processing in vaccine safety
Source: BMC Med Inform Decis Mak. 2007 Feb 12;7:3. doi: 10.1186/1472-6947-7-3 (PMC1805499; doi:10.1186/1472-6947-7-3)
Supplement: Additional file 13 — RAP application directory. Sets up directory for RAP (RDF application for PHP) source code [file 1472-6947-7-3-S13.gz › rap/api/util/adodb/docs/docs-datadict.htm]

ADODB Data Dictionary Manual


## ADOdb Data Dictionary Library for PHP

V4.50 6 July 2004 (c) 2000-2004 John Lim (jlim#natsoft.com.my).  
AXMLS (c) 2004 ars Cognita, Inc

This software is dual licensed using BSD-Style and
LGPL. This means you can use it in compiled proprietary and commercial
products.

|  |
| --- |
| Kindly note that the ADOdb home page has moved to http://adodb.sourceforge.net/ because of the persistent unreliability of http://php.weblogs.com. **Please change your links**! |

Useful ADOdb links: Download
  Other Docs

This documentation describes a PHP class library to automate the
creation of tables, indexes and foreign key constraints portably for
multiple databases. Richard Tango-Lowy and Dan Cech have been kind
enough to contribute AXMLS, an XML schema
system for defining databases. You can contact them at
dcech#phpwerx.net and richtl#arscognita.com.

Currently the following databases are supported:

**Well-tested:** PostgreSQL, MySQL, Oracle, MSSQL.  
**Beta-quality:** DB2, Informix, Sybase, Interbase, Firebird.  
**Alpha-quality:** MS Access (does not support DEFAULT values) and
generic ODBC.

### Example Usage

```
  include_once('adodb.inc.php');  
  # First create a normal connection  
  $db->NewADOConnection('mysql');  
  $db->Connect(...);  
  
  # Then create a data dictionary object, using this connection  
  $dict = NewDataDictionary($db);  
  
  # We have a portable declarative data dictionary format in ADOdb, similar to SQL.  
  # Field types use 1 character codes, and fields are separated by commas.  
  # The following example creates three fields: "col1", "col2" and "col3":  
  $flds = "   
   col1 C(32) NOTNULL DEFAULT 'abc',  
   col2 I  DEFAULT 0,  
   col3 N(12.2)  
  ";  
  
  # We demonstrate creating tables and indexes  
  $sqlarray = $dict->CreateTableSQL($tabname, $flds, $taboptarray);  
  $dict->ExecuteSQLArray($sqlarray);  
  
  $idxflds = 'co11, col2';  
  $sqlarray = $dict->CreateIndexSQL($idxname, $tabname, $idxflds);  
  $dict->ExecuteSQLArray($sqlarray);
```

### Functions

#### function CreateDatabase($dbname, $optionsarray=false)

Create a database with the name $dbname;

#### function CreateTableSQL($tabname, $fldarray, $taboptarray=false)

```
  RETURNS:      an array of strings, the sql to be executed, or false  
  $tabname:     name of table  
  $fldarray:    string (or array) containing field info  
  $taboptarray: array containing table options
```

The new format of $fldarray uses a free text format, where each
field is comma-delimited.
The first token for each field is the field name, followed by the type
and optional
field size. Then optional keywords in $otheroptions:

```
  "$fieldname $type $colsize $otheroptions"
```

The older (and still supported) format of $fldarray is a
2-dimensional array, where each row in the 1st dimension represents one
field. Each row has this format:

```
  array($fieldname, $type, [,$colsize] [,$otheroptions]*)
```

The first 2 fields must be the field name and the field type. The
field type can be a portable type codes or the actual type for that
database.

Legal portable type codes include:

```
  C:  varchar  
  X:  Largest varchar size   
  XL: For Oracle, returns CLOB, otherwise same as 'X' above  
  
  C2: Multibyte varchar  
  X2: Multibyte varchar (largest size)  
  
  B:  BLOB (binary large object)  
  
  D:  Date (some databases do not support this, and we return a datetime type)  
  T:  Datetime or Timestamp  
  L:  Integer field suitable for storing booleans (0 or 1)  
  I:  Integer (mapped to I4)  
  I1: 1-byte integer  
  I2: 2-byte integer  
  I4: 4-byte integer  
  I8: 8-byte integer  
  F:  Floating point number  
  N:  Numeric or decimal number
```

The $colsize field represents the size of the field. If a decimal
number is used, then it is assumed that the number following the dot is
the precision, so 6.2 means a number of size 6 digits and 2 decimal
places. It is recommended that the default for number types be
represented as a string to avoid any rounding errors.

The $otheroptions include the following keywords (case-insensitive):

```
  AUTO            For autoincrement number. Emulated with triggers if not available.  
                  Sets NOTNULL also.  
  AUTOINCREMENT   Same as auto.  
  KEY             Primary key field. Sets NOTNULL also. Compound keys are supported.  
  PRIMARY         Same as KEY.  
  DEF             Synonym for DEFAULT for lazy typists.  
  DEFAULT         The default value. Character strings are auto-quoted unless  
                  the string begins and ends with spaces, eg ' SYSDATE '.  
  NOTNULL         If field is not null.  
  DEFDATE         Set default value to call function to get today's date.  
  DEFTIMESTAMP    Set default to call function to get today's datetime.  
  NOQUOTE         Prevents autoquoting of default string values.  
  CONSTRAINTS     Additional constraints defined at the end of the field  
                  definition.
```

The Data Dictonary accepts two formats, the older array
specification:

```
  $flds = array(  
    array('COLNAME',   'DECIMAL', '8.4', 'DEFAULT' => 0, 'NOTNULL'),  
    array('id',        'I'      , 'AUTO'),  
    array('`MY DATE`', 'D'      , 'DEFDATE'),  
    array('NAME',      'C'      , '32', 'CONSTRAINTS' => 'FOREIGN KEY REFERENCES reftable')  
  );
```

Or the simpler declarative format:

```
  $flds = "  
    COLNAME DECIMAL(8.4) DEFAULT 0 NOTNULL,  
    id I AUTO,  
    `MY DATE` D DEFDATE,  
    NAME C(32) CONSTRAINTS 'FOREIGN KEY REFERENCES reftable'  
  ";
```

Note that if you have special characters in the field name (e.g. My
Date), you should enclose it in back-quotes. Normally field names are
not case-sensitive, but if you enclose it in back-quotes, some
databases will treat the names as case-sensitive (eg. Oracle) , and
others won't. So be careful.

The $taboptarray is the 3rd parameter of the CreateTableSQL
function. This contains table specific settings. Legal keywords include:

- **REPLACE**  
  Indicates that the previous table definition should be removed
  (dropped)together with ALL data. See first example below.
- **DROP**  
  Drop table. Useful for removing unused tables.
- **CONSTRAINTS**  
  Define this as the key, with the constraint as the value. See the
  postgresql example below. Additional constraints defined for the whole
  table. You will probably need to prefix this with a comma.

Database specific table options can be defined also using the name
of the database type as the array key. In the following example, *create
the table as ISAM with MySQL, and store the table in the "users"
tablespace if using Oracle*. And because we specified REPLACE, drop
the table first.

```
  $taboptarray = array('mysql' => 'TYPE=ISAM', 'oci8' => 'tablespace users', 'REPLACE');
```

You can also define foreignkey constraints. The following is syntax
for postgresql:

```
  $taboptarray = array('constraints' => ', FOREIGN KEY (col1) REFERENCES reftable (refcol)');
```

#### function DropTableSQL($tabname)

Returns the SQL to drop the specified table.

#### function ChangeTableSQL($tabname, $flds)

Checks to see if table exists, if table does not exist, behaves like
CreateTableSQL. If table exists, generates appropriate ALTER TABLE
MODIFY COLUMN commands if field already exists, or ALTER TABLE ADD
$column if field does not exist.

The class must be connected to the database for ChangeTableSQL to
detect the existence of the table. Idea and code contributed by Florian
Buzin.

#### function CreateIndexSQL($idxname, $tabname, $flds, $idxoptarray=false)

```
  RETURNS:      an array of strings, the sql to be executed, or false  
  $idxname:     name of index  
  $tabname:     name of table  
  $flds:        list of fields as a comma delimited string or an array of strings  
  $idxoptarray: array of index creation options
```

$idxoptarray is similar to $taboptarray in that index specific
information can be embedded in the array. Other options include:

```
  CLUSTERED     Create clustered index (only mssql)  
  BITMAP        Create bitmap index (only oci8)  
  UNIQUE        Make unique index  
  FULLTEXT      Make fulltext index (only mysql)  
  HASH          Create hash index (only postgres)  
  DROP          Drop legacy index
```

#### function DropIndexSQL ($idxname, $tabname = NULL)

Returns the SQL to drop the specified index.

#### function AddColumnSQL($tabname, $flds)

Add one or more columns. Not guaranteed to work under all situations.

#### function AlterColumnSQL($tabname, $flds)

Warning, not all databases support this feature.

#### function DropColumnSQL($tabname, $flds)

Drop 1 or more columns.

#### function SetSchema($schema)

Set the schema.

#### function &MetaTables()

#### function &MetaColumns($tab, $upper=true, $schema=false)

#### function &MetaPrimaryKeys($tab,$owner=false,$intkey=false)

#### function &MetaIndexes($table, $primary = false, $owner = false)

These functions are wrappers for the corresponding functions in the
connection object. However, the table names will be autoquoted by the
TableName function (see below) before being passed to the connection
object.

#### function NameQuote($name = NULL)

If the provided name is quoted with backquotes (`) or contains
special characters, returns the name quoted with the appropriate quote
character, otherwise the name is returned unchanged.

#### function TableName($name)

The same as NameQuote, but will prepend the current schema if
specified

#### function MetaType($t,$len=-1,$fieldobj=false)

#### function ActualType($meta)

Convert between database-independent 'Meta' and database-specific
'Actual' type codes.

#### function ExecuteSQLArray($sqlarray, $contOnError = true)

```
  RETURNS:      0 if failed, 1 if executed all but with errors, 2 if executed successfully  
  $sqlarray:    an array of strings with sql code (no semicolon at the end of string)  
  $contOnError: if true, then continue executing even if error occurs
```

Executes an array of SQL strings returned by CreateTableSQL or
CreateIndexSQL.

---


## ADOdb XML Schema (AXMLS)

This is a class contributed by Richard Tango-Lowy and Dan Cech that
allows the user to quickly
and easily build a database using the excellent ADODB database library
and a simple XML formatted file.
You can download
the latest version of AXMLS here.

### Quick Start

Adodb-xmlschema, or AXMLS, is a set of classes that allow the user
to quickly and easily build or upgrade a database on almost any RDBMS
using the excellent ADOdb database library and a simple XML formatted
schema file. Our goal is to give developers a tool that's simple to
use, but that will allow them to create a single file that can build,
upgrade, and manipulate databases on most RDBMS platforms.

 Installing axmls

The easiest way to install AXMLS to download and install any recent
version of the ADOdb database abstraction library. To install AXMLS
manually, simply copy the adodb-xmlschema.inc.php file and the xsl
directory into your adodb directory.

 Using AXMLS in Your Application

There are two steps involved in using AXMLS in your application:
first, you must create a schema, or XML representation of your
database, and second, you must create the PHP code that will parse and
execute the schema.

Let's begin with a schema that describes a typical, if simplistic
user management table for an application.

```
```
<?xml version="1.0"?>  
<schema version="0.2">  
  
  <table name="users">  
    <desc>A typical users table for our application.</desc>  
    <field name="userId" type="I">  
      <descr>A unique ID assigned to each user.</descr>  
  
      <KEY/>  
      <AUTOINCREMENT/>  
    </field>  
      
    <field name="userName" type="C" size="16"><NOTNULL/></field>  
  
      
    <index name="userName">  
      <descr>Put a unique index on the user name</descr>  
      <col>userName</col>  
      <UNIQUE/>  
  
    </index>  
  </table>  
    
  <sql>  
    <descr>Insert some data into the users table.</descr>  
    <query>insert into users (userName) values ( 'admin' )</query>  
  
    <query>insert into users (userName) values ( 'Joe' )</query>  
  </sql>  
</schema>			
```
```

Let's take a detailed look at this schema.

The opening <?xml version="1.0"?> tag is required by XML. The
<schema> tag tells the parser that the enclosed markup defines an
XML schema. The version="0.2" attribute sets *the version of the
AXMLS DTD used by the XML schema.*

All versions of AXMLS prior to version 1.0 have a schema version of
"0.1". The current schema version is "0.2".

```
```
<?xml version="1.0"?>  
<schema version="0.2">  
  ...  
</schema>
```
```

Next we define one or more tables. A table consists of a fields (and
other objects) enclosed by <table> tags. The name="" attribute
specifies the name of the table that will be created in the database.

```
```
<table name="users">  
  
    <desc>A typical users table for our application.</desc>  
    <field name="userId" type="I">  
  
      <descr>A unique ID assigned to each user.</descr>  
      <KEY/>  
      <AUTOINCREMENT/>  
    </field>  
      
    <field name="userName" type="C" size="16"><NOTNULL/></field>  
  
      
</table>
```
```

This table is called "users" and has a description and two fields.
The description is optional, and is currently only for your own
information; it is not applied to the database.

The first <field> tag will create a field named "userId" of
type "I", or integer. (See the ADOdb Data Dictionary documentation for
a list of valid types.) This <field> tag encloses two special
field options: <KEY/>, which specifies this field as a primary
key, and <AUTOINCREMENT/>, which specifies that the database
engine should automatically fill this field with the next available
value when a new row is inserted.

The second <field> tag will create a field named "userName" of
type "C", or character, and of length 16 characters. The
<NOTNULL/> option specifies that this field does not allow NULLs.

There are two ways to add indexes to a table. The simplest is to
mark a field with the <KEY/> option as described above; a primary
key is a unique index. The second and more powerful method uses the
<index> tags.

```
```
<table name="users">  
  ...  
      
  <index name="userName">  
    <descr>Put a unique index on the user name</descr>  
    <col>userName</col>  
  
    <UNIQUE/>  
  </index>  
      
</table>
```
```

The <index> tag specifies that an index should be created on
the enclosing table. The name="" attribute provides the name of the
index that will be created in the database. The description, as above,
is for your information only. The <col> tags list each column
that will be included in the index. Finally, the <UNIQUE/> tag
specifies that this will be created as a unique index.

Finally, AXMLS allows you to include arbitrary SQL that will be
applied to the database when the schema is executed.

```
```
<sql>  
  <descr>Insert some data into the users table.</descr>  
  <query>insert into users (userName) values ( 'admin' )</query>  
  
  <query>insert into users (userName) values ( 'Joe' )</query>  
</sql>
```
```

The <sql> tag encloses any number of SQL queries that you
define for your own use.

Now that we've defined an XML schema, you need to know how to apply
it to your database. Here's a simple PHP script that shows how to load
the schema.

```
```
<?PHP  
/* You must tell the script where to find the ADOdb and  
 * the AXMLS libraries.  
 */  
require( "path_to_adodb/adodb.inc.php");  
require( "path_to_adodb/adodb-xmlschema.inc.php" );  
  
/* Configuration information. Define the schema filename,  
 * RDBMS platform (see the ADODB documentation for valid  
 * platform names), and database connection information here.  
 */  
$schemaFile = 'example.xml';  
$platform = 'mysql';  
$dbHost = 'localhost';  
$dbName = 'database';  
$dbUser = 'username';  
$dbPassword = 'password';  
  
/* Start by creating a normal ADODB connection.  
 */  
$db = ADONewConnection( $platform );  
$db->Connect( $dbHost, $dbUser, $dbPassword, $dbName );  
  
/* Use the database connection to create a new adoSchema object.  
 */  
$schema = new adoSchema( $db );  
  
/* Call ParseSchema() to build SQL from the XML schema file.  
 * Then call ExecuteSchema() to apply the resulting SQL to   
 * the database.  
 */  
$sql = $schema->ParseSchema( $schemaFile );  
$result = $schema->ExecuteSchema();  
?>
```
```

Let's look at each part of the example in turn. After you manually
create the database, there are three steps required to load (or
upgrade) your schema.

First, create a normal ADOdb connection. The variables and values
here should be those required to connect to your database.

```
```
$db = ADONewConnection( 'mysql' );  
$db->Connect( 'host', 'user', 'password', 'database' );
```
```

Second, create the adoSchema object that load and manipulate your
schema. You must pass an ADOdb database connection object in order to
create the adoSchema object.

```
```
$schema = new adoSchema( $db );
```
```

Third, call ParseSchema() to parse the schema and then
ExecuteSchema() to apply it to the database. You must pass
ParseSchema() the path and filename of your schema file.

```
```
$schema->ParseSchema( $schemaFile );   
$schema->ExecuteSchema();
```
```

Execute the above code and then log into your database. If you've
done all this right, you should see your tables, indexes, and SQL.

You can find the source files for this tutorial in the examples
directory as tutorial\_shema.xml and tutorial.php. See the class
documentation for a more detailed description of the adoSchema methods,
including methods and schema elements that are not described in this
tutorial.

### Upgrading

If your schema version is older, than XSLT is used to transform the
schema to the newest version. This means that if you are using an older
XML schema format, you need to have the XSLT extension installed.
If you do not want to require your users to have the XSLT extension
installed, make sure you modify your XML schema to conform to the
latest version.

---

If you have any questions or comments, please email them to
Richard at richtl#arscognita.com.
