# Supplementary material for: A UMLS-based spell checker for natural language processing in vaccine safety
Source: BMC Med Inform Decis Mak. 2007 Feb 12;7:3. doi: 10.1186/1472-6947-7-3 (PMC1805499; doi:10.1186/1472-6947-7-3)
Supplement: Additional file 13 — RAP application directory. Sets up directory for RAP (RDF application for PHP) source code [file 1472-6947-7-3-S13.gz › rap/api/util/adodb/docs/docs-perf.htm]

ADOdb Performance Monitoring Library


### The ADOdb Performance Monitoring Library

V4.50 6 July 2004 (c) 2000-2004 John Lim (jlim#natsoft.com.my)

This software is dual licensed using BSD-Style and
LGPL. This means you can use it in compiled proprietary and commercial
products.

|  |
| --- |
| Kindly note that the ADOdb home page has moved to http://adodb.sourceforge.net/ because of the persistent unreliability of http://php.weblogs.com. **Please change your links**! |

Useful ADOdb links: Download
  Other Docs

### Introduction

This module, part of the ADOdb package, provides both CLI and HTML
interfaces for viewing key performance indicators of your database.
This is very useful because web apps such as the popular phpMyAdmin
currently do not provide effective database health monitoring tools.
The module provides the following:

- A quick health check of your database server using `$perf->HealthCheck()`
  or `$perf->HealthCheckCLI()`.
- User interface for performance monitoring, `$perf->UI()`.
  This UI displays:
  - the health check,
  - all SQL logged and their query plans,
  - a list of all tables in the current database
  - an interface to continiously poll the server for key
    performance indicators such as CPU, Hit Ratio, Disk I/O
  - a form where you can enter and run SQL interactively.
- Gives you an API to build database monitoring tools for a server
  farm, for example calling `$perf->DBParameter('data cache hit
  ratio')` returns this very important statistic in a database
  independant manner.

ADOdb also has the ability to log all SQL executed, using LogSQL. All SQL logged can be
analyzed through the performance monitor UI. In the *View
SQL* mode, we categorize the SQL into 3 types:

- **Suspicious SQL**: queries with high average execution times,
  and are potential candidates for rewriting
- **Expensive SQL**: queries with high total execution times
  (#executions \* avg execution time). Optimizing these queries will
  reduce your database server load.
- **Invalid SQL**: queries that generate errors.

Each query is hyperlinked to a description of the query plan, and
every PHP script that executed that query is also shown.

Please note that the information presented is a very basic database
health check, and does not provide a complete overview of database
performance. Although some attempt has been made to make it work across
multiple databases in the same way, it is impossible to do so. For the
health check, we do try to display the following key database
parameters for all drivers:

- **data cache size** - The amount of memory allocated to the
  cache.
- **data cache hit ratio** - A measure of how effective the
  cache is, as a percentage. The higher, the better.
- **current connections** - The number of sessions currently
  connected to the database.

You will need to connect to the database as an administrator to view
most of the parameters.

Code improvements as very welcome, particularly adding new database
parameters and automated tuning hints.

### Usage

Currently, the following drivers: *mysql*, *postgres*,
*oci8*, *mssql*, *informix* and *db2* are
supported. To create a new performance monitor, call NewPerfMonitor( )
as demonstrated below:

```
<?php  
include_once('adodb.inc.php');  
session_start(); # session variables required for monitoring  
$conn = ADONewConnection($driver);  
$conn->Connect($server,$user,$pwd,$db);  
$perf =& NewPerfMonitor($conn);  
$perf->UI($pollsecs=5);  
?>
```

It is also possible to retrieve a single database parameter:

```
$size = $perf->DBParameter('data cache size');
```

Thx to Fernando Ortiz for the informix module.

### Methods

function **UI($pollsecs=5)**

Creates a web-based user interface for performance monitoring. When
you click on Poll, server statistics will be displayed every $pollsecs
seconds. See Usage above.

Since 4.11, we allow users to enter and run SQL interactively via
the "Run SQL" link. To disable this for security reasons, set this
constant before calling $perf->UI().

```
define('ADODB_PERF_NO_RUN_SQL',1);
```

Sample output follows below:

|  |
| --- |
| **ADOdb Performance Monitor** for localhost, db=test  PostgreSQL 7.3.2 on i686-pc-cygwin, compiled by GCC gcc (GCC) 3.2 20020927 (prerelease) |
| Performance Stats   View SQL   View Tables   Poll Stats |

|  |  |  |
| --- | --- | --- |
| postgres7 | | |
| **Parameter** | **Value** | **Description** |
| *Ratios* | | |
| statistics collector | TRUE | Value must be TRUE to enable hit ratio statistics (*stats\_start\_collector*,*stats\_row\_level* and *stats\_block\_level* must be set to true in postgresql.conf) |
| data cache hit ratio | 99.7967555299239 |  |
| *IO* | | |
| data reads | 125 |  |
| data writes | 21.78125000000000000 | Count of inserts/updates/deletes \* coef |
| *Data Cache* | | |
| data cache buffers | 640 | Number of cache buffers. Tuning |
| cache blocksize | 8192 | (estimate) |
| data cache size | 5M |  |
| operating system cache size | 80M | (effective cache size) |
| *Memory Usage* | | |
| sort buffer size | 1M | Size of sort buffer (per query) |
| *Connections* | | |
| current connections | 0 |  |
| max connections | 32 |  |
| *Parameters* | | |
| rollback buffers | 8 | WAL buffers |
| random page cost | 4 | Cost of doing a seek (default=4). See random\_page\_cost |

function **HealthCheck**()

Returns database health check parameters as a HTML table. You will
need to echo or print the output of this function,

function **HealthCheckCLI**()

Returns database health check parameters formatted for a command
line interface. You will need to echo or print the output of this
function. Sample output for mysql:

```
-- Ratios --   
          MyISAM cache hit ratio => 56.5635738832   
          InnoDB cache hit ratio => 0   
             sql cache hit ratio => 0   
 -- IO --   
                      data reads => 2622   
                     data writes => 2415.5   
 -- Data Cache --   
          MyISAM data cache size => 512K   
             BDB data cache size => 8388600  
          InnoDB data cache size => 8M  
 -- Memory Pools --   
                read buffer size => 131072   
                sort buffer size => 65528   
                     table cache => 4   
 -- Connections --   
             current connections => 3  
                 max connections => 100
```

function **Poll**($pollSecs=5)

Run in infinite loop, displaying the following information every
$pollSecs. This will not work properly if output buffering is enabled.
In the example below, $pollSecs=3:

```
Accumulating statistics...  
 Time   WS-CPU%   Hit%   Sess        Reads/s          Writes/s  
11:08:30    0.7  56.56      1         0.0000            0.0000  
11:08:33    1.8  56.56      2         0.0000            0.0000  
11:08:36   11.1  56.55      3         2.5000            0.0000  
11:08:39    9.8  56.55      2         3.1121            0.0000  
11:08:42    2.8  56.55      1         0.0000            0.0000  
11:08:45    7.4  56.55      2         0.0000            1.5000
```

**WS-CPU%** is the Web Server CPU load of the server that PHP is
running from (eg. the database client), and not the database. The **Hit%**
is the data cache hit ratio. **Sess** is the current number of
sessions connected to the database. If you are using persistent
connections, this should not change much. The **Reads/s** and **Writes/s**
are synthetic values to give the viewer a rough guide to I/O, and are
not to be taken literally.

function **SuspiciousSQL**($numsql=10)

Returns SQL which have high average execution times as a HTML table.
Each sql statement
is hyperlinked to a new window which details the execution plan and the
scripts that execute this SQL.

The number of statements returned is determined by $numsql. Data is
taken from the adodb\_logsql table, where the sql statements are logged
when
$connection->LogSQL(true) is enabled. The adodb\_logsql table is
populated using $conn->LogSQL.

For Oracle, Ixora Suspicious SQL returns a list of SQL statements
that are most cache intensive as a HTML table. These are data intensive
SQL statements that could benefit most from tuning.

function **ExpensiveSQL**($numsql=10)

Returns SQL whose total execution time (avg time \* #executions) is
high as a HTML table. Each sql statement
is hyperlinked to a new window which details the execution plan and the
scripts that execute this SQL.

The number of statements returned is determined by $numsql. Data is
taken from the adodb\_logsql table, where the sql statements are logged
when
$connection->LogSQL(true) is enabled. The adodb\_logsql table is
populated using $conn->LogSQL.

For Oracle, Ixora Expensive SQL returns a list of SQL statements
that are taking the most CPU load when run.

function **InvalidSQL**($numsql=10)

Returns a list of invalid SQL as an HTML table.

Data is taken from the adodb\_logsql table, where the sql statements
are logged when
$connection->LogSQL(true) is enabled.

function **Tables**($orderby=1)

Returns information on all tables in a database, with the first two
fields containing the table name and table size, the remaining fields
depend on the database driver. If $orderby is set to 1, it will sort by
name. If $orderby is set to 2, then it will sort by table size. Some
database drivers (mssql and mysql) will ignore the $orderby clause. For
postgresql, the information is up-to-date since the last *vacuum*.
Not supported currently for db2.

### Raw Functions

Raw functions return values without any formatting.

function **DBParameter**($paramname)

Returns the value of a database parameter, such as
$this->DBParameter("data cache size").

function **CPULoad**()

Returns the CPU load of the database client (NOT THE SERVER) as a
percentage. Only works for Linux and Windows. For Windows, WMI must be
available.

### Format of $settings Property

To create new database parameters, you need to understand
$settings. The $settings data structure is an associative array. Each
element of the array defines a database parameter. The key is the name
of the database parameter. If no key is defined, then it is assumed to
be a section break, and the value is the name of the section break. If
this is too confusing, looking at the source code will help a lot!

Each database parameter is itself an array consisting of the
following elements:

0. Category code, used to group related db parameters. If the
   category code is 'HIDE', then
   the database parameter is not shown when HTML() is called.
1. either
   1. sql string to retrieve value, eg. "select value from
      v\$parameter where name='db\_block\_size'",
   2. array holding sql string and field to look for, e.g.
      array('show variables','table\_cache'); optional 3rd parameter is the
      $rs->fields[$index] to use (otherwise $index=1), and optional 4th
      parameter is a constant to multiply the result with (typically 100 for
      percentage calculations),
   3. a string prefixed by =, then a PHP method of the class is
      invoked, e.g. to invoke $this->GetIndexValue(), set this array
      element to '=GetIndexValue',
2. Description of database parameter. If description begins with an
   =, then it is interpreted as a method call, just as in (1c) above,
   taking one parameter, the current value. E.g. '=GetIndexDescription'
   will invoke $this->GetIndexDescription($val). This is useful for
   generating tuning suggestions. For an example, see WarnCacheRatio().

Example from MySQL, table\_cache database parameter:

```
'table cache' => array('CACHE',            # category code  
   array("show variables", 'table_cache'), # array (type 1b)  
   'Number of tables to keep open'),       # description
```

### Example Health Check Output

db2 informix mysql mssql oci8
postgres

|  |  |  |
| --- | --- | --- |
| db2 | | |
| **Parameter** | **Value** | **Description** |
| *Ratios* | | |
| data cache hit ratio | 0 |  |
| *Data Cache* | | |
| data cache buffers | 250 | See tuning reference. |
| cache blocksize | 4096 |  |
| data cache size | 1000K |  |
| *Connections* | | |
| current connections | 2 |  |

|  |  |  |
| --- | --- | --- |
| informix | | |
| **Parameter** | **Val ue** | **Description** |
| *Ratios* | | |
| data cache hit ratio | 95.89 |  |
| *IO* | | |
| data reads | 1883884 | Page reads |
| data writes | 1716724 | Page writes |
| *Connections* | | |
| current connections | 263.0 | Number of sessions |

|  |  |  |
| --- | --- | --- |
| mysql | | |
| **Parameter** | **Value** | **Description** |
| *Ratios* | | |
| MyISAM cache hit ratio | 56.5658301822 | **Cache ratio should be at least 90%** |
| InnoDB cache hit ratio | 0 | **Cache ratio should be at least 90%** |
| sql cache hit ratio | 0 |  |
| *IO* | | |
| data reads | 2622 | Number of selects (Key\_reads is not accurate) |
| data writes | 2415.5 | Number of inserts/updates/deletes \* coef (Key\_writes is not accurate) |
| *Data Cache* | | |
| MyISAM data cache size | 512K |  |
| BDB data cache size | 8388600 |  |
| InnoDB data cache size | 8M |  |
| *Memory Pools* | | |
| read buffer size | 131072 | (per session) |
| sort buffer size | 65528 | Size of sort buffer (per session) |
| table cache | 4 | Number of tables to keep open |
| *Connections* | | |
| current connections | 3 |  |
| max connections | 100 |  |

|  |  |  |
| --- | --- | --- |
| mssql | | |
| **Parameter** | **Value** | **Description** |
| *Ratios* | | |
| data cache hit ratio | 99.9999694824 |  |
| prepared sql hit ratio | 99.7738579828 |  |
| adhoc sql hit ratio | 98.4540169133 |  |
| *IO* | | |
| data reads | 2858 |  |
| data writes | 1438 |  |
| *Data Cache* | | |
| data cache size | 4362 | in K |
| *Connections* | | |
| current connections | 14 |  |
| max connections | 32767 |  |

|  |  |  |
| --- | --- | --- |
| oci8 | | |
| **Parameter** | **Value** | **Description** |
| *Ratios* | | |
| data cache hit ratio | 96.98 |  |
| sql cache hit ratio | 99.96 |  |
| *IO* | | |
| data reads | 842938 |  |
| data writes | 16852 |  |
| *Data Cache* | | |
| data cache buffers | 3072 | Number of cache buffers |
| data cache blocksize | 8192 |  |
| data cache size | 48M | shared\_pool\_size |
| *Memory Pools* | | |
| java pool size | 0 | java\_pool\_size |
| sort buffer size | 512K | sort\_area\_size (per query) |
| user session buffer size | 8M | large\_pool\_size |
| *Connections* | | |
| current connections | 1 |  |
| max connections | 170 |  |
| data cache utilization ratio | 88.46 | Percentage of data cache actually in use |
| user cache utilization ratio | 91.76 | Percentage of user cache (large\_pool) actually in use |
| rollback segments | 11 |  |
| *Transactions* | | |
| peak transactions | 24 | Taken from high-water-mark |
| max transactions | 187 | max transactions / rollback segments < 3.5 (or transactions\_per\_rollback\_segment) |
| *Parameters* | | |
| cursor sharing | EXACT | Cursor reuse strategy. Recommended is FORCE (8i+) or SIMILAR (9i+). See cursor\_sharing. |
| index cache cost | 0 | % of indexed data blocks expected in the cache. Recommended is 20-80. Default is 0. See optimizer\_index\_caching. |
| random page cost | 100 | Recommended is 10-50 for TP, and 50 for data warehouses. Default is 100. See optimizer\_index\_cost\_adj. |

### Suspicious SQL

|  |  |  |
| --- | --- | --- |
| **LOAD** | **EXECUTES** | **SQL\_TEXT** |
| .73% | 89 | select u.name, o.name, t.spare1, t.pctfree$ from sys.obj$ o, sys.user$ u, sys.tab$ t where (bitand(t.trigflag, 1048576) = 1048576) and o.obj#=t.obj# and o.owner# = u.user# select i.obj#, i.flags, u.name, o.name from sys.obj$ o, sys.user$ u, sys.ind$ i where (bitand(i.flags, 256) = 256 or bitand(i.flags, 512) = 512) and (not((i.type# = 9) and bitand(i.flags,8) = 8)) and o.obj#=i.obj# and o.owner# = u.user# |
| .84% | 3 | select /\*+ RULE \*/ distinct tabs.table\_name, tabs.owner , partitioned, iot\_type , TEMPORARY, table\_type, table\_type\_owner from DBA\_ALL\_TABLES tabs where tabs.owner = :own |
| 3.95% | 6 | SELECT round(count(1)\*avg(buf.block\_size)/1048576) FROM DBA\_OBJECTS obj, V$BH bh, dba\_segments seg, v$buffer\_pool buf WHERE obj.object\_id = bh.objd AND obj.owner != 'SYS' and obj.owner = seg.owner and obj.object\_name = seg.segment\_name and obj.object\_type = seg.segment\_type and seg.buffer\_pool = buf.name and buf.name = 'DEFAULT' |
| 4.50% | 6 | SELECT round(count(1)\*avg(tsp.block\_size)/1048576) FROM DBA\_OBJECTS obj, V$BH bh, dba\_segments seg, dba\_tablespaces tsp WHERE obj.object\_id = bh.objd AND obj.owner != 'SYS' and obj.owner = seg.owner and obj.object\_name = seg.segment\_name and obj.object\_type = seg.segment\_type and seg.tablespace\_name = tsp.tablespace\_name |
| 57.34% | 9267 | select t.schema, t.name, t.flags, q.name from system.aq$\_queue\_tables t, sys.aq$\_queue\_table\_affinities aft, system.aq$\_queues q where aft.table\_objno = t.objno and aft.owner\_instance = :1 and q.table\_objno = t.objno and q.usage = 0 and bitand(t.flags, 4+16+32+64+128+256) = 0 for update of t.name, aft.table\_objno skip locked |

### Expensive SQL

|  |  |  |
| --- | --- | --- |
| **LOAD** | **EXECUTES** | **SQL\_TEXT** |
| 5.24% | 1 | select round(sum(bytes)/1048576) from dba\_segments |
| 6.89% | 6 | SELECT round(count(1)\*avg(buf.block\_size)/1048576) FROM DBA\_OBJECTS obj, V$BH bh, dba\_segments seg, v$buffer\_pool buf WHERE obj.object\_id = bh.objd AND obj.owner != 'SYS' and obj.owner = seg.owner and obj.object\_name = seg.segment\_name and obj.object\_type = seg.segment\_type and seg.buffer\_pool = buf.name and buf.name = 'DEFAULT' |
| 7.85% | 6 | SELECT round(count(1)\*avg(tsp.block\_size)/1048576) FROM DBA\_OBJECTS obj, V$BH bh, dba\_segments seg, dba\_tablespaces tsp WHERE obj.object\_id = bh.objd AND obj.owner != 'SYS' and obj.owner = seg.owner and obj.object\_name = seg.segment\_name and obj.object\_type = seg.segment\_type and seg.tablespace\_name = tsp.tablespace\_name |
| 33.69% | 89 | select u.name, o.name, t.spare1, t.pctfree$ from sys.obj$ o, sys.user$ u, sys.tab$ t where (bitand(t.trigflag, 1048576) = 1048576) and o.obj#=t.obj# and o.owner# = u.user# |
| 36.44% | 89 | select i.obj#, i.flags, u.name, o.name from sys.obj$ o, sys.user$ u, sys.ind$ i where (bitand(i.flags, 256) = 256 or bitand(i.flags, 512) = 512) and (not((i.type# = 9) and bitand(i.flags,8) = 8)) and o.obj#=i.obj# and o.owner# = u.user# |

|  |  |  |
| --- | --- | --- |
| postgres7 | | |
| **Parameter** | **Value** | **Description** |
| *Ratios* | | |
| statistics collector | FALSE | Must be set to TRUE to enable hit ratio statistics (*stats\_start\_collector*,*stats\_row\_level* and *stats\_block\_level* must be set to true in postgresql.conf) |
| data cache hit ratio | 99.9666031916603 |  |
| *IO* | | |
| data reads | 15 |  |
| data writes | 0.000000000000000000 | Count of inserts/updates/deletes \* coef |
| *Data Cache* | | |
| data cache buffers | 1280 | Number of cache buffers. Tuning |
| cache blocksize | 8192 | (estimate) |
| data cache size | 10M |  |
| operating system cache size | 80000K | (effective cache size) |
| *Memory Pools* | | |
| sort buffer size | 1M | Size of sort buffer (per query) |
| *Connections* | | |
| current connections | 13 |  |
| max connections | 32 |  |
| *Parameters* | | |
| rollback buffers | 8 | WAL buffers |
| random page cost | 4 | Cost of doing a seek (default=4). See random\_page\_cost |
