# Supplementary material for: A UMLS-based spell checker for natural language processing in vaccine safety
Source: BMC Med Inform Decis Mak. 2007 Feb 12;7:3. doi: 10.1186/1472-6947-7-3 (PMC1805499; doi:10.1186/1472-6947-7-3)
Supplement: Additional file 13 — RAP application directory. Sets up directory for RAP (RDF application for PHP) source code [file 1472-6947-7-3-S13.gz › rap/api/util/adodb/docs/docs-session.htm]

ADODB Session Management Manual


### ADODB Session Management Manual

V4.50 6 July 2004 (c) 2000-2004 John Lim (jlim#natsoft.com.my)

This software is dual licensed using BSD-Style and
LGPL. This means you can use it in compiled proprietary and commercial
products. 

|  |
| --- |
| Kindly note that the ADOdb home page has moved to http://adodb.sourceforge.net/ because of the persistent unreliability of http://php.weblogs.com. **Please change your links**! |
|

Useful ADOdb links: Download
  Other Docs

### Introduction

We store state information specific to a user or web client in
session variables. These session variables persist throughout a
session, as the user moves from page to page.

To use session variables, call session\_start() at the beginning of
your web page, before your HTTP headers are sent. Then for every
variable you want to keep alive for the duration of the session, call
session\_register($variable\_name). By default, the session handler will
keep track of the session by using a cookie. You can save objects or
arrays in session variables also.

The default method of storing sessions is to store it in a file.
However if you have special needs such as you:

- Have multiple web servers that need to share session info
- Need to do special processing of each session
- Require notification when a session expires

Then the ADOdb session handler provides you with the above
additional capabilities by storing the session information as records
in a database table that can be shared across multiple servers.

**Important Upgrade Notice:** Since ADOdb 4.05, the session files
have been moved to its own folder, adodb/session. This is a rewrite
of the session code by Ross Smith. The old session code is in
adodb/session/old.

#### ADOdb Session Handler Features

- Ability to define a notification function that is called when a
  session expires. Typically
  used to detect session logout and release global resources.
- Optimization of database writes. We crc32 the session data and
  only perform an update
  to the session data if there is a data change.
- Support for large amounts of session data with CLOBs (see
  adodb-session-clob.php). Useful
  for Oracle.
- Support for encrypted session data, see
  adodb-cryptsession.inc.php. Enabling encryption is simply a matter of
  including adodb-cryptsession.inc.php instead of adodb-session.inc.php.

### Setup

There are 3 session management files that you can use:

```
adodb-session.php        : The default  
adodb-session-clob.php   : Use this if you are storing DATA in clobs  
adodb-cryptsession.php   : Use this if you want to store encrypted session data in the database
```

**Examples**

```
     include('adodb/adodb.inc.php');  
      
    $ADODB_SESSION_DRIVER='mysql';  
    $ADODB_SESSION_CONNECT='localhost';  
    $ADODB_SESSION_USER ='scott';  
    $ADODB_SESSION_PWD ='tiger';  
    $ADODB_SESSION_DB ='sessiondb';  
      
    include('adodb/session/adodb-session.php');  
    session_start();  
      
    #  
    # Test session vars, the following should increment on refresh  
    #  
    $_SESSION['AVAR'] += 1;  
    print "<p>\$_SESSION['AVAR']={$_SESSION['AVAR']}</p>";
```

To force non-persistent connections, call adodb\_session\_open first before session\_start():

```
    include('adodb/adodb.inc.php');  
      
    $ADODB_SESSION_DRIVER='mysql';  
    $ADODB_SESSION_CONNECT='localhost';  
    $ADODB_SESSION_USER ='scott';  
    $ADODB_SESSION_PWD ='tiger';  
    $ADODB_SESSION_DB ='sessiondb';  
      
    include('adodb/session/adodb-session.php');  
    adodb_sess_open(false,false,false);  
    session_start();
```

To use a encrypted sessions, simply replace the file adodb-session.php:

```
    include('adodb/adodb.inc.php');  
      
    $ADODB_SESSION_DRIVER='mysql';  
    $ADODB_SESSION_CONNECT='localhost';  
    $ADODB_SESSION_USER ='scott';  
    $ADODB_SESSION_PWD ='tiger';  
    $ADODB_SESSION_DB ='sessiondb';  
      
    include('adodb/session/adodb-cryptsession.php');  
    session_start();
```

And the same technique for adodb-session-clob.php:

```
    include('adodb/adodb.inc.php');  
      
    $ADODB_SESSION_DRIVER='mysql';  
    $ADODB_SESSION_CONNECT='localhost';  
    $ADODB_SESSION_USER ='scott';  
    $ADODB_SESSION_PWD ='tiger';  
    $ADODB_SESSION_DB ='sessiondb';  
      
    include('adodb/session/adodb-session-clob.php');  
    session_start();
```

#### Installation

1. Create this table in your database (syntax might vary depending on your db):

```
  create table sessions (  
       SESSKEY char(32) not null,  
       EXPIRY int(11) unsigned not null,  
       EXPIREREF varchar(64),  
       DATA text not null,  
      primary key (sesskey)  
  );
```

For the adodb-session-clob.php version, create this:

```
    create table sessions (  
       SESSKEY char(32) not null,  
       EXPIRY int(11) unsigned not null,  
       EXPIREREF varchar(64),  
       DATA CLOB,  
      primary key (sesskey)  
  );
```

2. Then define the following parameters. You can either modify this file, or define them before this file is included:

```
    $ADODB_SESSION_DRIVER='database driver, eg. mysql or ibase';  
    $ADODB_SESSION_CONNECT='server to connect to';  
    $ADODB_SESSION_USER ='user';  
    $ADODB_SESSION_PWD ='password';  
    $ADODB_SESSION_DB ='database';  
    $ADODB_SESSION_TBL = 'sessions'; # setting this is optional  
	
```

When the session is created, $**ADODB\_SESS\_CONN** holds the connection object.  
   
 3. Recommended is PHP 4.0.6 or later. There are documented session bugs in earlier versions of PHP.

### Notifications

If you want to receive notification when a session expires, then tag
the session record with a EXPIREREF tag (see
the definition of the sessions table above). Before any session record
is deleted, ADOdb will call a notification function, passing in the
EXPIREREF.

When a session is first created, we check a global variable
$ADODB\_SESSION\_EXPIRE\_NOTIFY. This is an array with 2 elements, the
first being the name of the session variable you would like to store in
the EXPIREREF field, and the 2nd is the notification function's name.

Suppose we want to be notified when a user's session has expired,
based on the userid. The user id in the global session variable
$USERID. The function name is 'NotifyFn'. So we define:

```
        $ADODB_SESSION_EXPIRE_NOTIFY = array('USERID','NotifyFn');
```

And when the NotifyFn is called (when the session expires), we pass the
$USERID as the first parameter, eg. NotifyFn($userid, $sesskey). The
session key (which is the primary key of the record in the sessions
table) is the 2nd parameter.

Here is an example of a Notification function that deletes some
records in the database and temporary files:

```
        function NotifyFn($expireref, $sesskey)  
        {  
        global $ADODB_SESS_CONN; # the session connection object  
  
          $user = $ADODB_SESS_CONN->qstr($expireref);  
          $ADODB_SESS_CONN->Execute("delete from shopping_cart where user=$user");  
          system("rm /work/tmpfiles/$expireref/*");  
        }
```

NOTE 1: If you have register\_globals disabled in php.ini, then you
will have to manually set the EXPIREREF. E.g.

```
    $GLOBALS['USERID'] =& $_SESSION['USERID'];
    $ADODB_SESSION_EXPIRE_NOTIFY = array('USERID','NotifyFn');
```

NOTE 2: If you want to change the EXPIREREF after the session
record has been created, you will need to modify any session variable
to force a database record update.

#### Neat Notification Tricks

*ExpireRef* normally holds the user id of the current session.

1. You can then write a session monitor, scanning expireref to see
who is currently logged on.

2. If you delete the sessions record for a specific user, eg.

```
delete from sessions where expireref = '$USER'
```

then the user is logged out. Useful for ejecting someone from a
site.

3. You can scan the sessions table to ensure no user
can be logged in twice. Useful for security reasons.

### Compression/Encryption Schemes

Since ADOdb 4.05, thanks to Ross Smith, multiple encryption and
compression schemes are supported. Currently, supported are:

```
  MD5Crypt (crypt.inc.php)  
  MCrypt  
  Secure (Horde's emulation of MCrypt, if MCrypt module is not available.)  
  GZip  
  BZip2
```

These are stackable. E.g.

```
ADODB_Session::filter(new ADODB_Compress_Bzip2());  
ADODB_Session::filter(new ADODB_Encrypt_MD5());
```

will compress and then encrypt the record in the database.

Also see the core ADOdb documentation.
