# Supplementary material for: A UMLS-based spell checker for natural language processing in vaccine safety
Source: BMC Med Inform Decis Mak. 2007 Feb 12;7:3. doi: 10.1186/1472-6947-7-3 (PMC1805499; doi:10.1186/1472-6947-7-3)
Supplement: Additional file 13 — RAP application directory. Sets up directory for RAP (RDF application for PHP) source code [file 1472-6947-7-3-S13.gz › rap/api/util/adodb/docs/old-changelog.htm]

### Old Changelog

**3.50 19 May 2003**

Fixed mssql compat with FreeTDS. FreeTDS does not implement mssql\_fetch\_assoc().

Merged back connection and recordset code into adodb.inc.php.

ADOdb sessions using oracle clobs contributed by achim.gosse#ddd.de. See adodb-session-clob.php.

Added /s modifier to preg\_match everywhere, which ensures that regex does not
stop at /n. Thx Pao-Hsi Huang.

Fixed error in metacolumns() for mssql.

Added time format support for SQLDate.

Image => B added to metatype.

MetaType now checks empty($this->blobSize) instead of empty($this).

Datadict has beta support for informix, sybase (mapped to mssql), db2 and generic
(which is a fudge).

BlobEncode for postgresql uses pg\_escape\_bytea, if available. Needed for compat
with 7.3.

Added $ADODB\_LANG, to support multiple languages in MetaErrorMsg().

Datadict can now parse table definition as declarative text.

For DataDict, oci8 autoincrement trigger missing semi-colon. Fixed.

For DataDict, when REPLACE flag enabled, drop sequence in datadict for autoincrement
field in postgres and oci8.s

Postgresql defaults to template1 database if no database defined in connect/pconnect.

We now clear \_resultid in postgresql if query fails.

**3.40 19 May 2003**

Added insert\_id for odbc\_mssql.

Modified postgresql UpdateBlobFile() because it did not work in safe mode.

Now connection object is passed to raiseErrorFn as last parameter. Needed by
StartTrans().

Added StartTrans() and CompleteTrans(). It is recommended that you do not modify
transOff, but use the above functions.

oci8po now obeys ADODB\_ASSOC\_CASE settings.

Added virtualized error codes, using PEAR DB equivalents. Requires you to manually
include adodb-error.inc.php yourself, with MetaError() and MetaErrorMsg($errno).

GetRowAssoc for mysql and pgsql were flawed. Fix by Ross Smith.

Added to datadict types I1, I2, I4 and I8. Changed datadict type 'T' to map
to timestamp instead of datetime for postgresql.

Error handling in ExecuteSQLArray(), adodb-datadict.inc.php did not work.

We now auto-quote postgresql connection parameters when building connection
string.

Added session expiry notification.

We now test with odbc mysql - made some changes to odbc recordset constructor.

MetaColumns now special cases access and other databases for odbc.

**3.31 17 March 2003**

Added row checking for \_fetch in postgres.

Added Interval type to MetaType for postgres.

Remapped postgres driver to call postgres7 driver internally.

Adorecordset\_array::getarray() did not return array when nRows >= 0.

Postgresql: at times, no error message returned by pg\_result\_error() but error
message returned in pg\_last\_error(). Recoded again.

Interbase blob's now use chunking for updateblob.

Move() did not set EOF correctly. Reported by Jorma T.

We properly support mysql timestamp fields when we are creating mysql tables
using the data-dict interface.

Table regex includes backticks character now.

**3.30 3 March 2003**

Added $ADODB\_EXTENSION and $ADODB\_COMPAT\_FETCH constant.

Made blank1stItem configurable using syntax "value:text" in GetMenu/GetMenu2.
Thx to Gabriel Birke.

Previously ADOdb differed from the Microsoft standard because it did not define
what to set $this->fields when EOF was reached. Now at EOF, ADOdb sets $this->fields
to false for all databases, which is consist with Microsoft's implementation.
Postgresql and mysql have always worked this way (in 3.11 and earlier). If you
are experiencing compatibility problems (and you are not using postgresql nor
mysql) on upgrading to 3.30, try setting the global variables $ADODB\_COUNTRECS
= true (which is the default) and $ADODB\_FETCH\_COMPAT = true (this is a new
global variable).

We now check both pg\_result\_error and pg\_last\_error as sometimes pg\_result\_error
does not display anything. Iman Mayes

We no longer check for magic quotes gpc in Quote().

Misc fixes for table creation in adodb-datadict.inc.php. Thx to iamsure.

Time calculations use adodb\_time library for all negative timestamps due to
problems in Red Hat 7.3 or later. Formerly, only did this for Windows.

In mssqlpo, we now check if $sql in \_query is a string before we change ||
to +. This is to support prepared stmts.

Move() and MoveLast() internals changed to support to support EOF and $this->fields
change.

Added ADODB\_FETCH\_BOTH support to mssql. Thx to Angel Fradejas afradejas#mediafusion.es

We now check if link resource exists before we run mysql\_escape\_string in
qstr().

Before we flock in csv code, we check that it is not a http url.

**3.20 17 Feb 2003**

Added new Data Dictionary classes for creating tables and indexes. Warning
- this is very much alpha quality code. The API can still change. See adodb/tests/test-datadict.php
for more info.

We now ignore $ADODB\_COUNTRECS for mysql, because PHP truncates incomplete
recordsets when mysql\_unbuffered\_query() is called a second time.

Now postgresql works correctly when $ADODB\_COUNTRECS = false.

Changed \_adodb\_getcount to properly support SELECT DISTINCT.

Discovered that $ADODB\_COUNTRECS=true has some problems with prepared queries
- suspect PHP bug.

Now GetOne and GetRow run in $ADODB\_COUNTRECS=false mode for better performance.

Added support for mysql\_real\_escape\_string() and pg\_escape\_string() in qstr().

Added an intermediate variable for mysql \_fetch() and MoveNext() to store fields,
to prevent overwriting field array with boolean when mysql\_fetch\_array() returns
false.

Made arrays for getinsertsql and getupdatesql case-insensitive. Suggested by
Tim Uckun" tim#diligence.com

**3.11 11 Feb 2003**

Added check for ADODB\_NEVER\_PERSIST constant in PConnect(). If defined, then
PConnect() will actually call non-persistent Connect().

Modified interbase to properly work with Prepare().

Added $this->ibase\_timefmt to allow you to change the date and time format.

Added support for $input\_array parameter in CacheFlush().

Added experimental support for dbx, which was then removed when i found that
it was slower than using native calls.

Added MetaPrimaryKeys for mssql and ibase/firebird.

Added new $trim parameter to GetCol and CacheGetCol

Uses updated adodb-time.inc.php 0.06.

**3.10 27 Jan 2003**

Added adodb\_date(), adodb\_getdate(), adodb\_mktime() and adodb-time.inc.php.

For interbase, added code to handle unlimited number of bind parameters. From
Daniel Hasan daniel#hasan.cl.

Added BlobDecode and UpdateBlob for informix. Thx to Fernando Ortiz.

Added constant ADODB\_WINDOWS. If defined, means that running on Windows.

Added constant ADODB\_PHPVER which stores php version as a hex num. Removed
$ADODB\_PHPVER variable.

Felho Bacsi reported a minor white-space regular expression problem in GetInsertSQL.

Modified ADO to use variant to store \_affectedRows

Changed ibase to use base class Replace(). Modified base class Replace() to
support ibase.

Changed odbc to auto-detect when 0 records returned is wrong due to bad odbc
drivers.

Changed mssql to use datetimeconvert ini setting only when 4.30 or later (does
not work in 4.23).

ExecuteCursor($stmt, $cursorname, $params) now accepts a new $params array
of additional bind parameters -- William Lovaton walovaton#yahoo.com.mx.

Added support for sybase\_unbuffered\_query if ADODB\_COUNTRECS == false. Thx
to chuck may.

Fixed FetchNextObj() bug. Thx to Jorma Tuomainen.

We now use SCOPE\_IDENTITY() instead of @@IDENTITY for mssql - thx to marchesini#eside.it

Changed postgresql movenext logic to prevent illegal row number from being
passed to pg\_fetch\_array().

Postgresql initrs bug found by "Bogdan RIPA" bripa#interakt.ro $f1 accidentally
named $f

**3.00 6 Jan 2003**

Fixed adodb-pear.inc.php syntax error.

Improved \_adodb\_getcount() to use SELECT COUNT(\*) FROM ($sql) for languages
that accept it.

Fixed \_adodb\_getcount() caching error.

Added sql to retrive table and column info for odbc\_mssql.

**2.91 3 Jan 2003**

Revised PHP version checking to use $ADODB\_PHPVER with legal values 0x4000,
0x4050, 0x4200, 0x4300.

Added support for bytea fields and oid blobs in postgres by allowing BlobDecode()
to detect and convert non-oid fields. Also added BlobEncode to postgres when
you want to encode oid blobs.

Added blobEncodeType property for connections to inform phpLens what encoding
method to use for blobs.

Added BlobDecode() and BlobEncode() to base ADOConnection class.

Added umask() to \_gencachename() when creating directories.

Added charPage for ado drivers, so you can set the code page.

```
$conn->charPage = CP_UTF8;
$conn->Connect($dsn);
```

Modified \_seek in mysql to check for num rows=0.

Added to metatypes new informix types for IDS 9.30. Thx Fernando Ortiz.

\_maxrecordcount returned in CachePageExecute $rsreturn

Fixed sybase cacheselectlimit( ) problems

MetaColumns() max\_length should use precision for types X and C for ms access.
Fixed.

Speedup of odbc non-SELECT sql statements.

Added support in MetaColumns for Wide Char types for ODBC. We halve max\_length
if unicode/wide char.

Added 'B' to types handled by GetUpdateSQL/GetInsertSQL.

Fixed warning message in oci8 driver with $persist variable when using PConnect.

**2.90 11 Dec 2002**

Mssql and mssqlpo and oci8po now support ADODB\_ASSOC\_CASE.

Now MetaType() can accept a field object as the first parameter.

New $arr = $db->ServerInfo( ) function. Returns $arr['description'] which
is the string description, and $arr['version'].

PostgreSQL and MSSQL speedups for insert/updates.

Implemented new SetFetchMode() that removes the need to use $ADODB\_FETCH\_MODE.
Each connection has independant fetchMode.

ADODB\_ASSOC\_CASE now defaults to 2, use native defaults. This is because we
would break backward compat for too many applications otherwise.

Patched encrypted sessions to use replace()

The qstr function supports quoting of nulls when escape character is \

Rewrote bits and pieces of session code to check for time synch and improve
reliability.

Added property ADOConnection::hasTransactions = true/false;

Added CreateSequence and DropSequence functions

Found misplaced MoveNext() in adodb-postgres.inc.php. Fixed.

Sybase SelectLimit not reliable because 'set rowcount' not cached - fixed.

Moved ADOConnection to adodb-connection.inc.php and ADORecordSet to adodb-recordset.inc.php.
This allows us to use doxygen to generate documentation. Doxygen doesn't like
the classes in the main adodb.inc.php file for some mysterious reason.

**2.50, 14 Nov 2002**

Added transOff and transCnt properties for disabling (transOff = true) and
tracking transaction status (transCnt>0).

Added inputarray handling into \_adodb\_pageexecute\_all\_rows - "Ross Smith" RossSmith#bnw.com.

Fixed postgresql inconsistencies in date handling.

Added support for mssql\_fetch\_assoc.

Fixed $ADODB\_FETCH\_MODE bug in odbc MetaTables() and MetaPrimaryKeys().

Accidentally declared UnixDate() twice, making adodb incompatible with php
4.3.0. Fixed.

Fixed pager problems with some databases that returned -1 for \_currentRow on
MoveLast() by switching to MoveNext() in adodb-lib.inc.php.

Also fixed uninited $discard in adodb-lib.inc.php.

**2.43, 25 Oct 2002**

Added ADODB\_ASSOC\_CASE constant to better support ibase and odbc field names.

Added support for NConnect() for oracle OCINLogin.

Fixed NumCols() bug.

Changed session handler to use Replace() on write.

Fixed oci8 SelectLimit aggregate function bug again.

Rewrote pivoting code.

**2.42, 4 Oct 2002**

Fixed ibase\_fetch() problem with nulls. Also interbase now does automatic blob
decoding, and is backward compatible. Suggested by Heinz Hombergs heinz#hhombergs.de.

Fixed postgresql MoveNext() problems when called repeatedly after EOF. Also
suggested by Heinz Hombergs.

PageExecute() does not rewrite queries if SELECT DISTINCT is used. Requested
by hans#velum.net

Added additional fixes to oci8 SelectLimit handling with aggregate functions
- thx to Christian Bugge for reporting the problem.

**2.41, 2 Oct 2002**

Fixed ADODB\_COUNTRECS bug in odbc. Thx to Joshua Zoshi jzoshi#hotmail.com.

Increased buffers for adodb-csvlib.inc.php for extremely long sql from 8192
to 32000.

Revised pivottable.inc.php code. Added better support for aggregate fields.

Fixed mysql text/blob types problem in MetaTypes base class - thx to horacio
degiorgi.

Added SQLDate($fmt,$date) function, which allows an sql date format string
to be generated - useful for group by's.

Fixed bug in oci8 SelectLimit when offset>100.

**2.40 4 Sept 2002**

Added new NLS\_DATE\_FORMAT property to oci8. Suggested by Laurent NAVARRO ln#altidev.com

Now use bind parameters in oci8 selectlimit for better performance.

Fixed interbase replaceQuote for dialect != 1. Thx to "BEGUIN Pierre-Henri
- INFOCOB" phb#infocob.com.

Added white-space check to QA.

Changed unixtimestamp to support fractional seconds (we always round down/floor
the seconds). Thanks to beezly#beezly.org.uk.

Now you can set the trigger\_error type your own user-defined type in adodb-errorhandler.inc.php.
Suggested by Claudio Bustos clbustos#entelchile.net.

Added recordset filters with rsfilter.inc.php.

$conn->\_rs2rs does not create a new recordset when it detects it is of type
array. Some trickery there as there seems to be a bug in Zend Engine

Added render\_pagelinks to adodb-pager.inc.php. Code by "Pablo Costa" pablo#cbsp.com.br.

MetaType() speedup in adodb.inc.php by using hashing instead of switch. Best
performance if constant arrays are supported, as they are in PHP5.

adodb-session.php now updates only the expiry date if the crc32 check indicates
that the data has not been modified.

**2.31 20 Aug 2002**

Made changes to pivottable.inc.php due to daniel lucuzaeu's suggestions (we sum the pivottable column if desired).

Fixed ErrorNo() in postgres so it does not depend on \_errorMsg property.

Robert Tuttle added support for oracle cursors. See ExecuteCursor().

Fixed Replace() so it works with mysql when updating record where data has not changed. Reported by
Cal Evans (cal#calevans.com).

**2.30 1 Aug 2002**

Added pivottable.inc.php. Thanks to daniel.lucazeau#ajornet.com for the original
concept.

Added ADOConnection::outp($msg,$newline) to output error and debugging messages. Now
you can override this using the ADODB\_OUTP constant and use your own output handler.

Changed == to === for 'null' comparison. Reported by ericquil#yahoo.com

Fixed mssql SelectLimit( ) bug when distinct used.

**2.30 1 Aug 2002**

New GetCol() and CacheGetCol() from ross#bnw.com that returns the first field as a 1 dim array.

We have an empty recordset, but RecordCount() could return -1. Fixed. Reported by "Jonathan Polansky" jonathan#polansky.com.

We now check for session variable changes using strlen($sessval).crc32($sessval).
Formerly we only used crc32().

Informix SelectLimit() problem with $ADODB\_COUNTRECS fixed.

Fixed informix SELECT FIRST x DISTINCT, and not SELECT DISTINCT FIRST x - reported by F Riosa

Now default adodb error handlers ignores error if @ used.

If you set $conn->autoRollback=true, we auto-rollback persistent connections for odbc, mysql, oci8, mssql.
Default for autoRollback is false. No need to do so for postgres.
As interbase requires a transaction id (what a flawed api), we don't do it for interbase.

Changed PageExecute() to use non-greedy preg\_match when searching for "FROM" keyword.

**2.20 9 July 2002**

Added CacheGetOne($secs2cache,$sql), CacheGetRow($secs2cache,$sql), CacheGetAll($secs2cache,$sql).

Added $conn->OffsetDate($dayFraction,$date=false) to generate sql that calcs
date offsets. Useful for scheduling appointments.

Added connection properties: leftOuter, rightOuter that hold left and right
outer join operators.

Added connection property: ansiOuter to indicate whether ansi outer joins supported.

New driver *mssqlpo*, the portable mssql driver, which converts string
concat operator from || to +.

Fixed ms access bug - SelectLimit() did not support ties - fixed.

Karsten Kraus (Karsten.Kraus#web.de), contributed error-handling code to ADONewConnection.
Unfortunately due to backward compat problems, had to rollback most of the changes.

Added new parameter to GetAssoc() to allow returning an array of key-value pairs,
ignoring any additional columns in the recordset. Off by default.

Corrected mssql $conn->sysDate to return only date using convert().

CacheExecute() improved debugging output.

Changed rs2html() so newlines are converted to BR tags. Also optimized rs2html() based
on feedback by "Jerry Workman" jerry#mtncad.com.

Added support for Replace() with Interbase, using DELETE and INSERT.

Some minor optimizations (mostly removing & references when passing arrays).

Changed GenID() to allows id's larger than the size of an integer.

Added force\_session property to oci8 for better updateblob() support.

Fixed PageExecute() which did not work properly with sql containing GROUP BY.

**2.12 12 June 2002**

Added toexport.inc.php to export recordsets in CSV and tab-delimited format.

CachePageExecute() does not work - fixed - thx John Huong.

Interbase aliases not set properly in FetchField() - fixed. Thx Stefan Goethals.

Added cache property to adodb pager class. The number of secs to cache recordsets.

SQL rewriting bug in pageexecute() due to skipping of newlines due to missing /s modifier. Fixed.

Max size of cached recordset due to a bug was 256000 bytes. Fixed.

Speedup of 1st invocation of CacheExecute() by tuning code.

We compare $rewritesql with $sql in pageexecute code in case of rewrite failure.

**2.11 7 June 2002**

Fixed PageExecute() rewrite sql problem - COUNT(\*) and ORDER BY don't go together with
mssql, access and postgres. Thx to Alexander Zhukov alex#unipack.ru

DB2 support for CHARACTER type added - thx John Huong huongch#bigfoot.com

For ado, $argProvider not properly checked. Fixed - kalimero#ngi.it

Added $conn->Replace() function for update with automatic insert if the record does not exist.
Supported by all databases except interbase.

**2.10 4 June 2002**

Added uniqueSort property to indicate mssql ORDER BY cols must be unique.

Optimized session handler by crc32 the data. We only write if session data has changed.

adodb\_sess\_read in adodb-session.php now returns ''correctly - thanks to Jorma Tuomainen, webmaster#wizactive.com

Mssql driver did not throw EXECUTE errors correctly because ErrorMsg() and ErrorNo() called in wrong order.
Pointed out by Alexios Fakos. Fixed.

Changed ado to use client cursors. This fixes BeginTran() problems with ado.

Added handling of timestamp type in ado.

Added to ado\_mssql support for insert\_id() and affected\_rows().

Added support for mssql.datetimeconvert=0, available since php 4.2.0.

Made UnixDate() less strict, so that the time is ignored if present.

Changed quote() so that it checks for magic\_quotes\_gpc.

Changed maxblobsize for odbc to default to 64000.

**2.00 13 May 2002**

Added drivers *informix72* for pre-7.3 versions, and *oci805* for
oracle 8.0.5, and postgres64 for postgresql 6.4 and earlier. The postgres and postgres7 drivers
are now identical.

Interbase now partially supports ADODB\_FETCH\_BOTH, by defaulting to ASSOC mode.

Proper support for blobs in mssql. Also revised blob support code
is base class. Now UpdateBlobFile() calls UpdateBlob() for consistency.

Added support for changed odbc\_fetch\_into api in php 4.2.0
with $conn->\_has\_stupid\_odbc\_fetch\_api\_change.

Fixed spelling of tablock locking hint in GenID( ) for mssql.

Added RowLock( ) to several databases, including oci8, informix, sybase, etc.
Fixed where error in mssql RowLock().

Added sysDate and sysTimeStamp properties to most database drivers. These are the sql
functions/constants for that database that return the current date and current timestamp, and
are useful for portable inserts and updates.

Support for RecordCount() caused date handling in sybase and mssql to break.
Fixed, thanks to Toni Tunkkari, by creating derived classes for ADORecordSet\_array for
both databases. Generalized using arrayClass property. Also to support RecordCount(),
changed metatype handling for ado drivers. Now the type returned in FetchField
is no longer a number, but the 1-char data type returned by MetaType.
At the same time, fixed a lot of date handling. Now mssql support dmy and mdy date formats.
Also speedups in sybase and mssql with preg\_match and ^ in date/timestamp handling.
Added support in sybase and mssql for 24 hour clock in timestamps (no AM/PM).

Extensive revisions to informix driver - thanks to Samuel CARRIERE samuel\_carriere#hotmail.com

Added $ok parameter to CommitTrans($ok) for easy rollbacks.

Fixed odbc MetaColumns and MetaTables to save and restore $ADODB\_FETCH\_MODE.

Some odbc drivers did not call the base connection class constructor. Fixed.

Fixed regex for GetUpdateSQL() and GetInsertSQL() to support more legal character combinations.

**1.99 21 April 2002**

Added emulated RecordCount() to all database drivers if $ADODB\_COUNTRECS = true
(which it is by default). Inspired by Cristiano Duarte (cunha17#uol.com.br).

Unified stored procedure support for mssql and oci8. Parameter() and PrepareSP()
functions implemented.

Added support for SELECT FIRST in informix, modified hasTop property to support
this.

Changed csv driver to handle updates/deletes/inserts properly (when Execute() returns true).
Bind params also work now, and raiseErrorFn with csv driver. Added csv driver to QA process.

Better error checking in oci8 UpdateBlob() and UpdateBlobFile().

Added TIME type to MySQL - patch by Manfred h9125297#zechine.wu-wien.ac.at

Prepare/Execute implemented for Interbase/Firebird

Changed some regular expressions to be anchored by /^ $/ for speed.

Added UnixTimeStamp() and UnixDate() to ADOConnection(). Now these functions
are in both ADOConnection and ADORecordSet classes.

Empty recordsets were not cached - fixed.

Thanks to Gaetano Giunta (g.giunta#libero.it) for the oci8 code review. We
didn't agree on everything, but i hoped we agreed to disagree!

**1.90 6 April 2002**

Now all database drivers support fetch modes ADODB\_FETCH\_NUM and ADODB\_FETCH\_ASSOC, though
still not fully tested. Eg. Frontbase, Sybase, Informix.

NextRecordSet() support for mssql. Contributed by "Sven Axelsson" sven.axelsson#bokochwebb.se

Added blob support for SQL Anywhere. Contributed by Wade Johnson wade#wadejohnson.de

Fixed some security loopholes in server.php. Server.php also supports fetch mode.

Generalized GenID() to support odbc and mssql drivers. Mssql no longer generates GUID's.

Experimental RowLock($table,$where) for mssql.

Properly implemented Prepare() in oci8 and ODBC.

Added Bind() support to oci8 to support Prepare().

Improved error handler. Catches CacheExecute() and GenID() errors now.

Now if you are running php from the command line, debugging messages do not output html formating.
Not 100% complete, but getting there.

**1.81 22 March 2002**

Restored default $ADODB\_FETCH\_MODE = ADODB\_FETCH\_DEFAULT for backward compatibility.

SelectLimit for oci8 improved - Our FIRST\_ROWS optimization now does not overwrite existing hint.

New Sybase SQL Anywhere driver. Contributed by Wade Johnson wade#wadejohnson.de

**1.80 15 March 2002**

Redesigned directory structure of ADOdb files. Added new driver directory where
all database drivers reside.

Changed caching algorithm to create subdirectories. Now we scale better.

Informix driver now supports insert\_id(). Contribution by "Andrea Pinnisi" pinnisi#sysnet.it

Added experimental ISO date and FetchField support for informix.

Fixed a quoting bug in Execute() with bind parameters, causing problems with blobs.

Mssql driver speedup by 10-15%.

Now in CacheExecute($secs2cache,$sql,...), $secs2cache is optional. If missing, it will
take the value defined in $connection->cacheSecs (default is 3600 seconds). Note that
CacheSelectLimit(), the secs2cache is still compulsory - sigh.

Sybase SQL Anywhere driver (using ODBC) contributed by Wade Johnson wade#wadejohnson.de

**1.72 8 March 2002**

Added @ when returning Fields() to prevent spurious error - "Michael William Miller" mille562#pilot.msu.edu

MetaDatabases() for postgres contributed by Phil pamelant#nerim.net

Mitchell T. Young (mitch#youngfamily.org) contributed informix driver.

Fixed rs2html() problem. I cannot reproduce, so probably a problem with pre PHP 4.1.0 versions,
when supporting new ADODB\_FETCH\_MODEs.

Mattia Rossi (mattia#technologist.com) contributed BlobDecode() and UpdateBlobFile() for postgresql
using the postgres specific pg\_lo\_import()/pg\_lo\_open() - i don't use them but hopefully others will
find this useful. See this posting
for an example of usage.

Added UpdateBlobFile() for uploading files to a database.

Made UpdateBlob() compatible with oci8po driver.

Added noNullStrings support to oci8 driver. Oracle changes all ' ' strings to nulls,
so you need to set strings to ' ' to prevent the nullifying of strings. $conn->noNullStrings = true;
will do this for you automatically. This is useful when you define a char column as NOT NULL.

Fixed UnixTimeStamp() bug - wasn't setting minutes and seconds properly. Patch from Agusti Fita i Borrell agusti#anglatecnic.com.

Toni Tunkkari added patch for sybase dates. Problem with spaces in day part of date fixed.

**1.71 18 Jan 2002**

Sequence start id support. Now $conn->Gen\_ID('seqname', 50) to start sequence from 50.

CSV driver fix for selectlimit, from Andreas - akaiser#vocote.de.

Gam3r spotted that a global variable was undefined in the session handler.

Mssql date regex had error. Fixed - reported by Minh Hoang vb\_user#yahoo.com.

DBTimeStamp() and DBDate() now accept iso dates and unix timestamps. This means
that the PostgreSQL handling of dates in GetInsertSQL() and GetUpdateSQL() can
be removed. Also if these functions are passed '' or null or false, we return a SQL null.

GetInsertSQL() and GetUpdateSQL() now accept a new parameter, $magicq to
indicate whether quotes should be inserted based on magic quote settings - suggested by
dj#4ict.com.

Reformated docs slightly based on suggestions by Chris Small.

**1.65 28 Dec 2001**

Fixed borland\_ibase class naming bug.

Now instead of using $rs->fields[0] internally, we use reset($rs->fields) so
that we are compatible with ADODB\_FETCH\_ASSOC mode. Reported by Nico S.

Changed recordset constructor and \_initrs() for oci8 so that it returns the field definitions even
if no rows in the recordset. Reported by Rick Hickerson (rhickers#mv.mv.com).

Improved support for postgresql in GetInsertSQL and GetUpdateSQL by
"mike" mike#partner2partner.com and "Ryan Bailey" rebel#windriders.com

**1.64 20 Dec 2001**

Danny Milosavljevic <danny.milo#gmx.net> added some patches for MySQL error handling
and displaying default values.

Fixed some ADODB\_FETCH\_BOTH inconsistencies in odbc and interbase.

Added more tests to test suite to cover ADODB\_FETCH\_\* and ADODB\_ERROR\_HANDLER.

Added firebird (ibase) driver

Added borland\_ibase driver for interbase 6.5

**1.63 13 Dec 2001**

Absolute to the adodb-lib.inc.php file not set properly. Fixed.

**1.62 11 Dec 2001**

Major speedup of ADOdb for low-end web sites by reducing the php code loading and compiling
cycle. We conditionally compile not so common functions.
Moved csv code to adodb-csvlib.inc.php to reduce adodb.inc.php parsing. This file
is loaded only when the csv/proxy driver is used, or CacheExecute() is run.
Also moved PageExecute(), GetSelectSQL() and GetUpdateSQL() core code to adodb-lib.inc.php.
This reduced the 70K main adodb.inc.php file to 55K, and since at least 20K of the file
is comments, we have reduced 50K of code in adodb.inc.php to 35K. There
should be 35% reduction in memory and thus 35% speedup in compiling the php code for the
main adodb.inc.php file.

Highly tuned SelectLimit() for oci8 for massive speed improvements on large files.
Selecting 20 rows starting from the 20,000th row of a table is now 7 times faster.
Thx to Tomas V V Cox.

Allow . and # in table definitions in GetInsertSQL and GetUpdateSQL.
See ADODB\_TABLE\_REGEX constant. Thx to Ari Kuorikoski.

Added ADODB\_PREFETCH\_ROWS constant, defaulting to 10. This determines the number
of records to prefetch in a SELECT statement. Only used by oci8.

Added high portability Oracle class called oci8po. This uses ? for bind variables, and
lower cases column names.

Now all database drivers support $ADODB\_FETCH\_MODE, including interbase, ado, and odbc:
ADODB\_FETCH\_NUM and ADODB\_FETCH\_ASSOC. ADODB\_FETCH\_BOTH is not fully implemented for all
database drivers.

**1.61 Nov 2001**

Added PO\_RecordCount() and PO\_Insert\_ID(). PO stands for portable. Pablo Roca
[pabloroca#mvps.org]

GenID now returns 0 if not available. Safer is that you should check $conn->hasGenID
for availability.

M'soft ADO we now correctly close recordset in \_close() peterd#telephonetics.co.uk

MSSQL now supports GenID(). It generates a 16-byte GUID from mssql newid()
function.

Changed ereg\_replace to preg\_replace in SelectLimit. This is a fix for mssql.
Ereg doesn't support t or n! Reported by marino Carlos xaplo#postnuke-espanol.org

Added $recordset->connection. This is the ADOConnection object for the recordset.
Works with cached and normal recordsets. Surprisingly, this had no affect on performance!

**1.54 15 Nov 2001**

Fixed some more bugs in PageExecute(). I am getting sick of bug in this and will have to
reconsider my QA here. The main issue is that I don't use PageExecute() and
to check whether it is working requires a visual inspection of the html generated currently.
It is possible to write a test script but it would be quite complicated :(

More speedups of SelectLimit() for DB2, Oci8, access, vfp, mssql.

**1.53 7 Nov 2001**

Added support for ADODB\_FETCH\_ASSOC for ado and odbc drivers.

Tuned GetRowAssoc(false) in postgresql and mysql.

Stephen Van Dyke contributed ADOdb icon, accepted with some minor mods.

Enabled Affected\_Rows() for postgresql

Speedup for Concat() using implode() - Benjamin Curtis ben\_curtis#yahoo.com

Fixed some more bugs in PageExecute() to prevent infinite loops

**1.52 5 Nov 2001**

Spelling error in CacheExecute() caused it to fail. $ql should be $sql in line 625!

Added fixes for parsing [ and ] in GetUpdateSQL().

**1.51 5 Nov 2001**

Oci8 SelectLimit() speedup by using OCIFetch().

Oci8 was mistakenly reporting errors when $db->debug = true.

If a connection failed with ODBC, it was not correctly reported - fixed.

\_connectionID was inited to -1, changed to false.

Added $rs->FetchRow(), to simplify API, ala PEAR DB

Added PEAR DB compat mode, which is still faster than PEAR! See adodb-pear.inc.php.

Removed postgres pconnect debugging statement.

**1.50 31 Oct 2001**

ADOdbConnection renamed to ADOConnection, and ADOdbFieldObject to ADOFieldObject.

PageExecute() now checks for empty $rs correctly, and the errors in the docs on this subject have been fixed.

odbc\_error() does not return 6 digit error correctly at times. Implemented workaround.

Added ADORecordSet\_empty class. This will speedup INSERTS/DELETES/UPDATES because the return
object created is much smaller.

Added Prepare() to odbc, and oci8 (but doesn't work properly for oci8 still).

Made pgsql a synonym for postgre7, and changed SELECT LIMIT to use OFFSET for compat with
postgres 7.2.

Revised adodb-cryptsession.php thanks to Ari.

Set resources to false on \_close, to force freeing of resources.

Added adodb-errorhandler.inc.php, adodb-errorpear.inc.php and raiseErrorFn on Freek's urging.

GetRowAssoc($toUpper=true): $toUpper added as default.

Errors when connecting to a database were not captured formerly. Now we do it correctly.

**1.40 19 September 2001**

PageExecute() to implement page scrolling added. Code and idea by Iván Oliva.

Some minor postgresql fixes.

Added sequence support using GenID() for postgresql, oci8, mysql, interbase.

Added UpdateBlob support for interbase (untested).

Added encrypted sessions (see adodb-cryptsession.php). By Ari Kuorikoski <kuoriari#finebyte.com>

**1.31 21 August 2001**

Many bug fixes thanks to "GaM3R (Cameron)" <gamr#outworld.cx>. Some session changes due to Gam3r.

Fixed qstr() to quote also.

rs2html() now pretty printed.

Jonathan Younger jyounger#unilab.com contributed the great idea GetUpdateSQL() and GetInsertSQL() which
generates SQL to update and insert into a table from a recordset. Modify the recordset fields
array, then can this function to generate the SQL (the SQL is not executed).

"Nicola Fankhauser" <nicola.fankhauser#couniq.com> found some bugs in date handling for mssql.

Added minimal Oracle support for LOBs. Still under development.

Added $ADODB\_FETCH\_MODE so you can control whether recordsets return arrays which are
numeric, associative or both. This is a global variable you set. Currently only MySQL, Oci8, Postgres
drivers support this.

PostgreSQL properly closes recordsets now. Reported by several people.

Added UpdateBlob() for Oracle. A hack to make it easier to save blobs.

Oracle timestamps did not display properly. Fixed.

**1.20 6 June 2001**

Now Oracle can connect using tnsnames.ora or server and service name

Extensive Oci8 speed optimizations.
Oci8 code revised to support variable binding, and /\*+ FIRST\_ROWS \*/ hint.

Worked around some 4.0.6 bugs in odbc\_fetch\_into().

Paolo S. Asioli paolo.asioli#libero.it suggested GetRowAssoc().

Escape quotes for oracle wrongly set to '. Now '' is used.

Variable binding now works in ODBC also.

Jumped to version 1.20 because I don't like 13 :-)

**1.12 6 June 2001**

Changed $ADODB\_DIR to ADODB\_DIR constant to plug a security loophole.

Changed \_close() to close persistent connections also. Prevents connection leaks.

Major revision of oracle and oci8 drivers.
Added OCI\_RETURN\_NULLS and OCI\_RETURN\_LOBS to OCIFetchInto(). BLOB, CLOB and VARCHAR2 recognition
in MetaType() improved. MetaColumns() returns columns in correct sort order.

Interbase timestamp input format was wrong. Fixed.

**1.11 20 May 2001**

Improved file locking for Windows.

Probabilistic flushing of cache to avoid avalanche updates when cache timeouts.

Cached recordset timestamp not saved in some scenarios. Fixed.

**1.10 19 May 2001**

Added caching. CacheExecute() and CacheSelectLimit().

Added csv driver. See http://php.weblogs.com/ADODB\_csv.

Fixed SelectLimit(), SELECT TOP not working under certain circumstances.

Added better Frontbase support of MetaTypes() by Frank M. Kromann.

**1.01 24 April 2001**

Fixed SelectLimit bug. not quoted properly.

SelectLimit: SELECT TOP -1 \* FROM TABLE not support by Microsoft. Fixed.

GetMenu improved by glen.davies#cce.ac.nz to support multiple hilited items

FetchNextObject() did not work with only 1 record returned. Fixed bug reported by $tim#orotech.net

Fixed mysql field max\_length problem. Fix suggested by Jim Nicholson (jnich#att.com)

**1.00 16 April 2001**

Given some brilliant suggestions on how to simplify ADOdb by akul. You no longer need to
setup $ADODB\_DIR yourself, and ADOLoadCode() is automatically called by ADONewConnection(),
simplifying the startup code.

FetchNextObject() added. Suggested by Jakub Marecek. This makes FetchObject() obsolete, as
this is more flexible and powerful.

Misc fixes to SelectLimit() to support Access (top must follow distinct) and Fields()
in the array recordset. From Reinhard Balling.

**0.96 27 Mar 2001**

ADOConnection Close() did not return a value correctly. Thanks to akul#otamedia.com.

When the horrible magic\_quotes is enabled, back-slash () is changed to double-backslash (\).
This doesn't make sense for Microsoft/Sybase databases. We fix this in qstr().

Fixed Sybase date problem in UnixDate() thanks to Toni Tunkkari. Also fixed MSSQL problem
in UnixDate() - thanks to milhouse31#hotmail.com.

MoveNext() moved to leaf classes for speed in MySQL/PostgreSQL. 10-15% speedup.

Added null handling in bindInputArray in Execute() -- Ron Baldwin suggestion.

Fixed some option tags. Thanks to john#jrmstudios.com.

**0.95 13 Mar 2001**

Added postgres7 database driver which supports LIMIT and other version 7 stuff in the future.

Added SelectLimit to ADOConnection to simulate PostgreSQL's "select \* from table limit 10 offset 3".
Added helper function GetArrayLimit() to ADORecordSet.

Fixed mysql metacolumns bug. Thanks to Freek Dijkstra (phpeverywhere#macfreek.com).

Also many PostgreSQL changes by Freek. He almost rewrote the whole PostgreSQL driver!

Added fix to input parameters in Execute for non-strings by Ron Baldwin.

Added new metatype, X for TeXt. Formerly, metatype B for Blob also included
text fields. Now 'B' is for binary/image data. 'X' for textual data.

Fixed $this->GetArray() in GetRows().

Oracle and OCI8: 1st parameter is always blank -- now warns if it is filled.

Now *hasLimit* and *hasTop* added to indicate whether
SELECT \* FROM TABLE LIMIT 10 or SELECT TOP 10 \* FROM TABLE are supported.

**0.94 04 Feb 2001**

Added ADORecordSet::GetRows() for compatibility with Microsoft ADO. Synonym for GetArray().

Added new metatype 'R' to represent autoincrement numbers.

Added ADORecordSet.FetchObject() to return a row as an object.

Finally got a Linux box to test PostgreSql. Many fixes.

Fixed copyright misspellings in 0.93.

Fixed mssql MetaColumns type bug.

Worked around odbc bug in PHP4 for sessions.

Fixed many documentation bugs (affected\_rows, metadatabases, qstr).

Fixed MySQL timestamp format (removed comma).

Interbase driver did not call ibase\_pconnect(). Fixed.

**0.93 18 Jan 2002**

Fixed GetMenu bug.

Simplified Interbase commit and rollback.

Default behaviour on closing a connection is now to rollback all active transactions.

Added field object handling for array recordset for future XML compatibility.

Added arr2html() to convert array to html table.

**0.92 2 Jan 2002**

Interbase Commit and Rollback should be working again.

Changed initialisation of ADORecordSet. This is internal and should not affect users. We
are doing this to support cached recordsets in the future.

Implemented ADORecordSet\_array class. This allows you to simulate a database recordset
with an array.

Added UnixDate() and UnixTimeStamp() to ADORecordSet.

**0.91 21 Dec 2000**

Fixed ODBC so ErrorMsg() is working.

Worked around ADO unrecognised null (0x1) value problem in COM.

Added Sybase support for FetchField() type

Removed debugging code and unneeded html from various files

Changed to javadoc style comments to adodb.inc.php.

Added maxsql as synonym for mysqlt

Now ODBC downloads first 8K of blob by default

**0.90 15 Nov 2000**

Lots of testing of Microsoft ADO. Should be more stable now.

Added $ADODB\_COUNTREC. Set to false for high speed selects.

Added Sybase support. Contributed by Toni Tunkkari (toni.tunkkari#finebyte.com). Bug in Sybase
API: GetFields is unable to determine date types.

Changed behaviour of RecordSet.GetMenu() to support size parameter (listbox) properly.

Added emptyDate and emptyTimeStamp to RecordSet class that defines how to represent
empty dates.

Added MetaColumns($table) that returns an array of ADOFieldObject's listing
the columns of a table.

Added transaction support for PostgresSQL -- thanks to "Eric G. Werk" egw#netguide.dk.

Added adodb-session.php for session support.

**0.80 30 Nov 2000**

Added support for charSet for interbase. Implemented MetaTables for most databases.
PostgreSQL more extensively tested.

**0.71 22 Nov 2000**

Switched from using require\_once to include/include\_once for backward compatability with PHP 4.02 and earlier.

**0.70 15 Nov 2000**

Calls by reference have been removed (call\_time\_pass\_reference=Off) to ensure compatibility with future versions of PHP,
except in Oracle 7 driver due to a bug in php\_oracle.dll.

PostgreSQL database driver contributed by Alberto Cerezal (acerezalp#dbnet.es).

Oci8 driver for Oracle 8 contributed by George Fourlanos (fou#infomap.gr).

Added *mysqlt* database driver to support MySQL 3.23 which has transaction
support.

Oracle default date format (DD-MON-YY) did not match ADOdb default date format (which is YYYY-MM-DD). Use ALTER SESSION to force the default date.

Error message checking is now included in test suite.

MoveNext() did not check EOF properly -- fixed.

**0.60 Nov 8 2000**

Fixed some constructor bugs in ODBC and ADO. Added ErrorNo function to ADOConnection
class.

**0.51 Oct 18 2000**

Fixed some interbase bugs.

**0.50 Oct 16 2000**

Interbase commit/rollback changed to be compatible with PHP 4.03.

CommitTrans( ) will now return true if transactions not supported.

Conversely RollbackTrans( ) will return false if transactions not supported.

**0.46 Oct 12**

Many Oracle compatibility issues fixed.

**0.40 Sept 26**

Many bug fixes

Now Code for BeginTrans, CommitTrans and RollbackTrans is working. So is the Affected\_Rows
and Insert\_ID. Added above functions to test.php.

ADO type handling was busted in 0.30. Fixed.

Generalised Move( ) so it works will all databases, including ODBC.

**0.30 Sept 18**

Renamed ADOLoadDB to ADOLoadCode. This is clearer.

Added BeginTrans, CommitTrans and RollbackTrans functions.

Added Affected\_Rows() and Insert\_ID(), \_affectedrows() and \_insertID(), ListTables(),
ListDatabases(), ListColumns().

Need to add New\_ID() and hasInsertID and hasAffectedRows, autoCommit

**0.20 Sept 12**

Added support for Microsoft's ADO.

Added new field to ADORecordSet -- canSeek

Added new parameter to \_fetch($ignore\_fields = false). Setting to true will
not update fields array for faster performance.

Added new field to ADORecordSet/ADOConnection -- dataProvider to indicate whether
a class is derived from odbc or ado.

Changed class ODBCFieldObject to ADOFieldObject -- not documented currently.

Added benchmark.php and testdatabases.inc.php to the test suite.

Added to ADORecordSet FastForward( ) for future high speed scrolling. Not documented.

Realised that ADO's Move( ) uses relative positioning. ADOdb uses absolute.

**0.10 Sept 9 2000**

First release
