# Supplementary material for: A UMLS-based spell checker for natural language processing in vaccine safety
Source: BMC Med Inform Decis Mak. 2007 Feb 12;7:3. doi: 10.1186/1472-6947-7-3 (PMC1805499; doi:10.1186/1472-6947-7-3)
Supplement: Additional file 13 — RAP application directory. Sets up directory for RAP (RDF application for PHP) source code [file 1472-6947-7-3-S13.gz › rap/api/util/adodb/docs/readme.htm]

ADODB Manual


### ADOdb Library for PHP

ADOdb is a suite of database libraries that allow you to connect to multiple
databases in a portable manner. Download from http://adodb.sourceforge.net/.

- The ADOdb documentation has moved to docs-adodb.htm
  This allows you to query, update and insert records using a portable API.

  - The ADOdb data dictionary docs are at docs-datadict.htm.
    This allows you to create database tables and indexes in a portable manner.

    - The ADOdb database performance monitoring docs are at docs-perf.htm.
      This allows you to perform health checks, tune and monitor your database.

      - The ADOdb database-backed session docs are at docs-session.htm.

### Installation

Make sure you are running PHP4.0.4 or later. Unpack all the files into a directory accessible by your webserver.

To test, try modifying some of the tutorial examples. Make sure you customize the connection settings correctly. You can debug using:

```
<?php
include('adodb/adodb.inc.php');

$db = ADONewConnection($driver); # eg. 'mysql' or 'oci8' 
$db->debug = true;
$db->Connect($server, $user, $password, $database);
$rs = $db->Execute('select * from some_small_table');
print "<pre>";
print_r($rs->GetRows());
print "</pre>";
?>
```

### How are people using ADOdb

Here are some examples of how people are using ADOdb:

- **PhpLens** is a commercial data grid component that allows
  both cool Web designers and serious unshaved programmers to develop and
  maintain databases on the Web easily. Developed by the author of ADOdb.
- **PHAkt**: PHP Extension for DreamWeaver Ultradev allows
  you to script PHP in the popular Web page editor. Database handling provided
  by ADOdb.
- **Analysis Console for Intrusion Databases (ACID)**: PHP-based
  analysis engine to search and process a database of security incidents
  generated by security-related software such as IDSes and firewalls (e.g.
  Snort, ipchains). By Roman Danyliw.
- **PostNuke** is a very popular free content management system
  and weblog system. It offers full CSS support, HTML 4.01 transitional
  compliance throughout, an advanced blocks system, and is fully multi-lingual
  enabled.
- **EasyPublish CMS** is another free content management system
  for managing information and integrated modules on your internet, intranet-
  and extranet-sites. From Norway.
- **NOLA** is a full featured accounting, inventory, and job
  tracking application. It is licensed under the GPL, and developed by Noguska.
