# Supplementary material for: A UMLS-based spell checker for natural language processing in vaccine safety
Source: BMC Med Inform Decis Mak. 2007 Feb 12;7:3. doi: 10.1186/1472-6947-7-3 (PMC1805499; doi:10.1186/1472-6947-7-3)
Supplement: Additional file 13 — RAP application directory. Sets up directory for RAP (RDF application for PHP) source code [file 1472-6947-7-3-S13.gz › rap/api/util/adodb/docs/tips_portable_sql.htm]

Tips on Writing Portable SQL for Multiple Databases for PHP


|  |  |
| --- | --- |
| Tips on Writing Portable SQL |  |

Updated 18 Sep 2003. Added Portable Native SQL section.

If you are writing an application that is used in multiple environments and
operating systems, you need to plan to support multiple databases. This article
is based on my experiences with multiple database systems, stretching from 4th
Dimension in my Mac days, to the databases I currently use, which are: Oracle,
FoxPro, Access, MS SQL Server and MySQL. Although most of the advice here applies
to using SQL with Perl, Python and other programming languages, I will focus on PHP and how
the ADOdb database abstraction library
offers some solutions.

Most database vendors practice product lock-in. The best or fastest way to
do things is often implemented using proprietary extensions to SQL. This makes
it extremely hard to write portable SQL code that performs well under all conditions.
When the first ANSI committee got together in 1984 to standardize SQL, the database
vendors had such different implementations that they could only agree on the
core functionality of SQL. Many important application specific requirements
were not standardized, and after so many years since the ANSI effort began,
it looks as if much useful database functionality will never be standardized.
Even though ANSI-92 SQL has codified much more, we still have to implement portability
at the application level.

### **Selects**

The SELECT statement has been standardized to a great degree. Nearly every
database supports the following:

SELECT [cols] FROM [tables]  
  [WHERE conditions]  
  [GROUP BY cols]  
  [HAVING conditions]   
  [ORDER BY cols]

But so many useful techniques can only be implemented by using proprietary
extensions. For example, when writing SQL to retrieve the first 10 rows for
paging, you could write...

|  |  |
| --- | --- |
| **Database** | **SQL Syntax** |
| DB2 | select \* from table fetch first 10 rows only |
| Informix | select first 10 \* from table |
| Microsoft SQL Server and Access | select top 10 \* from table |
| MySQL and PostgreSQL | select \* from table limit 10 |
| Oracle 8i | select \* from (select \* from table) where rownum <= 10 |

This feature of getting a subset of data is so useful that in the PHP class
library ADOdb, we have a SelectLimit( ) function that allows you to hide the
implementation details within a function that will rewrite your SQL for you:

```
$connection->SelectLimit('select * from table', 10);
```

**Selects: Fetch Modes**

PHP allows you to retrieve database records as arrays. You can choose to have
the arrays indexed by field name or number. However different low-level PHP
database drivers are inconsistent in their indexing efforts. ADOdb allows you
to determine your prefered mode. You set this by setting the variable $ADODB\_FETCH\_MODE
to either of the constants ADODB\_FETCH\_NUM (for numeric indexes) or ADODB\_FETCH\_ASSOC
(using field names as an associative index).

The default behaviour of ADOdb varies depending on the database you are using.
For consistency, set the fetch mode to either ADODB\_FETCH\_NUM (for speed) or
ADODB\_FETCH\_ASSOC (for convenience) at the beginning of your code.

**Selects: Counting Records**

Another problem with SELECTs is that some databases do not return the number
of rows retrieved from a select statement. This is because the highest performance
databases will return records to you even before the last record has been found.

In ADOdb, RecordCount( ) returns the number of rows returned, or will emulate
it by buffering the rows and returning the count after all rows have been returned.
This can be disabled for performance reasons when retrieving large recordsets
by setting the global variable $ADODB\_COUNTRECS = false. This variable is checked
every time a query is executed, so you can selectively choose which recordsets
to count.

If you prefer to set $ADODB\_COUNTRECS = false, ADOdb still has the PO\_RecordCount(
) function. This will return the number of rows, or if it is not found, it will
return an estimate using SELECT COUNT(\*):

```
$rs = $db->Execute("select * from table where state=$state");
$numrows = $rs->PO_RecordCount('table', "state=$state");
```

**Selects: Locking**

SELECT statements are commonly used to implement row-level locking of tables.
Other databases such as Oracle, Interbase, PostgreSQL and MySQL with InnoDB
do not require row-level locking because they use versioning to display data
consistent with a specific point in time.

Currently, I recommend encapsulating the row-level locking in a separate function,
such as RowLock($table, $where):

```
$connection->BeginTrans( );
$connection->RowLock($table, $where);
```

```
# some operation
```

```
if ($ok) $connection->CommitTrans( );
else $connection->RollbackTrans( );
```

**Selects: Outer Joins**

Not all databases support outer joins. Furthermore the syntax for outer joins
differs dramatically between database vendors. One portable (and possibly slower)
method of implementing outer joins is using UNION.

For example, an ANSI-92 left outer join between two tables t1 and t2 could
look like:

```
SELECT t1.col1, t1.col2, t2.cola   
  FROM t1 LEFT JOIN t2 ON t1.col = t2.col
```

This can be emulated using:

```
SELECT t1.col1, t1.col2, t2.cola FROM t1, t2   
	   WHERE t1.col = t2.col 
   UNION ALL
SELECT col1, col2, null FROM t1   
	   WHERE t1.col not in (select distinct col from t2)
```

Since ADOdb 2.13, we provide some hints in the connection object as to legal
join variations. This is still incomplete and sometimes depends on the database
version you are using, but is useful as a general guideline:

$conn->leftOuter: holds the
operator used for left outer joins (eg. '\*='), or false if not known or not
available.  
$conn->rightOuter: holds the
operator used for right outer joins (eg '=\*'), or false if not known or not
available.  
$conn->ansiOuter: boolean
that if true means that ANSI-92 style outer joins are supported, or false if
not known.

### **Inserts**

When you create records, you need to generate unique id's for each record.
There are two common techniques: (1) auto-incrementing columns and (2) sequences.

Auto-incrementing columns are supported by MySQL, Sybase and Microsoft Access
and SQL Server. However most other databases do not support this feature. So
for portability, you have little choice but to use sequences. Sequences are
special functions that return a unique incrementing number every time you call
it, suitable to be used as database keys. In ADOdb, we use the GenID( ) function.
It has takes a parameter, the sequence name. Different tables can have different
sequences.

```
$id = $connection->GenID('sequence_name');  
$connection->Execute("insert into table (id, firstname, lastname)   
			   values ($id, $firstname, $lastname)");
```

For databases that do not support sequences natively, ADOdb emulates sequences
by creating a table for every sequence.

### **Binding**

Binding variables in an SQL statement is another tricky feature. Binding is
useful because it allows pre-compilation of SQL. When inserting multiple records
into a database in a loop, binding can offer a 50% (or greater) speedup. However
many databases such as Access and MySQL do not support binding natively and
there is some overhead in emulating binding. Furthermore, different databases
(specificly Oracle!) implement binding differently. My recommendation is to
use binding if your database queries are too slow, but make sure you are using
a database that supports it like Oracle.

ADOdb supports portable Prepare/Execute with:

```
$stmt = $db->Prepare('select * from customers where custid=? and state=?');
$rs = $db->Execute($stmt, array($id,'New York'));
```

Oracle uses named bind placeholders, not "?", so to support portable binding, we have Param() that generates
the correct placeholder (available since ADOdb 3.92):

```
$sql = 'insert into table (col1,col2) values ('.$DB->Param('a').','.$DB->Param('b').')';
# generates 'insert into table (col1,col2) values (?,?)'
# or        'insert into table (col1,col2) values (:a,:b)'
$stmt = $DB->Prepare($sql);
$stmt = $DB->Execute($stmt,array('one','two'));
```

## Portable Native SQL

ADOdb provides the following functions for portably generating SQL functions
as strings to be merged into your SQL statements (some are only available since
ADOdb 3.92):

|  |  |
| --- | --- |
| **Function** | **Description** |
| DBDate($date) | Pass in a UNIX timestamp or ISO date and it will convert it to a date string formatted for INSERT/UPDATE |
| DBTimeStamp($date) | Pass in a UNIX timestamp or ISO date and it will convert it to a timestamp string formatted for INSERT/UPDATE |
| SQLDate($date, $fmt) | Portably generate a date formatted using $fmt mask, for use in SELECT statements. |
| OffsetDate($date, $ndays) | Portably generate a $date offset by $ndays. |
| Concat($s1, $s2, ...) | Portably concatenate strings. Alternatively, for mssql use mssqlpo driver, which allows || operator. |
| IfNull($fld, $replaceNull) | Returns a string that is the equivalent of MySQL IFNULL or Oracle NVL. |
| Param($name) | Generates bind placeholders, using ? or named conventions as appropriate. |
| $db->sysDate | Property that holds the SQL function that returns today's date |
| $db->sysTimeStamp | Property that holds the SQL function that returns the current timestamp (date+time). |
| $db->concat\_operator | Property that holds the concatenation operator |
| $db->length | Property that holds the name of the SQL strlen function. |
| $db->upperCase | Property that holds the name of the SQL strtoupper function. |
| $db->random | Property that holds the SQL to generate a random number between 0.00 and 1.00. |
| $db->substr | Property that holds the name of the SQL substring function. |

## DDL and Tuning

There are database design tools such as ERWin or Dezign that allow you to generate data definition language commands such as ALTER TABLE or CREATE INDEX from Entity-Relationship diagrams.

However if you prefer to use a PHP-based table creation scheme, adodb provides you with this feature. Here is the code to generate the SQL to create a table with:

1. Auto-increment primary key 'ID',
2. The person's 'NAME' VARCHAR(32) NOT NULL and defaults to '',
3. The date and time of record creation 'CREATED',
4. The person's 'AGE', defaulting to 0, type NUMERIC(16).

Also create a compound index consisting of 'NAME' and 'AGE':

```
$datadict = NewDataDictionary($connection);
$flds = " 
  ID I AUTOINCREMENT PRIMARY,
  NAME C(32) DEFAULT '' NOTNULL,
  CREATED T DEFTIMESTAMP,
  AGE N(16) DEFAULT 0
";
$sql1 = $datadict->CreateTableSQL('tabname', $flds);
$sql2 = $datadict->CreateIndexSQL('idx_name_age', 'tabname', 'NAME,AGE');
```

### Data Types

Stick to a few data types that are available in most databases. Char, varchar
and numeric/number are supported by most databases. Most other data types (including
integer, boolean and float) cannot be relied on being available. I recommend
using char(1) or number(1) to hold booleans.

Different databases have different ways of representing dates and timestamps/datetime.
ADOdb attempts to display all dates in ISO (YYYY-MM-DD) format. ADOdb also provides
DBDate( ) and DBTimeStamp( ) to convert dates to formats that are acceptable
to that database. Both functions accept Unix integer timestamps and date strings
in ISO format.

```
$date1 = $connection->DBDate(time( ));  
$date2 = $connection->DBTimeStamp('2002-02-23 13:03:33');
```

We also provide functions to convert database dates to Unix timestamps:

```
$unixts = $recordset->UnixDate('#2002-02-30#'); # MS Access date => unix timestamp
```

The maximum length of a char/varchar field is also database specific. You can
only assume that field lengths of up to 250 characters are supported. This is
normally impractical for web based forum or content management systems. You
will need to be familiar with how databases handle large objects (LOBs). ADOdb
implements two functions, UpdateBlob( ) and UpdateClob( ) that allow you to
update fields holding Binary Large Objects (eg. pictures) and Character Large
Objects (eg. HTML articles):

```
# for oracle 
$conn->Execute('INSERT INTO blobtable (id, blobcol) VALUES (1,empty_blob())'); 
$conn->UpdateBlob('blobtable','blobcol',$blobvalue,'id=1'); 
   
# non-oracle databases
$conn->Execute('INSERT INTO blobtable (id, blobcol) VALUES (1, null)'); 
$conn->UpdateBlob('blobtable','blobcol',$blobvalue,'id=1');
```

Null handling is another area where differences can occur. This is a mine-field,
because 3-value logic is tricky.

In general, I avoid using nulls except for dates and default all my numeric
and character fields to 0 or the empty string. This maintains consistency with
PHP, where empty strings and zero are treated as equivalent, and avoids SQL
ambiguities when you use the ANY and EXISTS operators. However if your database
has significant amounts of missing or unknown data, using nulls might be a good
idea.

ADOdb also supports a portable IfNull function, so you can define what to display
if the field contains a null.

### **Stored Procedures**

Stored procedures are another problem area. Some databases allow recordsets
to be returned in a stored procedure (Microsoft SQL Server and Sybase), and
others only allow output parameters to be returned. Stored procedures sometimes
need to be wrapped in special syntax. For example, Oracle requires such code
to be wrapped in an anonymous block with BEGIN and END. Also internal sql operators
and functions such as +, ||, TRIM( ), SUBSTR( ) or INSTR( ) vary between vendors.

An example of how to call a stored procedure with 2 parameters and 1 return
value follows:

```
	switch ($db->databaseType) {
	case 'mssql':
	  $sql = 'SP_RUNSOMETHING'; break;
	case 'oci8':
	  $sql = 
	  "declare RETVAL integer;begin :RETVAL := SP_RUNSOMETHING(:myid,:group);end;";
	  break;
	default:
	  die('Unsupported feature');
	}
	# @RETVAL = SP_RUNSOMETHING @myid,@group
	$stmt = $db->PrepareSP($sql);	  
	$db->Parameter($stmt,$id,'myid'); 
	$db->Parameter($stmt,$group,'group');
	# true indicates output parameter  
	$db->Parameter($stmt,$ret,'RETVAL',true); 
	$db->Execute($stmt);
```

As you can see, the ADOdb API is the same for both databases. But the stored
procedure SQL syntax is quite different between databases and is not portable,
so be forewarned! However sometimes you have little choice as some systems only
allow data to be accessed via stored procedures. This is when the ultimate portability
solution might be the only solution: *treating portable SQL as a localization
exercise...*

### **SQL as a Localization Exercise**

In general to provide real portability, you will have to treat SQL coding
as a localization exercise. In PHP, it has become common to define separate
language files for English, Russian, Korean, etc. Similarly, I would suggest
you have separate Sybase, Intebase, MySQL, etc files, and conditionally include
the SQL based on the database. For example, each MySQL SQL statement would be
stored in a separate variable, in a file called 'mysql-lang.inc.php'.

```
$sqlGetPassword = 'select password from users where userid=%s';
$sqlSearchKeyword = "SELECT * FROM articles WHERE match (title,body) against (%s)";
```

In our main PHP file:

```
# define which database to load...
$database = 'mysql';
include_once("$database-lang.inc.php");

$db = &NewADOConnection($database);
$db->PConnect(...) or die('Failed to connect to database');

# search for a keyword $word
$rs = $db->Execute(sprintf($sqlSearchKeyWord,$db->qstr($word)));
```

Note that we quote the $word variable using the qstr( ) function. This is because
each database quotes strings using different conventions.

### Final Thoughts

The best way to ensure that you have portable SQL is to have your data tables designed using
sound principles. Learn the theory of normalization and entity-relationship diagrams and model
your data carefully. Understand how joins and indexes work and how they are used to tune performance.

Visit the following page for more references on database theory and vendors:
http://php.weblogs.com/sql\_tutorial.
Also read this article on Optimizing PHP.

(c) 2002-2003 John Lim.
