# Supplementary material for: A UMLS-based spell checker for natural language processing in vaccine safety
Source: BMC Med Inform Decis Mak. 2007 Feb 12;7:3. doi: 10.1186/1472-6947-7-3 (PMC1805499; doi:10.1186/1472-6947-7-3)
Supplement: Additional file 13 — RAP application directory. Sets up directory for RAP (RDF application for PHP) source code [file 1472-6947-7-3-S13.gz › rap/api/util/adodb/docs/tute.htm]

Tutorial: Moving from MySQL to ADODB


# Tutorial: Moving from MySQL to ADODB

```
		You say eether and I say eyether, 
		You say neether and I say nyther; 
		Eether, eyether, neether, nyther - 
		Let's call the whole thing off ! 
  

		You like potato and I like po-tah-to, 
		You like tomato and I like to-mah-to; 
		Potato, po-tah-to, tomato, to-mah-to - 
		Let's call the whole thing off !
```

I love this song, especially the version with Louis Armstrong and Ella singing
duet. It is all about how hard it is for two people in love to be compatible
with each other. It's about compromise and finding a common ground, and that's
what this article is all about.

PHP is all about creating dynamic web-sites with the least fuss and the most
fun. To create these websites we need to use databases to retrieve login information,
to splash dynamic news onto the web page and store forum postings. So let's
say we were using the popular MySQL database for this. Your company has done
such a fantastic job that the Web site is more popular than your wildest dreams.
You find that MySQL cannot scale to handle the workload; time to switch databases.

Unfortunately in PHP every database is accessed slightly differently. To connect
to MySQL, you would use *mysql\_connect()*; when you decide to upgrade to
Oracle or Microsoft SQL Server, you would use *ocilogon()* or *mssql\_connect()*
respectively. What is worse is that the parameters you use for the different
connect functions are different also.. One database says po-tato, the other
database says pota-to. Oh-oh.

### Let's NOT call the whole thing off

A database wrapper library such as ADODB comes in handy when you need to ensure portability. It provides
you with a common API to communicate with any supported database so you don't have to call things off.

ADODB stands for Active Data Objects DataBase (sorry computer guys are sometimes
not very original). ADODB currently supports MySQL, PostgreSQL, Oracle, Interbase,
Microsoft SQL Server, Access, FoxPro, Sybase, ODBC and ADO. You can download
ADODB from http://php.weblogs.com/adodb.

### MySQL Example

The most common database used with PHP is MySQL, so I guess you should be familiar
with the following code. It connects to a MySQL server at *localhost*,
database *mydb*, and executes an SQL select statement. The results are
printed, one line per row.

```
$db = mysql_connect("localhost", "root", "password");
mysql_select_db("mydb",$db);
$result = mysql_query("SELECT * FROM employees",$db);
if ($result === false) die("failed"); 
while ($fields = mysql_fetch_row($result)) {
 for ($i=0, $max=sizeof($fields); $i < $max; $i++) {
		print $fields[$i].' ';
 }
 print "<br>\n";
}
```

The above code has been color-coded by section. The first section is the connection
phase. The second is the execution of the SQL, and the last section is displaying
the fields. The *while* loop scans the rows of the result, while the *for*
loop scans the fields in one row.

Here is the equivalent code in ADODB

```
 include("adodb.inc.php");
 $db = NewADOConnection('mysql');
 $db->Connect("localhost", "root", "password", "mydb");
 $result = $db->Execute("SELECT * FROM employees");
 if ($result === false) die("failed");  
 while (!$result->EOF) {
	for ($i=0, $max=$result->FieldCount(); $i < $max; $i++)
		   print $result->fields[$i].' ';
	$result->MoveNext();
	print "<br>\n";
 }
```

Now porting to Oracle is as simple as changing the second line to `NewADOConnection('oracle')`.
Let's walk through the code...

### Connecting to the Database

```
include("adodb.inc.php");
$db = NewADOConnection('mysql');
$db->Connect("localhost", "root", "password", "mydb");
```

The connection code is a bit more sophisticated than MySQL's because our needs
are more sophisticated. In ADODB, we use an object-oriented approach to managing
the complexity of handling multiple databases. We have different classes to
handle different databases. If you aren't familiar with object-oriented programing,
don't worry -- the complexity is all hidden away in the `NewADOConnection()`
function.

To conserve memory, we only load the PHP code specific to the database you
are connecting to. We do this by calling `NewADOConnection(databasedriver)`.
Legal database drivers include *mysql, mssql, oracle, oci8, postgres, sybase,
vfp, access, ibase* and many others.

Then we create a new instance of the connection class by calling `NewADOConnection()`.
Finally we connect to the database using `$db->Connect().`

### Executing the SQL

`$result = $db->Execute("SELECT *
FROM employees");  
if ($result === false) die("failed")``;`

Sending the SQL statement to the server is straight forward. Execute() will
return a recordset object on successful execution. You should check $result
as we do above.

An issue that confuses beginners is the fact that we have two types of objects
in ADODB, the connection object and the recordset object. When do we use each?

The connection object ($db) is responsible for connecting to the database,
formatting your SQL and querying the database server. The recordset object ($result)
is responsible for retrieving the results and formatting the reply as text or
as an array.

The only thing I need to add is that ADODB provides several helper functions
for making INSERT and UPDATE statements easier, which we will cover in the Advanced
section.

### Retrieving the Data

```
while (!$result->EOF) {
   for ($i=0, $max=$result->FieldCount(); $i < $max; $i++)
	   print $result->fields[$i].' ';
   $result->MoveNext();
   print "<br>\n";
}
```

The paradigm for getting the data is that it's like reading a file. For every
line, we check first whether we have reached the end-of-file (EOF). While not
end-of-file, loop through each field in the row. Then move to the next line
(MoveNext) and repeat.

The `$result->fields[]` array is generated by the PHP database
extension. Some database extensions do not index the array by field name.
To force indexing by name - that is associative arrays -
use the $ADODB\_FETCH\_MODE global variable.

```
	$ADODB_FETCH_MODE = ADODB_FETCH_NUM;
	$rs1 = $db->Execute('select * from table');
	$ADODB_FETCH_MODE = ADODB_FETCH_ASSOC;
	$rs2 = $db->Execute('select * from table');
	print_r($rs1->fields); // shows array([0]=>'v0',[1] =>'v1')
	print_r($rs2->fields); // shows array(['col1']=>'v0',['col2'] =>'v1')
```

As you can see in the above example, both recordsets store and use different fetch modes
based on the $ADODB\_FETCH\_MODE setting when the recordset was created by Execute().

## ADOConnection

Object that performs the connection to the database, executes SQL statements
and has a set of utility functions for standardising the format of SQL statements
for issues such as concatenation and date formats.

### Other Useful Functions

`$recordset->Move($pos)` scrolls to that particular row. ADODB supports forward
scrolling for all databases. Some databases will not support backwards scrolling.
This is normally not a problem as you can always cache records to simulate backwards
scrolling.

`$recordset->RecordCount()` returns the number of records accessed by the
SQL statement. Some databases will return -1 because it is not supported.

`$recordset->GetArray()` returns the result as an array.

`rs2html($recordset)` is a function that is generates a HTML table based on the
$recordset passed to it. An example with the relevant lines in bold:

```
   include('adodb.inc.php'); 
   include('tohtml.inc.php'); /* includes the rs2html function */
   $conn = &ADONewConnection('mysql'); 
   $conn->PConnect('localhost','userid','password','database');
   $rs = $conn->Execute('select * from table');
   rs2html($rs); /* recordset to html table */
```

There are many other helper functions that are listed in the documentation available at http://php.weblogs.com/adodb\_manual.

## Advanced Material

### Inserts and Updates

Let's say you want to insert the following data into a database.

**ID** = 3  
**TheDate**=mktime(0,0,0,8,31,2001) /\* 31st August 2001 \*/  
**Note**= sugar why don't we call it off

When you move to another database, your insert might no longer work.

The first problem is that each database has a different default date format.
MySQL expects YYYY-MM-DD format, while other databases have different defaults.
ADODB has a function called DBDate() that addresses this issue by converting
converting the date to the correct format.

The next problem is that the **don't** in the Note needs to be quoted. In
MySQL, we use **don\'t** but in some other databases (Sybase, Access, Microsoft
SQL Server) we use **don''t.** The qstr() function addresses this issue.

So how do we use the functions? Like this:

```
$sql = "INSERT INTO table (id, thedate,note) values (" 
   . $ID . ','
   . $db->DBDate($TheDate) .','
   . $db->qstr($Note).")";
$db->Execute($sql);
```

ADODB also supports `$connection->Affected_Rows()` (returns the
number of rows affected by last update or delete) and `$recordset->Insert_ID()`
(returns last autoincrement number generated by an insert statement). Be forewarned
that not all databases support the two functions.

### MetaTypes

You can find out more information about each of the fields (I use the words
fields and columns interchangebly) you are selecting by calling the recordset
method `FetchField($fieldoffset)`. This will return an object with
3 properties: name, type and max\_length.

```
For example:
```

```
$recordset = $conn->Execute("select adate from table");  
$f0 = $recordset->FetchField(0);
```

Then `$f0->name` will hold *'adata'*, `$f0->type`
will be set to '*date'*. If the max\_length is unknown, it will be set to
-1.

One problem with handling different databases is that each database often calls
the same type by a different name. For example a *timestamp* type is called
*datetime* in one database and *time* in another. So ADODB has a special
`MetaType($type, $max_length)` function that standardises the types
to the following:

C: character and varchar types  
X: text or long character (eg. more than 255 bytes wide).  
B: blob or binary image  
D: date  
T: timestamp  
L: logical (boolean)  
I: integer  
N: numeric (float, double, money)

In the above date example,

`$recordset = $conn->Execute("select adate from table");  
$f0 = $recordset->FetchField(0);  
$type = $recordset->MetaType($f0->type, $f0->max_length);  
print $type; /* should print 'D'` \*/

**Select Limit and Top Support**

ADODB has a function called $connection->SelectLimit($sql,$nrows,$offset) that allows
you to retrieve a subset of the recordset. This will take advantage of native
SELECT TOP on Microsoft products and SELECT ... LIMIT with PostgreSQL and MySQL, and
emulated if the database does not support it.

**Caching Support**

ADODB allows you to cache recordsets in your file system, and only requery the database
server after a certain timeout period with $connection->CacheExecute($secs2cache,$sql) and
$connection->CacheSelectLimit($secs2cache,$sql,$nrows,$offset).

**PHP4 Session Handler Support**

ADODB also supports PHP4 session handlers. You can store your session variables
in a database for true scalability using ADODB. For further information, visit
http://php.weblogs.com/adodb-sessions

### Commercial Use Encouraged

If you plan to write commercial PHP applications that you want to resell, you should consider ADODB. It has been released using the lesser GPL, which means you can legally include it in commercial applications, while keeping your code proprietary. Commercial use of ADODB is strongly encouraged! We are using it internally for this reason.

## Conclusion

As a thank you for finishing this article, here are the complete lyrics for
*let's call the whole thing off*.  
  

```
   Refrain 
  

		You say eether and I say eyether, 
		You say neether and I say nyther; 
		Eether, eyether, neether, nyther - 
		Let's call the whole thing off ! 
  

		You like potato and I like po-tah-to, 
		You like tomato and I like to-mah-to; 
		Potato, po-tah-to, tomato, to-mah-to - 
		Let's call the whole thing off ! 
  

But oh, if we call the whole thing off, then we must part. 
And oh, if we ever part, then that might break my heart. 
  

		So, if you like pajamas and I like pa-jah-mas, 
		I'll wear pajamas and give up pa-jah-mas. 
		For we know we 
		Need each other, so we 
		Better call the calling off off. 
		Let's call the whole thing off ! 
  

   Second Refrain 
  

		You say laughter and I say lawfter, 
		You say after and I say awfter; 
		Laughter, lawfter, after, awfter - 
		Let's call the whole thing off ! 
  

		You like vanilla and I like vanella, 
		You, sa's'parilla and I sa's'parella; 
		Vanilla, vanella, choc'late, strawb'ry - 
		Let's call the whole thing off ! 
  

But oh, if we call the whole thing off, then we must part. 
And oh, if we ever part, then that might break my heart. 
  

		So, if you go for oysters and I go for ersters, 
		I'll order oysters and cancel the ersters. 
		For we know we 
		Need each other, so we 
		Better call the calling off off. 
		Let's call the whole thing off !
```

Song and lyrics by George and Ira Gershwin, introduced by Fred Astaire and Ginger Rogers
in the film "Shall We Dance?" 

(c)2001-2002 John Lim.
